# Host genetic regulation of rumen 6-hydroxymelatonin reduces methane emissions in dairy cattle

**DOI:** 10.1073/pnas.2604454123

**Published:** 2026-06-08

**Authors:** Chenguang Zhang, Ye Liu, Guoyan Wang, Mengqi Huang, Jun Zhang, Zongjun Li, Linhao Zhou, Guangfu Tang, Shengru Wu, Lu Deng, Junhu Yao

**Affiliations:** ^a^ Department of Animal Nutrition and Environmental Hygiene, College of Animal Science and Technology, Northwest A&F University, Yangling 712100, Shaanxi, China; ^b^National Center of Technology Innovation for Dairy, Inner Mongolia Dairy Technology Research Institute Co. Ltd, Hohhot 010100, Inner Mongolia, China

**Keywords:** dairy cattle, host genetic, rumen microbiota, 6-hydroxymelatonin, methane mitigation

## Abstract

The mechanisms linking host genetics to ruminal methane emissions remain unclear. Here, we integrated multiomics data from 304 lactating cows and demonstrated that methane emission per dry matter intake (M/D) exhibitd higher heritability (*h^2^* = 0.42) than microbiability (*m^2^* = 0.19), highlighting the predominant role of host genetics. Mendelian randomization (MR) analysis identified three heritable *Prevotella* species (including *Prevotella_bryantii*) that causally reduce methane emissions. Network suggested that *Prevotella_bryantii*, which harbors the [NiFe]_Group_1d hydrogenase, exerts this effect by competing with methanogens for H₂. Furthermore, the methane-reducing effect of *Prevotella _bryantii* was confirmed by in vitro fermentation experiments. To trace the host regulation upstream, the host-derived metabolite 6-hydroxymelatonin was identified as a key regulator that positively influences these *Prevotella* species by MR analysis, which was further validated by in vitro fermentation and pure bacterial culture experiments. Genome-wide association studies linked ruminal 6-hydroxymelatonin levels to host genetic variants (e.g., 5:106926534) near candidate genes including *ITFG*2. Functional studies in bovine hepatocytes revealed that *ITFG*2 knockdown activated the mTORC1 pathway, upregulated *CYP*1*A*2 expression, and increased 6-hydroxymelatonin synthesis. Furthermore, cattle carrying the TA genotype at 5:106926534 exhibited significantly lower predicted and measured methane emissions. Collectively, this study unveils a pathway whereby host genetics (via *ITFG*2/mTORC1) modulate hepatic 6-hydroxymelatonin synthesis, which enriches specific rumen *Prevotella* that compete with methanogens for hydrogen, thereby reducing methane.

## Significance

This study reveals a host-genetic pathway that regulates rumen microbial fermentation to mitigate methane emissions from livestock—a major contributor to climate change. We demonstrate how specific cow genes influence the production of a liver metabolite (6-hydroxymelatonin), which enriches *Prevotella_bryantii* in the rumen. These bacteria compete with methanogenic archaea, effectively reducing emissions. This host-driven mechanism provides concrete genetic and metabolic targets for breeding or dietary interventions, offering a sustainable strategy to lower the environmental footprint of dairy farming without compromising productivity.

Methane (CH^4^) is a potent greenhouse gas, and rumen fermentation in ruminants, particularly dairy cattle, represents a major anthropogenic source (1). Moreover, for every mole of methane produced, 2 to 12% of dietary energy is lost, reducing feed efficiency (2). While nutritional strategies have been the mainstay of mitigation efforts, they often face trade offs such as compromised lactation performance, increased costs, or diminished long-term efficacy (3, 4). A growing body of evidence indicates that methane emission is a moderately heritable trait (5), suggesting that host genetics may offer a sustainable avenue for genetic selection of low-methane-emitting cattle. Therefore, understanding how host genetic variation influences methane emissions is a critical step toward developing effective mitigation strategies.

To unravel the effects of host genetic on methane emissions, it is essential to understand how host genetics shapes the rumen microbiota, as methane is a direct metabolic product of rumen microbiota. Recent studies in ruminants have identified heritable rumen bacteria and host genetic variants associated with microbial composition. Genome-wide association studies (GWAS) have mapped single nucleotide polymorphisms (SNPs) associated with heritable rumen microbes to genes potentially involved in rumen epithelial function [*HSD17B4* regulating pH (6)], ion transport [*SLC30A9* affecting zinc homeostasis (7)], and immune regulation [*TOX* modulating T cell immunity (8)]. However, the regulatory pathways in these studies remain largely unclear. A complete pathway from a specific host genetic variant to a specific rumen microbial feature and methane emissions has yet to be established. Classic studies in humans and pigs have demonstrated that host genetics can shape the gut microbiota through host-derived metabolites. For example, a deletion in the pig ABO gene reduces intestinal N-acetylgalactosamine levels, thereby suppressing GalNAc-dependent bacteria (9). In humans, the LCT gene variant (rs4988235) influences *Bifidobacterium* abundance in lactose-intolerant individuals by altering lactose availability (10). These examples highlight that identifying the host-derived metabolites mediating host–microbe interactions is key to dissecting causal pathways (11). In ruminants, such metabolite-mediated regulatory mechanisms remain largely unexplored. Mendelian randomization (MR) analysis, which has a higher evidence level than correlation analysis but is lower than randomized controlled trials (12), is an effective analytical method that utilizes host SNPs as instrumental variables in large cohort studies to explore the relationship between microbes and the host’s physiology. However, since it requires host SNPs as instrumental variables, this method is particularly suitable for investigating host genetic regulation of rumen microbial methane emissions.

In this study, we will utilize whole-genome resequencing, rumen metagenomic, and metabolomic data from 304 Holstein cattle to first investigate the associations between methane emissions and lactation performance, rumen environment at both host genetic and rumen microbial levels. Subsequently, based on MR and GWAS, the host genetic regulatory mechanism of methane emissions involving “host SNP variation–host-derived metabolites–heritable rumen microbes” was explored, and validation was conducted through in vitro fermentation, pure bacterial culture, and molecular cell experiments.

## 1. Materials and Methods

### 1.1. Animals, Phenotypic Data, and Sample Collection.

As reported by our previous study (7), a total of 304 lactating Holstein dairy cattle with the same dietary and similar physiological state were selected in our study. The methane emission to dry matter intake ratio (M/D) of dairy cattle was predicted by the previously constructed artificial neural network model (Training set: *R^2^* = 0.62, RMSE = 60.01; Validation set: *R^2^* = 0.61, RMSE = 54.52) (3).

### 1.2. Metagenome Sequencing.

DNA extraction, library construction, alignment, and annotation of metagenomic data from rumen contents were performed as described in previous study (7). The nonredundant gene catalog was aligned to the NR database and HydDB (13) with 1e^−5^ by Diamond. The microbiability (*m^2^*) and the microbial correlation for rumen microbes, rumen metabolites, and phenotypes was estimated based microbial relationship matrix (MRM) (*SI Appendix*, Fig. S1*A*) by GCTA (14) (https://yanglab.westlake.edu.cn/software/gcta), and using the population structure (*SI Appendix*, Fig. S1*B*), days in milk and parity as the covariate. The construction of the MRM uses the top 100 rumen microbes based on CLR-normalized relative abundance (15). Using metagenome-wide analysis (MWAS) with M/D as the phenotype to explore methane emission-related microbes among the top 100 species-level microbes by relative abundance, with specific models referenced (8). The *P*-values obtained from the MWAS were uniformly corrected using the false discovery rate (*FDR*)

### 1.3. Metabolomic Analysis.

Metabolite extraction, LC–MS analysis, and data processing for the rumen content metabolome were performed as described in previous study (7). The final data were analyzed using MetOrigin (16) (http://metorigin.met-bioinformatics.cn/) to trace the origins of rumen metabolites, classifying them into categories of host, microbial, dietary, and environmental sources.

### 1.4. Whole-Genome Resequencing.

Host whole blood genomic DNA extraction, alignment, sorting, deduplication, SNP variant detection, and quality control were performed as described in previous studies (7). Ultimately, 2,337,054 SNPs from 304 dairy cattle across 30 chromosomes were obtained for analysis.

The heritability (*h^2^*) and the genetic correlation for rumen microbes, rumen metabolites, and phenotypes was estimated based genetic relationship matrix (GRM) (*SI Appendix*, Fig. S1*C*) by GCTA (14) (https://yanglab.westlake.edu.cn/software/gcta), and using the population structure (*SI Appendix*, Fig. S1*D*), days in milk and parity as the covariate. Microbial heritability reflects the extent to which host genetics influences the gut microbiota, or the similarity of microbiota among related individuals (17). Heritable microbes are defined as those with relatively high host genetic influence (*h*^2^ ≥ 0.2).

### 1.5. Mendelian Randomization.

To investigate the causal relationship between methane, rumen metabolites, and rumen microbes. We used the 123 heritable rumen metabolites and the top 100 rumen heritable microbes with the relative abundance as exposure. The M/D were used as the outcome. For the exposure group, the mixed linear model (MLM) was conducted with 166 samples from all 304 dairy cattle using GEMMA with the command “-r2 0.3” as independence settings. A total of 899,623 independent SNP (linkage disequilibrium) was used to filter instrumental variables with 1e^-5^ as significance threshold. For the outcome group, the MLM was also conducted with 152 samples from all 304 dairy cattle using GEMMA. A total of 2,440,076 SNPs were obtained to match instrumental variables filtered by exposure. The sample overlap rate between the exposure group and outcome group was 4.4%.

After determining the instrumental variables, the MR analysis was conducted using TwoSampleMR (version 0.5.6) in the R project, employing five different methods: inverse variance weighting (IVW), MR-Egger regression, weighted median, simple mode, and weighted mode. IVW combines the effect estimates of each genetic variant, weighting them by the inverse of their variance to obtain a meta-analytic estimate of the causal effect. MR-Egger regression is used to assess the presence of directional pleiotropy and provides a causal estimate adjusted for this bias. Weighted median calculates a consistent estimate based on the median, even if up to 50% of the genetic variants are invalid instruments. Both simple mode and weighted mode of the mode-based methods were used, which cluster genetic variants into groups with similar causal estimates and derive an overall causal effect from the largest cluster. In cases where there is disagreement in the results between the models, IVW mode is used as the evaluation criterion, and a pleiotropy test is performed (18–20). The *P*-values obtained from the IVW model were uniformly corrected using the *FDR*.

### 1.6. Genome-Wide Association Studies.

The MLM was established to identify significant SNPs for 6-hydroxylmelatonin, a kinship matrix was established as the random effect using GEMMA (21), and the population structure was established as the fixed effect using PLINK. The significant SNPs were detected using a MLM with the GEMMA. Subsequently, we set the genome-wide significance threshold based on a significance level of 0.05/nSNPs [0.05/2337054 = 2.14E−08, −log10(*P*) = 7.67] for significant associations. The Variant Effect Predictor (https://www.ensembl.org/vep) was used for gene annotation.

### 1.7. Structure Variations.

structural variations (SVs) are variable genomic segments that may be absent in some individuals and present at varying abundances in others. Using cleaned metagenomic reads, we detected microbial SVs with SGV-Finder (v.1) (22), which identifies two types: variable SVs (vSVs) and deletion SVs (dSVs). The process includes three main steps: 1. The metagenomic reads after quality control with fastp were aligned to the reference database provided by SGV-Finder, which is based on the proGenomes database (http://progenomes1.embl.de/) (23) using GEM mapper. 2. The ambiguous reads after aligning were reassigned to the most likely reference with high accuracy according to the mapping quality and genomic coverage by Iterative coverage-based read assignment (ICRA). 3. On a group level, detect genome fragments with highly variable standardization coverage. For the identification of dSVs within each species, genomic bins are classified as either deleted (with coverage close to 0) or retained (with coverage close to the median coverage of the genome) bins in each sample. Bins that are deleted in 25 to 75% of samples are considered as raw dSVs and included in the analysis. Raw dSVs that show high co-occurrence correlation are then merged to form larger SV regions, resulting in the final dSV profile. To identify vSVs within each species, the coverage of genomic bins in each sample is standardized using the Z-score approach. Each bin is evaluated across all samples, and bins that exhibit high variability based on a β’ distribution are considered as raw vSVs. Raw vSVs with high correlation in standardized coverage are further merged to form larger SV regions, generating the final vSV profile.

### 1.8. Structural Equation Modeling.

Structural equation modeling (SEM) was constructed to evaluate the effect of 6-hydroxylmelatonin on reducing methane emissions through rumen microbiota. The key microbes (*Prevotella_bryantii*, *Prevotella_mizrahii*, and *Prevotella_sp.*_AGR2160) for methane emission reduction obtained based on MR analysis were converted into latent variables in SEM. The goodness-of-fit of the SEM was checked using the χ2 test, the rootmean square error (RMSE), and the comparative fit index (CFI). The model had a good fit when the CFI value was close to 1 and RMSE was close to 0 (24). SEM was conducted using the lavaan package (25).

### 1.9. Network.

The microbial interaction network was constructed based on all *Methanobrevibacter* in the metagenome, The key microbes (*Prevotella_bryantii*, *Prevotella_mizrahii*, and *Prevotella_sp._*AGR2160) for methane emission reduction obtained based on MR analysis and hydrogenase using the R package ggClusterNet (26).

### 1.10. Rumen In Vitro Fermentation Experiment.

To validate the impact of the screened rumen microbes and metabolites on methane emissions, a static rumen fermentation system was established following the protocol of Li et al. (27). Briefly, a 60 mL system (20 mL rumen fluid + 40 mL artificial saliva) was incubated with 0.5 g TMR substrate for 6 h at 39 °C. Treatments included *Prevotella_bryantii*_B14 (10^8^ cfu/mL), melatonin (0.1 and 1 mM), and 6-hydroxymelatonin (0.1 and 1 mM). Gas production, fermentation parameters, and microbial absolute quantification were measured. *Prevotella_bryantii*_B14 was purchased from Huizao Biotechnology (Wuhan, China, isolated from dairy cattle rumen, NCBI Taxonomy ID: 77095). melatonin and 6-hydroxylmelatonin were purchased from Macklin Reagent (Shanghai, China, CAS numbers: 66521-38-8 and 2208-41-5, respectively).

### 1.11. 16S rDNA Quantitative PCR.

Genomic DNA was extracted via repeated bead beating. Absolute quantification of *Prevotella_bryantii* and *Methanobrevibacter* was performed by qPCR using specific primers and SYBR Green Master Mix in 25 µL reactions. Standard curves were generated from serial dilutions of purified 16S rDNA PCR products. Data are expressed as lg (16S rDNA copies per mL of content). The 16S rDNA-specific primers were used: *SI Appendix*, Table S1.

### 1.12. Culture Rumen-Derived Prevotella_bryantii_B14.

As mentioned previously, *Prevotella_bryantii*_B14 was purchased from Huizao Biotechnology. Its resuscitation and culture protocol was carried out according to the DSMZ protocols (https://www.dsmz.de/). Based on the existing culture protocol, experimental groups were set up with additional supplementation of 1.0, 1.5, and 2.0 mM of 6-hydroxymelatonin, respectively. The optical density at 600 nm (OD_600_) of the culture broth was measured every 30 min using an Alto microbial growth curve analyzer (Cerillo, TX) for a total cultivation period of 600 min, ultimately generating the growth curve.

### 1.13. Cell Culture and Treatment.

Bovine hepatocyte cell line were immortal lines and cultured in Dulbecco’s Modified Eagle Medium (Gibco) supplemented with 10% fetal bovine serum (Gibco, Grand Island) and 1% penicillin-streptomycin (Solarbio, Beijing, China) at 37 °C in a 5% CO_2_ atmosphere. The medium was changed every 24 h.

Rapamycin (V900930, a selective mTOR inhibitor), obtained from Sigma-Aldrich, was dissolved in DMSO to prepare a stock solution. Prior to treatment, cells were seeded into appropriate plates and allowed to adhere overnight. Cells were then treated with Rapamycin at 10 nM concentrations for 12 h. Control groups received an equal volume of DMSO vehicle, for all experiments of hepatocyte cells, both the treatment and control groups were set up in triplicate (n = 3) to ensure biological reproducibility.

#### 1.13.1. siRNA transfection.

siRNAs against genes *NCAM*2, *PLXNA*2, *AGMO*, *NRIP*2, and *ITFG*2 found in GWAS for 6-hydroxymelatonin (GenePharma, Shanghai, China), and Lipofectamine 2000 (Invitrogen, CA) were mixed and incubated in serum-free medium for 30 min. After 6 h, the serum-free medium was replaced by complete medium. The siRNAs were used: *SI Appendix*, Table S1.

#### 1.13.2. Western blotting.

The protein of cells was extracted as previously described (28). The antibodies against S6 (2217S) and p-S6 (4858S) were obtained from Cell Signaling Technology. Actin (66009-1-Ig) antibodies were obtained from Proteintech. Secondary antibodies were purchased from Sigma-Aldrich. Primary and secondary antibodies were diluted at 1:1,000 to 1:2,000 and 1:5,000, respectively. Following PBST washes, the membranes were incubated at room temperature with horseradish peroxidase (HRP)-conjugated secondary antibodies at a 1:5,000 dilution. After another wash step, protein bands were visualized using a Bio-Rad imaging system. Band intensities were quantified with Image-Pro Plus software (Media Cybernetics), normalized to Actin as the internal control, and expressed as relative protein abundance.

#### 1.13.3. qRT-PCR.

Total RNA was extracted from primary hepatocytes with TRIzol reagent (DP424, TIANGEN) according to the manufacturer’s instructions. RNA concentration and purity were assessed using a NanoDrop ND-1000 Spectrophotometer (Thermo Fisher Scientific), with A260/A280 and A260/A230 ratios ranging between 2.0 to 2.1 and 2.0 to 2.2, respectively. RNA integrity was verified by denaturing agarose gel electrophoresis. Subsequently, cDNA was synthesized from total RNA using the HiScript II 1st Strand cDNA Synthesis Kit (R212-02, Vazyme). Quantitative real-time PCR (qRT-PCR) was carried out in technical triplicates on a Roche LightCycler 96 system with ChamQ Universal SYBR qPCR Master Mix (Q711-02, Vazyme). The reaction protocol consisted of an initial step at 95 °C for 30 s, followed by 40 cycles of 95 °C for 10 s and 60 °C for 30 s. Relative mRNA expression levels of target genes were determined via the comparative Ct method (2^−ΔΔCt^) (29), normalized to β-actin and expressed as fold changes relative to the calibrator sample. The gene-specific primers were used: *SI Appendix*, Table S1.

#### 1.13.4. Measuring 6-hydroxymelatonin concentration.

6-hydroxymelatonin level was measured using ultraperformance liquid chromatography-mass spectrometry (UPLC-MS). Briefly, the 50 mg of N-Propyl Ethylenediamine (PSA) was added to 2 mL of cell culture medium and vortex thoroughly. After centrifugation at 10,000 rpm for 5 min, the supernatant was collected and filtered through a 0.45 µm membrane. The standard 6-hydroxymelatonin (2208-41-5, Sigma-Aldrich) was formulated into different concentration gradients of 0, 100, 250, 500, 1000, 2000 ng/mL for establishing a standard curve. Total 5 μL volumes sample was injected into the UPLC-MS system (AB Sciex) and equipped with a C18 column (100 mm × 2.1 mm×1.8 μm, Thermo) maintained at 30 °C by gradient elution with a mobile phase flow rate of 0.3 mL/min. Gradient elution mobile phase was consisted of A (0.1% formic acid water) and B (acetonitrile).

### 1.14. Methane Emission Measurement.

To validate the function of the 5:106926534 locus, 31 healthy dairy cattle were selected from a different farm unrelated to the aforementioned experimental herd of 304 cattle, based on similar parity and days in milk. The high-throughput Hi-SNP genotyping approach was used to determine the 5:106926534 genotype of each cattle (30). Briefly, Blood genomic DNA was extracted from cattle blood samples. Target SNP locus 5:106926534 was amplified using primers F: AAAATACTTTCACAGCAATGTCTG and R: AGAAGAAGATTGTAGTTTCTGTGC in a multiplex PCR system. Amplicon quality was checked by agarose gel electrophoresis. Libraries were constructed using a barcoding PCR approach, pooled, purified, and subjected to high-throughput sequencing on an Illumina X-10 for genotype calling.

Subsequently, methane emissions from these 31 lactating cattle were measured using the automated head-chamber (AHC) system. All cattle were designated as a single measurement group and were guided to a dedicated exercise area equipped with the AHC system during fixed daily time slots (10:00 to 12:00 and 18:00 to 21:00) for 14 consecutive days. Throughout this process, each cattle was ensured equal opportunity to access the device during each time slot to obtain valid individual data on methane (CH^4^ g/d) emissions, while related dry matter intake (DMI, kg/d) data were recorded simultaneously. Detailed methods refer to Wang et al. (31).

## 2. Results

### 2.1. predicted Methane Emissions From 304 Dairy Cattle and Their Spearman, Microbial, and Genetic Correlations With Phenotypes.

In order to obtain the individual value of methane emissions of dairy cattle, we used the previously constructed artificial neural network model (3) for predicting methane emissions and combined the model with the dietary nutritional composition and lactation performance of 304 dairy cattle in our study to obtain the M/D for the experimental dairy herd. The individual values of methane emissions ranged from 12.65 to 30.72 g/kg of DMI, with an average of 20.21 g/kg of DMI (*SI Appendix*, Table S2).

**Fig. 1.**
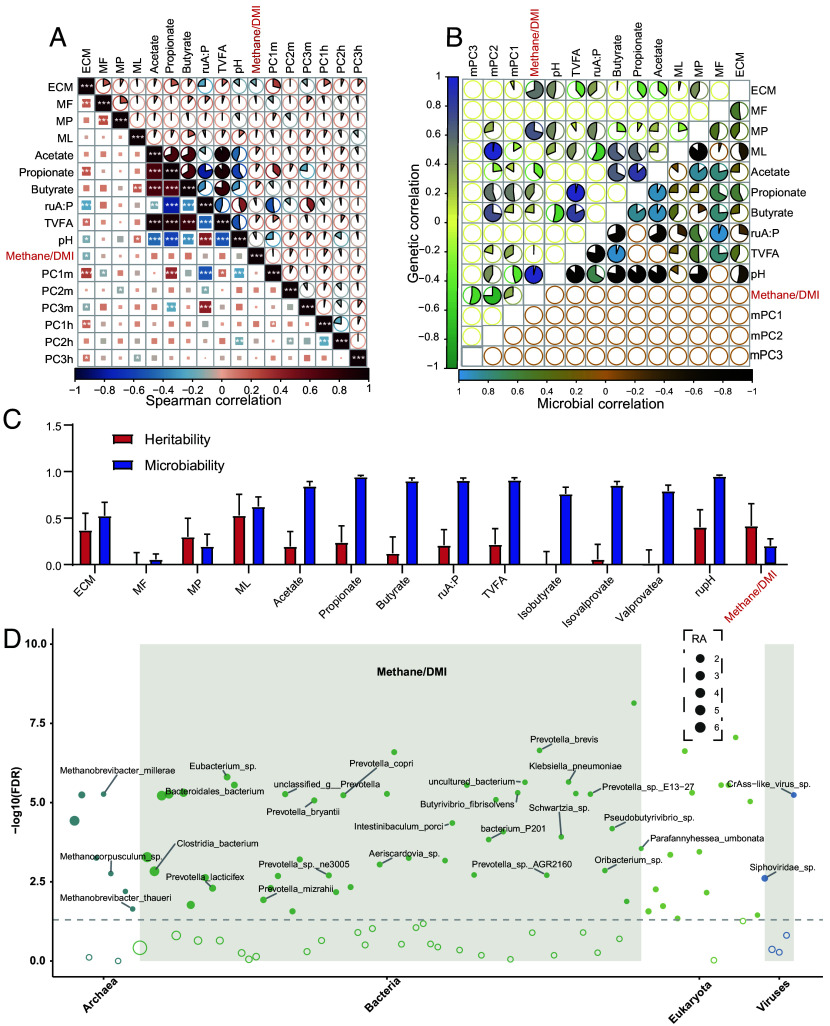
The interrelationships among methane emission, lactation performance, and rumen environment. (*A*) The Spearman correlation between methane and lactation performance (ECM, MF, MP, ML), and rumen environment (pH, Acetate, Propionate, Butyrate, and ruAP). (*B*) The genetic correlation (upper diagonal) and microbial correlation (lower diagonal) between methane and lactation performance (ECM, MF, MP, ML), rumen environment (pH, Acetate, Propionate, Butyrate, and ruAP). (*C*) The heritability and microbiability of methane, lactation performance (ECM, MF, MP, ML), rumen environment (pH, Acetate, Propionate, Butyrate, and ruAP). (*D*) Using metagenome-wide analysis (MWAS) with M/D as the phenotype to explore methane emission-related heritable microbes among the top 100 species-level microbes by relative abundance.

Among all phenotypes, the methane emission had a significant negative Spearman correlation with ECM (*P* < 0.05) (Fig. 1*A*). Next, we further focused on the genetic and microbial correlation between methane emission and other phenotypes. Compared with Spearman correlation, the methane emission showed genetic correlation with more phenotypes, including ECM, MP, propionate, and microbial PC2 and PC3 (mPC2 and mPC3) (|coefficients| >0.4) (above diagonal lines of Fig. 1*B*). However, there was no microbial correlation between methane emission and other phenotypes (below diagonal lines of Fig. 1*B*).

In order to quantify the effect of host genetic and rumen microbiome on phenotypes, we calculated the heritability and microbiability of all phenotypes (Fig. 1*C*). The microbiability of rumen fermentation parameters indicators in dairy cattle was greater than their heritability. However, it was worth noting that the heritability of methane emissions (*h^2^* = 0.42) is greater than its microbiability (*m^2^* = 0.19) (*SI Appendix*, Table S2). The above results indicated that although methane was a metabolic product of rumen microbes, its emissions were more influenced by host genetics, and the relationship between methane emissions and lactation performance, rumen fermentation parameters, and rumen microbes needs to be determined through host genetics.

### 2.2. The Heritability of Rumen Microbiota and Its Relationship With Methane Emissions.

The above results indicated that host genetics plays an important role in rumen methane emissions. Therefore, we further focused on the heritability of rumen microbiota. However, the archaea domain had lower heritability as direct producers of methane. The heritability of the bacterial domain and the proportion of heritable species are relatively high (*h^2^* ≥ 0.2) (*SI Appendix*, Fig. S2*A*). Moreover, we focused on the heritability of enzymes involved in hydrogenase in the HydDB database (32). [FeFe]_Group_A1d, which belongs to the Ferredoxin pathway; [NiFe]_Group_1d, which belongs to Fumarate respiration; and [NiFe]_Group_3a and [NiFe]_Group_4h, which belong to Methanogenesis, are all highly heritable hydrogenases. (*h^2^* ≥ 0.2) (*SI Appendix*, Fig. S2*B*).

In order to further screen for microbes related to methane emissions, we conducted MWAS on the top 100 rumen microbes with relative abundance at the species level, using methane emissions as the phenotype. The results showed that 66 microbes were closely related to methane emissions, of which 27 belonged to heritable microbes (Fig. 1*D*), which was identified the key mediators of host genetic influence on rumen methane emissions, mainly including: *Prevotella_mizrahii*, *Prevotella_bryantii*, *Prevotella_sp._*AGR2160, and *etc* (*FDR* < 0.05) (*SI Appendix*, Table S3).

### 2.3. Screening Key Heritable Rumen Microbes Regulating Methane Emissions Based On Mendelian Randomization.

**Fig. 2.**
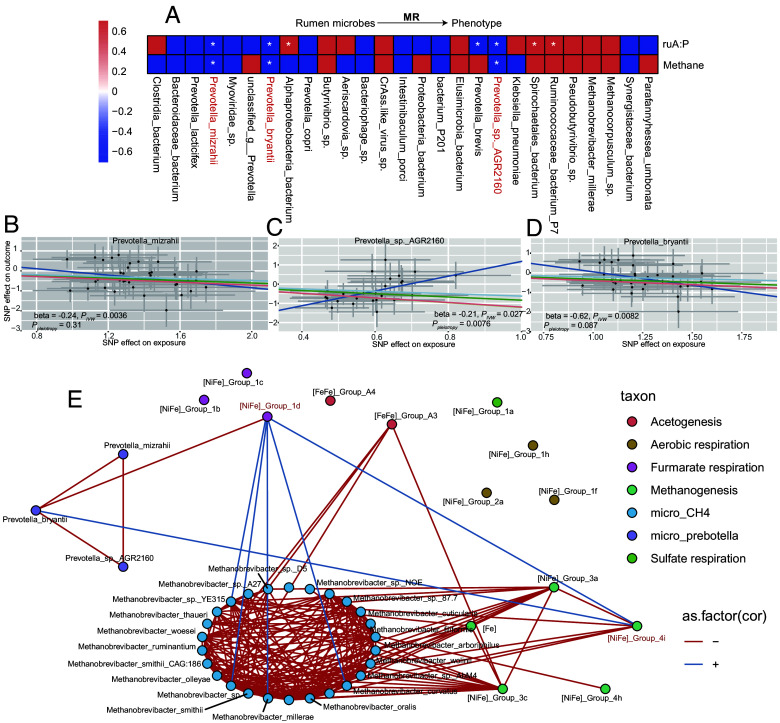
Exploring heritable rumen microbes regulating methane emissions through Mendelian randomization analysis. (*A*) The causal relationship between heritable rumen microbes (related to methane emissions and heritable through MWAS mining) as exposure factors and methane emissions, ruAP as outcomes. (*B*–*D*) The pleiotropy test of *Prevotella_mizrahii*, *Prevotella_sp._*AGR2160, and *Prevotella_bryantii.* (*E*) The network relationship of rumen hydrogenase, *Methanobrevibacter*, and *Prevotella* (*Prevotella_mizrahii*, *Prevotella_sp._*AGR2160, and *Prevotella_bryantii*).

In order to screen the key rumen microbes regulating methane emissions using the MR analysis. Here, we first used the 27 heritable microbes related to M/D identified by MWAS as exposure factors, based on independence and significance settings, an average of 15 instrumental variable SNPs can be screened for each microbe (*SI Appendix*, Table S4). Subsequently, M/D was considered as the outcome, revealing a negative causal correlation with *Prevotella_bryantii*, *Prevotella_mizrahii*, and *Prevotella_sp._*AGR2160 (*FDR_IVW_* < 0.05) (Fig. 2*A*). Therein, *Prevotella_bryantii* and *Prevotella_mizrahii* satisfied the pleiotropy test (*P_pleiotropy_* > 0.05) (Fig. 2 *B*–*D*). Moreover, *Prevotella_bryantii* exhibited the strongest negative effect on M/D with a beta coefficient of −0.62 (*SI Appendix*, Table S5).

Due to *Prevotella_bryantii*, *Prevotella_mizrahii*, and *Prevotella_sp._*AGR2160 all belong to the Bacteria, the methane was directly produced by Archaea, especially *Methanobrevibacter*. To investigate how the three heritable *Prevotella* species identified through MR analysis reduced methane emissions, we incorporated these three *Prevotella* species, *Methanobrevibacter* strains, and hydrogenases into the ecological network (Fig. 2*E*). The results indicated that there was a strong positive relationship within the *Prevotella* flora and within the *Methanobrevibacter* flora, but there were few direct connections between the two floras. The relationship between these two floras and hydrogenases was primarily as follows: The *Prevotella* flora was positively correlated with the [NiFe]_Group_1d of Fumarate respiration and negatively correlated with the [NiFe]_Group_4i of Methanogenesis. In contrast, the *Methanobrevibacter* flora was positively correlated with the hydrogenases of Methanogenesis and negatively correlated with the [NiFe]_Group_1d of Fumarate respiration. That was to say, the way *Prevotella* reduce methane emissions may be through [NiFe]_Group_1d competing with the methanogenic hydrogenase [NiFe]_Group_4i for hydrogen. Given that the association between the *Prevotella* flora and hydrogenase was mediated by *Prevotella_bryantii*, this study further investigated whether the *Prevotella_bryantii* genome harbored the functional [NiFe]_Group_1d gene. We downloaded all available complete genome sequences of *Prevotella_bryantii* strains from NCBI. By aligning these sequences with 10 [NiFe]_Group_1d amino acid sequences (*SI Appendix*, Table S6), we found that the *Prevotella_bryantii*_B14 genome contained a match for [NiFe]_Group_1d (*SI Appendix*, Fig. S3).

### 2.4. Structural Variation Characteristics of Rumen Microbiota and Key Structural Variants Influencing Methane Emissions in Prevotella_bryantii.

**Fig. 3.**
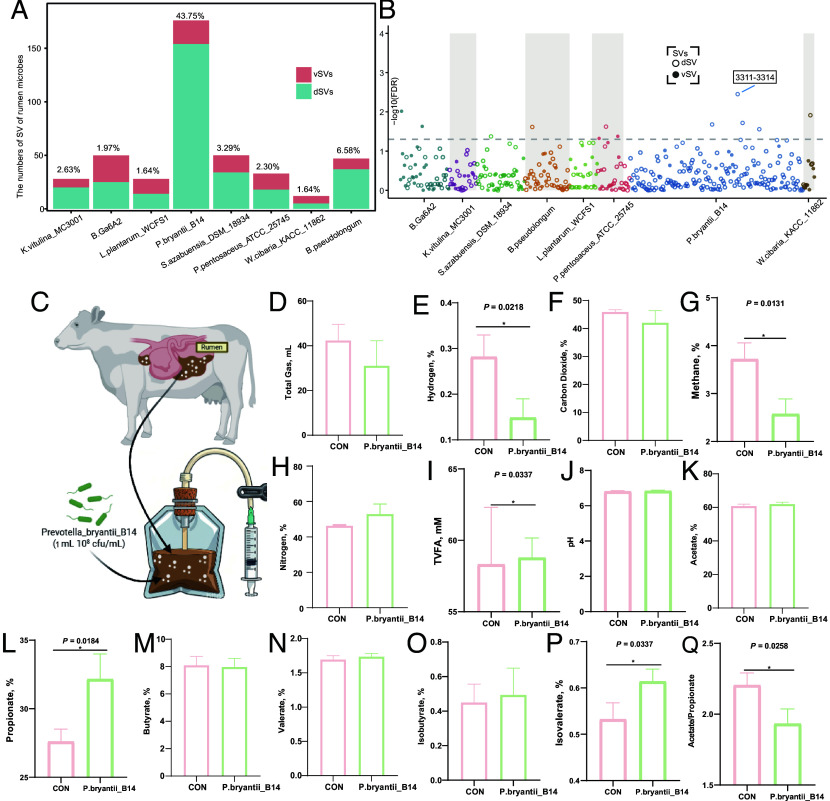
The relationship between the structural variation of *Prevotella_bryantii* and methane emission. (*A*) Structural variation characteristics of rumen microbes. (*B*) Microbial structural variations related to methane emissions by MWAS. (*C*) In vitro fermentation validation of methane emission reduction effect of *Prevotella_bryantii*_B14. (*D*–*Q*) Changes in rumen fermentation parameters and gas parameters after adding *Prevotella_bryantii*_B14 to an in vitro fermentation system.

In a total of 304 rumen metagenomics samples, we detected SV of 8 microbes by SGVfinder (*SI Appendix*, Table S7), in which the 8 vSVs and 20 dSVs of *Kandleria_vitulina_MC3001* were detected in 8 samples; the 25 vSVs and 25 dSVs of *Bacteroides_sp.Ga6A2* were detected in 6 samples; the 14 vSVs and 14 dSVs of *Lactobacillus_plantarum_WCFS1* were detected in 5 samples; the 22 vSVs and 154 dSVs of *Prevotella_bryantii*_B14 were detected in 133 samples; the 16 vSVs and 34 dSVs of *Sharpea_azabuensis_DSM* were detected in 10 samples; the 15 vSVs and 18 dSVs of *Pediococcus_pentosaceus_ATCC* were detected in 7 samples; the 7 vSVs and 5 dSVs of *Weissella_clibaria_KACC* were detected in 5 samples; the 20 vSVs and 37 dSVs of *Bifidobacterium_pseudolongum_subsp.globosum* were detected in 20 samples (Fig. 3*A*). Next, we used the MWAS to link the SVs with the M/D of dairy cattle, and results showed that the dSV of rumen *Prevotella_bryantii*_B14 was associated with M/D (Fig. 3*B*).

MR analysis revealed that *Prevotella_bryantii* had the largest coefficient for methane emission reduction, and it was found that SVs in its genome were closely associated with methane emissions. Therefore, we further added *Prevotella_bryantii*_B14 to an in vitro rumen fermentation system to validate its methane reduction effect (Fig. 3*C*). The results showed that compared to the control group, the addition of *Prevotella_bryantii*_B14 significantly increased TVFA, reduced the production of hydrogen and methane in the in vitro fermentation system, while also decreasing the ruAP (Fig. 3 *D*–*P*).

### 2.5. Screening for Rumen Metabolites Regulating 3 Prevotella and Subsequently Affecting Methane Emissions Based On Mendelian Randomization.

**Fig. 4.**
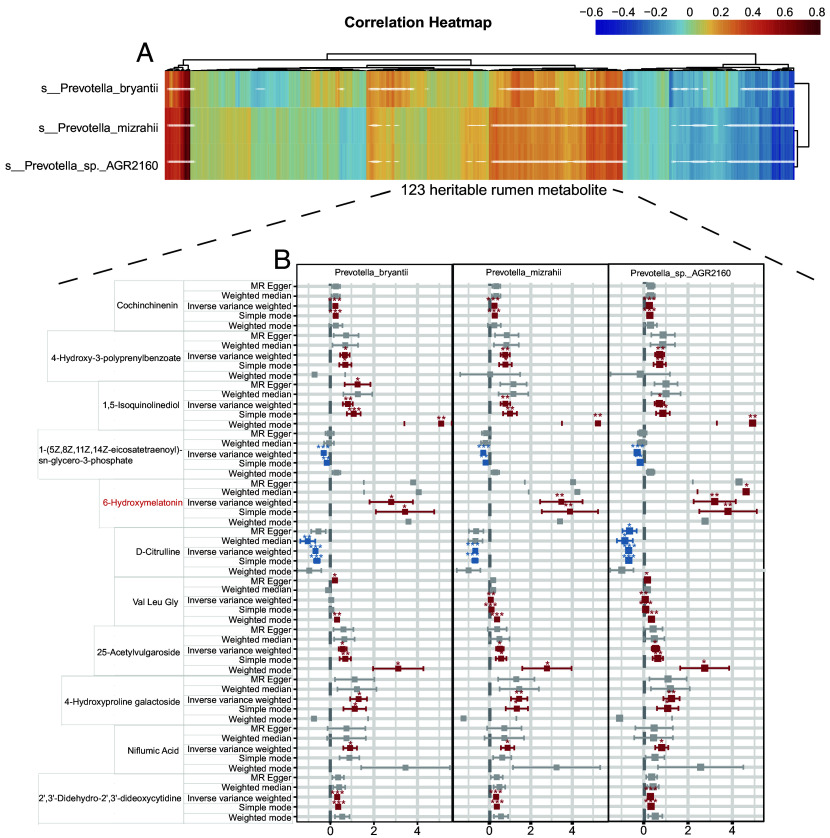
Exploring host-derived heritable rumen metabolites regulating *Prevotella* (*Prevotella_mizrahii*, *Prevotella_sp._*AGR2160, and *Prevotella_bryanti*) through Mendelian randomization analysis. (*A*) The Spearman relationship between *Prevotella* (*Prevotella_mizrahii*, *Prevotella_sp._*AGR2160, and *Prevotella_bryanti*) and 446 heritable rumen metabolites in the rumen metabolome. (*B*) 11 heritable rumen metabolites regulating *Prevotella* (*Prevotella_mizrahii*, *Prevotella_sp._*AGR2160, and *Prevotella_bryanti*) analyzed by Mendelian randomization analysis.

To elucidate how the host influences 3 *Prevotella* and subsequently affects methane emissions, we first focused on the relationship between heritable metabolites in the rumen metabolome and *Prevotella_bryantii*, *Prevotella_mizrahii*, *Prevotella_sp._*AGR2160 (*SI Appendix*, Table S8). The results indicated that 123 out of 446 heritable metabolites were associated with *Prevotella_bryantii*, *Prevotella_mizrahii*, *Prevotella_sp._*AGR2160 (*FDR* < 0.05) (Fig. 4*A*). Based on these 123 associated heritable metabolites, further application of MR analysis revealed that 11 of these metabolites could serve as exposure factors (*SI Appendix*, Table S9) regulating *Prevotella_bryantii*, *Prevotella_mizrahii*, *Prevotella*_sp._AGR2160 (*SI Appendix*, Table S10). Combined with metabolite source tracing analysis by MetOrigin (16) (*SI Appendix*, Fig. S4 and Table S11), we focused on host-derived metabolites and found that 6-hydroxymelatonin exerted the strongest positive regulatory effect on *Prevotella_bryantii*, *Prevotella_mizrahii*, and *Prevotella_sp._*AGR2160 (*FDR_IVW_* < 0.05). (Fig. 4*B*).

### 2.6. In Vitro Verification of 6-Hydroxymelatonin Reducing Methane Emissions.

**Fig. 5.**
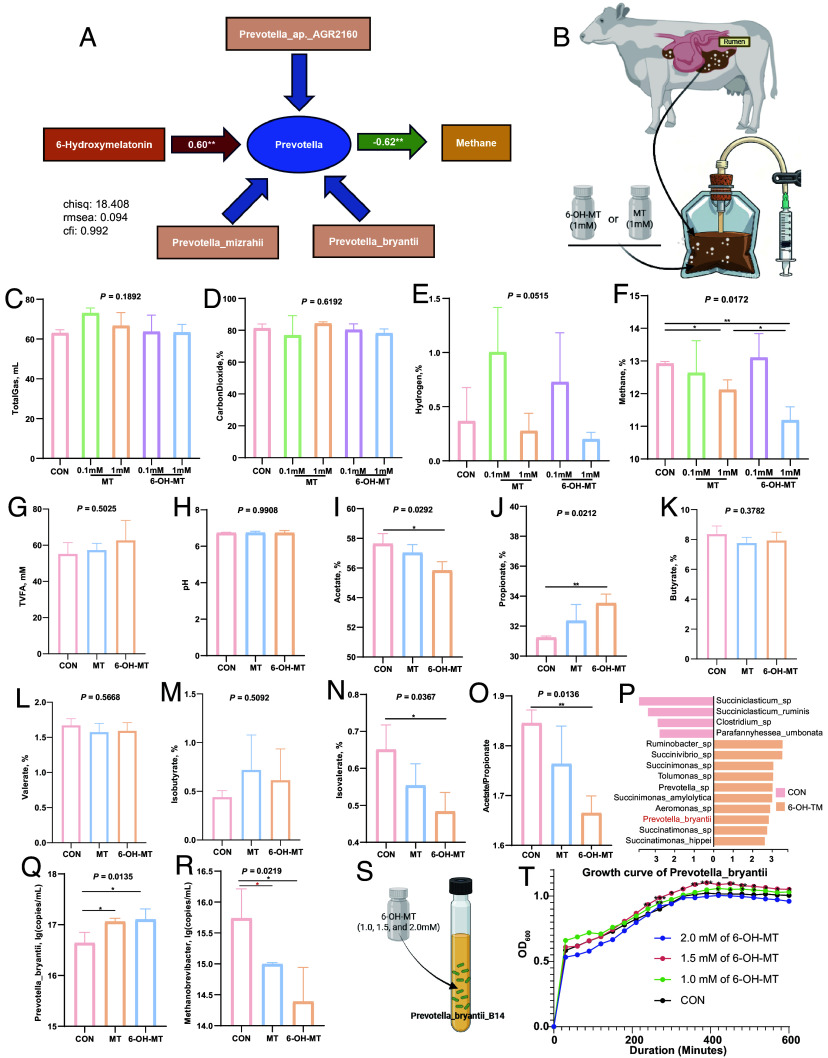
The effect of 6-hydroxymelatonin on methane emission reduction. (*A*) Structural equation modeling shows that 6-hydroxymelatonin *Prevotella* (*Prevotella_mizrahii*, *Prevotella_p._*AGR2160, and *Prevotella_bryanti*) regulated methane emissions. (*B*) In vitro fermentation validation of methane emission reduction effect of 6-hydroxymelatonin. (*C*–*O*) Changes in rumen fermentation parameters and gas parameters after adding 6-hydroxymelatonin to an in vitro fermentation system. (*P*) Lefse analysis of biomarker microbes in the control and 1 mM 6-hydroxymelatonin group of in vitro fermentation system. (*Q* and *R*) The differences in lg(copies/mL) of *Prevotella_bryantii* and *Methanobrevibacter* among the in vitro fermentation control group, 1 mM melatonin group, and 1 mM 6-hydroxymelatonin group. (*S* and *T*) The growth curve changes of *Prevotella_bryantii*_B14 in pure culture medium after adding 6-hydroxymelatonin at concentrations of 1.0, 1.5, and 2.0 mM, respectively.

Through MR analysis, we found that 6-hydroxymelatonin can increase the abundance of ruminal *Prevotella*, but no direct link with methane emissions was established. Therefore, we employed a SEM incorporating 6-hydroxymelatonin, a latent variable composed of three *Prevotella* species (*Prevotella_bryantii*, *Prevotella_mizrahii*, *Prevotella_sp._*AGR2160), and methane emissions (Fig. 5*A*). The results revealed that 6-hydroxymelatonin could reduce methane emissions via its effect on *Prevotella*. Six-hydroxymelatonin is a major metabolite of melatonin produced in the host liver (33). Subsequently, to validate the effect of 6-hydroxymelatonin, we conducted in vitro fermentation experiments with supplementation of either 6-hydroxymelatonin or melatonin (Fig. 5*B*). The results showed that melatonin and 6-hydroxymelatonin at a concentration of 0.1 mM had no effect on gas production during in vitro fermentation (*P* > 0.05); however, both 1 mM melatonin and 6-hydroxymelatonin significantly reduced methane emissions, with 6-hydroxymelatonin exhibiting a more pronounced inhibitory effect (*P* < 0.05). Additionally, neither melatonin nor 6-hydroxymelatonin affected total gas production (*P* > 0.05) (Fig. 5 *C*–*F*). Based on these findings, we focused on the effects of 1 mM melatonin and 6-hydroxymelatonin. It was observed that only 6-hydroxymelatonin, not melatonin, significantly promoted ruminal propionate-type fermentation and significantly decreased the concentration of isovalerate in the rumen (*P* < 0.05) (Fig. 5 *G*–*O*). Through metagenomic sequencing, compared with the control group, *Prevotella_bryantii* was identified as the marker microbes in the 1 mM 6-hydroxymelatonin group by Lefse analysis (Fig. 5*P*). Furthermore, using the 16S rDNA absolute quantification method, it was found that the copy numbers of *Prevotella_bryantii* in the 1 mM 6-hydroxymelatonin group and the 1 mM melatonin group were significantly higher than those in the control group (*P* < 0.05), with the 6-hydroxymelatonin group exhibiting the highest numerical value (Fig. 5*Q*). Conversely, the copy numbers of *Methanobrevibacter* in the 1 mM 6-hydroxymelatonin group and the 1 mM melatonin group were significantly lower than those in the control group (*P* < 0.05), with the 6-hydroxymelatonin group showing the lowest numerical value (Fig. 5*R*). However, the above results were obtained in the rumen microecosystem, which cannot prove that 6-hydroxymelatonin has a direct effect on *Prevotella_bryantii*. Therefore, 6-hydroxymelatonin at concentrations of 1, 1.5, and 2.0 mM was further added to the pure culture medium of *Prevotella_bryantii*_B14 (Fig. 5S). The results showed that compared to *Prevotella_bryantii*_B14 without 6-hydroxymelatonin, the OD_600_ at 270 and 420-510 min in the 1 mM group was significantly higher, which is consistent with the result that the 1 mM group increased the absolute copy number of *Prevotella_bryantii*_B14 in the in vitro rumen fermentation broth. Additionally, the OD_600_ of the 1.5 mM group was consistently significantly higher than that of the control group after 240 min, indicating the most pronounced effect in promoting the growth of *Prevotella_bryantii*_B14. However, at a concentration of 2.0 mM, the OD_600_ was numerically lower than or equal to that of the control group (Fig. 5T). Therefore, 1.5 mM 6-hydroxymelatonin can directly act on *Prevotella_bryantii*_B14 and promote its growth.

### 2.7. To Identify the Genetic Variants and Elucidate the Molecular Mechanisms of Host‘s Synthesis of 6-hydroxymelatonin Based on GWAS.

**Fig. 6.**
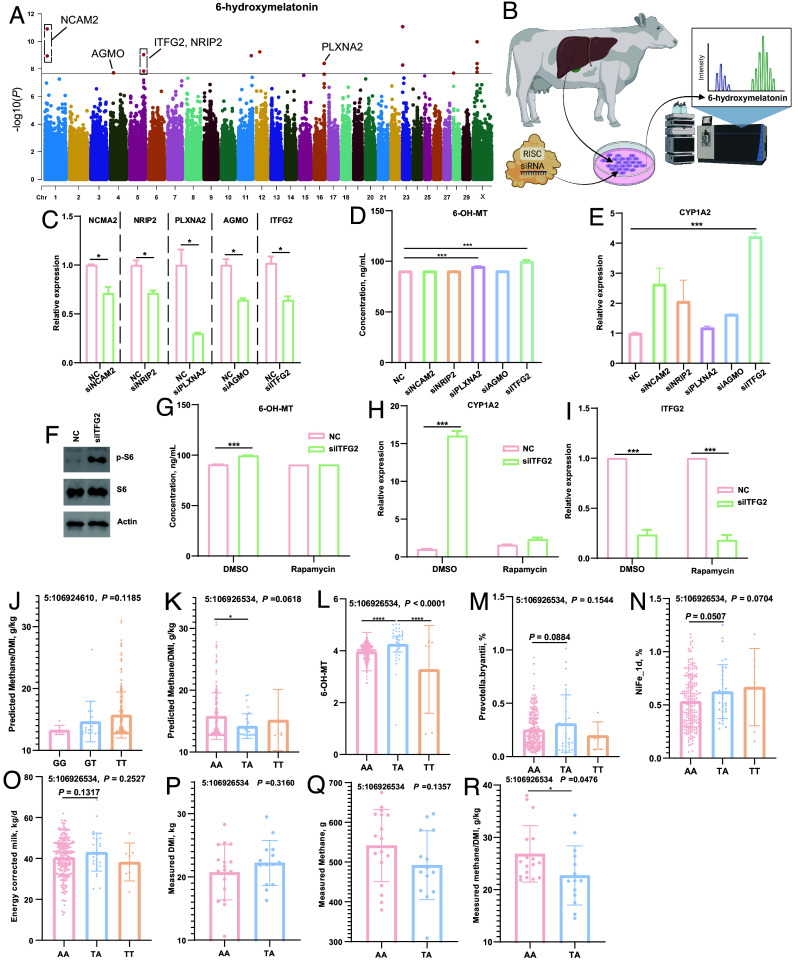
Regulation of Host Genetics on 6-Hydroxymelatonin. (*A*) GWAS for rumen 6-hydroxymelatonin. (*B*) Exploring key genetic genes regulating 6-hydroxymelatonin production based on bovine liver cells. (*C*) siRNA transfection efficiency of *NCAM2, PLXNA2, AGMO, NRIP2,* and *ITFG2.* (*D* and *E*) The change of 6-hydroxymelatonin concentration and *CYP*1*A*2 mRNA expression level after siRNA transfection. (*F*) The change of p-S6 and S6 protein level after siITFG2. (*G*–*I*) The effect of siITFG2 on the concentration of 6-hydroxymelatonin and *CYP*1*A*2 mRNA expression in bovine liver cells treated with Rapamycin. (*J*–*L*) The difference of predicted methane emissions, 6-hydroxymelatonin, *Prevotella_bryantii*, [NiFe]_Group_1d, and ECM among genotypes at 5:106926534. (*M*–*O*) The difference of measured methane emissions among genotypes 5:106926534 of 31 lactating cattle.

After confirming the effect of 6-hydroxymelatonin in reducing methane emissions, host genetic variants regulating 6-hydroxymelatonin were further explored through GWAS (Fig. 6*A*). A total of 15 SNP variants associated with ruminal 6-hydroxymelatonin were identified (-log10(P) > 7.67), which were annotated to the candidate genes *NCAM*2, *PLXNA*2, *AGMO*, *NRIP*2, and *ITFG*2 (*SI Appendix*, Table S12). Six-hydroxymelatonin is a metabolite formed by the hydroxylation of melatonin in the host liver by the *CYP*1*A*2 gene (34). Therefore, siRNA knockdown of these five candidate genes was performed in hepatocytes to identify the major gene influencing 6-hydroxymelatonin levels (Fig. 6*B*). The designed siRNAs significantly knocked down all five candidate genes with comparable efficiency (*P* < 0.05) (Fig. 6*C*). Among the five candidate genes, knockdown of *PLXNA*2 and *ITFG*2 resulted in a significant increase in 6-hydroxymelatonin concentration (*P* < 0.05) (Fig. 6*D*), however, only knockdown of resulted in a significant increase *CYP*1*A*2 gene expression (*P* < 0.05) (Fig. 6*E*). Thus, focus was placed on *ITFG*2, an immunomodulatory intracellular protein that modulates the fate of B cells and negatively regulates mTORC1 signaling (35). This study also found that knocking down *ITFG*2 significantly activated S6K1, a downstream marker of the mTORC1 pathway (Fig. 6*F*). Subsequent treatment with Rapamycin revealed that inhibition of the mTORC1 pathway prevented the increase in 6-hydroxymelatonin concentration and *CYP*1*A*2 gene expression induced by *ITFG*2 knockdown (*P* > 0.05) (Fig. 6 *G* and *H*). Additionally, Rapamycin did not affect the knockdown efficiency of *ITFG*2 itself (*P* < 0.05) (Fig. 6*I*). These results indicated that *ITFG*2 influences hepatic 6-hydroxymelatonin synthesis through the mTORC1 pathway. The key genetic variants affecting 6-hydroxymelatonin synthesis identified by GWAS—5:106924610 and 5:106926534—were annotated downstream of the *ITFG*2 gene (*SI Appendix*, Table S12). Further investigation confirmed the relationship between polymorphisms of variants and the efficiency of 6-hydroxymelatonin synthesis. Results showed that there were no significant differences among genotypes of 5:106924610 (Fig. 6*J*), only cattle with the TA genotype of 5:106926534 exhibited significantly reduced predicted M/D (*P* < 0.05) (Fig. 6*K*). Furthermore, the TA genotype of 5:106926534 have a significantly higher rumen 6-hydroxymelatonin (*P* < 0.05) (Fig. 6*L*), and an increasing trend observed for *Prevotella_bryantii* and [NiFe]_Group_1d (0.05 < *P* < 0.10) (Fig. 6 *M* and *N*). Additionally, different genotypes do not affect lactation performance, but the TA genotype shows higher values numerically (AA: 40.50 kg/d, TA: 43.12 kg/d, TT: 38.31 kg/d) (Fig. 6*O*). To further validate whether differences in methane emissions exist among cattle with different genotypes at the 5:106926534 locus, 31 lactating cattle were selected from a dairy farm different from the 304 experimental cattle for methane emission measurement using the AHC system. Among the 31 cattle, 17 were identified as having the AA genotype and 14 as having the TA genotype at the 5:106926534 locus. No significant differences were observed between the two genotypes in terms of methane emissions and DMI (*P* > 0.05) (Fig. 6 *P* and *Q*). However, consistent with the predicted M/D results from the 304 cattle, the measured M/D of the TA genotype was significantly lower than that of the AA genotype (*P* < 0.05) (Fig. 6*R*).

## 3. Discussion

Methane emissions, as metabolic byproducts of rumen microbes, represent a loss of energy and are also a significant contributor to the greenhouse effect. Extensive research has focused on regulating methane emissions, with a primary emphasis on nutritional strategies (36). However, whether through dietary adjustments or additives, most strategies to mitigate methane emissions face significant trade offs. These often include compromised production performance, impaired animal health, increased operational costs, or a lack of sustained efficacy over time (3, 4). Given these factors, a growing body of research has begun to focus on the breeding of dairy cattle with low methane emissions. Existing studies have shown that methane emission is a moderately heritable trait (37, 38). In this study, based on the artificial neural network model of methane emissions constructed from previous research (3), we found that predicted M/D is also a medium heritable trait (0.41). Meanwhile, among the various phenotypes (rumen environment and lactation performance), methane emissions showed a significant negative correlation only with ECM. While, methane emissions have shown genetic correlations with more phenotypes. Interestingly, previous studies have found that the absolute amount of methane emissions is positively genetically correlated with milk yield (38, 39), which limits genetic selection for cattle with lower methane emissions due to the need to balance potential losses in lactation performance. In this study, the predicted methane emission value represents methane emitted per kilogram of DMI, and its genetic correlation with ECM is negative. Therefore, using M/D as a phenotype for genetic selection targeting methane emissions may be more advantageous. Additionally, a negative genetic correlation between M/D has been reported in other research (40). Interestingly, no microbial correlation was found between methane emissions and other phenotypic indicators. This highlights that the association between methane emissions and other traits relies on host genetics.

However, when studying the heritability of ruminal methane emissions, the rumen microbiome is an unavoidable topic. Numerous studies have focused on the heritable subsets of rumen microbes and their relationship with methane emission. Here, we first estimated the microbiability of methane emissions and found that it was lower than the corresponding heritability. This observation has also been reported in previous studies (41). Combined with the strong genetic correlation between methane emissions and the rumen microbiome (PC of microbial PCoA), these results suggest that the regulation of rumen microbial methane emissions is dependent on host genetics. However, there is no universally accepted and unified classification of heritable rumen microbes that influence methane emissions. Some studies have found that methanogenic archaea belong to heritable taxa (41, 42), thereby directly affecting methane emissions. Other studies have indicated that methanogenic archaea have low heritability, whereas Bacteroidetes exhibit higher heritability, thus indirectly inhibiting methane emissions (43). Here, using MR analysis, we identified microbes with a causal relationship to methane emissions: *Prevotella_bryantii*, *Prevotella_mizrahii*, and *Prevotella_sp._*AGR2160, all of which belong to the Bacteroidetes phylum. In contrast, archaea showed lower heritability. Interestingly, studies with similar findings to our study, which identified heritable microbes that indirectly inhibit methane emissions, employed metagenomic sequencing (44), while studies that identified methanogenic archaea as heritable microbes used 16S amplicon sequencing (42). Therefore, the sequencing method may be the primary reason for the inconsistent results, and metagenomic sequencing is more recommended here, as it provides a more refined classification of taxa.

In addition, microbial respiration and fermentation both rely on the distribution of hydrogen, where the hydrogenase system plays a crucial role (45, 46). Different hydrogenases directly influence the direction of microbial fermentation. For instance, [NiFe]_Group_3a, [NiFe]_Group_3C, [NiFe]_Group_4h, [NiFe]_Group_4i, and pure iron hydrogenases are involved in methane production pathways. Meanwhile, [NiFe]_Group_1b, [NiFe]_Group_1c, and [NiFe]_Group_1d participate in fumarate metabolism pathway (47), which are closely related to ruminal propionate-type fermentation (48, 49). This study reveals that *Prevotella_bryantii*_B14 possesses [NiFe]_Group_1d capability and competes with methanogenic [NiFe]_Group_4i for hydrogen ions, thereby reducing methane emissions.

After the study adding *Prevotella_bryantii*, which exhibited the largest effect value of MR analysis, into the in vitro fermentation system, it was indeed observed that methane emissions decreased while ruAP decreased. An in vitro culture study on *Prevotella_bryantii* found that it can oxidate reduction of hydrogen to fumarate, thereby avoiding the use of hydrogen as an electron donor for methanogens and ultimately reducing overall methane emissions (50). Further mining of heritable rumen metabolites regulating *Prevotella_bryantii* through MR analysis revealed that 6-hydroxymelatonin could enhance the abundance of *Prevotella*. Subsequent SEM demonstrated that 6-hydroxymelatonin may reduce methane emissions by increasing the abundance of these three *Prevotella* species. Interestingly, previous studies have found that melatonin can reduce methane emissions in dairy cattle (51). In this study, in vitro fermentation experiments were conducted to compare the effects of melatonin and its metabolite 6-hydroxymelatonin on methane emissions. The results showed that although both effectively inhibited methane production, 6-hydroxymelatonin had a more significant effect. This may be attributed to the additional hydroxyl group in 6-hydroxymelatonin, which enhances its water solubility (52), allowing it to distribute more readily in the rumen fluid environment and interact with microbes. Furthermore, in the pure culture medium of *Prevotella_bryantii*_B14, the addition of 1.5 mM 6-hydroxymelatonin accelerated its growth rate. This indicates that 6-hydroxymelatonin can directly act on *Prevotella_bryantii* in the complex rumen microecological environment, enhancing its ecological niche advantage by promoting growth rate, thereby achieving the goal of methane emission reduction.

Six-hydroxymelatonin is a primary metabolite of melatonin catalyzed by the cytochrome P450 enzyme system (particularly *CYP1A2*) in the host liver. Through combined analysis of GWAS and cellular molecular experiments, *ITFG2* was ultimately identified as part of the KICSTOR complex, which inhibits the mTORC1 pathway (35, 53, 54). This pathway plays a critical role in cells, particularly in signal transduction and metabolic regulation (55). Here, we found that the expression of *ITFG*2 suppresses the mTOR pathway, thereby inhibiting *CYP*1*A*2 expression and ultimately reducing the production of 6-hydroxymelatonin in the liver. Although our study demonstrates that hepatic *ITFG*2 regulates 6-hydroxymelatonin synthesis, we acknowledge that other organs may also contribute to its production. Future studies are needed to determine the relative contribution of extrahepatic sources to ruminal 6-hydroxymelatonin levels. Moreover, other candidate genes discovered through GWAS include *NCAM*2 (56), *PLXNA*2 (57), and *AGMO* (58). They are associated with host brain neural activity, which may indirectly influence melatonin secretion from the pineal gland, thereby affecting the concentration of 6-hydroxymelatonin in the rumen. For example, *NCAM*2/*PLXNA*2 are involved in neural synapse formation and may influence melatonin secretion by modulating pineal gland activity (56, 57). Integrating these findings with previous results, we have linked host liver metabolism to rumen microbial activity, uncovering a metabolic process through which host genetics influence methane emissions. Specifically, dairy cattle with the TA genotype at locus 5:106926534 in the *ITFG*2 gene exhibited lower predicted and measured M/D, identifying it as a potential genetic marker for low-carbon breeding in dairy cattle. However, compared to other genotypes, the ruminal levels of *Prevotella_bryantii*_B14 and [NiFe]_Group_1d in cows of this genotype did not show significantly higher levels (they were numerically higher). The reason may be that the rumen microbial environment is complex and involves a certain degree of stochasticity. The influence of the key genotype identified in this study on the rumen microbiota may be obscured by this inherent complexity. However, the resulting change in methane emissions is robust (validated across cohorts).

There were some limitations: 1) This study was primarily conducted in Holstein dairy cattle. The generalizability of the host–microbiota interaction pathway identified here to other ruminant species (such as beef cattle, sheep, and goats) requires further validation. 2) While we linked the key genetic variant (5:106926534) to the region of the *ITFG*2 gene and confirmed, via cellular models, *ITFG*2’s regulatory role in 6-hydroxymelatonin synthesis, the precise mechanism by which this variant influences *ITFG*2 transcription or expression (e.g., through enhancer/suppressor activity or chromatin conformation changes) warrants more in-depth molecular investigation. 3) In both in vitro fermentation systems and single-strain pure culture systems, this study found that 6-hydroxymelatonin could promote the growth of *Prevotella_bryantii_B14*. However, the mechanisms by which it promotes microbial growth still require further investigation, such as whether it acts as a growth-promoting factor or a nutritional substrate. In summary, this research provides a framework for investigating how host genetics can regulate gastrointestinal microbes and subsequently influence host phenotypes. Future studies can build upon this approach to uncover more host genetic regulatory patterns of the microbiome.

## 4. Conclusion

**Fig. 7.**
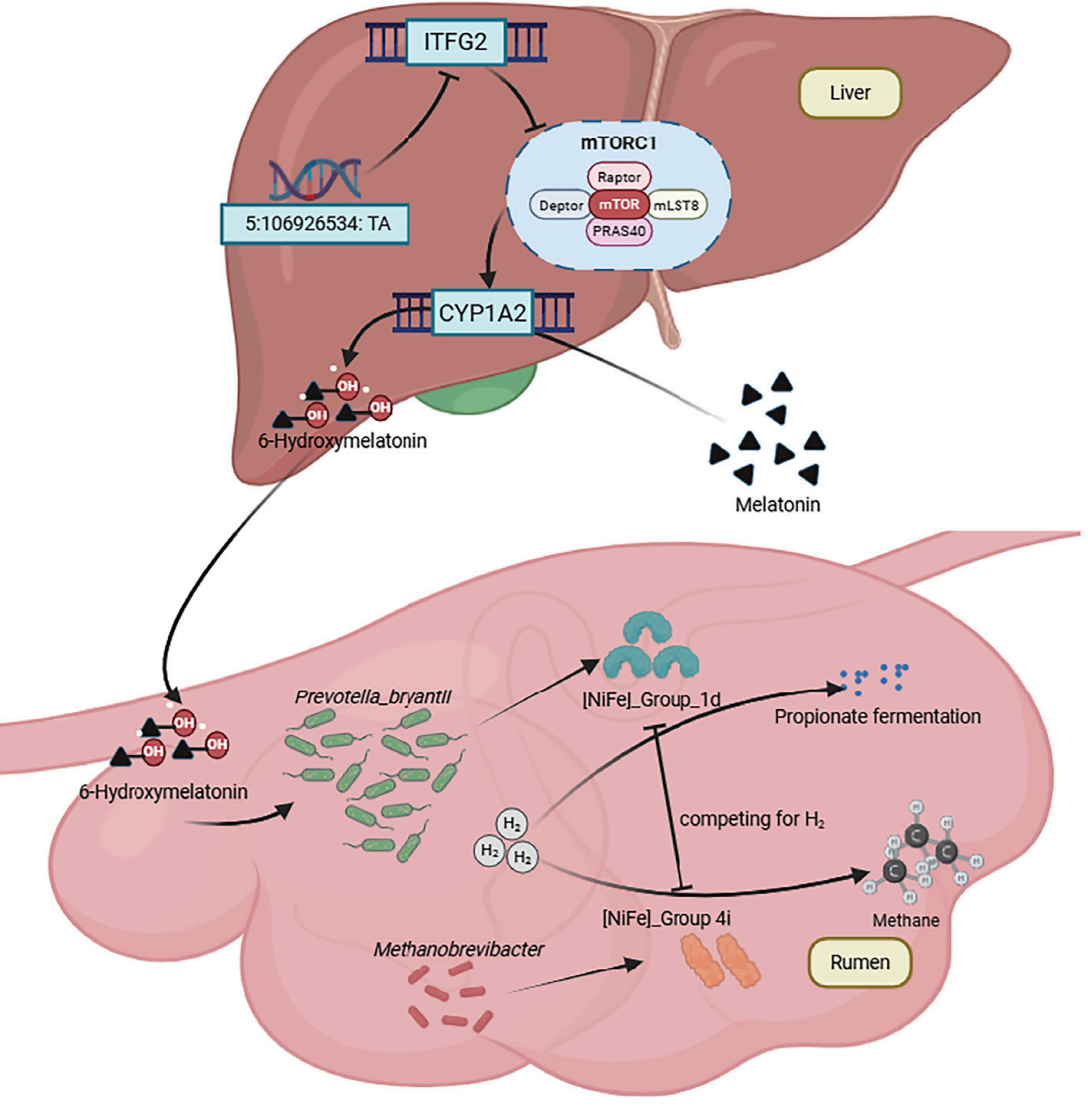
The mechanism by which host genetics affects methane emissions by regulating the production of 6-hydroxymelatonin.

In summary, this study is based on 304 dairy cattle and employs methods such as MWAS, GWAS, MR analysis, metagenomic SVs, and in vitro fermentation validation to explore the potential mechanisms of host genetic regulation of methane emissions in dairy cattle (Fig. 7): Cattle carrying the TA genotype (locus 5:106926534) exhibit lower expression of the liver gene *ITFG*2. This promotes mTOR pathway activity, which in turn increases the synthesis of the enzyme *CYP1A*2 and its product, 6-hydroxymelatonin. In the rumen, 6-hydroxymelatonin enriches the bacterium *Prevotella_bryantii* and its [NiFe]_Group_1d hydrogenase. This bacterial enzyme competes with the methanogen‘s [NiFe]_Group_4i hydrogenase for hydrogen, thereby suppressing methane production. This study provides methodological references for exploring the host genetic driven rumen microbial regulation mechanisms related to ruminant phenotypes.

## 5. EthicsApproval and Consent to Participate

This experiment was conducted at the Animal Research and Technology Centre of Northwest A&F University (Yangling, Shaanxi, China). All analyses were performed in accordance with the guidelines recommended by the Administration of Affairs Concerning Experimental Animals (Ministry of Science and Technology, China, revised 2004). The protocol was approved by the Institutional Animal Care and Use Committee of Northwest A&F University.

## Supplementary Material

Appendix 01 (PDF)

## Data Availability

The raw sequencing data used and described in this study have been deposited into CNGB Sequence Archive (CNSA) (https://db.cngb.org/cnsa/) of China National GeneBank DataBase (CNGBdb) with accession number CNP0005323 (Metagenome data) (59), CNP0005324 (whole-genome resequencing data) (60), and CNP0005479 (Metabolome data) (61).
