# Supplementary material for: Host genetic regulation of rumen 6-hydroxymelatonin reduces methane emissions in dairy cattle
Source: Proc Natl Acad Sci U S A. 2026 Jun 8;123(25):e2604454123. doi: 10.1073/pnas.2604454123 (PMC13291679; doi:10.1073/pnas.2604454123)
Supplement: Supplementary file 1 — Appendix 01 (PDF) [file pnas.2604454123.sapp.pdf]

Supplemental figures

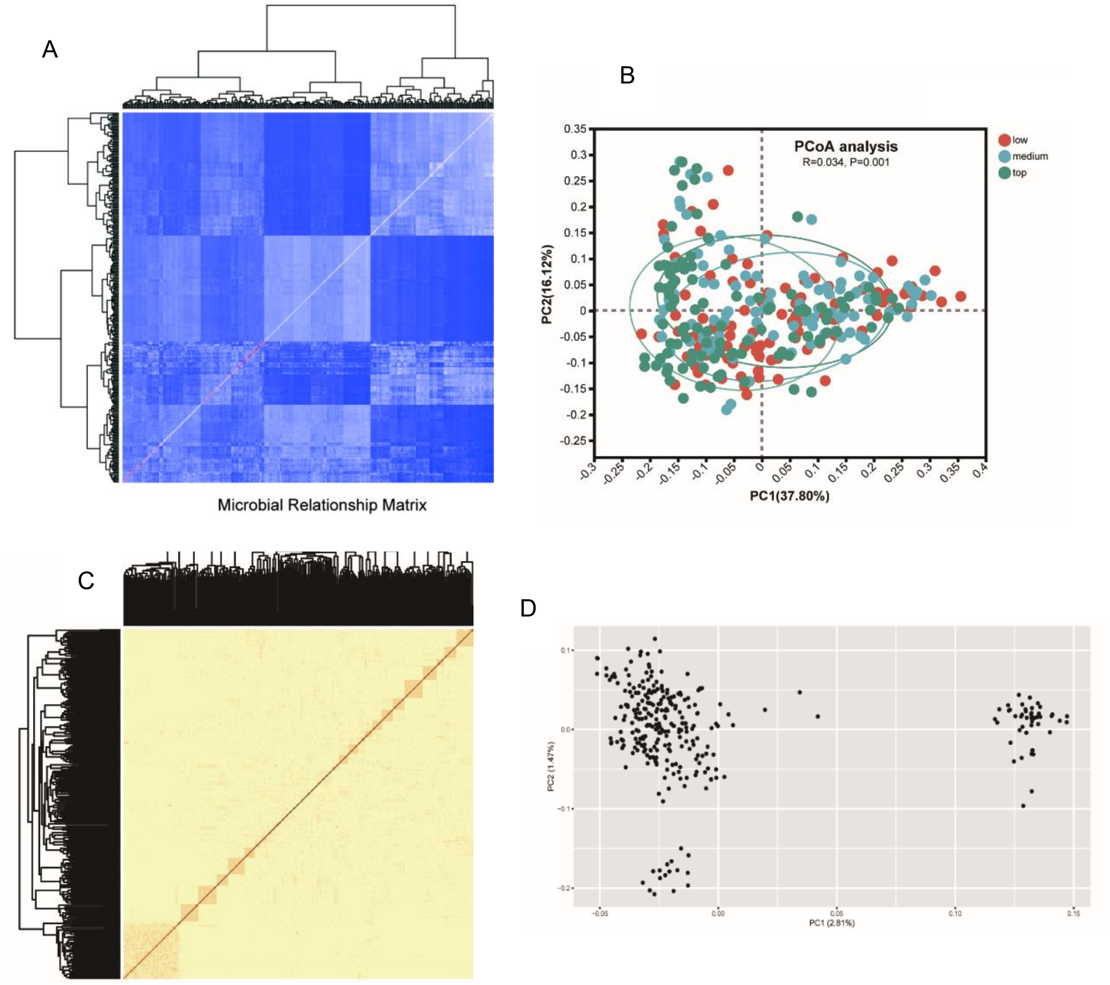

Figure S1. Rumen micobial and host genetic structure of dairy cattle

A. Microbial relationship matrix

B. Microbial population structure

C. Genetic relationship matrix

D. Genetic population structure

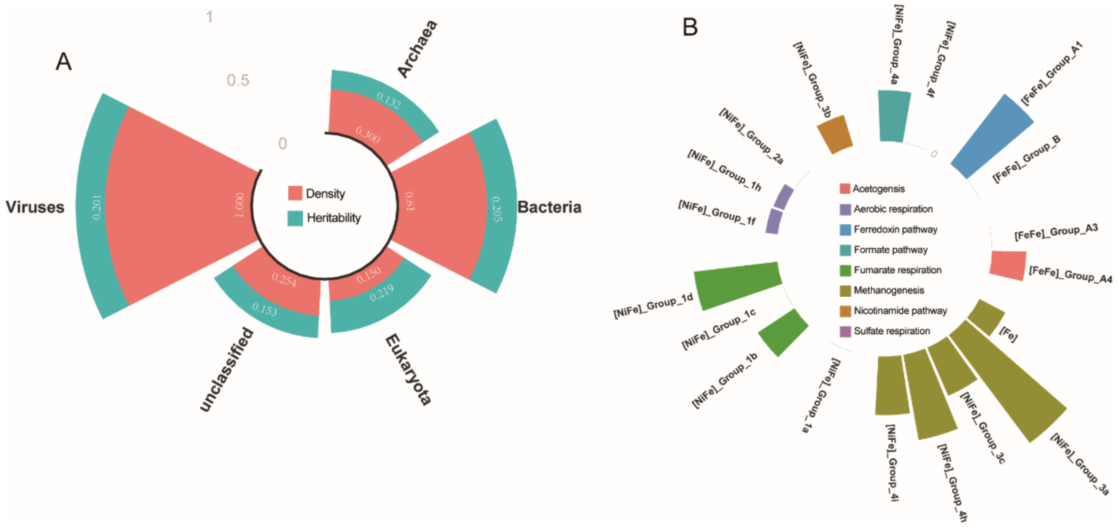

Figure S2. The heritability of rumen mcirobial taxon and hydrogenase

A. The heritability of rumen mcirobial taxon at domain level

B. The heritability of rumen mcirobial hydrogenase

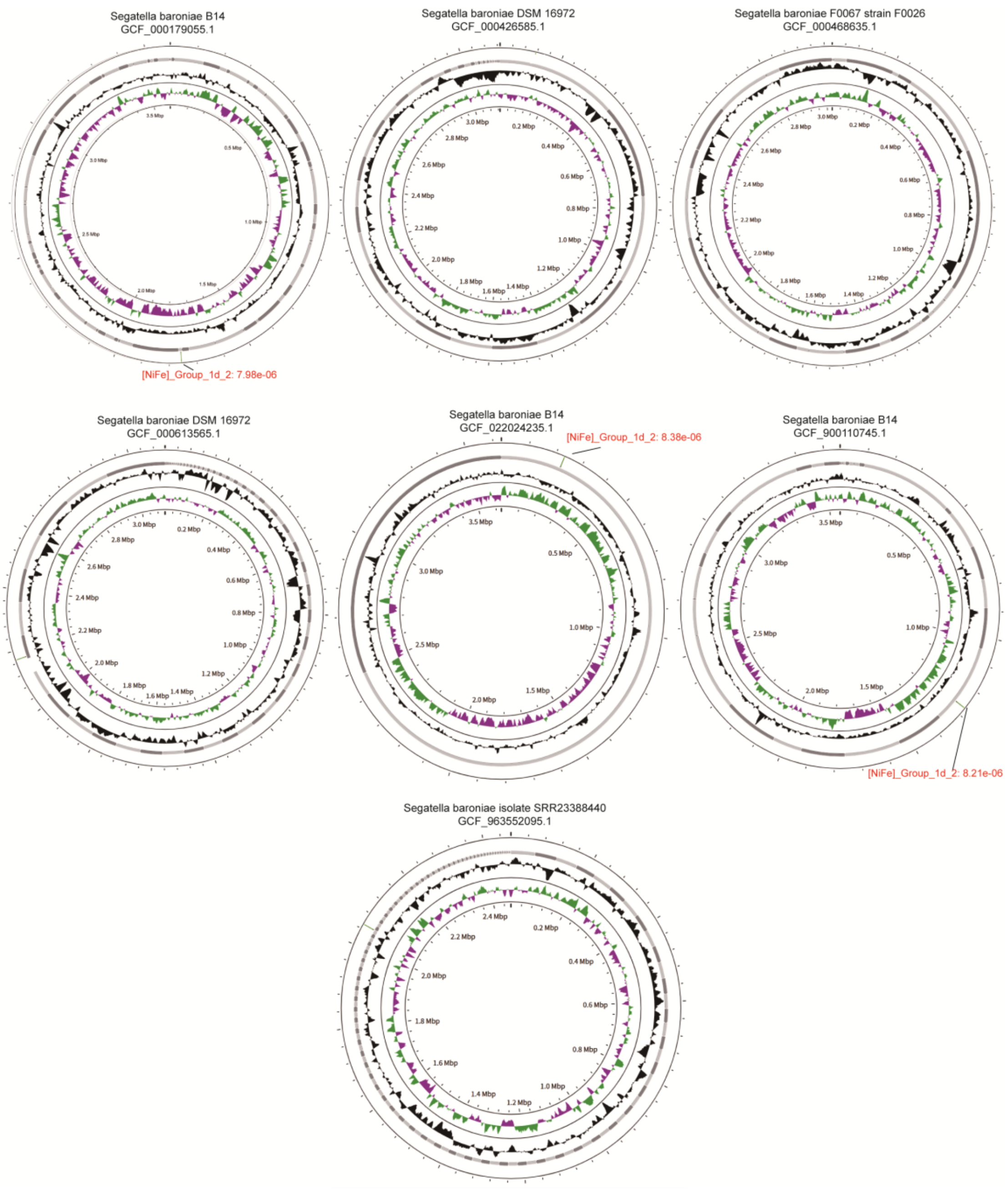

Figure S3. BLAST Analysis of NiFe_1d Against the Genome of *Prevotella_bryantii* B14

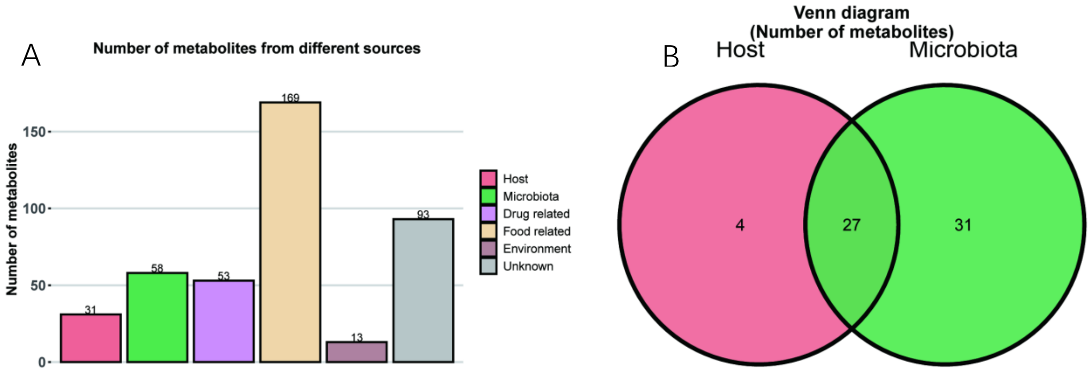

Figure S4. Origin Tracing of Rumen Metabolites

A. Number of metabolites from different sources

B. Venn diagram analysis of host-derived and microbiota-derived rumen metabolites

Supplemental Tables

Table S1.The sequences of primers and siRNAs

16S rDNA-specific primers	
Prevotella_bryantii	F' ACTGCAGCGCGAACTGTCAGA, R' ACCTTACGGTGGCAGTGTCTC
Methanobrevibacter	F' GAGGAAGGAGTGGACGACGGTA, R' ACGGGCGGTGTGTGCAAG
siRNAs sequences	
siNCAM2	5′- CTCTTCTTCAAGTGACAATTTCA-3′
siPLXNA2	5′- CTGAGTTCAAGTTCTACAACTGC-3′
siAGMO	5′- TCGCATGATGTTTTATGTGATGA-3′
siNRIP2	5′- GAGTAAGACAGTGGTGTAACTAA-3′
siITFG2	5′- ACGTTGACAACGACACGTTAAAT-3′
gene-specific primers	
ITFG2	F' GAAGTGCGATGAGGTCGGTT, R' CCCCAACGCAAGTGAGCATT
AGMO	F' GACCTTTGCAGATAAAGCTGTGCT, R' TACTCCCCAGAAAGCAATGCAG
PLXNA2	F' TGAATGCGTCCTCAACATCCA, R' CACACTTGTAGAGGTGGACTTTC
NCAM2	F' GAATTCAGCATGCCGCCTAAG, R' TTTTGATCCATCTTTCAGGTATGC
NRIP2	F' CCCAGCTTCATCTCGGCCT, R' CAGAGCTTGCAGTAGAGGGA
CYP1A2	F' TTCCGACACTCCTCCTTCGT, R' CCCCACAGCTTCGGGTCAT

Table S2. Data statistics of phenotype indicators for 304 dairy cows

Phenotype	Average	Median	Max	Min	1/4 percentile	3/4 percentile	h^2	m^2
ECM, kg/d	42.24	42.72	77.06	12.59	36.54	48.20	0.37	0.53
Milk fat, %	3.87	3.85	5.83	2.44	3.47	4.17	0.00	0.06
Milk protein, %	3.28	3.28	4.25	2.26	3.06	3.49	0.30	0.20
Acetate, %	53.59	53.63	74.46	17.82	47.41	60.11	0.20	0.84
Propionate, %	24.87	24.77	45.45	6.38	19.60	28.74	0.24	0.94
Butyrate, %	3.67	3.72	6.74	1.14	3.01	4.25	0.12	0.90
Isobutyrate, %	1.00	1.05	1.81	0.14	0.75	1.25	0.00	0.76
Isovalprovate, %	0.42	0.43	0.85	0.12	0.31	0.52	0.06	0.85
Valprovate, %	0.49	0.49	1.02	0.14	0.40	0.57	0.01	0.79
Acetate/Propionate	2.25	2.18	6.33	1.45	1.93	2.50	0.21	0.90
TVFA, mM	84.04	84.98	128.09	26.11	73.54	93.98	0.22	0.91
Rumen pH	6.44	6.48	7.47	5.19	6.19	6.71	0.40	0.95
Methane/DMI	20.21	19.30	30.72	12.65	15.51	25.51	0.42	0.19
Lactation time	99.75	94.00	302.00	2.00	62.00	130.00	-	-
Parity	2.38	2.00	6.00	1.00	2.00	3.00	-	-

Table S3. Metagenome-wide analysis between top 100 rumen microbes with relative abundances and methane emissions

Domin	Phylum	Genus	Species	RA	FDR	h^2
Bacteria	Bacteroidota	Prevotella	Prevotella_sp.	20.50	0.38	0.24
Bacteria	Firmicutes	unclassified_f__Lachnospiraceae	Lachnospiraceae_bacterium	7.45	0.00	0.13
Archaea	unclassified_Archaea	unclassified_Archaea	archaeon	6.64	0.00	0.20
Bacteria	Firmicutes	unclassified_c__Clostridia	Clostridia_bacterium	6.32	0.00	0.25
Bacteria	Bacteroidota	unclassified_o__Bacteroidales	Bacteroidales_bacterium	6.24	0.00	0.22
Bacteria	Firmicutes	unclassified_f__Oscillospiraceae	Oscillospiraceae_bacterium	4.42	0.00	0.00
Bacteria	Bacteroidota	unclassified_f__Bacteroidaceae	Bacteroidaceae_bacterium	3.61	0.16	0.20
Bacteria	Bacteroidota	unclassified_f__Muribaculaceae	Muribaculaceae_bacterium	3.04	0.00	0.19
Bacteria	Firmicutes	unclassified_c__Bacilli	Bacilli_bacterium	2.86	0.02	0.00
Bacteria	Firmicutes	Ruminococcus	Ruminococcus_sp.	1.80	0.23	0.02
Bacteria	Firmicutes	unclassified_o__Eubacteriales	Clostridiales_bacterium	1.30	0.00	0.00
Bacteria	Bacteroidota	Prevotella	Prevotella_lacticifex	1.18	0.01	0.35
Bacteria	Bacteroidota	Prevotella	Prevotella_ruminicola	1.11	0.23	0.31
Archaea	Euryarchaeota	Methanobrevibacter	Methanobrevibacter_sp.	1.09	0.00	0.15
Bacteria	Firmicutes	Eubacterium	Eubacterium_sp.	0.91	0.00	0.35
Bacteria	Firmicutes	unclassified_f__Acidaminococcaceae	Acidaminococcaceae_bacterium	0.89	0.00	0.16
Bacteria	Firmicutes	unclassified_p__Firmicutes	Firmicutes_bacterium	0.84	0.56	0.00
Viruses	Uroviricota	unclassified_f__Siphoviridae	Siphoviridae_sp.	0.81	0.00	0.40
Bacteria	unclassified_Bacteria	unclassified_Bacteria	bacterium	0.70	0.88	0.00
Bacteria	Bacteroidota	unclassified_f__Paludibacteraceae	Paludibacteraceae_bacterium	0.70	0.73	0.06
Bacteria	Bacteroidota	Prevotella	Prevotella_mizrahii	0.64	0.01	0.50
Viruses	Uroviricota	unclassified_f__Myoviridae	Myoviridae_sp.	0.52	0.43	0.47
Bacteria	Firmicutes	Mogibacterium	Mogibacterium_sp.	0.49	0.01	0.00
Bacteria	Spirochaetes	Treponema	Treponema_sp.	0.45	0.00	0.00
Eukaryota	Ciliophora	Stylonychia	Stylonychia_lemnae	0.43	0.03	0.09
Bacteria	Bacteroidota	Prevotella	unclassified_g__Prevotella	0.41	0.00	0.37
Bacteria	Proteobacteria	unclassified_f__Succinivibrionaceae	Succinivibrionaceae_bacterium	0.40	0.03	0.00
Bacteria	Firmicutes	unclassified_f__Erysipelotrichaceae	Erysipelotrichaceae_bacterium	0.37	0.00	0.15
Bacteria	Actinobacteria	unclassified_f__Atopobiaceae	Atopobiaceae_bacterium	0.36	0.50	0.09
Bacteria	Bacteroidota	Prevotella	Prevotella_bryantii	0.34	0.00	0.33
Bacteria	Proteobacteria	unclassified_c__Alphaproteobacteria	Alphaproteobacteria_bacterium	0.32	0.23	0.23
Bacteria	Bacteroidota	Prevotella	Prevotella_sp._ne3005	0.32	0.00	0.38
Bacteria	Bacteroidota	Prevotella	Prevotella_sp._tc2-28	0.31	0.01	0.07
Bacteria	Bacteroidota	Prevotella	Prevotella_copri	0.29	0.00	0.35
Bacteria	Tenericutes	unclassified_o__Acholeplasmatales	Acholeplasmatales_bacterium	0.28	0.00	0.11
Bacteria	Firmicutes	Butyrivibrio	Butyrivibrio_sp.	0.28	0.13	0.33
Eukaryota	Ciliophora	Blepharisma	Blepharisma_stoltei	0.28	0.01	0.11
Eukaryota	Ciliophora	Stentor	Stentor_coeruleus	0.28	0.02	0.15
Bacteria	Candidatus_Saccharibacteria	unclassified_p__Candidatus_Saccharibacteria	Candidatus_Saccharibacteria_bacterium	0.28	0.31	0.04
Bacteria	Firmicutes	Solobacterium	Solobacterium_sp.	0.26	0.09	0.00
Bacteria	Actinobacteria	Aeriscardovia	Aeriscardovia_sp.	0.25	0.00	0.40
Eukaryota	Ciliophora	Tetrahymena	Tetrahymena_thermophila	0.23	0.00	0.10

Bacteria	Firmicutes	Clostridium	Clostridium_sp.	0.23	0.00	0.16
Bacteria	Firmicutes	Blautia	Blautia_sp.	0.23	0.00	0.00
Viruses	Uroviricota	unclassified_f__Podoviridae	Podoviridae_sp.	0.19	0.53	0.34
Eukaryota	Ciliophora	Entodinium	Entodinium_caudatum	0.19	0.05	0.14
Bacteria	Firmicutes	Succiniclasticum	Succiniclasticum_ruminis	0.19	0.29	0.09
Viruses	unclassified_Viruses	unclassified_Viruses	Bacteriophage_sp.	0.19	0.15	0.30
Bacteria	Firmicutes	Sarcina	Sarcina_sp.	0.18	0.00	0.00
Bacteria	Bacteroidota	Prevotella	Prevotella_multisaccharivorax	0.18	0.09	0.49
Eukaryota	Chytridiomycota	Neocallimastix	Neocallimastix_sp._JGI-2020a	0.18	0.00	0.11
Eukaryota	Ciliophora	Ichthyophthirius	Ichthyophthirius_multifiliis	0.17	0.00	0.11
Bacteria	Firmicutes	Sarcina	Sarcina_sp._DSM_11001	0.17	0.07	0.00
Viruses	Uroviricota	unclassified_o__Crassvirales	CrAss-like_virus_sp.	0.17	0.00	0.31
Bacteria	Bacteroidota	Prevotella	Prevotella_sp._E2-28	0.16	0.29	0.22
Eukaryota	Ciliophora	Pseudocohnilembus	Pseudocohnilembus_persalinus	0.14	0.00	0.08
Bacteria	Bacteroidota	unclassified_f__Prevotellaceae	Prevotellaceae_bacterium	0.14	0.36	0.47
Eukaryota	Ciliophora	Halteria	Halteria_grandinella	0.13	0.01	0.16
Bacteria	Firmicutes	Parasporobacterium	Parasporobacterium_sp.	0.13	0.00	0.00
Bacteria	Firmicutes	Intestinibaculum	Intestinibaculum_porci	0.13	0.00	0.40
Bacteria	Bacteroidota	Prevotella	Prevotella_sp._tf2-5	0.12	0.45	0.13
Eukaryota	Ciliophora	Paramecium	Paramecium_sonneborni	0.12	0.94	0.11
Bacteria	Actinobacteria	unclassified_f__Eggerthellaceae	Eggerthellaceae_bacterium	0.12	0.00	0.00
Bacteria	Firmicutes	Selenomonas	Selenomonas_sp.	0.12	0.00	0.02
Bacteria	Proteobacteria	unclassified_p__Proteobacteria	Proteobacteria_bacterium	0.12	0.66	0.20
Archaea	Euryarchaeota	Methanosphaera	Methanosphaera_sp.	0.11	0.77	0.00
Bacteria	unclassified_Bacteria	unclassified_Bacteria	bacterium_P201	0.10	0.00	0.30
Bacteria	Firmicutes	Saccharofermentans	Saccharofermentans_sp.	0.10	0.00	0.08
Bacteria	Fibrobacteres	Fibrobacter	Fibrobacter_sp.	0.10	0.00	0.16
Bacteria	Elusimicrobia	unclassified_p__Elusimicrobia	Elusimicrobia_bacterium	0.09	0.88	0.31
Bacteria	Firmicutes	Butyrivibrio	Butyrivibrio_fibrisolvens	0.09	0.00	0.41
Bacteria	unclassified_Bacteria	unclassified_Bacteria	uncultured_bacterium	0.09	0.00	0.26
Bacteria	Bacteroidota	Prevotella	Prevotella_sp._E15-22	0.09	0.13	0.28
Bacteria	Bacteroidota	Prevotella	Prevotella_brevis	0.09	0.00	0.29
Eukaryota	Chytridiomycota	Neocallimastix	Neocallimastix_californiae	0.09	0.00	0.06
Bacteria	Bacteroidota	Prevotella	Prevotella_sp._AGR2160	0.09	0.00	0.44
Archaea	Euryarchaeota	Methanobrevibacter	Methanobrevibacter_ruminantium	0.08	0.00	0.15
Eukaryota	Chytridiomycota	Piromyces	Piromyces_sp._E2	0.08	0.00	0.14
Bacteria	Firmicutes	Ruminococcus	Ruminococcus_sp._M6(2020)	0.08	0.66	0.15
Bacteria	Firmicutes	Schwartzia	Schwartzia_sp.	0.08	0.00	0.36
Bacteria	Proteobacteria	Klebsiella	Klebsiella_pneumoniae	0.08	0.00	0.41
Bacteria	Kiritimatiellaeota	unclassified_c__Kiritimatiellae	Kiritimatiellae_bacterium	0.08	0.00	0.13
Bacteria	Spirochaetes	unclassified_o__Spirochaetales	Spirochaetales_bacterium	0.08	0.13	0.35
Bacteria	Bacteroidota	Prevotella	Prevotella_sp._E13-27	0.08	0.00	0.23
Bacteria	Firmicutes	unclassified_f__Oscillospiraceae	Ruminococcaceae_bacterium_P7	0.08	0.55	0.23
Eukaryota	Ciliophora	Paramecium	Paramecium_tetraurelia	0.07	0.00	0.09

Bacteria	Firmicutes	Oribacterium	Oribacterium_sp.	0.07	0.00	0.26
Bacteria	Firmicutes	Pseudobutyrivibrio	Pseudobutyrivibrio_sp.	0.07	0.00	0.47
Archaea	Euryarchaeota	Methanobrevibacter	Methanobrevibacter_millerae	0.07	0.00	0.29
Archaea	Euryarchaeota	Methanocorpusculum	Methanocorpusculum_sp.	0.07	0.00	0.60
Eukaryota	Ciliophora	Paramecium	Paramecium_primaurelia	0.07	0.05	0.09
Eukaryota	Chytridiomycota	Anaeromyces	Anaeromyces_robustus	0.07	0.00	0.16
Bacteria	Synergistetes	unclassified_f__Synergistaceae	Synergistaceae_bacterium	0.07	0.20	0.23
Archaea	Euryarchaeota	Methanosphaera	Methanosphaera_stadtmanae	0.06	1.00	0.00
Archaea	Euryarchaeota	Methanobrevibacter	Methanobrevibacter_olleyae	0.06	0.01	0.17
Bacteria	Firmicutes	Lachnoclostridium	Lachnoclostridium_sp._MSJ-17	0.06	0.01	0.15
Archaea	Euryarchaeota	Methanobrevibacter	Methanobrevibacter_thaueri	0.06	0.02	0.26
Bacteria	Firmicutes	unclassified_f__Selenomonadaceae	Selenomonadaceae_bacterium	0.06	0.00	0.00
Eukaryota	Ciliophora	Paramecium	Paramecium_octaurelia	0.06	0.04	0.08
Bacteria	Actinobacteria	Parafannyhessea	Parafannyhessea_umbonata	0	0.00	0.47

Table S4. Genetic associations with 28 rumen heritable microbes as expourse factor

Microbial feature	SNP	EA exposure	OA exposure	EA outcome	OA outcome	beta exposure	beta outcome	eaf exposure	pval outcome
Clostridia_bacterium	1:131823732	A	C	A	C	0.18	-0.06	0.09	0.94
Clostridia_bacterium	11:93061160	T	C	T	C	-0.26	1.74	0.06	0.02
Clostridia_bacterium	12:50126431	T	C	T	C	0.15	0.19	0.16	0.71
Clostridia_bacterium	19:48165383	A	G	A	G	0.20	0.83	0.09	0.18
Clostridia_bacterium	22:44694087	G	A	G	A	0.22	-0.33	0.08	0.65
Clostridia_bacterium	26:3016852	T	C	T	C	-0.28	0.63	0.05	0.42
Clostridia_bacterium	28:1013786	T	C	T	C	-0.15	-0.69	0.17	0.15
Clostridia_bacterium	28:1047550	T	G	T	G	-0.14	-0.53	0.14	0.40
Clostridia_bacterium	28:1047595	G	A	G	A	-0.14	-0.50	0.14	0.43
Clostridia_bacterium	28:1047646	A	G	A	G	-0.20	-0.22	0.13	0.69
Clostridia_bacterium	30:137574799	T	C	T	C	-0.17	-0.15	0.14	0.80
Clostridia_bacterium	7:13824320	T	C	T	C	-0.15	0.27	0.15	0.59
Clostridia_bacterium	7:74070680	A	G	A	G	0.13	-0.26	0.17	0.69
Clostridia_bacterium	8:51642766	A	G	A	G	-0.22	1.08	0.06	0.22
Bacteroidaceae_bacterium	1:148034141	T	G	T	G	-0.19	-0.90	0.08	0.19
Bacteroidaceae_bacterium	12:75290050	T	G	T	G	-0.25	0.74	0.06	0.38
Bacteroidaceae_bacterium	15:49694739	C	A	C	A	-0.19	0.75	0.14	0.17
Bacteroidaceae_bacterium	17:11836159	A	C	A	C	-0.16	-0.11	0.13	0.84
Bacteroidaceae_bacterium	17:44906574	G	A	G	A	-0.18	-0.21	0.10	0.71
Bacteroidaceae_bacterium	23:51593541	G	A	G	A	-0.15	0.36	0.15	0.59
Bacteroidaceae_bacterium	24:3530858	C	T	C	T	-0.19	0.80	0.09	0.26
Bacteroidaceae_bacterium	30:90324452	A	C	A	C	-0.28	1.04	0.06	0.22
Bacteroidaceae_bacterium	30:90324469	C	T	C	T	-0.26	1.22	0.06	0.13
Bacteroidaceae_bacterium	9:15496536	C	T	C	T	-0.17	0.76	0.14	0.15
Bacteroidaceae_bacterium	9:15496545	C	T	C	T	-0.18	0.76	0.14	0.15
Bacteroidaceae_bacterium	9:61337023	T	C	T	C	-0.27	-1.24	0.07	0.12
Prevotella_lacticifex	10:37342450	A	G	A	G	-1.20	-0.38	0.09	0.57
Prevotella_lacticifex	10:38458687	G	A	G	A	-1.13	-0.54	0.09	0.42
Prevotella_lacticifex	12:15430296	C	T	C	T	-1.45	-0.46	0.06	0.55
Prevotella_lacticifex	12:22786174	A	G	A	G	-0.91	0.03	0.16	0.94
Prevotella_lacticifex	12:6984081	C	A	C	A	-1.13	-0.36	0.10	0.56
Prevotella_lacticifex	17:66164187	A	G	A	G	-1.39	0.43	0.07	0.45
Prevotella_lacticifex	19:30943825	A	G	A	G	-1.23	1.04	0.07	0.14
Prevotella_lacticifex	19:47464525	C	T	C	T	-0.96	-0.58	0.16	0.20
Prevotella_lacticifex	19:48165383	A	G	A	G	-1.26	0.83	0.09	0.18
Prevotella_lacticifex	19:48193196	G	A	G	A	-1.03	0.89	0.11	0.15
Prevotella_lacticifex	19:6097957	T	C	T	C	-1.20	0.28	0.09	0.70
Prevotella_lacticifex	23:29607710	G	A	G	A	1.17	-0.54	0.14	0.32
Prevotella_lacticifex	23:29607720	G	A	G	A	1.16	-0.37	0.14	0.50
Prevotella_lacticifex	23:29607721	C	A	C	A	1.07	-0.40	0.14	0.47
Prevotella_lacticifex	26:10546335	G	A	G	A	-1.47	0.03	0.07	0.97

Prevotella_lacticifex	26:10607539	T	C	T	C	-1.37	0.70	0.06	0.35
Prevotella_lacticifex	26:10612819	A	G	A	G	-1.53	0.82	0.06	0.28
Prevotella_lacticifex	26:10613785	G	A	G	A	-1.36	0.67	0.07	0.40
Prevotella_lacticifex	5:125926	G	A	G	A	1.07	0.75	0.15	0.24
Prevotella_lacticifex	8:12347603	C	T	C	T	-1.16	0.47	0.13	0.53
Prevotella_mizrahii	1:124976323	A	G	A	G	-1.26	0.48	0.06	0.52
Prevotella_mizrahii	1:127263861	G	A	G	A	-1.29	0.53	0.08	0.39
Prevotella_mizrahii	1:127288937	T	C	T	C	-1.57	0.18	0.06	0.83
Prevotella_mizrahii	10:37342450	A	G	A	G	-1.32	-0.38	0.09	0.57
Prevotella_mizrahii	10:37427478	C	T	C	T	-1.31	-0.16	0.08	0.79
Prevotella_mizrahii	10:38458687	G	A	G	A	-1.17	-0.54	0.09	0.42
Prevotella_mizrahii	10:38458711	T	C	T	C	-1.13	-0.67	0.09	0.33
Prevotella_mizrahii	11:48604680	C	T	C	T	-1.40	1.24	0.07	0.13
Prevotella_mizrahii	11:6115143	A	G	A	G	-1.37	-0.46	0.06	0.58
Prevotella_mizrahii	12:15430296	C	T	C	T	-1.48	-0.46	0.06	0.55
Prevotella_mizrahii	17:66164187	A	G	A	G	-1.46	0.43	0.07	0.45
Prevotella_mizrahii	19:30770670	G	A	G	A	-1.18	0.85	0.08	0.15
Prevotella_mizrahii	19:30943825	A	G	A	G	-1.24	1.04	0.07	0.14
Prevotella_mizrahii	19:47464525	C	T	C	T	-0.97	-0.58	0.16	0.20
Prevotella_mizrahii	19:48165383	A	G	A	G	-1.34	0.83	0.09	0.18
Prevotella_mizrahii	19:48193196	G	A	G	A	-1.09	0.89	0.11	0.15
Prevotella_mizrahii	19:48196165	C	T	C	T	-0.99	0.99	0.11	0.09
Prevotella_mizrahii	19:61612519	C	A	C	A	-1.66	1.02	0.05	0.26
Prevotella_mizrahii	20:13854960	A	G	A	G	-1.52	1.98	0.06	0.02
Prevotella_mizrahii	22:27571745	A	G	A	G	-1.28	0.13	0.08	0.84
Prevotella_mizrahii	22:27598590	C	T	C	T	-1.23	0.41	0.07	0.54
Prevotella_mizrahii	22:27599348	C	T	C	T	-1.27	0.10	0.08	0.89
Prevotella_mizrahii	23:29607710	G	A	G	A	1.13	-0.54	0.14	0.32
Prevotella_mizrahii	26:10546335	G	A	G	A	-1.69	0.03	0.07	0.97
Prevotella_mizrahii	26:10563485	G	A	G	A	-1.69	0.47	0.06	0.57
Prevotella_mizrahii	26:10593453	G	T	G	T	-1.61	1.22	0.07	0.13
Prevotella_mizrahii	26:10605943	T	C	T	C	-1.26	-0.80	0.08	0.24
Prevotella_mizrahii	26:10607539	T	C	T	C	-1.58	0.70	0.06	0.35
Prevotella_mizrahii	26:10609431	T	C	T	C	-1.64	0.51	0.06	0.56
Prevotella_mizrahii	26:10610939	T	C	T	C	-1.43	0.12	0.07	0.87
Prevotella_mizrahii	26:10612819	A	G	A	G	-1.74	0.82	0.06	0.28
Prevotella_mizrahii	26:10613785	G	A	G	A	-1.58	0.67	0.07	0.40
Prevotella_mizrahii	26:10616232	A	G	A	G	-1.44	0.22	0.07	0.76
Prevotella_mizrahii	26:10629385	C	T	C	T	-1.44	0.08	0.06	0.93
Prevotella_mizrahii	26:24313903	C	A	C	A	-1.30	0.49	0.09	0.59
Prevotella_mizrahii	4:10558060	T	C	T	C	-1.22	-0.65	0.09	0.29
Prevotella_mizrahii	4:116032750	A	C	A	C	-1.32	0.57	0.06	0.39
Prevotella_mizrahii	4:45932932	A	C	A	C	-1.14	0.44	0.10	0.49
Moviridae_sp.	11:8797300	C	T	C	T	0.41	-1.17	0.10	0.08

Moviridae_sp.	20:66975191	G	A	G	A	-0.32	-0.78	0.17	0.17
Moviridae_sp.	21:57469142	T	C	T	C	-0.34	0.33	0.15	0.52
Moviridae_sp.	21:57469148	T	C	T	C	-0.34	0.21	0.15	0.68
Moviridae_sp.	23:11887987	G	A	G	A	0.39	0.11	0.09	0.90
Moviridae_sp.	23:21829710	A	G	A	G	0.30	-0.25	0.17	0.65
Moviridae_sp.	23:21829715	G	A	G	A	0.30	-0.26	0.17	0.64
Moviridae_sp.	24:11920980	T	G	T	G	0.41	-0.34	0.09	0.63
Moviridae_sp.	24:11920999	T	G	T	G	0.44	-0.21	0.08	0.77
Moviridae_sp.	5:47090295	A	G	A	G	-0.38	-0.72	0.13	0.30
unclassified_g__Prevotella	12:49391967	G	A	G	A	-0.31	-0.37	0.10	0.58
unclassified_g__Prevotella	13:44925741	T	C	T	C	-0.25	-0.02	0.15	0.98
unclassified_g__Prevotella	19:48165383	A	G	A	G	-0.34	0.83	0.09	0.18
unclassified_g__Prevotella	19:48196054	C	T	C	T	-0.29	0.36	0.12	0.53
unclassified_g__Prevotella	19:61415287	C	T	C	T	-0.33	-0.67	0.07	0.46
unclassified_g__Prevotella	3:40314450	C	T	C	T	0.28	0.30	0.15	0.61
unclassified_g__Prevotella	30:106456336	C	A	C	A	-0.40	-0.76	0.06	0.41
unclassified_g__Prevotella	9:61337023	T	C	T	C	-0.35	-1.24	0.07	0.12
Prevotella_bryantii	10:101885293	G	A	G	A	-0.48	1.23	0.13	0.05
Prevotella_bryantii	11:104324166	T	C	T	C	-0.63	0.10	0.08	0.89
Prevotella_bryantii	15:56425168	T	G	T	G	-0.67	-0.08	0.06	0.93
Prevotella_bryantii	16:47814594	C	T	C	T	-0.60	1.45	0.07	0.07
Prevotella_bryantii	16:48677272	A	C	A	C	-0.59	0.86	0.07	0.29
Prevotella_bryantii	19:45861607	A	G	A	G	-0.55	0.74	0.10	0.18
Prevotella_bryantii	19:48165383	A	G	A	G	-0.60	0.83	0.09	0.18
Prevotella_bryantii	19:48193196	G	A	G	A	-0.50	0.89	0.11	0.15
Prevotella_bryantii	19:48196054	C	T	C	T	-0.47	0.36	0.12	0.53
Prevotella_bryantii	19:48196102	A	G	A	G	-0.47	0.74	0.11	0.21
Prevotella_bryantii	19:61415287	C	T	C	T	-0.59	-0.67	0.07	0.46
Prevotella_bryantii	22:15385053	G	A	G	A	-0.63	-1.26	0.07	0.16
Prevotella_bryantii	22:8503957	G	A	G	A	-0.71	-0.65	0.07	0.36
Prevotella_bryantii	26:10612819	A	G	A	G	-0.65	0.82	0.06	0.28
Prevotella_bryantii	26:23891716	C	T	C	T	-0.57	0.48	0.09	0.56
Prevotella_bryantii	26:23892717	C	T	C	T	-0.62	0.61	0.07	0.36
Prevotella_bryantii	26:24032549	A	G	A	G	-0.64	-0.28	0.08	0.74
Prevotella_bryantii	26:24313903	C	A	C	A	-0.60	0.49	0.09	0.59
Prevotella_bryantii	26:24389778	A	C	A	C	-0.51	0.51	0.13	0.37
Prevotella_bryantii	26:24621267	G	A	G	A	-0.46	0.68	0.16	0.25
Prevotella_bryantii	27:4443345	G	A	G	A	-0.67	-0.15	0.07	0.83
Prevotella_bryantii	27:6342002	A	G	A	G	-0.54	1.03	0.13	0.14
Prevotella_bryantii	28:41486694	A	G	A	G	-0.70	0.58	0.06	0.47
Prevotella_bryantii	28:43550230	A	G	A	G	-0.82	-0.46	0.05	0.52
Prevotella_bryantii	7:34551711	G	A	G	A	-0.67	-0.44	0.07	0.58
Alphaproteobacteria_bacterium	1:127288937	T	C	T	C	0.36	0.18	0.06	0.83
Alphaproteobacteria_bacterium	12:12534883	C	T	C	T	0.38	-1.38	0.06	0.04

Alphaproteobacteria_bacterium	12:81255565	C	T	C	T	-0.21	-0.25	0.18	0.64
Alphaproteobacteria_bacterium	27:27035899	G	A	G	A	-0.29	0.68	0.10	0.35
Alphaproteobacteria_bacterium	28:43550160	C	T	C	T	0.42	-0.32	0.05	0.67
Alphaproteobacteria_bacterium	30:134928785	A	G	A	G	0.36	2.58	0.06	0.00
Alphaproteobacteria_bacterium	5:12493993	A	G	A	G	0.36	1.00	0.05	0.24
Alphaproteobacteria_bacterium	5:92635761	C	T	C	T	0.32	-0.02	0.06	0.97
Alphaproteobacteria_bacterium	5:92637241	G	A	G	A	0.30	-0.74	0.06	0.28
Prevotella_copri	1:141189992	C	T	C	T	-0.35	0.15	0.10	0.83
Prevotella_copri	1:141190044	G	A	G	A	-0.35	-0.56	0.10	0.40
Prevotella_copri	12:49391967	G	A	G	A	-0.34	-0.37	0.10	0.58
Prevotella_copri	19:47464525	C	T	C	T	-0.29	-0.58	0.16	0.20
Prevotella_copri	19:48165383	A	G	A	G	-0.41	0.83	0.09	0.18
Prevotella_copri	19:61415287	C	T	C	T	-0.37	-0.67	0.07	0.46
Prevotella_copri	23:29607710	G	A	G	A	0.36	-0.54	0.14	0.32
Prevotella_copri	23:29607720	G	A	G	A	0.36	-0.37	0.14	0.50
Prevotella_copri	23:29607721	C	A	C	A	0.32	-0.40	0.14	0.47
Prevotella_copri	26:10612819	A	G	A	G	-0.39	0.82	0.06	0.28
Prevotella_copri	26:24313903	C	A	C	A	-0.37	0.49	0.09	0.59
Butyrivibrio_sp.	12:50153846	C	A	C	A	0.20	1.09	0.08	0.12
Butyrivibrio_sp.	5:46293333	C	T	C	T	0.24	0.03	0.09	0.97
Butyrivibrio_sp.	5:59920983	C	T	C	T	-0.18	-0.12	0.14	0.86
Aeriscardovia_sp.	11:2282193	G	A	G	A	-0.19	1.09	0.06	0.23
Aeriscardovia_sp.	14:78248542	C	A	C	A	-0.16	-0.45	0.08	0.47
Aeriscardovia_sp.	17:44374245	G	T	G	T	-0.16	1.40	0.08	0.08
Aeriscardovia_sp.	22:41663644	T	C	T	C	-0.15	-1.07	0.07	0.17
Aeriscardovia_sp.	5:2886185	T	G	T	G	-0.17	0.00	0.08	1.00
Aeriscardovia_sp.	5:71876643	T	C	T	C	-0.19	1.54	0.05	0.05
Aeriscardovia_sp.	9:23090091	G	A	G	A	-0.12	0.30	0.15	0.63
Aeriscardovia_sp.	9:26906817	A	G	A	G	-0.16	0.48	0.09	0.48
Bacteriophage_sp.	21:57469142	T	C	T	C	-0.29	0.33	0.15	0.52
Bacteriophage_sp.	21:57469148	T	C	T	C	-0.29	0.21	0.15	0.68
Bacteriophage_sp.	22:10535843	G	A	G	A	0.39	-0.26	0.07	0.76
Bacteriophage_sp.	23:11887987	G	A	G	A	0.34	0.11	0.09	0.90
Bacteriophage_sp.	23:21829710	A	G	A	G	0.26	-0.25	0.17	0.65
Bacteriophage_sp.	23:26778629	C	T	C	T	0.40	0.43	0.07	0.58
Bacteriophage_sp.	5:47090295	A	G	A	G	-0.33	-0.72	0.13	0.30
CrAss-like_virus_sp.	1:116617977	A	G	A	G	0.51	-0.23	0.10	0.74
CrAss-like_virus_sp.	1:147022501	G	A	G	A	0.52	1.60	0.07	0.08
CrAss-like_virus_sp.	21:57469142	T	C	T	C	-0.36	0.33	0.15	0.52
CrAss-like_virus_sp.	21:57469148	T	C	T	C	-0.37	0.21	0.15	0.68
CrAss-like_virus_sp.	22:49811	T	C	T	C	-0.51	-0.43	0.09	0.55
CrAss-like_virus_sp.	22:49812	C	A	C	A	-0.51	-0.43	0.09	0.55
CrAss-like_virus_sp.	23:11887987	G	A	G	A	0.46	0.11	0.09	0.90
CrAss-like_virus_sp.	8:10055952	A	G	A	G	0.55	0.45	0.08	0.55

Intestinibaculum_porci	10:26648928	T	C	T	C	1.12	-0.43	0.16	0.38
Intestinibaculum_porci	15:82976077	G	A	G	A	1.55	-0.61	0.09	0.34
Intestinibaculum_porci	19:62450263	T	G	T	G	1.56	-0.20	0.10	0.80
Intestinibaculum_porci	25:3012882	G	A	G	A	1.23	0.72	0.17	0.18
Intestinibaculum_porci	7:41045496	T	G	T	G	1.98	0.02	0.06	0.98
Proteobacteria_bacterium	12:61711133	A	G	A	G	-0.61	0.70	0.10	0.31
Proteobacteria_bacterium	12:71128195	G	T	G	T	0.45	0.68	0.19	0.11
Proteobacteria_bacterium	17:66977150	A	G	A	G	0.91	-0.78	0.05	0.42
Proteobacteria_bacterium	17:68718619	G	A	G	A	1.00	-1.38	0.05	0.08
Proteobacteria_bacterium	2:107717878	G	A	G	A	0.67	0.80	0.07	0.24
Proteobacteria_bacterium	20:68989642	G	A	G	A	-0.58	-0.13	0.09	0.88
Proteobacteria_bacterium	21:29152445	T	C	T	C	0.77	0.27	0.05	0.68
Proteobacteria_bacterium	21:45519586	C	T	C	T	0.67	0.04	0.07	0.97
Proteobacteria_bacterium	21:45543159	A	G	A	G	0.69	0.72	0.07	0.40
Proteobacteria_bacterium	23:44234971	G	A	G	A	0.98	-0.25	0.06	0.73
Proteobacteria_bacterium	26:28009815	T	C	T	C	0.52	0.32	0.14	0.55
Proteobacteria_bacterium	28:1013786	T	C	T	C	0.47	-0.69	0.17	0.15
Proteobacteria_bacterium	29:4602396	G	A	G	A	0.76	-0.45	0.08	0.51
Proteobacteria_bacterium	4:50334539	A	G	A	G	0.64	0.25	0.11	0.61
Proteobacteria_bacterium	4:50334788	T	C	T	C	0.54	0.74	0.13	0.18
Proteobacteria_bacterium	5:10567974	A	G	A	G	0.59	-0.72	0.14	0.18
Proteobacteria_bacterium	6:31394712	A	G	A	G	0.73	-0.72	0.06	0.32
Proteobacteria_bacterium	6:32569332	C	A	C	A	0.79	1.60	0.06	0.01
Proteobacteria_bacterium	6:32569344	A	C	A	C	0.74	1.10	0.07	0.10
Proteobacteria_bacterium	6:33045647	A	C	A	C	0.78	0.93	0.05	0.35
Proteobacteria_bacterium	6:33045661	C	T	C	T	0.78	0.78	0.05	0.40
Proteobacteria_bacterium	6:66181168	G	A	G	A	0.52	-0.13	0.17	0.81
Proteobacteria_bacterium	8:71512173	C	T	C	T	0.86	0.89	0.07	0.19
Proteobacteria_bacterium	8:71512257	G	A	G	A	0.90	0.90	0.05	0.28
Proteobacteria_bacterium	8:71513246	G	T	G	T	0.87	0.32	0.06	0.69
Proteobacteria_bacterium	8:71513265	A	G	A	G	0.84	0.38	0.06	0.62
Proteobacteria_bacterium	8:71514473	C	T	C	T	0.85	1.39	0.06	0.07
Proteobacteria_bacterium	8:71514685	C	A	C	A	0.82	1.03	0.06	0.18
Proteobacteria_bacterium	8:71604926	C	T	C	T	1.02	1.09	0.05	0.22
Proteobacteria_bacterium	8:76704790	G	A	G	A	0.76	-0.79	0.08	0.20
bacterium_P201	1:141190044	G	A	G	A	-0.25	-0.56	0.10	0.40
bacterium_P201	12:49391967	G	A	G	A	-0.25	-0.37	0.10	0.58
bacterium_P201	15:49694739	C	A	C	A	-0.21	0.75	0.14	0.17
bacterium_P201	19:48165383	A	G	A	G	-0.28	0.83	0.09	0.18
bacterium_P201	19:48196054	C	T	C	T	-0.23	0.36	0.12	0.53
bacterium_P201	19:61415287	C	T	C	T	-0.27	-0.67	0.07	0.46
bacterium_P201	23:27981872	C	T	C	T	-0.23	-0.87	0.10	0.23
bacterium_P201	24:3523350	G	A	G	A	-0.27	1.41	0.06	0.04
bacterium_P201	3:40314450	C	T	C	T	0.21	0.30	0.15	0.61

bacterium_P201	9:23090091	G	A	G	A	-0.20	0.30	0.15	0.63
bacterium_P201	9:61337023	T	C	T	C	-0.32	-1.24	0.07	0.12
Elusimicrobia_bacterium	25:1196760	G	A	G	A	-0.39	-0.28	0.16	0.60
Elusimicrobia_bacterium	27:27035899	G	A	G	A	-0.51	0.68	0.10	0.35
Elusimicrobia_bacterium	30:18807501	G	T	G	T	-0.57	-0.05	0.10	0.95
Elusimicrobia_bacterium	5:7301034	G	A	G	A	-0.64	-0.71	0.06	0.41
Elusimicrobia_bacterium	8:39252223	C	T	C	T	-0.66	0.08	0.06	0.92
Elusimicrobia_bacterium	8:39569999	G	A	G	A	-0.54	0.05	0.08	0.95
Prevotella_brevis	1:148034141	T	G	T	G	-0.27	-0.90	0.08	0.19
Prevotella_brevis	15:38701025	T	C	T	C	-0.26	-0.81	0.08	0.30
Prevotella_brevis	15:49694739	C	A	C	A	-0.22	0.75	0.14	0.17
Prevotella_brevis	15:49701531	C	T	C	T	-0.23	0.47	0.12	0.40
Prevotella_brevis	16:64797085	A	G	A	G	-0.35	-0.86	0.06	0.32
Prevotella_brevis	17:44374245	G	T	G	T	-0.28	1.40	0.08	0.08
Prevotella_brevis	17:69950708	C	T	C	T	-0.23	0.37	0.11	0.52
Prevotella_brevis	19:48196054	C	T	C	T	-0.22	0.36	0.12	0.53
Prevotella_brevis	2:57864500	A	G	A	G	-0.26	0.43	0.10	0.54
Prevotella_brevis	2:62767181	G	A	G	A	-0.22	-0.27	0.11	0.64
Prevotella_brevis	23:20014447	C	A	C	A	-0.32	-0.58	0.07	0.38
Prevotella_brevis	9:61337023	T	C	T	C	-0.32	-1.24	0.07	0.12
Prevotella_sp._AGR2160	1:127263861	G	A	G	A	-1.19	0.53	0.08	0.39
Prevotella_sp._AGR2160	1:127288937	T	C	T	C	-1.40	0.18	0.06	0.83
Prevotella_sp._AGR2160	10:37342450	A	G	A	G	-1.20	-0.38	0.09	0.57
Prevotella_sp._AGR2160	10:37855031	C	A	C	A	-1.20	-0.88	0.07	0.18
Prevotella_sp._AGR2160	10:38458687	G	A	G	A	-1.09	-0.54	0.09	0.42
Prevotella_sp._AGR2160	10:38458711	T	C	T	C	-1.06	-0.67	0.09	0.33
Prevotella_sp._AGR2160	12:15430296	C	T	C	T	-1.40	-0.46	0.06	0.55
Prevotella_sp._AGR2160	17:66164187	A	G	A	G	-1.35	0.43	0.07	0.45
Prevotella_sp._AGR2160	19:30770670	G	A	G	A	-1.10	0.85	0.08	0.15
Prevotella_sp._AGR2160	19:30943825	A	G	A	G	-1.19	1.04	0.07	0.14
Prevotella_sp._AGR2160	19:47464525	C	T	C	T	-0.93	-0.58	0.16	0.20
Prevotella_sp._AGR2160	19:48165383	A	G	A	G	-1.25	0.83	0.09	0.18
Prevotella_sp._AGR2160	20:13854960	A	G	A	G	-1.43	1.98	0.06	0.02
Prevotella_sp._AGR2160	20:15830337	C	T	C	T	-1.21	0.06	0.09	0.94
Prevotella_sp._AGR2160	20:16355871	G	A	G	A	-1.20	0.74	0.10	0.33
Prevotella_sp._AGR2160	22:27571745	A	G	A	G	-1.21	0.13	0.08	0.84
Prevotella_sp._AGR2160	22:27598590	C	T	C	T	-1.14	0.41	0.07	0.54
Prevotella_sp._AGR2160	22:30132918	T	G	T	G	-0.95	0.65	0.14	0.21
Prevotella_sp._AGR2160	23:29607710	G	A	G	A	1.11	-0.54	0.14	0.32
Prevotella_sp._AGR2160	23:29607720	G	A	G	A	1.09	-0.37	0.14	0.50
Prevotella_sp._AGR2160	26:10546335	G	A	G	A	-1.55	0.03	0.07	0.97
Prevotella_sp._AGR2160	26:10563485	G	A	G	A	-1.54	0.47	0.06	0.57
Prevotella_sp._AGR2160	26:10593453	G	T	G	T	-1.43	1.22	0.07	0.13
Prevotella_sp._AGR2160	26:10607539	T	C	T	C	-1.44	0.70	0.06	0.35

Prevotella_sp._AGR2160	26:10610939	T	C	T	C	-1.32	0.12	0.07	0.87
Prevotella_sp._AGR2160	26:10612819	A	G	A	G	-1.60	0.82	0.06	0.28
Prevotella_sp._AGR2160	26:10613785	G	A	G	A	-1.48	0.67	0.07	0.40
Prevotella_sp._AGR2160	26:10616232	A	G	A	G	-1.28	0.22	0.07	0.76
Prevotella_sp._AGR2160	28:37756141	A	G	A	G	-1.03	-0.51	0.10	0.28
Prevotella_sp._AGR2160	4:10558060	T	C	T	C	-1.13	-0.65	0.09	0.29
Prevotella_sp._AGR2160	4:116032750	A	C	A	C	-1.26	0.57	0.06	0.39
Prevotella_sp._AGR2160	8:12347603	C	T	C	T	-1.10	0.47	0.13	0.53
Prevotella_sp._AGR2160	8:12362990	C	T	C	T	-1.05	0.08	0.12	0.91
Klebsiella_pneumoniae	1:147022501	G	A	G	A	0.32	1.60	0.07	0.08
Klebsiella_pneumoniae	12:77711280	A	G	A	G	0.36	-0.91	0.08	0.19
Klebsiella_pneumoniae	18:22291164	T	C	T	C	0.25	0.97	0.14	0.07
Klebsiella_pneumoniae	22:10535843	G	A	G	A	0.34	-0.26	0.07	0.76
Klebsiella_pneumoniae	22:10535857	C	A	C	A	0.34	-0.16	0.07	0.84
Klebsiella_pneumoniae	23:11887987	G	A	G	A	0.30	0.11	0.09	0.90
Klebsiella_pneumoniae	28:10313566	G	A	G	A	0.41	-0.30	0.06	0.75
Klebsiella_pneumoniae	28:10578704	G	A	G	A	0.40	-1.18	0.05	0.12
Klebsiella_pneumoniae	28:10616705	A	G	A	G	0.38	-0.71	0.06	0.34
Klebsiella_pneumoniae	30:101156473	G	T	G	T	0.35	-0.47	0.07	0.52
Klebsiella_pneumoniae	9:24026748	C	T	C	T	0.34	1.48	0.08	0.09
Spirochaetales_bacterium	10:66835529	G	A	G	A	0.24	-0.72	0.10	0.26
Spirochaetales_bacterium	11:8792722	A	G	A	G	0.29	-1.12	0.08	0.27
Spirochaetales_bacterium	23:26778629	C	T	C	T	0.31	0.43	0.07	0.58
Spirochaetales_bacterium	24:11920980	T	G	T	G	0.29	-0.34	0.09	0.63
Spirochaetales_bacterium	24:11920999	T	G	T	G	0.32	-0.21	0.08	0.77
Spirochaetales_bacterium	5:47090295	A	G	A	G	-0.25	-0.72	0.13	0.30
Spirochaetales_bacterium	8:10055954	G	A	G	A	0.33	0.45	0.07	0.55
Spirochaetales_bacterium	8:10055956	C	T	C	T	0.34	0.45	0.07	0.55
Ruminococcaceae_bacterium_P7	1:139852410	C	T	C	T	0.57	-0.12	0.11	0.84
Ruminococcaceae_bacterium_P7	1:145572251	G	A	G	A	-0.60	-0.83	0.10	0.37
Ruminococcaceae_bacterium_P7	12:14639052	C	T	C	T	0.42	-0.50	0.18	0.30
Ruminococcaceae_bacterium_P7	12:32987405	C	T	C	T	0.50	0.08	0.16	0.88
Ruminococcaceae_bacterium_P7	16:49877713	G	C	G	C	-0.76	0.73	0.06	0.31
Ruminococcaceae_bacterium_P7	22:26339371	T	C	T	C	0.72	-0.45	0.06	0.54
Ruminococcaceae_bacterium_P7	22:27571745	A	G	A	G	0.72	0.13	0.08	0.84
Ruminococcaceae_bacterium_P7	22:27591326	G	A	G	A	0.67	0.17	0.08	0.82
Ruminococcaceae_bacterium_P7	22:27598590	C	T	C	T	0.63	0.41	0.07	0.54
Ruminococcaceae_bacterium_P7	22:27598777	C	T	C	T	0.69	0.46	0.07	0.38
Ruminococcaceae_bacterium_P7	22:27599348	C	T	C	T	0.70	0.10	0.08	0.89
Ruminococcaceae_bacterium_P7	5:110064170	A	G	A	G	-0.79	0.05	0.05	0.95
Ruminococcaceae_bacterium_P7	8:83392667	G	A	G	A	-0.48	-0.13	0.15	0.80
Ruminococcaceae_bacterium_P7	9:85766025	A	G	A	G	-0.87	-0.83	0.06	0.25
Pseudobutyrivibrio_sp.	11:19231457	G	A	G	A	0.34	0.06	0.06	0.94
Pseudobutyrivibrio_sp.	11:20607601	A	C	A	C	0.28	0.33	0.08	0.66

Pseudobutyrivibrio_sp.	12:3321978	C	T	C	T	0.23	-0.01	0.10	0.99
Pseudobutyrivibrio_sp.	12:3404870	C	T	C	T	0.31	-1.04	0.08	0.18
Pseudobutyrivibrio_sp.	12:53664098	G	A	G	A	0.23	0.50	0.11	0.40
Pseudobutyrivibrio_sp.	12:83479120	G	A	G	A	0.18	0.31	0.17	0.58
Pseudobutyrivibrio_sp.	16:7158688	A	G	A	G	0.32	-0.63	0.06	0.47
Pseudobutyrivibrio_sp.	18:22309346	G	A	G	A	0.36	2.16	0.06	0.01
Pseudobutyrivibrio_sp.	2:130417240	A	G	A	G	0.30	-0.16	0.06	0.81
Pseudobutyrivibrio_sp.	2:130417241	A	G	A	G	0.30	-0.15	0.06	0.82
Pseudobutyrivibrio_sp.	2:15548429	G	A	G	A	0.28	0.93	0.07	0.29
Pseudobutyrivibrio_sp.	20:18880589	G	A	G	A	0.25	-0.38	0.10	0.63
Pseudobutyrivibrio_sp.	23:40616772	G	A	G	A	0.36	-0.55	0.06	0.47
Pseudobutyrivibrio_sp.	4:113157209	A	G	A	G	0.37	0.49	0.05	0.61
Pseudobutyrivibrio_sp.	4:24133351	C	T	C	T	0.30	0.15	0.06	0.87
Pseudobutyrivibrio_sp.	4:49247816	G	A	G	A	0.37	-0.15	0.06	0.86
Pseudobutyrivibrio_sp.	7:70509616	C	T	C	T	0.34	0.42	0.06	0.56
Pseudobutyrivibrio_sp.	7:70511362	G	A	G	A	0.37	0.39	0.06	0.63
Pseudobutyrivibrio_sp.	7:70549186	T	C	T	C	0.36	1.12	0.05	0.21
Pseudobutyrivibrio_sp.	9:27452642	T	C	T	C	0.27	0.61	0.09	0.43
Pseudobutyrivibrio_sp.	9:99001061	G	T	G	T	0.24	-0.69	0.11	0.44
Methanobrevibacter_millerae	11:67480312	T	C	T	C	0.42	-0.30	0.10	0.62
Methanobrevibacter_millerae	11:67482518	G	T	G	T	0.35	0.53	0.12	0.38
Methanobrevibacter_millerae	11:67482711	C	T	C	T	0.37	0.52	0.11	0.43
Methanobrevibacter_millerae	11:67484683	A	G	A	G	0.38	0.61	0.11	0.32
Methanobrevibacter_millerae	11:67488014	C	A	C	A	0.37	-0.28	0.11	0.65
Methanobrevibacter_millerae	11:67490174	T	C	T	C	0.50	0.61	0.07	0.44
Methanobrevibacter_millerae	11:68991977	T	C	T	C	0.42	-0.81	0.07	0.34
Methanobrevibacter_millerae	11:69336500	C	A	C	A	0.34	-0.01	0.10	0.99
Methanobrevibacter_millerae	14:67006568	T	C	T	C	0.44	0.70	0.07	0.28
Methanobrevibacter_millerae	14:78248542	C	A	C	A	0.42	-0.45	0.08	0.47
Methanobrevibacter_millerae	14:78249468	A	G	A	G	0.39	-0.48	0.08	0.41
Methanobrevibacter_millerae	14:81631401	C	T	C	T	0.30	0.40	0.14	0.50
Methanobrevibacter_millerae	17:12036943	A	G	A	G	0.42	-0.17	0.07	0.78
Methanobrevibacter_millerae	17:12269881	G	A	G	A	0.40	-0.54	0.09	0.46
Methanobrevibacter_millerae	2:126812966	T	C	T	C	0.34	0.55	0.13	0.35
Methanobrevibacter_millerae	2:56308229	T	C	T	C	0.54	0.65	0.06	0.33
Methanobrevibacter_millerae	2:57511976	C	T	C	T	0.36	0.66	0.12	0.30
Methanobrevibacter_millerae	2:62467087	G	A	G	A	0.44	-0.17	0.09	0.81
Methanobrevibacter_millerae	2:63093704	G	A	G	A	0.41	0.45	0.09	0.52
Methanobrevibacter_millerae	2:63093713	G	A	G	A	0.41	0.34	0.09	0.61
Methanobrevibacter_millerae	2:64128284	C	T	C	T	0.42	0.28	0.08	0.69
Methanobrevibacter_millerae	2:64283787	G	A	G	A	0.44	-0.19	0.08	0.79
Methanobrevibacter_millerae	22:59057038	G	T	G	T	0.35	0.90	0.12	0.23
Methanobrevibacter_millerae	23:32293951	C	T	C	T	0.36	-0.14	0.11	0.86
Methanobrevibacter_millerae	23:32889397	T	G	T	G	0.40	0.21	0.10	0.76

Methanobrevibacter_millerae	23:34010302	C	T	C	T	0.33	-0.45	0.15	0.46
Methanobrevibacter_millerae	23:34231684	A	G	A	G	0.33	-0.24	0.13	0.75
Methanobrevibacter_millerae	24:19129345	A	C	A	C	0.46	1.23	0.09	0.12
Methanobrevibacter_millerae	24:39338253	T	C	T	C	0.53	-0.82	0.05	0.33
Methanobrevibacter_millerae	26:24313903	C	A	C	A	0.43	0.49	0.09	0.59
Methanocorpusculum_sp.	1:102211459	A	C	A	C	-0.65	-0.78	0.09	0.32
Methanocorpusculum_sp.	1:102300360	C	T	C	T	-0.58	-0.83	0.12	0.18
Methanocorpusculum_sp.	1:102438068	C	T	C	T	-0.65	-1.06	0.09	0.18
Methanocorpusculum_sp.	1:102458968	G	A	G	A	-0.68	-1.45	0.07	0.06
Methanocorpusculum_sp.	1:102465446	G	A	G	A	-0.66	-0.92	0.10	0.19
Methanocorpusculum_sp.	1:102468952	A	G	A	G	-0.64	-0.35	0.09	0.63
Methanocorpusculum_sp.	1:102469725	A	C	A	C	-0.74	-0.50	0.07	0.54
Methanocorpusculum_sp.	1:127263861	G	A	G	A	-0.65	0.53	0.08	0.39
Methanocorpusculum_sp.	11:23110028	C	T	C	T	-0.73	1.25	0.06	0.12
Methanocorpusculum_sp.	11:32821464	A	G	A	G	-0.78	1.04	0.06	0.27
Methanocorpusculum_sp.	11:651701	A	G	A	G	-0.73	-0.95	0.06	0.25
Methanocorpusculum_sp.	15:82781818	G	A	G	A	-1.11	2.30	0.05	0.01
Methanocorpusculum_sp.	17:28546014	C	T	C	T	-0.93	0.38	0.05	0.60
Methanocorpusculum_sp.	17:32492922	T	G	T	G	-0.66	-0.64	0.09	0.38
Methanocorpusculum_sp.	17:34168018	A	G	A	G	-0.87	-0.81	0.05	0.31
Methanocorpusculum_sp.	21:27319998	T	G	T	G	-0.67	-0.40	0.09	0.54
Methanocorpusculum_sp.	23:29432095	G	A	G	A	-0.70	0.51	0.07	0.50
Methanocorpusculum_sp.	26:10593453	G	T	G	T	-0.77	1.22	0.07	0.13
Methanocorpusculum_sp.	26:42324478	T	C	T	C	-0.52	0.52	0.15	0.43
Methanocorpusculum_sp.	26:42773010	G	A	G	A	-0.78	-0.45	0.08	0.54
Methanocorpusculum_sp.	28:28108934	A	G	A	G	-0.60	0.36	0.12	0.61
Methanocorpusculum_sp.	29:24206314	C	T	C	T	-0.66	-1.25	0.09	0.07
Methanocorpusculum_sp.	29:8277336	A	C	A	C	-0.85	-0.47	0.07	0.56
Methanocorpusculum_sp.	29:8277337	G	T	G	T	-0.86	-0.43	0.07	0.60
Methanocorpusculum_sp.	29:8277357	C	T	C	T	-0.85	-0.72	0.07	0.40
Methanocorpusculum_sp.	3:31924042	C	T	C	T	-0.74	0.55	0.07	0.50
Methanocorpusculum_sp.	3:31973383	T	C	T	C	-0.82	-0.38	0.07	0.70
Methanocorpusculum_sp.	30:134968978	G	A	G	A	-0.92	0.36	0.05	0.61
Methanocorpusculum_sp.	5:1064658	C	T	C	T	-0.82	0.10	0.06	0.89
Methanocorpusculum_sp.	5:114553033	G	A	G	A	-0.77	0.06	0.05	0.95
Methanocorpusculum_sp.	6:38631445	G	T	G	T	-0.65	0.05	0.09	0.96
Methanocorpusculum_sp.	7:106245395	A	G	A	G	-0.90	-0.86	0.06	0.33
Methanocorpusculum_sp.	9:72717712	T	C	T	C	-0.86	-0.79	0.06	0.31
Synergistaceae_bacterium	19:47464525	C	T	C	T	0.18	-0.58	0.16	0.20
Synergistaceae_bacterium	24:3529020	G	A	G	A	0.22	0.85	0.08	0.18
Synergistaceae_bacterium	27:27035899	G	A	G	A	-0.21	0.68	0.10	0.35
Synergistaceae_bacterium	3:40314450	C	T	C	T	-0.21	0.30	0.15	0.61
Synergistaceae_bacterium	30:94187879	A	G	A	G	0.25	0.96	0.06	0.24
Parafannyhessea_umbonata	1:147022501	G	A	G	A	0.89	1.60	0.07	0.08

Parafannyhessea_umbonata	11:7040304	G	A	G	A	0.70	0.43	0.14	0.42
Parafannyhessea_umbonata	12:57095987	A	G	A	G	0.83	-0.84	0.10	0.25
Parafannyhessea_umbonata	15:54900094	G	A	G	A	-0.75	-0.12	0.11	0.87
Parafannyhessea_umbonata	23:11026885	C	T	C	T	0.87	-0.25	0.10	0.66
Parafannyhessea_umbonata	23:13263371	G	A	G	A	1	0	0	1

Table S5. Two-sample MR identifying the causal associations between microbiome features and methane emissons

id.exposure (ivw)	b_methane	se_methane	FDR_methane	b_AP	se_AP	FDR_AP
s__Prevotella_sp.	-0.74	1.38	0.59	-0.18	0.15	0.21
s__Lachnospiraceae_bacterium	-0.47	0.58	0.42	0.21	0.09	0.01
s__archaeon	-0.03	0.21	0.88	0.01	0.04	0.73
s__Clostridia_bacterium	-0.16	0.94	0.86	0.11	0.16	0.49
s__Bacteroidales_bacterium	-0.90	0.72	0.21	0.68	0.11	0.00
s__Oscillospiraceae_bacterium	-0.05	0.81	0.95	0.17	0.12	0.16
s__Bacteroidaceae_bacterium	-1.65	1.03	0.11	-0.24	0.14	0.09
s__Muribaculaceae_bacterium	0.17	0.33	0.62	-0.15	0.05	0.00
s__Ruminococcus_sp.	0.32	0.34	0.35	0.15	0.05	0.00
s__Clostridiales_bacterium	-0.89	1.31	0.50	0.32	0.19	0.10
s__Prevotella_lacticifex	-0.17	0.12	0.14	-0.03	0.02	0.06
s__Prevotella_ruminicola	-0.55	1.21	0.65	0.15	0.16	0.34
s__Methanobrevibacter_sp.	-0.23	1.22	0.85	0.49	0.27	0.07
s__Eubacterium_sp.	1.60	1.42	0.26	0.10	0.22	0.64
s__Acidaminococcaceae_bacterium	0.62	1.12	0.58	-0.01	0.16	0.96
s__Firmicutes_bacterium	0.13	1.57	0.94	0.41	0.22	0.07
s__Siphoviridae_sp.	-0.33	0.76	0.66	-0.24	0.15	0.11
s__bacterium	-0.02	0.95	0.98	-0.22	0.28	0.44
s__Paludibacteraceae_bacterium	-1.57	0.85	0.06	0.02	0.23	0.94
s__Prevotella_mizrahii	-0.24	0.08	0.00	-0.05	0.01	0.00
s__Myoviridae_sp.	-0.41	0.54	0.45	-0.10	0.10	0.30
s__Mogibacterium_sp.	-0.47	0.54	0.38	0.12	0.08	0.15
s__Treponema_sp.	-0.78	0.61	0.20	0.10	0.09	0.28
s__Stylonychia_lemnae	0.03	0.10	0.73	0.01	0.02	0.47
s__unclassified_g__Prevotella	0.44	0.77	0.57	-0.11	0.11	0.33
s__Succinivibrionaceae_bacterium	0.18	0.21	0.39	-0.07	0.03	0.03
s__Erysipelotrichaceae_bacterium	-0.10	0.57	0.86	0.04	0.09	0.62
s__Atopobiaceae_bacterium	0.17	0.54	0.75	0.01	0.09	0.89
s__Prevotella_bryantii	-0.62	0.24	0.01	-0.23	0.04	0.00
s__Alphaproteobacteria_bacterium	-0.14	1.06	0.89	0.40	0.16	0.01
s__Prevotella_sp._ne3005	1.59	1.29	0.22	-0.04	0.19	0.85
s__Prevotella_sp._tc2-28	0.05	0.45	0.92	-0.24	0.12	0.04
s__Prevotella_copri	-0.44	0.54	0.41	-0.08	0.08	0.30
s__Acholeplasmatales_bacterium	0.14	0.43	0.74	0.07	0.06	0.29
s__Butyrivibrio_sp.	2.10	2.03	0.30	0.81	0.70	0.24
s__Blepharisma_stoltei	0.02	0.12	0.85	0.02	0.02	0.32
s__Stentor_coeruleus	0.04	0.15	0.81	0.01	0.02	0.76
s__Candidatus_Saccharibacteria_bacterium	-1.26	1.45	0.39	0.11	0.19	0.55
s__Solobacterium_sp.	0.29	0.61	0.63	0.17	0.11	0.10
s__Aeriscardovia_sp.	-2.12	1.80	0.24	0.19	0.23	0.41
s__Tetrahymena_thermophila	-0.01	0.10	0.94	0.02	0.02	0.33
s__Clostridium_sp.	1.04	1.37	0.45	0.45	0.22	0.04

s__Blautia_sp.	-0.59	0.60	0.33	0.19	0.09	0.03
s__Entodinium_caudatum	0.18	0.21	0.38	0.02	0.04	0.64
s__Succiniclasticum_ruminis	0.07	0.40	0.85	-0.16	0.07	0.01
s__Bacteriophage_sp.	-0.09	0.75	0.91	-0.08	0.13	0.51
s__Sarcina_sp.	-0.43	0.57	0.45	0.21	0.08	0.01
s__Neocallimastix_sp._JGI-2020a	0.01	0.12	0.90	0.02	0.02	0.29
s__Ichthyophthirius_multifiliis	0.01	0.10	0.95	0.00	0.02	0.84
s__Sarcina_sp._DSM_11001	-0.20	0.45	0.66	0.19	0.07	0.00
s__CrAss-like_virus_sp.	0.36	0.51	0.48	0.01	0.08	0.85
s__Prevotella_sp._E2-28	0.29	0.76	0.71	-0.19	0.12	0.11
s__Pseudocohnilembus_persalinus	0.00	0.09	0.96	0.01	0.02	0.46
s__Prevotellaceae_bacterium	-0.11	0.41	0.78	-0.02	0.06	0.77
s__Halteria_grandinella	0.09	0.15	0.55	0.02	0.03	0.44
s__Parasporobacterium_sp.	-0.67	0.61	0.27	0.19	0.09	0.03
s__Intestinibaculum_porci	-0.06	0.20	0.74	-0.02	0.03	0.57
s__Prevotella_sp._tf2-5	0.67	1.05	0.52	0.11	0.16	0.50
s__Paramecium_sonneborni	0.05	0.11	0.64	0.02	0.02	0.39
s__Eggerthellaceae_bacterium	-0.55	0.63	0.38	0.12	0.09	0.20
s__Selenomonas_sp.	1.73	1.00	0.08	-0.20	0.15	0.17
s__Proteobacteria_bacterium	0.33	0.18	0.07	-0.05	0.02	0.05
s__Methanosphaera_sp.	1.92	1.53	0.21	0.08	0.22	0.73
s__bacterium_P201	-0.31	0.96	0.75	-0.17	0.12	0.14
s__Saccharofermentans_sp.	0.06	0.50	0.91	-0.18	0.13	0.15
s__Fibrobacter_sp.	-2.08	0.82	0.01	0.17	0.12	0.15
s__Elusimicrobia_bacterium	0.07	0.55	0.90	0.08	0.08	0.33
s__uncultured_bacterium	-1.75	0.78	0.02	-0.04	0.12	0.76
s__Prevotella_sp._E15-22	0.51	1.13	0.65	0.02	0.16	0.90
s__Prevotella_brevis	0.13	0.82	0.87	-0.30	0.11	0.01
s__Neocallimastix_californiae	-0.02	0.09	0.82	0.01	0.01	0.47
s__Prevotella_sp._AGR2160	-0.21	0.09	0.03	-0.05	0.01	0.00
s__Methanobrevibacter_ruminantium	1.19	1.39	0.39	0.00	0.13	0.99
s__PiroFCMces_sp._E2	0.14	0.13	0.29	0.00	0.03	0.94
s__Ruminococcus_sp._M6(2020)	0.26	0.35	0.46	0.12	0.05	0.02
s__Schwartzia_sp.	-2.97	1.21	0.01	-0.20	0.25	0.42
s__Klebsiella_pneumoniae	-0.19	0.83	0.82	0.14	0.10	0.16
s__Kiritimatiellae_bacterium	0.24	0.56	0.66	0.41	0.11	0.00
s__Spirochaetales_bacterium	0.05	0.89	0.96	0.32	0.13	0.02
s__Prevotella_sp._E13-27	0.24	0.68	0.72	-0.17	0.10	0.08
s__Ruminococcaceae_bacterium_P7	0.22	0.28	0.43	0.12	0.04	0.00
s__Paramecium_tetraurelia	0.08	0.10	0.47	0.01	0.02	0.70
s__Pseudobutyrivibrio_sp.	0.57	0.56	0.31	0.06	0.08	0.51
s__Methanobrevibacter_millerae	0.34	0.31	0.27	0.07	0.04	0.11
s__Methanocorpusculum_sp.	0.18	0.19	0.34	0.04	0.03	0.14
s__Paramecium_primaurelia	0.10	0.09	0.29	0.01	0.02	0.45

s__AnaeroFCMces_robustus	0.18	0.17	0.29	0.01	0.03	0.71
s__Synergistaceae_bacterium	-0.24	1.59	0.88	-0.15	0.20	0.44
s__Methanosphaera_stadtmanae	-0.37	0.63	0.55	0.19	0.09	0.04
s__Methanobrevibacter_olleyae	2.20	1.54	0.15	-0.10	0.15	0.53
s__Lachnoclostridium_sp._MSJ-17	0.30	0.36	0.41	0.10	0.05	0.06
s__Methanobrevibacter_thaueri	0.24	0.45	0.58	0.06	0.07	0.42
s__Selenomonadaceae_bacterium	0.52	1.51	0.73	0.03	0.22	0.89
s__Paramecium_octaurelia	0.07	0.08	0.41	0.00	0.01	0.95
s__Parafannyhessea_umbonata	0.09	0.36	0.81	-0.07	0.05	0.16

Table S6. BLAST annotation results of [NiFe]_Group_1d hydrogenase on the genome of Prevotella bryantii strain

Query*	subject	Alignment_length	mismatches	q.start	q.end	s.start	s.end	evalue	Bit_score
>[NiFe]_Group_1d_Hydro_Hydrogenase_1	GCF_000179055.1	-	-	-	-	-	-	-	-
	GCF_000426585.1	-	-	-	-	-	-	-	-
	GCF_000468635.1	-	-	-	-	-	-	-	-
	GCF_000613565.1	-	-	-	-	-	-	-	-
	GCF_022024235.1	-	-	-	-	-	-	-	-
	GCF_900110745.1	-	-	-	-	-	-	-	-
	GCF_963552095.1	-	-	-	-	-	-	-	-
>[NiFe]_Group_1d_Hydro_Hydrogenase_2	GCF_000179055.1	94	62	14	107	3683	3405	0.00	46.2
	GCF_000426585.1	-	-	-	-	-	-	-	-
	GCF_000468635.1	-	-	-	-	-	-	-	-
	GCF_000613565.1	-	-	-	-	-	-	-	-
	GCF_022024235.1	94	62	14	107	231089	231367	0.00	46.2
	GCF_900110745.1	94	62	14	107	223865	224143	0.00	46.2
	GCF_963552095.1	-	-	-	-	-	-	-	-
>[NiFe]_Group_1d_Hydro_Hydrogenase_3	GCF_000179055.1	-	-	-	-	-	-	-	-
	GCF_000426585.1	-	-	-	-	-	-	-	-
	GCF_000468635.1	-	-	-	-	-	-	-	-
	GCF_000613565.1	-	-	-	-	-	-	-	-
	GCF_022024235.1	-	-	-	-	-	-	-	-
	GCF_900110745.1	-	-	-	-	-	-	-	-
	GCF_963552095.1	-	-	-	-	-	-	-	-
>[NiFe]_Group_1d_Hydro_Hydrogenase_4	GCF_000179055.1	-	-	-	-	-	-	-	-
	GCF_000426585.1	-	-	-	-	-	-	-	-
	GCF_000468635.1	-	-	-	-	-	-	-	-
	GCF_000613565.1	-	-	-	-	-	-	-	-
	GCF_022024235.1	-	-	-	-	-	-	-	-
	GCF_900110745.1	-	-	-	-	-	-	-	-
	GCF_963552095.1	-	-	-	-	-	-	-	-
>[NiFe]_Group_1d_Hydro_Hydrogenase_5	GCF_000179055.1	-	-	-	-	-	-	-	-
	GCF_000426585.1	-	-	-	-	-	-	-	-
	GCF_000468635.1	-	-	-	-	-	-	-	-
	GCF_000613565.1	-	-	-	-	-	-	-	-
	GCF_022024235.1	-	-	-	-	-	-	-	-
	GCF_900110745.1	-	-	-	-	-	-	-	-
	GCF_963552095.1	-	-	-	-	-	-	-	-
>[NiFe]_Group_1d_Hydro_Hydrogenase_6	GCF_000179055.1	-	-	-	-	-	-	-	-
	GCF_000426585.1	-	-	-	-	-	-	-	-
	GCF_000468635.1	-	-	-	-	-	-	-	-
	GCF_000613565.1	-	-	-	-	-	-	-	-
	GCF_022024235.1	-	-	-	-	-	-	-	-
	GCF_900110745.1	-	-	-	-	-	-	-	-
	GCF_963552095.1	-	-	-	-	-	-	-	-

>[NiFe]_Group_1d_Hydro_Hydrogenase_7	GCF_000179055.1	-	-	-	-	-	-	-	-
	GCF_000426585.1	-	-	-	-	-	-	-	-
	GCF_000468635.1	-	-	-	-	-	-	-	-
	GCF_000613565.1	-	-	-	-	-	-	-	-
	GCF_022024235.1	-	-	-	-	-	-	-	-
	GCF_900110745.1	-	-	-	-	-	-	-	-
	GCF_963552095.1	-	-	-	-	-	-	-	-
>[NiFe]_Group_1d_Hydro_Hydrogenase_8	GCF_000179055.1	-	-	-	-	-	-	-	-
	GCF_000426585.1	-	-	-	-	-	-	-	-
	GCF_000468635.1	-	-	-	-	-	-	-	-
	GCF_000613565.1	-	-	-	-	-	-	-	-
	GCF_022024235.1	-	-	-	-	-	-	-	-
	GCF_900110745.1	-	-	-	-	-	-	-	-
	GCF_963552095.1	-	-	-	-	-	-	-	-
>[NiFe]_Group_1d_Hydro_Hydrogenase_9	GCF_000179055.1	-	-	-	-	-	-	-	-
	GCF_000426585.1	-	-	-	-	-	-	-	-
	GCF_000468635.1	-	-	-	-	-	-	-	-
	GCF_000613565.1	-	-	-	-	-	-	-	-
	GCF_022024235.1	-	-	-	-	-	-	-	-
	GCF_900110745.1	-	-	-	-	-	-	-	-
	GCF_963552095.1	-	-	-	-	-	-	-	-
>[NiFe]_Group_1d_Hydro_Hydrogenase_10	GCF_000179055.1	-	-	-	-	-	-	-	-
	GCF_000426585.1	-	-	-	-	-	-	-	-
	GCF_000468635.1	-	-	-	-	-	-	-	-
	GCF_000613565.1	-	-	-	-	-	-	-	-
	GCF_022024235.1	-	-	-	-	-	-	-	-
	GCF_900110745.1	-	-	-	-	-	-	-	-
	GCF_963552095.1	-	-	-	-	-	-	-	-

*>[NiFe]_Group_1d_Hydro_Hydrogenase_1

MAKKIVVDPIPRIEGHLRIEATLNDSNMIEEAYSSGTMWRGIEVILKGRDPRDAWAFAERICGVCTTVHALASVRCVEDALGIAVPTNARIIRNLMNATQVTQDHLVHFYHLHALDWVDVVSALKAD

PKQASVIAQSISPWPKSSPGYFKELQQRLVGFVESGQLGIFSNGYWGHPAYKLPPEVNLIAVAHYLEALDFQKEIVKIHTIFGGKNPHPNYLVGGMACAIDPNSDTAINIERLSMIKKIIDETQTFIDQVYI

PDLLAIAGYYKDSLYGGGLGNYLSYGDFPETTLEDFKTLLWPRGAILNKDLTTVIDVDPRDASQVTEEVSHSWYTYTKGDAKGLHPWQGETTPKYTGPKPPFEHLDTDKKYSWLKTPRWKNHPME

VGPLARVLIAYAKGDPMIKETVGLVLSTLGVGPEALFSTLGRTAARGIECKQTAGFMRHYYDQLVANIKTGDLRTFNSERWDPSTWPEECKGFGYTEAPRGSLGHWIHIKDQKIKEYQIVVPSTWNAS

PRDANGNSGAYESALKGTPMVNPEQPLEILRTVHSFDPCLACASHLFDMNGKEITSVTIV"

>[NiFe]_Group_1d_Hydro_Hydrogenase_2

MARIVVDPVTRIEGHLRIELIVDEKTGKVVDAVSSGTMWRGIELILRGRDPRDAWAFAQRICGVCTSIHAQASVRCIEDALEIQIPKNTNYIRNIMYGSLQVHDHLVHFYHLHALDWVSPVEALKADP

VQTAVLQNKILEKYGAIADLLPDPVGHRAYPRKFPKATPGYFRTFQEKIKKLVESGQLGIFAAHWWDHPDYKLLPPEVHLMAVAHYLNMLDVQREFFIPQVVFGGKNPHPHYIVGGMTCSISLDDM

NAPVNAERLAVVEDAIYTQVEAVNLFYLTDLLAIGHIYVQKGQVYGGGLAKRRVIGYGEFPDEPYKGIKTGDYHNKILYHSNGVVENFVEGVEKAKYYPLVGKDFADPEVIQEFVAHSWYKYPDEK

KGLHPWDGITEPNYTGPKEGTKTHWKYLDEQGKYSWIKSPRWRGKPCEVGPLARYIVVYTAVKQGHIKPSWMDEMVVKQIDFVSKVLDIPPHVWLTTTVGRTAARGLEAQLGAAANLYFLKKLY

DNIKAGDTSVANMEKWDPSTWPKEAKGVGITEAPRGALGHWVIIKDGKIANYQCVVPTTWNGSPRDPMGGQGAFEECMKDTPLKVVDKPLEVLRGIHSFDPCLACSTHIYNAKGEEITNIKVQGS

VPVCFTGG"

>[NiFe]_Group_1d_Hydro_Hydrogenase_3

MKHVVVDPITRIEGHLRAEVTVDESGKVTDAVSSGTAWRGLELVMHNRDPRDAWAYIQRICGVCTTAHALASVRAVEDALQIRIPKNANYIRNIMAATLTVQDHLVHFYHLHALDWVSPVEALAAD

TIATANLQNTVLNTYKLPFRTPNASVTEAYEHDFPAATPQYFAEIQGKVKAIVESGQLGIFAANWWDHPDYKLLPPEVHLMAVAHYLEMLDKQRELVTPHVIFGGKNPHPHYVVGGMPCSISMTDG

NAPVNAARLSIVDRAINMGRTLVNNYYLPDLLAIGTAYVKAGRVDGGGLADERVLAYGSYPLEPNKGTTNGDFFNNLLVRCNGVVENFKGGAKSAKFTEIAAEDITDPAVFTEGVEHSWYTYPDEQ

QDADLHPWSGVTNAQYTGPKVGTATEWKELNEQGKYSWLKTPKWRGKLCEVGPLARYIIIYTKAQQGLLTAPTWAEQMMLDQIDAVSKVLGLAPEVWLPTMVGRTAARGLDCQLNAEINKFFFD

KLIANVKSGDTEVVNNEHWNPDTWPQKCKGVGLYEAPRGGLAHWVTIKDDKVDNYQCVVPTTWNACPRDDKAGHGAYERAMMDTHVAIPDKPLEIVKVIRSFDPCMACATHMFNAKGEKISIV

TTDPTSGTHVEPRK

>[NiFe]_Group_1d_Hydro_Hydrogenase_4

MKHVVVDPITRIEGHLRVEVQVDEGSGKVTDAISSGTAWRGLELVMKDRDPRDAWAYIQRICGVCTSAHALGSLRAVEDALGIKIPKNANYIRNIMAATLTVQDHIVHFYHLHALDWVSPVEALAA

DPVATANLQVAALNAYKLPFSAPGTSVTEAYDHAFPAATPQYFAEIKGKVQAIVESGQLGIFSANWWDHPDYKLLPPEVHLMAIAHYLEMLDKHRDLVTPQVIFGGKNPHPHYCVGGMPCSISLEDG

NAPVNTARLGIVDRAINLGRTLVNNFYIPDVLAIGAAYVKAGRVDGGGLAKERVLGYGSYPIEPFSGTSNGDFFKNLLIRTNGVVEDFGKGIDAAKVYEIDAEDITQPEVFTEGVEHSWYEYPANSDH

DLNPWDGVTAPKFTAPKEGTPTDWKALNEQGKYSWLKTPKWRGKLCEVGPLARYIIIYTKAKKGMLGEPTWAEKMMLDQIDAVSKVLNLAPEVWLPTMVGRTAARGLDCQLNAEINKFFFDKLI

ANIKSGDTKTANMEKWDPSTWPKTAKGVGLYEAPRGALSHYIVIKDGKTDNYQAVVPTTWNACPRDDEAGHGAYEKAMMDTQVKIADKPLEIVKVVRSFDPCMACATHMFNAKGEKINVITTDP

YSGTHVEK

>[NiFe]_Group_1d_Hydro_Hydrogenase_5

MKHVVVDPITRIEGHLRVEVTVDEGSGKVTDALSSGTAWRGLELVMKDRDPRDAWAYIQRICGVCTTAHALGAVRAVEDALGIRIPRNANYIRNIMAATLTVQDHIVHFYHLHALDWVSPVDALAA

DPVATANLQNNLLATYKLPFRAPNTYNSEAYEHDFPAATPQYFQQIKDKVKSIVDSGQLGIFAAQWWDHPDYKILPPEVHLLAVAHYLEMLDKQRELVTPHVIFGGKNPHPHYVVGGMPCSISMNDG

NAPVNAARLAVVDRAINLGRTLVNNYYLPDLLAIGNIYVKAGRVDGGGLAKTRVMSFGCYPMESYTGTSSGGYFEGLLVRSNGVVENFGAGVANAKFSEIVAEDLTAPETITEGVEHAWYEYPDAQ

GKDLHPWEGVTKAKYTGPKEGTGTEWKTLDENGKYSWLKTPKWKGKVCEVGPLAKYIVIYTKAKQGLLGELTWAEQMMVDQIEAVSKVLGLAPEVWLPTMVGRTACRGLDCQVAAEINKFFFD

KLIANLKAGDTQVANMEKWDPASWPKECKGVGLYEAPRGALSHWIHIKDGKTENYQCLVPTTWNSCPRDSKAGQGAYELAMMDTHVAIPDKPLEIAKVIRSFDPCMACATHMYNTKGERIAAITT

DPYSGTKVEN"

>[NiFe]_Group_1d_Hydro_Hydrogenase_6

MKRVVVDPVTRIEGHLRVEVSVDEATGQVQDALSSGTAWRGIELVAKDRDPRDVWAFVQRICGVCTSTHALASLRSVEDALGIQIPKNANYIRNIMHSCLDVHDHIVHFYHLHALDWVSPLEALQA

DPAKTAALQETVLKTYNISGLEPADVQSKTSAYPKEFPKGNATYYAAVQAKIKKIVDSGQLGIFAAQWWDHPDYKLLPPEVHLMGVAHYLAVLDRQRDIVTPHVVFGGKNPHPHYIVGGMPCSISM

DDMNAPINTARLAVVDESISMAHDLVDYYYLPDVLAIGHMYVKAGMVDGGGLSKKRVLAYGGYPDEPYSGISNGDYFKKAMVRANGVVENFEQGVDKAVFTNLEGKDLMDPQFLTEEVEHSWY

NYPDGTKSLHPSEGITKPNYTGPKTGTKEHWEFLDETKKYSWIKSPTFQGKACEVGPLAKYIVVYTKVKQGIIKNPTWAEALMVKQVDAVSSVIGVPAHVWMCSTVGRTACRALDAQVAASLSQY

FFDKLVSNIKGGDTVVANTEKFDPNTWPKEAKGVGVLDAPRGGLGHWCVIKDGKIDNYQCIVPTTWNACPKTIANEHGAYESNMIDTKVKIADKPLEILKGIHSFDPCLACSTHLYNKKGEEIVSVN

TDALCK"

>[NiFe]_Group_1d_Hydro_Hydrogenase_7

MSAYETQGFKMDDTGRRIVVDPVTRIEGHMRCEVNVDSNNVIRNAVSTGTMWRGLEVILKGRDPRDAWAFVERICGVCTGCHALASVRAVEDALGIAIPKNAYLIREIMAKTLQEHDHAVHFYHL

HALDWVDVVSALKADPKKTSELQQTVSPSHPMSSPGYFRDIQNRLKKFVESGQLGPFMNGYWGNKAYVLPPEANLMAVTHYLEALDLQKEWVKVHTIFGGKNPHPNYLVGGVPCAINIDKDGAA

GAPVNMERLNFIEARIREMIEFNNNVYVPDVLAIGTIYKQAGWLYGGGLSATNVMDYGTYEKVMGDRRTQQLPGGAILGGNWNEILPVDPRDPEQVQEFVNHSWYKYADESKGLHPWDGVTEPN

YELGAKTKGTRTNIERLDESAKYSWIKSPRWRGHAMEVGPLSRYILGYAHAAKGNPHCQRVKEQVDLSAAVINKVFPKALGMPETQYTVKQLLPTTIGRTLARALEAQYCAEMMLDDFQELVANI

KAGDTSTANVEKWDPATWPKEAKGVGTVAAPRGALGHWIRIKDGRIENYQCVVPTTWNGSPRDTKGQIGAFEASLMNTPMADPEQPVEILRTLHSFDPCLACSTHVMSPDGQELTSVTVR"

>[NiFe]_Group_1d_Hydro_Hydrogenase_8

MSIQTPNGFTLDNSGTRIVVDPVTRIEGHLRCEVNLDENNVIRNAVSSGTMWRGLEVILKGRDPRDAWAFTERICGVCTGTHALTSVRAVEDALGIQIPENANSIRNIMQLTLQSHDHLVHFYHLHAL

DWVDVVSALKADPKATSTLAQSISAWPQSSPGWFRDVQNRLKRFVESGQLGPFMNGYWGNPAYKLPPEANLMAVAHYLEALDFQREIVKIHTVYGGKNPHPNWLVGGVPCAINVDGTGAVGAIN

MERLNLVSSIIDRTIDFIDQVYIPDLLAIASFYKDWTFGGGLSSQSVMSYGDIPERANDNSAANQLLPRGAVINGNLNEVHEVNLRDPEQIQEFVAHSWYSYKDENKGLHPWDGVTEPNFVLGPNTKG

TRTNIQEIDEGAKYSWIKAPRWRGHAMEVGPLARWVIGYAQGTPEFKEPVEKVLTDLNLPVAALFSTLGRTAARGLEASWAVHKLRYFQDKLVANIKAGDSATANITKWDPDTWPREARGVGTTEA

PRGALGHWIVIKNGRIENYQAVVPTTWNGSPRDPAGNIGAFEAALLNTPVAVADQPLEILRTLHSFDPCLACSTHVMSPDGQELTRVKVR

>[NiFe]_Group_1d_Hydro_Hydrogenase_9

MSVTTANGFELDTAGRRLVVDPVTRIEGHLRCEVNLDENNVIRNAVSTGTMWRGLEVILRGRDPRDAWAFTERICGVCTGTHALTSVRAVEDALGIPIPENANSIRNIMHVTLQAHDHLVHFYHLHA

LDWVDVVSALGADPKATSALAQSISDWPKSSPGYFRDVQNRLKRFVESGQLGPFMNGYWGSPAYKLPPEANLMAVTHYLEALDFQKEIVKIHTVYGGKNPHPNWLVGGMPCAINVDGTGAVGAI

NMERLNLVSSIIDQTIAFIDKVYIPDLIAIASFYKDWTYGGGLSSQAVMSYGDIPDHANDMSSKNLLLPRGAIINGNLNEIHEIDLRNPEEIQEFVDHSWFSYKDETRGLHPWDGVTEPNFVLGPNAVGS

RTRIEALDEQAKYSWIKAPRWRGHAMEVGPLARYVIGYAKGIPEFKEPVDKVLTDLGQPLEAIFSTLGRTAARGLEASWAAHKMRYFQDKLVANIRAGDTATANVDNWDPKTWPKEARGVGTTEA

PRGALGHWIVIKDGKIDNYQAVVPTTWNGSPRDPAGNIGAFEASLLNTPLAKADEPLEILRTLHSFDPCLACATHIMGPDGEELTRIKVR

>[NiFe]_Group_1d_Hydro_Hydrogenase_10

MTIQTPNGFALDNSGKRIVVDPVTRIEGHMRVEVNVDENNIIRNAVSTGTMWRGIEVILKNRDPRDAWAFTERICGVCTGTHALTSVRAVENALGILIPENANSIRNLMQLALQVHDHVVHFYHLHA

LDWVDVVSALSADPRGTSALAQSISDWPLSSPGYFKDIQTRLKKFVESGQLGPFKNGYWGSKAYRLPPEANLMAVAHYLEALDFQKEIVKIHTIFGGKNPHPNWLVGGVPCPINVDGTGAVGAINM

ERLNHVSSIIDQLIEFNAKVYVPDVKAIGSFYKDWLYGGGLSSKCVMSYGDIPEHANDYSEASLKLPRGAIINGNLAEVFPIDLEDPNEIQEFVAHSWYKYPDETKGLHPWDGVTEPNYVLGPNAKG

TRTNIEELDEAGKYSWIKSPRWKGNAMEVGPLARWIIGYAQNKAEFKEPVEKLLKDLDLPVTALFSTLGRTAARALESDWAGLQMRHFFDKLVANIKAGDSATAFVDKWKPETWPAEVKGVGFTE

APRGALAHWVKIKDGKIDNYQCVVPTTWNGSPRDPTGNIGAFEAALMDTPMADPAQPLEILRTLHSFDPCLACSTHVMSPDGQEMAKVQVR

Table S7. Metagenome-wide analysis between structural variants of rumen microbes and methane emissions

type	sv	group	Estimate	Std. Error	FDR
vsgv	X1410608.PRJNA223492.101_102	B.Ga6A2	-0.80	0.887070934	0.53
vsgv	X1410608.PRJNA223492.1058_1059	B.Ga6A2	1.30	0.019735662	0.01
vsgv	X1410608.PRJNA223492.1094_1095	B.Ga6A2	2.39	0.443185845	0.12
vsgv	X1410608.PRJNA223492.1179_1180	B.Ga6A2	-2.29	2.062080666	0.47
vsgv	X1410608.PRJNA223492.118_119	B.Ga6A2	-0.95	0.360331461	0.23
vsgv	X1410608.PRJNA223492.124_125	B.Ga6A2	-0.60	1.577546116	0.77
dsgv	X1410608.PRJNA223492.127_128	B.Ga6A2	-8.14	1.856255279	0.14
dsgv	X1410608.PRJNA223492.13_14	B.Ga6A2	-3.64	17.97115506	0.87
dsgv	X1410608.PRJNA223492.131_132	B.Ga6A2	4.05	7.655336402	0.69
dsgv	X1410608.PRJNA223492.14_15	B.Ga6A2	-10.21	11.50147297	0.54
dsgv	X1410608.PRJNA223492.144_145	B.Ga6A2	-4.90	2.163971883	0.26
vsgv	X1410608.PRJNA223492.1489_1490	B.Ga6A2	-3.16	0.654734319	0.13
vsgv	X1410608.PRJNA223492.1551_1552	B.Ga6A2	-3.13	2.708253939	0.45
vsgv	X1410608.PRJNA223492.1796_1797	B.Ga6A2	1.47	1.359698338	0.48
vsgv	X1410608.PRJNA223492.1843_1844	B.Ga6A2	0.71	1.191831837	0.66
vsgv	X1410608.PRJNA223492.2052_2053	B.Ga6A2	2.75	0.996694889	0.22
vsgv	X1410608.PRJNA223492.2061_2062	B.Ga6A2	1.01	0.854628311	0.45
vsgv	X1410608.PRJNA223492.210_211	B.Ga6A2	4.40	0.545418292	0.08
dsgv	X1410608.PRJNA223492.216_217	B.Ga6A2	2.28	5.47845855	0.75
dsgv	X1410608.PRJNA223492.2223_2225	B.Ga6A2	6.14	0.842967097	0.09
vsgv	X1410608.PRJNA223492.2348_2349	B.Ga6A2	-1.51	1.04502417	0.39
dsgv	X1410608.PRJNA223492.24_25	B.Ga6A2	-2.28	5.47845855	0.75
vsgv	X1410608.PRJNA223492.241_242	B.Ga6A2	4.07	0.150074188	0.02
vsgv	X1410608.PRJNA223492.245_246	B.Ga6A2	-0.02	1.733766002	0.99
vsgv	X1410608.PRJNA223492.2552_2553	B.Ga6A2	2.04	0.875910653	0.26
dsgv	X1410608.PRJNA223492.26_27	B.Ga6A2	-8.14	1.856255279	0.14
vsgv	X1410608.PRJNA223492.264_265	B.Ga6A2	-1.25	0.462149534	0.23
dsgv	X1410608.PRJNA223492.27_28	B.Ga6A2	-3.06	6.274939668	0.71
dsgv	X1410608.PRJNA223492.3079_3080	B.Ga6A2	1.45	5.076017577	0.82
vsgv	X1410608.PRJNA223492.319_320	B.Ga6A2	0.21	1.793161418	0.93
dsgv	X1410608.PRJNA223492.3259_3261	B.Ga6A2	-3.06	6.274939668	0.71
dsgv	X1410608.PRJNA223492.3273_3275	B.Ga6A2	4.05	7.655336402	0.69
vsgv	X1410608.PRJNA223492.333_334	B.Ga6A2	0.81	2.053511865	0.76
vsgv	X1410608.PRJNA223492.345_346	B.Ga6A2	-0.10	1.788456205	0.97
vsgv	X1410608.PRJNA223492.3528_3529	B.Ga6A2	-0.26	1.738343865	0.90
dsgv	X1410608.PRJNA223492.378_380	B.Ga6A2	4.05	7.655336402	0.69
dsgv	X1410608.PRJNA223492.408_409	B.Ga6A2	8.14	1.856255279	0.14
dsgv	X1410608.PRJNA223492.443_444	B.Ga6A2	5.27	6.528927499	0.50
dsgv	X1410608.PRJNA223492.49_50	B.Ga6A2	4.05	7.655336402	0.69
vsgv	X1410608.PRJNA223492.551_552	B.Ga6A2	0.81	1.798740654	0.73
dsgv	X1410608.PRJNA223492.57_58	B.Ga6A2	-6.14	0.842967097	0.09
dsgv	X1410608.PRJNA223492.583_585	B.Ga6A2	-4.05	7.655336402	0.69

dsgv	X1410608.PRJNA223492.60_61	B.Ga6A2	3.64	17.97115506	0.87
dsgv	X1410608.PRJNA223492.64_66	B.Ga6A2	-8.14	1.856255279	0.14
dsgv	X1410608.PRJNA223492.71_72	B.Ga6A2	-6.01	4.538847691	0.41
dsgv	X1410608.PRJNA223492.713_714	B.Ga6A2	10.21	11.50147297	0.54
dsgv	X1410608.PRJNA223492.78_79	B.Ga6A2	-6.01	4.538847691	0.41
vsgv	X1410608.PRJNA223492.811_812	B.Ga6A2	2.19	1.061373082	0.29
dsgv	X1410608.PRJNA223492.9_11	B.Ga6A2	-1.45	5.076017577	0.82
vsgv	X1410608.PRJNA223492.959_960	B.Ga6A2	-0.49	3.432570879	0.91
dsgv	X1410658.PRJNA223501.11_13	K.vitulina_MC3001	4.13	3.320275795	0.30
dsgv	X1410658.PRJNA223501.1143_1144	K.vitulina_MC3001	2.00	4.535680643	0.69
dsgv	X1410658.PRJNA223501.1175_1176	K.vitulina_MC3001	-1.97	2.70397865	0.52
dsgv	X1410658.PRJNA223501.13_14	K.vitulina_MC3001	2.00	4.535680643	0.69
vsgv	X1410658.PRJNA223501.1304_1305	K.vitulina_MC3001	0.17	0.40915957	0.70
vsgv	X1410658.PRJNA223501.1464_1465	K.vitulina_MC3001	0.22	0.547166457	0.71
vsgv	X1410658.PRJNA223501.1581_1582	K.vitulina_MC3001	-0.03	0.188077429	0.90
vsgv	X1410658.PRJNA223501.1833_1834	K.vitulina_MC3001	0.44	0.257281043	0.19
dsgv	X1410658.PRJNA223501.19_20	K.vitulina_MC3001	3.14	2.644716703	0.32
dsgv	X1410658.PRJNA223501.24_25	K.vitulina_MC3001	-0.35	3.403562625	0.92
dsgv	X1410658.PRJNA223501.276_277	K.vitulina_MC3001	4.90	5.25602685	0.42
dsgv	X1410658.PRJNA223501.288_290	K.vitulina_MC3001	-0.42	3.008603562	0.90
dsgv	X1410658.PRJNA223501.293_294	K.vitulina_MC3001	0.02	2.908996432	0.99
dsgv	X1410658.PRJNA223501.378_379	K.vitulina_MC3001	-0.76	3.591751691	0.85
dsgv	X1410658.PRJNA223501.393_394	K.vitulina_MC3001	0.10	2.879585901	0.97
vsgv	X1410658.PRJNA223501.398_399	K.vitulina_MC3001	0.45	0.49419112	0.43
dsgv	X1410658.PRJNA223501.4_5	K.vitulina_MC3001	-0.42	3.008603562	0.90
dsgv	X1410658.PRJNA223501.400_401	K.vitulina_MC3001	3.89	1.812227579	0.12
dsgv	X1410658.PRJNA223501.434_435	K.vitulina_MC3001	4.74	1.963652853	0.09
vsgv	X1410658.PRJNA223501.588_589	K.vitulina_MC3001	-0.60	0.650147598	0.43
dsgv	X1410658.PRJNA223501.723_724	K.vitulina_MC3001	1.66	2.723915558	0.59
dsgv	X1410658.PRJNA223501.76_78	K.vitulina_MC3001	-16.74	10.84556989	0.22
dsgv	X1410658.PRJNA223501.78_79	K.vitulina_MC3001	0.91	2.678672866	0.76
vsgv	X1410658.PRJNA223501.793_796	K.vitulina_MC3001	0.01	0.010180466	0.36
vsgv	X1410658.PRJNA223501.914_915	K.vitulina_MC3001	0.31	0.313141374	0.39
dsgv	X1410658.PRJNA223501.942_943	K.vitulina_MC3001	-4.28	2.645876364	0.20
dsgv	X1410658.PRJNA223501.943_944	K.vitulina_MC3001	3.26	3.944365073	0.47
dsgv	X1410658.PRJNA223501.945_946	K.vitulina_MC3001	-1.89	4.008748372	0.67
dsgv	X1410674.PRJNA223483.1007_1008	S.azabuensis_DSM_18934	3.79	2.578112738	0.22
dsgv	X1410674.PRJNA223483.1064_1066	S.azabuensis_DSM_18934	-2.23	3.887154318	0.60
dsgv	X1410674.PRJNA223483.1068_1069	S.azabuensis_DSM_18934	1.61	3.669652937	0.68
dsgv	X1410674.PRJNA223483.114_115	S.azabuensis_DSM_18934	-3.62	4.368804268	0.45
dsgv	X1410674.PRJNA223483.12_13	S.azabuensis_DSM_18934	3.34	3.633651612	0.41
dsgv	X1410674.PRJNA223483.1220_1221	S.azabuensis_DSM_18934	0.75	3.915239674	0.86
dsgv	X1410674.PRJNA223483.1225_1226	S.azabuensis_DSM_18934	4.98	3.046119454	0.18
vsgv	X1410674.PRJNA223483.1239_1240	S.azabuensis_DSM_18934	-0.68	0.502304634	0.25

dsgv	X1410674.PRJNA223483.13_14	S.azabuensis_DSM_18934	-3.11	3.523816273	0.43
dsgv	X1410674.PRJNA223483.137_138	S.azabuensis_DSM_18934	3.72	2.998763943	0.28
dsgv	X1410674.PRJNA223483.138_139	S.azabuensis_DSM_18934	6.34	2.870376838	0.09
vsgv	X1410674.PRJNA223483.1387_1388	S.azabuensis_DSM_18934	0.02	0.917301733	0.98
dsgv	X1410674.PRJNA223483.139_140	S.azabuensis_DSM_18934	2.15	6.659120095	0.76
dsgv	X1410674.PRJNA223483.14_15	S.azabuensis_DSM_18934	3.49	3.690595976	0.40
dsgv	X1410674.PRJNA223483.140_141	S.azabuensis_DSM_18934	6.94	2.359313328	0.04
dsgv	X1410674.PRJNA223483.1638_1640	S.azabuensis_DSM_18934	2.19	5.459063292	0.71
vsgv	X1410674.PRJNA223483.1775_1776	S.azabuensis_DSM_18934	0.37	1.051741739	0.74
vsgv	X1410674.PRJNA223483.1807_1812	S.azabuensis_DSM_18934	0.01	0.488537163	0.99
vsgv	X1410674.PRJNA223483.1871_1872	S.azabuensis_DSM_18934	0.37	0.394413011	0.40
vsgv	X1410674.PRJNA223483.194_196	S.azabuensis_DSM_18934	1.58	1.603352063	0.38
dsgv	X1410674.PRJNA223483.2006_2007	S.azabuensis_DSM_18934	0.42	4.19231481	0.92
dsgv	X1410674.PRJNA223483.230_	S.azabuensis_DSM_18934	0.00	3.683671034	1.00
vsgv	X1410674.PRJNA223483.279_280	S.azabuensis_DSM_18934	-0.17	1.138460136	0.89
dsgv	X1410674.PRJNA223483.360_361	S.azabuensis_DSM_18934	-1.90	3.599479181	0.62
dsgv	X1410674.PRJNA223483.365_366	S.azabuensis_DSM_18934	-3.38	3.929601456	0.44
dsgv	X1410674.PRJNA223483.460_461	S.azabuensis_DSM_18934	-4.68	14.13520297	0.76
vsgv	X1410674.PRJNA223483.472_473	S.azabuensis_DSM_18934	-0.95	1.02813259	0.41
dsgv	X1410674.PRJNA223483.523_524	S.azabuensis_DSM_18934	-2.63	2.917314472	0.42
vsgv	X1410674.PRJNA223483.56_57	S.azabuensis_DSM_18934	2.44	2.631154856	0.41
dsgv	X1410674.PRJNA223483.603_604	S.azabuensis_DSM_18934	-1.63	3.369999553	0.65
dsgv	X1410674.PRJNA223483.607_609	S.azabuensis_DSM_18934	2.19	5.459063292	0.71
dsgv	X1410674.PRJNA223483.613_615	S.azabuensis_DSM_18934	-1.63	3.369999553	0.65
dsgv	X1410674.PRJNA223483.615_616	S.azabuensis_DSM_18934	2.63	2.917314472	0.42
dsgv	X1410674.PRJNA223483.619_620	S.azabuensis_DSM_18934	5.89	2.332171959	0.06
dsgv	X1410674.PRJNA223483.622_624	S.azabuensis_DSM_18934	1.98	4.453463084	0.68
dsgv	X1410674.PRJNA223483.624_625	S.azabuensis_DSM_18934	-0.92	3.1782532	0.79
dsgv	X1410674.PRJNA223483.626_627	S.azabuensis_DSM_18934	-4.26	4.674460284	0.41
dsgv	X1410674.PRJNA223483.629_630	S.azabuensis_DSM_18934	3.49	3.690595976	0.40
dsgv	X1410674.PRJNA223483.638_639	S.azabuensis_DSM_18934	-5.55	4.717977814	0.31
dsgv	X1410674.PRJNA223483.639_640	S.azabuensis_DSM_18934	-12.78	5.814630886	0.08
vsgv	X1410674.PRJNA223483.64_65	S.azabuensis_DSM_18934	0.96	0.853617799	0.32
dsgv	X1410674.PRJNA223483.640_642	S.azabuensis_DSM_18934	-1.97	3.666819243	0.62
vsgv	X1410674.PRJNA223483.679_680	S.azabuensis_DSM_18934	-0.05	0.914379023	0.96
dsgv	X1410674.PRJNA223483.7_8	S.azabuensis_DSM_18934	1.96	6.325861634	0.77
vsgv	X1410674.PRJNA223483.730_733	S.azabuensis_DSM_18934	-0.03	0.112043599	0.81
vsgv	X1410674.PRJNA223483.733_734	S.azabuensis_DSM_18934	0.07	0.249488852	0.80
vsgv	X1410674.PRJNA223483.734_735	S.azabuensis_DSM_18934	0.05	0.580894701	0.93
vsgv	X1410674.PRJNA223483.735_736	S.azabuensis_DSM_18934	-0.79	0.447259288	0.15
vsgv	X1410674.PRJNA223483.947_948	S.azabuensis_DSM_18934	0.72	1.235771239	0.59
dsgv	X1690.PRJNA239856.1029_1031	B.pseudolongum	-1.86	2.677905793	0.50
dsgv	X1690.PRJNA239856.1032_1033	B.pseudolongum	0.10	2.600990664	0.97
dsgv	X1690.PRJNA239856.1175_1176	B.pseudolongum	-1.81	4.167444189	0.67

vsgv	X1690.PRJNA239856.1232_1234	B.pseudolongum	-0.45	0.63083409	0.49
vsgv	X1690.PRJNA239856.1266_1269	B.pseudolongum	-0.24	0.270944659	0.40
vsgv	X1690.PRJNA239856.134_135	B.pseudolongum	0.16	0.68520472	0.82
dsgv	X1690.PRJNA239856.150_151	B.pseudolongum	-2.53	2.473127361	0.32
dsgv	X1690.PRJNA239856.151_152	B.pseudolongum	-5.30	2.097717662	0.02
dsgv	X1690.PRJNA239856.152_153	B.pseudolongum	-2.22	2.508027873	0.39
dsgv	X1690.PRJNA239856.155_156	B.pseudolongum	1.69	2.444235886	0.50
dsgv	X1690.PRJNA239856.156_159	B.pseudolongum	-0.25	2.551223745	0.92
dsgv	X1690.PRJNA239856.1578_1579	B.pseudolongum	1.16	2.927405419	0.70
dsgv	X1690.PRJNA239856.159_160	B.pseudolongum	1.52	2.886172955	0.61
dsgv	X1690.PRJNA239856.164_165	B.pseudolongum	1.34	3.037516882	0.67
dsgv	X1690.PRJNA239856.1662_1663	B.pseudolongum	3.97	3.090082237	0.22
dsgv	X1690.PRJNA239856.1676_1678	B.pseudolongum	0.36	2.671695172	0.89
dsgv	X1690.PRJNA239856.1685_1686	B.pseudolongum	-2.09	2.488016486	0.41
vsgv	X1690.PRJNA239856.1689_1690	B.pseudolongum	-0.17	0.65815474	0.80
vsgv	X1690.PRJNA239856.1690_1692	B.pseudolongum	-0.40	0.243376689	0.12
vsgv	X1690.PRJNA239856.1692_1693	B.pseudolongum	-0.22	0.214916225	0.33
vsgv	X1690.PRJNA239856.1693_1695	B.pseudolongum	-0.34	0.200333661	0.12
vsgv	X1690.PRJNA239856.1774_1775	B.pseudolongum	-0.29	0.521855765	0.59
dsgv	X1690.PRJNA239856.1854_1855	B.pseudolongum	0.91	2.71122497	0.74
dsgv	X1690.PRJNA239856.1886_1887	B.pseudolongum	2.24	2.61794675	0.41
dsgv	X1690.PRJNA239856.1889_1890	B.pseudolongum	1.99	2.927582633	0.51
vsgv	X1690.PRJNA239856.1930_1935	B.pseudolongum	-0.05	0.037799108	0.20
dsgv	X1690.PRJNA239856.233_234	B.pseudolongum	4.13	2.416438553	0.11
dsgv	X1690.PRJNA239856.334_335	B.pseudolongum	-1.14	3.204829424	0.73
dsgv	X1690.PRJNA239856.336_337	B.pseudolongum	-4.13	2.768029659	0.16
dsgv	X1690.PRJNA239856.345_346	B.pseudolongum	-0.76	2.410876774	0.76
dsgv	X1690.PRJNA239856.354_355	B.pseudolongum	3.46	2.774040853	0.23
vsgv	X1690.PRJNA239856.399_412	B.pseudolongum	0.43	0.367688662	0.27
dsgv	X1690.PRJNA239856.408_409	B.pseudolongum	1.65	2.700950515	0.55
dsgv	X1690.PRJNA239856.425_426	B.pseudolongum	0.57	2.437554622	0.82
dsgv	X1690.PRJNA239856.55_56	B.pseudolongum	-0.49	2.732129446	0.86
dsgv	X1690.PRJNA239856.56_57	B.pseudolongum	4.17	2.186999406	0.08
dsgv	X1690.PRJNA239856.57_59	B.pseudolongum	0.69	2.562557624	0.79
dsgv	X1690.PRJNA239856.59_60	B.pseudolongum	-0.36	3.047285668	0.91
dsgv	X1690.PRJNA239856.60_61	B.pseudolongum	-0.08	2.589634881	0.98
dsgv	X1690.PRJNA239856.63_64	B.pseudolongum	0.30	2.689044772	0.91
dsgv	X1690.PRJNA239856.660_661	B.pseudolongum	-0.54	2.487794273	0.83
dsgv	X1690.PRJNA239856.775_776	B.pseudolongum	1.33	2.518777905	0.61
dsgv	X1690.PRJNA239856.778_779	B.pseudolongum	0.26	2.749286086	0.93
dsgv	X1690.PRJNA239856.780_781	B.pseudolongum	-2.67	2.512857519	0.31
dsgv	X1690.PRJNA239856.781_782	B.pseudolongum	-0.55	2.421785208	0.82
dsgv	X1690.PRJNA239856.782_783	B.pseudolongum	-1.01	3.279514642	0.76
vsgv	X220668.PRJNA356.1065_1066	L.plantarum_WCFS1	1.21	0.318478747	0.16

dsgv	X220668.PRJNA356.1175_1176	L.plantarum_WCFS1	-2.18	8.036419735	0.83
dsgv	X220668.PRJNA356.1202_1203	L.plantarum_WCFS1	2.18	8.036419735	0.83
dsgv	X220668.PRJNA356.1204_1205	L.plantarum_WCFS1	-4.77	3.448182086	0.40
vsgv	X220668.PRJNA356.138_139	L.plantarum_WCFS1	-0.17	0.651460126	0.84
vsgv	X220668.PRJNA356.1560_1561	L.plantarum_WCFS1	0.93	0.419256188	0.27
dsgv	X220668.PRJNA356.1814_1815	L.plantarum_WCFS1	2.18	8.036419735	0.83
vsgv	X220668.PRJNA356.1834_1835	L.plantarum_WCFS1	0.90	0.626539079	0.39
vsgv	X220668.PRJNA356.190_191	L.plantarum_WCFS1	-0.35	1.388075697	0.84
dsgv	X220668.PRJNA356.199_200	L.plantarum_WCFS1	-2.18	8.036419735	0.83
dsgv	X220668.PRJNA356.204_205	L.plantarum_WCFS1	4.77	3.448182086	0.40
dsgv	X220668.PRJNA356.2163_2164	L.plantarum_WCFS1	-5.86	0.570027781	0.06
vsgv	X220668.PRJNA356.2333_2334	L.plantarum_WCFS1	1.60	0.182914052	0.07
vsgv	X220668.PRJNA356.24_25	L.plantarum_WCFS1	-1.01	0.85058607	0.45
vsgv	X220668.PRJNA356.266_26	L.plantarum_WCFS1	0.40	1.549806862	0.84
vsgv	X220668.PRJNA356.2796_2797	L.plantarum_WCFS1	1.32	0.478932891	0.22
vsgv	X220668.PRJNA356.2996_2997	L.plantarum_WCFS1	-1.07	3.044843221	0.78
dsgv	X220668.PRJNA356.331_332	L.plantarum_WCFS1	2.18	8.036419735	0.83
dsgv	X220668.PRJNA356.333_334	L.plantarum_WCFS1	4.77	3.448182086	0.40
dsgv	X220668.PRJNA356.346_347	L.plantarum_WCFS1	5.86	0.570027781	0.06
dsgv	X220668.PRJNA356.348_349	L.plantarum_WCFS1	3.47	2.294118824	0.37
dsgv	X220668.PRJNA356.399_400	L.plantarum_WCFS1	-2.18	8.036419735	0.83
vsgv	X220668.PRJNA356.43_44	L.plantarum_WCFS1	0.59	0.135691514	0.14
dsgv	X220668.PRJNA356.455_456	L.plantarum_WCFS1	-5.86	0.570027781	0.06
vsgv	X220668.PRJNA356.484_485	L.plantarum_WCFS1	-0.26	0.667483428	0.76
dsgv	X220668.PRJNA356.595_596	L.plantarum_WCFS1	-2.18	8.036419735	0.83
vsgv	X220668.PRJNA356.904_906	L.plantarum_WCFS1	2.22	1.562607051	0.39
vsgv	X220668.PRJNA356.924_925	L.plantarum_WCFS1	0.81	0.2745913	0.21
dsgv	X278197.PRJNA398.128_129	P.pentosaceus_ATCC_25745	-2.16	1.811970991	0.32
vsgv	X278197.PRJNA398.1395_1396	P.pentosaceus_ATCC_25745	-1.65	0.507545617	0.05
vsgv	X278197.PRJNA398.1542_1543	P.pentosaceus_ATCC_25745	-0.05	0.513056385	0.92
vsgv	X278197.PRJNA398.1665_1666	P.pentosaceus_ATCC_25745	-0.88	0.711581666	0.30
vsgv	X278197.PRJNA398.1723_1724	P.pentosaceus_ATCC_25745	-0.16	0.572732433	0.79
dsgv	X278197.PRJNA398.186_187	P.pentosaceus_ATCC_25745	0.36	2.521308093	0.90
dsgv	X278197.PRJNA398.205_206	P.pentosaceus_ATCC_25745	-2.25	1.771512943	0.29
dsgv	X278197.PRJNA398.235_236	P.pentosaceus_ATCC_25745	3.01	1.131698984	0.08
dsgv	X278197.PRJNA398.236_238	P.pentosaceus_ATCC_25745	5.21	1.224505689	0.02
vsgv	X278197.PRJNA398.312_313	P.pentosaceus_ATCC_25745	-0.06	0.500277696	0.91
vsgv	X278197.PRJNA398.38_39	P.pentosaceus_ATCC_25745	0.68	0.231496009	0.06
vsgv	X278197.PRJNA398.421_422	P.pentosaceus_ATCC_25745	-0.16	0.507926983	0.78
vsgv	X278197.PRJNA398.449_450	P.pentosaceus_ATCC_25745	-0.24	0.630928935	0.73
vsgv	X278197.PRJNA398.49_50	P.pentosaceus_ATCC_25745	-0.18	0.535810182	0.76
vsgv	X278197.PRJNA398.517_518	P.pentosaceus_ATCC_25745	-0.01	0.491792748	0.98
vsgv	X278197.PRJNA398.558_560	P.pentosaceus_ATCC_25745	0.12	0.280739738	0.69
dsgv	X278197.PRJNA398.56_58	P.pentosaceus_ATCC_25745	-1.22	2.539692884	0.66

dsgv	X278197.PRJNA398.578_579	P.pentosaceus_ATCC_25745	1.25	1.917383055	0.56
dsgv	X278197.PRJNA398.58_60	P.pentosaceus_ATCC_25745	0.13	2.624049182	0.96
dsgv	X278197.PRJNA398.627_628	P.pentosaceus_ATCC_25745	2.53	1.783156765	0.25
vsgv	X278197.PRJNA398.660_661	P.pentosaceus_ATCC_25745	-0.90	0.264339234	0.04
vsgv	X278197.PRJNA398.697_698	P.pentosaceus_ATCC_25745	1.43	0.723509353	0.14
vsgv	X278197.PRJNA398.742_745	P.pentosaceus_ATCC_25745	-0.10	0.629191512	0.88
dsgv	X278197.PRJNA398.75_76	P.pentosaceus_ATCC_25745	-2.58	1.624209863	0.21
dsgv	X278197.PRJNA398.77_78	P.pentosaceus_ATCC_25745	1.47	2.925006629	0.65
dsgv	X278197.PRJNA398.786_787	P.pentosaceus_ATCC_25745	0.26	2.163120882	0.91
dsgv	X278197.PRJNA398.79_80	P.pentosaceus_ATCC_25745	0.14	2.154857215	0.95
dsgv	X278197.PRJNA398.86_87	P.pentosaceus_ATCC_25745	-3.83	2.622523929	0.24
dsgv	X278197.PRJNA398.87_88.	P.pentosaceus_ATCC_25745	1.52	3.063907154	0.65
dsgv	X278197.PRJNA398.88_89	P.pentosaceus_ATCC_25745	1.48	3.393051835	0.69
dsgv	X278197.PRJNA398.89_91	P.pentosaceus_ATCC_25745	0.03	4.742072942	1.00
vsgv	X278197.PRJNA398.915_916	P.pentosaceus_ATCC_25745	0.17	0.673965536	0.81
dsgv	X278197.PRJNA398.92_93	P.pentosaceus_ATCC_25745	2.47	3.157395628	0.49
dsgv	X752555.PRJNA47067.1048_1049	P.bryantii_B14	-0.82	0.568071966	0.15
dsgv	X752555.PRJNA47067.1049_1050	P.bryantii_B14	0.34	0.568628915	0.55
vsgv	X752555.PRJNA47067.1295_1296	P.bryantii_B14	0.02	0.177603269	0.90
dsgv	X752555.PRJNA47067.1297_1298	P.bryantii_B14	-0.50	0.605086285	0.41
dsgv	X752555.PRJNA47067.1298_1299	P.bryantii_B14	0.11	0.591890596	0.85
dsgv	X752555.PRJNA47067.142_143	P.bryantii_B14	-0.02	0.590633172	0.98
dsgv	X752555.PRJNA47067.1425_1426	P.bryantii_B14	-0.58	0.5742052	0.31
dsgv	X752555.PRJNA47067.1485_1486	P.bryantii_B14	-0.41	0.570783736	0.47
dsgv	X752555.PRJNA47067.1486_1487	P.bryantii_B14	-0.26	0.571305567	0.66
dsgv	X752555.PRJNA47067.1492_1493	P.bryantii_B14	-0.22	0.571673066	0.71
dsgv	X752555.PRJNA47067.1497_1498	P.bryantii_B14	-0.95	0.585401047	0.11
dsgv	X752555.PRJNA47067.1500_1501	P.bryantii_B14	-0.61	0.564437018	0.29
dsgv	X752555.PRJNA47067.1501_1502	P.bryantii_B14	-0.17	0.600441473	0.78
dsgv	X752555.PRJNA47067.1502_1503	P.bryantii_B14	0.30	0.618650395	0.62
dsgv	X752555.PRJNA47067.1512_1513	P.bryantii_B14	-0.13	0.580942851	0.82
dsgv	X752555.PRJNA47067.1515_1516	P.bryantii_B14	-0.61	0.597365022	0.31
dsgv	X752555.PRJNA47067.1516_1517	P.bryantii_B14	0.07	0.601822816	0.91
dsgv	X752555.PRJNA47067.1517_1518	P.bryantii_B14	0.04	0.579075341	0.94
dsgv	X752555.PRJNA47067.1518_1519	P.bryantii_B14	-0.01	0.576352115	0.99
dsgv	X752555.PRJNA47067.1523_1524	P.bryantii_B14	0.23	0.583805569	0.70
dsgv	X752555.PRJNA47067.1527_1528	P.bryantii_B14	0.01	0.585987258	0.98
dsgv	X752555.PRJNA47067.1528_1529	P.bryantii_B14	-0.55	0.571445863	0.34
dsgv	X752555.PRJNA47067.1529_1530	P.bryantii_B14	-0.09	0.589947738	0.87
dsgv	X752555.PRJNA47067.1530_1531	P.bryantii_B14	-0.55	0.643172842	0.39
dsgv	X752555.PRJNA47067.1531_1532	P.bryantii_B14	-0.01	0.586708935	0.98
dsgv	X752555.PRJNA47067.1532_1533	P.bryantii_B14	-0.34	0.568079899	0.55
dsgv	X752555.PRJNA47067.1533_1534	P.bryantii_B14	-0.10	0.573381872	0.86
dsgv	X752555.PRJNA47067.1534_1535	P.bryantii_B14	-0.89	0.593948675	0.14

dsgv	X752555.PRJNA47067.1535_1536	P.bryantii_B14	0.79	0.581767373	0.18
vsgv	X752555.PRJNA47067.1538_1540	P.bryantii_B14	0.09	0.113015027	0.44
dsgv	X752555.PRJNA47067.1540_1541	P.bryantii_B14	0.47	0.633487992	0.46
dsgv	X752555.PRJNA47067.1545_1546	P.bryantii_B14	-0.06	0.576822464	0.92
dsgv	X752555.PRJNA47067.1546_1547	P.bryantii_B14	0.04	0.5697201	0.95
dsgv	X752555.PRJNA47067.155_156	P.bryantii_B14	-0.14	0.579770612	0.81
vsgv	X752555.PRJNA47067.1551_1552	P.bryantii_B14	0.00	0.205366286	1.00
vsgv	X752555.PRJNA47067.1552_1553	P.bryantii_B14	0.22	0.128963187	0.10
dsgv	X752555.PRJNA47067.156_157	P.bryantii_B14	-1.31	0.885668134	0.14
dsgv	X752555.PRJNA47067.1565_1566	P.bryantii_B14	0.29	0.636745603	0.65
dsgv	X752555.PRJNA47067.1567_1568	P.bryantii_B14	0.36	0.58435579	0.54
vsgv	X752555.PRJNA47067.1568_1569	P.bryantii_B14	-0.07	0.140227802	0.64
vsgv	X752555.PRJNA47067.1572_1573	P.bryantii_B14	0.11	0.091502824	0.23
vsgv	X752555.PRJNA47067.1573_1574	P.bryantii_B14	0.03	0.055249418	0.56
dsgv	X752555.PRJNA47067.1577_1578	P.bryantii_B14	-0.25	0.606252635	0.68
dsgv	X752555.PRJNA47067.1578_1579	P.bryantii_B14	-0.19	0.588740195	0.75
dsgv	X752555.PRJNA47067.1579_1580	P.bryantii_B14	-0.28	0.577410448	0.63
dsgv	X752555.PRJNA47067.1581_1582	P.bryantii_B14	0.12	0.571314165	0.83
dsgv	X752555.PRJNA47067.1582_1583	P.bryantii_B14	0.26	0.567387132	0.65
dsgv	X752555.PRJNA47067.1583_1584	P.bryantii_B14	-1.00	0.560404287	0.08
dsgv	X752555.PRJNA47067.1584_1585	P.bryantii_B14	0.22	0.630364696	0.73
dsgv	X752555.PRJNA47067.1585_1586	P.bryantii_B14	-0.21	0.616970326	0.73
dsgv	X752555.PRJNA47067.160_161	P.bryantii_B14	-0.61	0.62051809	0.33
dsgv	X752555.PRJNA47067.1606_1607	P.bryantii_B14	-0.14	0.612788837	0.82
vsgv	X752555.PRJNA47067.1636_1637	P.bryantii_B14	0.18	0.139633594	0.19
dsgv	X752555.PRJNA47067.1680_1681	P.bryantii_B14	1.11	0.588371259	0.06
dsgv	X752555.PRJNA47067.1688_1689	P.bryantii_B14	-0.63	0.638789137	0.33
dsgv	X752555.PRJNA47067.1690_1691	P.bryantii_B14	-0.72	0.597232349	0.23
dsgv	X752555.PRJNA47067.1691_1692	P.bryantii_B14	-0.18	0.582159485	0.76
dsgv	X752555.PRJNA47067.1692_1693	P.bryantii_B14	-0.24	0.588623219	0.68
dsgv	X752555.PRJNA47067.1693_1694	P.bryantii_B14	-0.17	0.597529359	0.77
dsgv	X752555.PRJNA47067.1694_1695	P.bryantii_B14	-0.64	0.580817452	0.28
dsgv	X752555.PRJNA47067.1695_1696	P.bryantii_B14	-0.58	0.573537048	0.32
dsgv	X752555.PRJNA47067.18_19	P.bryantii_B14	-0.70	0.632118136	0.27
dsgv	X752555.PRJNA47067.189_190	P.bryantii_B14	0.83	0.650646464	0.20
dsgv	X752555.PRJNA47067.1968_1969	P.bryantii_B14	-0.78	0.563348783	0.17
dsgv	X752555.PRJNA47067.1973_1974	P.bryantii_B14	0.40	0.648617299	0.54
dsgv	X752555.PRJNA47067.1976_1977	P.bryantii_B14	-0.28	0.676616187	0.67
dsgv	X752555.PRJNA47067.199_200	P.bryantii_B14	0.29	0.600420363	0.63
dsgv	X752555.PRJNA47067.1997_1998	P.bryantii_B14	-0.57	0.607326	0.35
dsgv	X752555.PRJNA47067.2029_2030	P.bryantii_B14	-1.03	0.593363124	0.09
dsgv	X752555.PRJNA47067.2046_2047	P.bryantii_B14	-0.63	0.620317524	0.31
dsgv	X752555.PRJNA47067.2097_2098	P.bryantii_B14	-0.36	0.615966909	0.56
dsgv	X752555.PRJNA47067.2098_2099	P.bryantii_B14	-0.95	0.599592102	0.12

dsgv	X752555.PRJNA47067.2099_2100	P.bryantii_B14	-0.11	0.651615235	0.86
dsgv	X752555.PRJNA47067.2100_2101	P.bryantii_B14	0.03	0.592719307	0.97
dsgv	X752555.PRJNA47067.2141_2142	P.bryantii_B14	-0.17	0.573085224	0.77
dsgv	X752555.PRJNA47067.2148_2149	P.bryantii_B14	-0.75	0.566406758	0.19
dsgv	X752555.PRJNA47067.2309_2312	P.bryantii_B14	-0.54	0.574152566	0.35
dsgv	X752555.PRJNA47067.2317_2319	P.bryantii_B14	-0.53	0.811523829	0.52
dsgv	X752555.PRJNA47067.2329_2330	P.bryantii_B14	0.13	0.608036199	0.83
dsgv	X752555.PRJNA47067.2330_2331	P.bryantii_B14	0.42	0.591130353	0.47
dsgv	X752555.PRJNA47067.2334_2335	P.bryantii_B14	-0.77	0.647263269	0.24
dsgv	X752555.PRJNA47067.2335_2336	P.bryantii_B14	-0.32	0.58068053	0.58
dsgv	X752555.PRJNA47067.2336_2337	P.bryantii_B14	-0.05	0.577753597	0.93
dsgv	X752555.PRJNA47067.2338_2339	P.bryantii_B14	-1.33	0.567701744	0.02
dsgv	X752555.PRJNA47067.2343_2344	P.bryantii_B14	0.04	0.585384485	0.94
dsgv	X752555.PRJNA47067.2411_2412	P.bryantii_B14	-0.24	0.58939535	0.69
vsgv	X752555.PRJNA47067.2415_2416	P.bryantii_B14	-0.02	0.194545634	0.92
vsgv	X752555.PRJNA47067.2447_2449	P.bryantii_B14	0.19	0.175876373	0.27
dsgv	X752555.PRJNA47067.255_256	P.bryantii_B14	-0.93	0.580471112	0.11
dsgv	X752555.PRJNA47067.258_260	P.bryantii_B14	-0.07	0.831137282	0.93
dsgv	X752555.PRJNA47067.262_263	P.bryantii_B14	-0.16	0.582652705	0.78
dsgv	X752555.PRJNA47067.2630_2631	P.bryantii_B14	0.03	0.581236948	0.96
dsgv	X752555.PRJNA47067.2646_2647	P.bryantii_B14	-0.39	0.592960273	0.51
dsgv	X752555.PRJNA47067.2647_2648	P.bryantii_B14	-0.76	0.621468119	0.23
dsgv	X752555.PRJNA47067.2648_2650	P.bryantii_B14	-0.39	0.704605474	0.58
dsgv	X752555.PRJNA47067.2650_2652	P.bryantii_B14	0.34	0.766828568	0.66
dsgv	X752555.PRJNA47067.2652_2653	P.bryantii_B14	0.13	0.621565986	0.84
dsgv	X752555.PRJNA47067.27_28	P.bryantii_B14	-0.64	0.585899946	0.28
dsgv	X752555.PRJNA47067.2762_2763	P.bryantii_B14	0.56	0.571131247	0.33
dsgv	X752555.PRJNA47067.2792_2793	P.bryantii_B14	0.52	0.575168382	0.37
vsgv	X752555.PRJNA47067.2807_2808	P.bryantii_B14	0.29	0.176992266	0.10
vsgv	X752555.PRJNA47067.3017_3019	P.bryantii_B14	0.13	0.118282944	0.26
dsgv	X752555.PRJNA47067.3022_3024	P.bryantii_B14	0.60	1.126512118	0.59
vsgv	X752555.PRJNA47067.3038_3049	P.bryantii_B14	0.04	0.028382972	0.13
vsgv	X752555.PRJNA47067.3049_3050	P.bryantii_B14	0.20	0.166725249	0.22
vsgv	X752555.PRJNA47067.3050_3051	P.bryantii_B14	0.07	0.070833169	0.33
dsgv	X752555.PRJNA47067.3139_3140	P.bryantii_B14	0.19	0.631386814	0.76
dsgv	X752555.PRJNA47067.3141_3142	P.bryantii_B14	0.65	0.566411145	0.25
dsgv	X752555.PRJNA47067.3168_3169	P.bryantii_B14	-0.31	0.567244357	0.59
dsgv	X752555.PRJNA47067.3313_3314	P.bryantii_B14	-1.64	0.55223745	0.00
dsgv	X752555.PRJNA47067.3522_3523	P.bryantii_B14	0.38	0.603092636	0.53
dsgv	X752555.PRJNA47067.3528_3529	P.bryantii_B14	-0.21	0.576007037	0.72
dsgv	X752555.PRJNA47067.3590_3591	P.bryantii_B14	-1.15	0.584523228	0.05
dsgv	X752555.PRJNA47067.3591_3592	P.bryantii_B14	-0.48	0.59216842	0.42
dsgv	X752555.PRJNA47067.3592_3593	P.bryantii_B14	-1.43	0.602927673	0.02
dsgv	X752555.PRJNA47067.406_407	P.bryantii_B14	0.12	0.6140078	0.85

dsgv	X752555.PRJNA47067.407_408	P.bryantii_B14	0.50	0.577979745	0.39
dsgv	X752555.PRJNA47067.408_409	P.bryantii_B14	-1.03	0.586081434	0.08
dsgv	X752555.PRJNA47067.409_410	P.bryantii_B14	0.53	0.591747542	0.37
dsgv	X752555.PRJNA47067.410_411	P.bryantii_B14	0.64	0.582129509	0.28
dsgv	X752555.PRJNA47067.411_412	P.bryantii_B14	0.23	0.57126977	0.68
dsgv	X752555.PRJNA47067.412_413	P.bryantii_B14	-0.21	0.579340521	0.71
dsgv	X752555.PRJNA47067.48_49	P.bryantii_B14	0.18	0.701429831	0.79
dsgv	X752555.PRJNA47067.49_50	P.bryantii_B14	0.35	0.574659327	0.55
dsgv	X752555.PRJNA47067.514_516	P.bryantii_B14	-0.99	0.566600823	0.08
dsgv	X752555.PRJNA47067.516_517	P.bryantii_B14	-0.80	0.585834934	0.18
vsgv	X752555.PRJNA47067.544_545	P.bryantii_B14	-0.30	0.250158262	0.23
dsgv	X752555.PRJNA47067.55_56	P.bryantii_B14	-0.26	0.585915908	0.66
vsgv	X752555.PRJNA47067.60_61	P.bryantii_B14	0.09	0.214479801	0.67
dsgv	X752555.PRJNA47067.609_610	P.bryantii_B14	-0.27	0.594700919	0.65
dsgv	X752555.PRJNA47067.613_614	P.bryantii_B14	0.78	0.614933355	0.21
dsgv	X752555.PRJNA47067.614_615	P.bryantii_B14	-1.33	0.596219576	0.03
dsgv	X752555.PRJNA47067.615_616	P.bryantii_B14	-0.25	0.624907486	0.69
dsgv	X752555.PRJNA47067.651_652	P.bryantii_B14	-0.45	0.599279309	0.46
dsgv	X752555.PRJNA47067.652_653	P.bryantii_B14	0.26	0.584025566	0.65
dsgv	X752555.PRJNA47067.655_656	P.bryantii_B14	0.56	0.571158804	0.33
dsgv	X752555.PRJNA47067.656_657	P.bryantii_B14	-0.23	0.636135361	0.71
dsgv	X752555.PRJNA47067.657_658	P.bryantii_B14	-0.42	0.653940837	0.52
dsgv	X752555.PRJNA47067.662_663	P.bryantii_B14	-0.95	0.576258079	0.10
dsgv	X752555.PRJNA47067.663_664	P.bryantii_B14	0.12	0.604868417	0.85
dsgv	X752555.PRJNA47067.664_665	P.bryantii_B14	-0.25	0.607512472	0.68
dsgv	X752555.PRJNA47067.669_671	P.bryantii_B14	0.37	0.606231978	0.54
dsgv	X752555.PRJNA47067.673_674	P.bryantii_B14	-0.68	0.571869616	0.23
dsgv	X752555.PRJNA47067.696_697	P.bryantii_B14	0.03	0.575029056	0.96
dsgv	X752555.PRJNA47067.697_698	P.bryantii_B14	0.38	0.630953694	0.54
dsgv	X752555.PRJNA47067.699_700	P.bryantii_B14	-0.08	0.594027708	0.90
vsgv	X752555.PRJNA47067.700_701	P.bryantii_B14	0.01	0.023168746	0.55
dsgv	X752555.PRJNA47067.703_704	P.bryantii_B14	-0.43	0.590075274	0.47
vsgv	X752555.PRJNA47067.704_705	P.bryantii_B14	0.02	0.174196354	0.91
dsgv	X752555.PRJNA47067.708_709	P.bryantii_B14	-1.19	0.609224453	0.05
dsgv	X752555.PRJNA47067.712_713	P.bryantii_B14	0.76	0.589841694	0.20
vsgv	X752555.PRJNA47067.720_721	P.bryantii_B14	0.38	0.211448999	0.07
dsgv	X752555.PRJNA47067.721_722	P.bryantii_B14	0.87	0.578773362	0.14
vsgv	X752555.PRJNA47067.722_723	P.bryantii_B14	0.03	0.163579876	0.88
vsgv	X752555.PRJNA47067.724_725	P.bryantii_B14	0.03	0.064909932	0.63
dsgv	X752555.PRJNA47067.899_900	P.bryantii_B14	0.52	0.616389817	0.40
dsgv	X752555.PRJNA47067.901_902	P.bryantii_B14	-0.50	0.674346546	0.46
dsgv	X752555.PRJNA47067.902_903	P.bryantii_B14	0.14	0.589499667	0.81
dsgv	X752555.PRJNA47067.903_904	P.bryantii_B14	-0.14	0.635726195	0.82
dsgv	X752555.PRJNA47067.904_905	P.bryantii_B14	-1.09	0.560470053	0.05

dsgv	X752555.PRJNA47067.93_94	P.bryantii_B14	-0.26	0.583622169	0.66
dsgv	X752555.PRJNA47067.938_939	P.bryantii_B14	0.63	0.580423047	0.28
dsgv	X752555.PRJNA47067.939_940	P.bryantii_B14	-0.04	0.617196565	0.95
dsgv	X752555.PRJNA47067.941_942	P.bryantii_B14	0.73	0.57479138	0.20
dsgv	X752555.PRJNA47067.942_943	P.bryantii_B14	-0.92	0.588559441	0.12
dsgv	X752555.PRJNA47067.943_944	P.bryantii_B14	0.41	0.601460201	0.49
dsgv	X752555.PRJNA47067.948_949	P.bryantii_B14	-0.26	0.58978339	0.66
dsgv	X752555.PRJNA47067.953_955	P.bryantii_B14	0.05	0.722117878	0.95
dsgv	X752555.PRJNA47067.973_975	P.bryantii_B14	-0.27	0.658496452	0.68
dsgv	X752555.PRJNA47067.975_976	P.bryantii_B14	0.12	0.586886219	0.84
dsgv	X752555.PRJNA47067.976_977	P.bryantii_B14	0.32	0.581036492	0.58
dsgv	X752555.PRJNA47067.977_978	P.bryantii_B14	0.83	0.578616044	0.16
dsgv	X752555.PRJNA47067.978_979	P.bryantii_B14	-1.00	0.757721334	0.19
dsgv	X752555.PRJNA47067.979_980	P.bryantii_B14	0.00	0.577654531	0.99
dsgv	X752555.PRJNA47067.981_982	P.bryantii_B14	-0.06	0.66134461	0.92
dsgv	X752555.PRJNA47067.982_983	P.bryantii_B14	-0.01	0.597916891	0.99
dsgv	X911104.PRJNA59737.0_1	W.cibaria_KACC_11862	-11.34	23.49000632	0.71
dsgv	X911104.PRJNA59737.1187_1188	W.cibaria_KACC_11862	0.22	0.638904037	0.79
vsgv	X911104.PRJNA59737.1907_1908	W.cibaria_KACC_11862	0.02	0.103964107	0.86
vsgv	X911104.PRJNA59737.2127_2128	W.cibaria_KACC_11862	0.06	0.11518967	0.71
vsgv	X911104.PRJNA59737.227_228	W.cibaria_KACC_11862	0.03	0.060335054	0.73
vsgv	X911104.PRJNA59737.261_263	W.cibaria_KACC_11862	0.60	0.172360281	0.18
vsgv	X911104.PRJNA59737.306_307	W.cibaria_KACC_11862	-0.01	0.205676284	0.96
dsgv	X911104.PRJNA59737.447_449	W.cibaria_KACC_11862	-3.01	0.335273728	0.01
dsgv	X911104.PRJNA59737.449_450	W.cibaria_KACC_11862	0.63	0.226608329	0.22
dsgv	X911104.PRJNA59737.454_455	W.cibaria_KACC_11862	0.65	0.218970446	0.21
vsgv	X911104.PRJNA59737.732_734.	W.cibaria_KACC_11862	-0.13	0.059391396	0.28
vsgv	X911104.PRJNA59737.974_975	W.cibaria_KACC_11862	0.060532700	0.05	0.46

Table S8. Correlation analysis between heritable rumen metabolites and Prevotella.

Metabolite	h^2	FDR		
		Prevotella_bryantii	Prevotella_mizrahii	Prevotella_sp._AGR2160
6-Amino-9H-purine-9-propanoic acid	0.26	0.57	0.49	0.51
Ceftazidime anhydrous	0.24	0.00	0.08	0.03
Ala-Leu	0.30	0.08	0.01	0.01
Dihydroprudomenin	0.30	0.29	0.43	0.52
L-Alanyl-l-Valine	0.58	0.01	0.01	0.01
2',3'-Didehydro-2',3'-dideoxycytidine	0.64	0.00	0.00	0.00
Haloxyfop-P	0.27	0.98	0.29	0.28
Linalool (8-hydroxydihydro-)	0.30	0.00	0.00	0.00
3'-O-Methylguanosine	0.32	0.33	0.08	0.10
(+-)-Carbovir	0.26	0.13	0.04	0.10
4-Pyridoxic Acid	0.26	0.77	0.26	0.18
N-Acetyl-L-glutamate 5-semialdehyde	0.42	0.24	0.26	0.20
Uracil	0.77	0.23	0.21	0.10
Niazirin	0.36	0.32	0.28	0.13
(7R)-7-(5-Carboxy-5-oxopentanoyl)aminocephalosporinate	0.26	0.83	0.23	0.19
Galactose lactate	0.27	0.14	0.82	0.90
2-Deoxy-L-ribono-1,4-lactone	0.80	0.08	1.00	0.90
Myo-Inositol	0.44	0.01	0.00	0.00
Prenyl cis-caffeate	0.26	0.05	0.06	0.02
7,8-Dihydropteroic acid	0.42	0.66	0.01	0.01
Gentisate aldehyde	0.21	0.17	0.09	0.11
Niflumic Acid	0.42	0.01	0.00	0.00
5-Aminopentanal	0.29	0.43	0.31	0.30
2-Hydroxypyridine	0.23	0.96	0.92	0.98
Neuraminic acid	0.29	0.85	0.87	0.73
Nepsilon-Acetyl-L-lysine	0.20	0.01	0.08	0.04
3,4-Dihydroxy-2-hydroxymethyl-1-pyrrolidinepropanamide	0.24	0.01	0.00	0.00
2-Aminomuconic acid semialdehyde	0.21	0.22	0.96	0.93
N-formimidoyl-glutamic acid	0.54	0.02	0.00	0.00
Acetamidopropanal	0.58	0.41	0.83	0.93
Deacetyldiltiazem	0.34	0.05	0.16	0.16
3-Hydroxy-3-methylglutarate	0.44	0.62	0.27	0.30
D-Erythrose	0.47	0.93	0.28	0.34
2-C-Methyl-1,4-erythrono-D-lactone	0.55	0.91	0.43	0.52
Verubecestat	0.27	0.60	0.16	0.16
3-(4-Hydroxyphenyl)-3,5,6,8-tetrahydro-2H-chromene-4,7-dione	0.22	0.64	0.07	0.07
Tetranor-PGEM	0.28	0.64	0.14	0.23
P-CHLOROPHENYLALANINE	0.20	0.68	0.02	0.02

4-Oxododecanedioic acid	0.42	0.00	0.00	0.00
(-)-11-Hydroxy-9,15,16-trioxooctadecanoic acid	0.53	0.00	0.01	0.01
9,10,13-TriHOME	0.43	0.85	0.00	0.00
Triterpenoids	0.30	0.03	0.10	0.07
3,4-Dimethyl-5-pentyl-2-furantridecanoic acid	0.22	0.85	0.24	0.21
Lysyltryptophan	0.20	0.56	0.65	0.65
MG(0:0/14:1(9Z)/0:0)	0.68	0.01	0.00	0.00
M-Coumaric acid	0.23	0.88	0.07	0.06
2,2'-(3-methylcyclohexane-1,1-diyl)diacetic acid	0.25	0.02	0.00	0.00
Floionolic acid	0.38	0.23	0.00	0.00
16-Hydroxy-10-oxohexadecanoic acid	0.21	0.55	0.12	0.19
9,10-DiHOME	0.29	0.91	0.01	0.02
(9S,10S)-9,10-dihydroxyoctadecanoate	0.30	0.01	0.00	0.00
Ethanone, 1-(4-butylphenyl)-	0.59	0.34	0.84	0.86
4-ene-Valproic acid	0.26	0.33	0.40	0.32
1-Methyladenosine	0.41	0.22	0.73	0.83
2-Hydroxydecanedioic acid	0.41	0.11	0.38	0.34
Oxoglutaric acid	0.45	0.04	0.08	0.09
9-Oxo-nonanoic acid	0.52	0.35	0.36	0.34
SM(d16:2(4E,8Z)/20:5(6E,8Z,11Z,14Z,17Z)-OH(5))	0.30	0.00	0.00	0.00
Isophorone	0.23	0.01	0.18	0.12
2,6-Dichloro-4-nitrophenol	0.40	0.49	0.00	0.00
Tropolone	0.35	0.01	0.60	0.62
3-Deoxyuridine	0.25	0.37	0.83	0.85
(+/-)-3-Hydroxynonanoic acid	0.50	0.33	0.01	0.01
D-glycero-L-galacto-Octulose	0.23	0.11	0.76	0.84
3-Phenyl-1-(2,4,6-trihydroxyphenyl)propan-1-one	0.30	0.61	0.03	0.03
Frenolicin B	0.51	0.45	0.00	0.00
N-Acetyl-b-D-galactosamine	0.23	0.97	0.31	0.29
4-Hydroxyproline galactoside	0.25	0.01	0.01	0.01
L-Leucyl-L-Alanine	0.23	0.15	0.03	0.02
Pantoyllactone glucoside	0.41	0.51	0.82	0.83
2-(Malonylamino)benzoic acid	0.21	0.04	0.14	0.09
Guadecitabine	0.30	0.00	0.00	0.00
2-Hydroxypentanoic Acid	0.21	0.77	0.14	0.10
3,4,5,6-Tetrahydrohippuric acid	0.21	0.10	0.00	0.00
Isoimperatorin	0.44	0.00	0.20	0.17
Glucolepidiin	0.30	0.05	0.01	0.05
Hydroxyphenylacetylglycine	0.37	0.31	0.14	0.11

N(G)-Nitroarginine-4-nitroanilide	0.23	0.00	0.00	0.00
LysoPE(18:4(6Z,9Z,12Z,15Z)/0:0)	0.25	0.93	0.02	0.01
Isoleucylproline	0.67	0.06	0.00	0.00
Cyclacillin	0.26	0.47	0.16	0.22
Integerrimine	0.23	0.72	0.28	0.28
1,2,3,4,5,6-Hexahydro-5-methyl-7H-cyclopenta[b]pyridin-7-one	0.26	0.00	0.00	0.00
4-Bis(2-hydroxyethyl)amino-L-phenylalanine	0.62	0.02	0.17	0.17
Acetaminophen glucuronide	0.72	0.45	0.92	0.83
Gladiatoside C2	0.24	0.01	0.00	0.00
Betanin	0.46	0.00	0.00	0.00
Phaseolus epsilon	0.33	0.10	0.26	0.28
Ethyl glucuronide	0.21	0.17	0.44	0.56
Coumatetralyl	0.27	0.77	0.85	0.87
Nikkomycin	0.43	0.98	0.73	0.66
3-Pyridinebutanoic acid	0.26	0.87	0.42	0.37
Junosine	0.28	0.00	0.00	0.00
5-Ethyl-1,2,3,4,5,6-hexahydro-7H-cyclopenta[b]pyridin-7-one	0.33	0.00	0.00	0.00
Camonagrel	0.26	0.00	0.19	0.21
6-Methylthioguanine	0.66	0.10	0.00	0.00
Pisatoside	0.45	0.22	0.52	0.53
Glutaminylhistidine	0.33	0.12	0.05	0.08
P-Tolyl Sulfate	0.24	0.85	0.05	0.04
Ile Phe	0.25	0.14	0.01	0.01
6-Hydroxysandoricin	0.32	0.00	0.00	0.00
6-Hydroxymelatonin	0.54	0.02	0.03	0.02
2-Hydroxy-3-Methylbutyric Acid	0.24	0.93	0.07	0.04
(E)-2-Methyl-2-buten-1-ol O-beta-D-Glucopyranoside	0.47	0.06	0.73	0.61
1,2-Cyclohexanedicarboxylic acid	0.35	0.45	0.88	0.83
Perindopril	0.49	0.32	0.52	0.44
D-Gluconic acid	0.37	0.00	0.00	0.00
N-OCTANOYL-L-HOMOSERINE LACTONE	0.42	0.91	0.35	0.28
5-Hydroxykynurenamine	0.23	0.25	0.34	0.30
Undecanedioic acid	0.26	0.00	0.00	0.00
5-(2-Furanyl)-1,2,3,4,5,6-hexahydro-7H-cyclopenta[b]pyridin-7-one	0.26	1.00	0.28	0.29
Arphamenine B	0.55	0.84	0.26	0.23
Cinncassiol C3	0.26	0.27	0.61	0.50
1-Octen-3-yl primeveroside	0.42	0.24	0.00	0.00
Hippuric Acid	0.88	0.66	0.45	0.53
4-Heptyloxyphenol	0.25	0.02	0.05	0.04

Asparaginylaspartic acid	0.58	0.01	0.02	0.01
Oxypurinol	0.46	0.00	0.01	0.00
Macaridine	0.40	0.08	0.01	0.01
Histidinyl-Asparagine	0.67	0.03	0.00	0.00
Caprylic acid	0.72	0.22	0.73	0.56
(+/-)-3',4'-Methylenedioxy-5,7-dimethylepicatechin	0.31	0.17	0.01	0.01
N-octyl-beta-D-thioglucopyranoside	0.44	0.02	0.62	0.37
Cfp-aaf-pab	0.25	0.49	0.99	0.93
7-Hydroxy-R-phenprocoumon	0.23	0.20	0.89	0.83
Fructose lactate	0.30	0.06	0.26	0.26
Valproic acid	0.57	0.05	0.00	0.00
Zilascorb	0.30	0.00	0.00	0.00
Prostaglandin F-main urinary metabolite	0.25	0.54	0.38	0.41
Tributyl citrate	0.45	0.01	0.13	0.12
(S)-p-Menth-1-ene-4,7-diol 4-glucoside	0.60	0.56	0.31	0.32
Octanoate	0.38	0.07	0.02	0.02
Lysylhydroxyproline	0.42	0.74	0.83	0.83
9-Hydroxy-7-megastigmen-3-one glucoside	0.26	0.85	1.00	0.89
11-Hydroxy-9-tridecenoic acid	0.50	0.37	0.48	0.38
3a,6b,7a,12a-Tetrahydroxy-5b-cholanoic acid	0.31	0.37	0.77	0.87
Ethoxyquin	0.22	0.00	0.00	0.00
Alpha-Bisabolol oxide A	0.39	0.27	0.70	0.63
24-Epibrassinolide	0.26	0.24	0.73	0.63
LysoPC(18:3(9Z,12Z,15Z)/0:0)	0.43	0.89	0.73	0.59
Cis-4-Decenedioic acid	0.40	0.02	0.65	0.48
3beta-Hydroxy-17-(1H-imidazol-1-yl)androsta-5,16-diene	0.53	0.19	0.00	0.00
Taurochenodesoxycholic acid	0.53	0.00	0.00	0.00
Benfuracarb	0.36	0.33	0.41	0.45
Thromboxane A2	0.27	0.70	0.00	0.00
GPEtn(16:0/12:0)	0.31	0.43	0.30	0.32
1,2-Naphthoquinone	0.25	0.15	0.01	0.01
Glycerophosphoserine	0.22	0.29	0.23	0.24
9,13-Dihydroxy-4-megastigmen-3-one 9-glucoside	0.43	0.16	0.83	0.56
4-Hydroxycyclohexylcarboxylic acid	0.58	0.72	0.33	0.34
25-Acetylvulgaroside	0.25	0.00	0.00	0.00
Diacetyl-L-tartaric Anhydride	0.31	0.25	0.12	0.10
Valproylglycine	0.21	0.66	0.97	0.87
Trans-Zeatin	0.40	0.43	0.24	0.19
Butyl 4-aminobenzoate	0.27	0.29	0.98	0.92
16-Hydroxyhexadecanoylcarnitine	0.28	0.00	0.00	0.00
9(S)-HpOTrE	0.43	0.33	0.45	0.54

9,10,13-Trihydroxystearic acid	0.65	0.78	0.01	0.01
Dioctyl succinate	0.51	0.03	0.73	0.71
2,4-Dichloro-6-nitrophenol	0.44	0.40	0.30	0.33
DTMP	0.23	0.02	0.00	0.00
PG(16:0/0:0)[U]	0.47	0.24	0.11	0.10
Aprobarbital	0.39	0.49	0.00	0.00
2-n-Propyl-4-oxopentanoic acid	0.34	0.32	0.54	0.47
Val Leu Gly	0.33	0.00	0.00	0.00
1,2-Dihydroxy-3,4-epoxy-1,2,3,4-tetrahydronaphthalene	0.44	0.33	0.99	0.92
Thromboxane B2	0.37	0.63	0.80	0.73
Prostaglandin D1	0.27	0.26	0.00	0.00
S-(2-carboxypropyl)-Cysteamine	0.38	0.17	0.97	0.92
LysoPS(16:0/0:0)	0.53	0.00	0.00	0.00
3'-demethyletoposide	0.35	0.00	0.00	0.00
1-[3,4-Dihydroxy-5-(hydroxymethyl)oxolan-2-yl]-4-hydroxyhydropyridin-2-one	0.40	0.74	0.71	0.76
Monensin A	0.36	0.64	0.92	0.93
[3-[2-Aminoethoxy(hydroxy)phosphoryl]oxy-2-hydroxypropyl] hexadecanoate	0.40	0.00	0.00	0.00
Gemfibrozil	0.81	0.81	0.62	0.56
Kaempferol 3-(6-acetylgalactoside)	0.45	0.43	0.39	0.34
Glucosylsphingosine	0.31	0.81	0.92	0.83
1,2,3-Benzotriazine	0.29	0.01	0.00	0.00
Cynaropicrin	0.53	0.02	0.02	0.01
D-Galactose	0.42	0.01	0.00	0.00
D-Glucose	0.64	0.05	0.00	0.00
Mycophenolic acid	0.22	0.62	0.64	0.63
Norlaudanosoline	0.28	0.08	0.01	0.01
P-Coumaric Acid	0.22	0.96	0.11	0.11
Phenyllactic acid	0.21	0.11	0.99	0.90
D-Glucamine	0.37	0.00	0.00	0.00
(2E)-2-butylidene-4-hydroxy-5-methyl-3(2H)-furanone	0.24	0.00	0.01	0.00
Prolyl-Lysine	0.31	0.18	0.62	0.55
Glutamic acid-betaxanthin	0.39	0.66	0.34	0.34
Guanidoacetic acid	0.20	0.37	0.00	0.00
Vidarabine	0.47	0.00	0.00	0.00
Tryptophol	0.24	0.45	0.14	0.10
Sudan I	0.21	0.00	0.00	0.00
(3Z)-phytochromobilin	0.35	0.93	0.35	0.28
N-(5-acetamidopentyl)acetamide	0.23	0.85	0.71	0.73
Gly-Ile	0.32	0.02	0.00	0.00
Lotaustralin	0.24	0.02	0.00	0.00

Acarviosin	0.22	0.06	0.00	0.00
Glucose lactate pyruvate glutamate	0.53	0.58	0.76	0.83
Glycyl-leucine	0.36	0.00	0.00	0.00
Valylhydroxyproline	0.35	0.91	0.59	0.55
Acrylamide-sodium acrylate resin	0.71	0.03	0.00	0.00
Valeraldehyde propyleneglycol acetal	0.22	0.00	0.00	0.00
Neotussilagine	0.25	0.98	0.31	0.28
Adenosine	0.51	0.00	0.00	0.00
(2-Ethyl-2,4,4-trimethyl-1,3-oxazolidin-3-yl)oxidanyl	0.41	0.49	0.92	0.87
Cyclocreatine	0.21	0.07	0.52	0.50
Asparaginyl-Proline	0.52	0.00	0.00	0.00
Glutamylarginine	0.47	0.00	0.00	0.00
D-Ribulose	0.21	0.71	0.43	0.41
Methyldopa	0.96	0.41	0.14	0.13
Glycyl-Gamma-glutamate	0.31	0.26	0.98	1.00
N-[4-[Acetyl(3-aminopropyl)amino]butyl]-N-(3-aminopropyl)acetamide	0.21	0.00	0.00	0.00
Lys Val	0.24	0.00	0.00	0.00
Equol 7-O-glucuronide	0.23	0.60	0.18	0.17
D-Tagatose 6-phosphate	0.34	0.18	0.97	0.93
Diflunisal glucuronide ether	0.40	0.26	0.89	0.83
D-fructose 6-phosphate	0.40	0.01	0.00	0.00
Isobutylidene	0.21	0.26	0.64	0.65
Lomefloxacin	0.53	0.11	0.96	0.84
Miserotoxin	0.38	0.00	0.01	0.00
Picolinic Acid	0.22	0.03	0.39	0.35
4-Trimethylammoniobutanoic Acid	0.27	0.00	0.29	0.21
N1-Acetylspermine	0.37	0.00	0.00	0.00
Besifloxacin	0.22	0.22	0.62	0.52
Neamine	0.32	0.16	0.62	0.67
(2R,3R)-(-)-2,3-Butanediol	0.30	0.12	0.05	0.06
Clozapine	0.48	0.97	0.72	0.80
Diflunisal glucuronide ester	0.39	0.91	0.88	0.77
Levulinic acid	0.21	0.04	0.00	0.00
2',3' cyclic CMP	0.32	0.00	0.00	0.00
2-amino-3,7-dideoxy-D-threo-hept-6-ulosonate	0.25	0.00	0.00	0.00
2-Aminoheptanedioic acid	0.27	0.66	0.07	0.06
7,8-Dihydrovomifoliol 9-[rhamnosyl-(1->6)-glucoside]	0.31	0.01	0.09	0.10
Ginsenoside Rh6	0.71	0.74	0.59	0.42
Alanylisoleucine	0.26	0.02	0.00	0.00
Guanosine	0.30	0.00	0.00	0.00

Flumethasone	0.27	0.20	0.65	0.55
DG(2:0/0:0/18:3(10,12,15)-OH(9))	0.22	0.01	0.00	0.01
Monensin	0.66	0.25	0.31	0.19
Cytarabine	0.23	0.00	0.00	0.00
LysoPE(18:3(6Z,9Z,12Z)/0:0)	0.37	0.00	0.00	0.00
Prostaglandin PGE2 1-glyceryl ester	0.29	0.00	0.00	0.00
1-(4Z,7Z,10Z,13Z,16Z,19Z-docosahexaenoyl)-glycero-3-phosphate	0.66	0.00	0.00	0.00
Momordicin II	0.56	0.33	0.28	0.42
Ganoderic acid F	0.27	0.00	0.01	0.02
N-Methacryloyl-L-glutamic acid	0.21	0.07	0.00	0.00
Xanthine	0.45	0.11	0.22	0.09
Venlafaxine	0.34	0.28	0.70	0.80
Eriodictyol 7-(6-trans-p-coumaroylglucoside)	0.39	0.00	0.00	0.00
3-(1-Hydroxymethyl-1-propenyl)pentanedioic acid	0.41	0.12	0.10	0.07
Venoterpine	0.20	0.61	0.24	0.23
1-Naphthylamine	0.29	0.00	0.00	0.00
(R)-2-(4-(Tert-butoxycarbonyl)morpholin-3-yl)acetic acid	0.34	1.00	0.06	0.05
3-oxo-2-propyl-1,4-dihydroisoquinoline-1-carboxylic acid	0.76	0.17	0.45	0.51
Austrobailignan 7	0.26	0.59	0.41	0.30
Monoacetyldiglyceride	0.26	0.02	0.01	0.01
Tyrosyl-Serine	0.35	0.82	0.09	0.10
Valylproline	0.73	0.15	0.00	0.00
5-Methyl-3'-Deoxyuridine	0.21	0.00	0.00	0.00
5,7-Megastigmadien-9-ol glucoside	0.22	0.77	0.97	0.87
N-(5-Amino-2-hydroxybenzoyl)glycine	0.21	0.41	0.80	0.84
6-Acetyl-1,2,3,4-tetrahydropyridine	0.29	0.00	0.00	0.00
Therafectin	0.42	0.33	0.31	0.42
4-(Phenylamino)benzoic acid	0.33	0.09	0.76	0.85
4-(3-Hydroxypropyl)morpholine	0.25	0.00	0.00	0.00
2-(4-Amino-1-isopropyl-1H-pyrazolo[3,4-d]pyrimidin-3-yl)-1H-indol-5-ol	0.58	0.02	0.00	0.00
L-phenylalanyl-L-proline	0.67	0.00	0.00	0.00
2-Aminobiphenyl	0.47	0.76	0.80	0.79
Serratol	0.20	0.00	0.00	0.00
N-(3-acetylphenyl)-4,5,6,7-tetrahydro-1-benzothiophene-3-carboxamide	0.31	0.07	0.01	0.01
3-Methyl-N-phenylaniline	0.22	0.74	0.60	0.65
7-methylsulfinylheptyl glucosinolate	0.38	0.05	0.06	0.05
Byakangelicol	0.21	0.33	0.00	0.00
20-trifluoro-LTB4	0.25	0.13	0.12	0.17

Traumatic Acid	0.39	0.11	0.83	0.63
D-Citrulline	0.29	0.00	0.00	0.00
Alpha-Ionone	0.24	0.01	0.46	0.35
Cerasinone	0.25	0.34	0.16	0.11
Vadocaine	0.39	0.00	0.00	0.00
(3b,6b,8b,12a)-8,12-Epoxy-7(11)-eremophilene-6-angeloyloxy-8,12-dimethoxy-3-ol	0.28	0.01	0.30	0.19
Dhv-PGE2	0.25	0.00	0.01	0.00
Aristospan	0.25	0.01	0.01	0.01
LysoPC(14:0/0:0)	0.21	0.00	0.00	0.00
Thienamycin	0.24	0.45	0.92	0.93
12,13-DHOME	0.22	0.26	0.40	0.36
Pheophorbide a	0.28	0.00	0.00	0.00
Glycyl-D-Alanine	0.43	0.00	0.00	0.00
Ritipenem	0.29	0.05	0.05	0.08
PE(16:0/0:0)	0.57	0.00	0.00	0.00
Vaccinoside	0.23	0.00	0.00	0.00
4-hydroxy Nonenal	0.21	0.00	0.01	0.00
9-Hydroxylinoleic acid	0.29	0.00	0.00	0.00
5(S),14(R)-Lipoxin B4	0.24	0.01	0.00	0.00
2,6-diamino-4-hydroxy-5-formamidopyrimidine	0.25	0.00	0.00	0.00
Patulin	0.27	0.00	0.00	0.00
20-carboxy-LTB4	0.22	0.17	0.26	0.28
4,5-Dimethoxybenzene-1,2-diamine	0.20	0.07	0.66	0.62
Trigonelline	0.27	0.00	0.00	0.00
FAPy-adenine	0.43	0.00	0.01	0.00
Morpholine	0.29	0.91	0.07	0.10
2,3-dinor-8-iso-PGF2a	0.26	0.02	0.01	0.00
5beta-CHOLANIC ACID-3alpha, 12alpha-DIOL N-(2-SULPHOETHYL)-AMIDE	0.30	0.01	0.04	0.05
Methyl (Z)-5-(1-propynyl)-2-thienylacrylate	0.52	0.93	0.90	0.93
Salicyluric acid	0.35	0.24	0.97	0.79
1-(5Z,8Z,11Z,14Z-eicosatetraenoyl)-sn-glycero-3-phosphate	0.22	0.00	0.00	0.00
P-Acetaminobenzaldehyde	0.20	0.01	0.42	0.35
Leu Pro Asp	0.23	0.71	0.23	0.21
Mycorradicin	0.22	0.20	0.64	0.67
Fasoracetam	0.75	0.08	0.00	0.00
Pergillin	0.32	0.17	0.00	0.00
L-3-(3-Hydroxy-4-pivaloyloxyphenyl)alanine	0.27	0.17	0.01	0.02
Propionylcarnitine	0.49	0.27	0.01	0.01
Diosbulbinoside F	0.32	0.00	0.00	0.00

Methoxamine	0.44	0.45	0.77	0.71
1-Pyrrolidinyloxy, 3-(aminocarbonyl)-2,2,5,5-tetramethyl-	0.24	0.17	0.00	0.00
6-Amino-N-[6-keto-6-(6-ketohexylamino)hexyl]hexanamide	0.22	0.47	0.13	0.09
5-(2-Aminopropyl)benzofuran	0.30	0.29	0.24	0.20
(-)-Salsoline	0.48	0.90	0.71	0.68
Tuliposide A	0.49	0.00	0.00	0.00
8-Aminoguanosine	0.59	0.00	0.00	0.00
20-hydroxyleukotriene B4	0.33	0.06	0.00	0.00
5-Hydroxy-6-methoxy-1h-indole-2-carboxylic acid	0.31	0.03	0.08	0.06
Beta-Thujaplicin	0.31	0.01	0.00	0.00
(3R)-3,4-Dihydroxy-3-(hydroxymethyl)butanenitrile 4-glucoside	0.22	0.07	0.02	0.07
Riboprine	0.22	0.01	0.04	0.03
2-Hydroxyestrone	0.54	0.06	0.01	0.00
3-Hydroxy-N6,N6,N6-trimethyl-L-lysine	0.21	0.17	0.84	0.90
Neomenthol-glucuronide	0.43	0.91	0.24	0.20
(+)-marmesin	0.26	0.33	0.39	0.42
(+)-cis-Khellactone	0.29	0.24	0.20	0.23
Diphenylamine	0.44	0.83	0.93	0.91
Val Val	0.28	0.62	0.16	0.18
Beta-D-Fructose 2-phosphate	0.34	0.02	0.29	0.28
Arginyllysine	0.20	0.03	0.00	0.00
Bafilomycin A1(Baf-A1)	0.48	0.49	0.34	0.34
Nandrolone	0.21	0.00	0.00	0.00
3-Indolepropionic acid	0.29	0.01	0.00	0.00
Metazin	0.22	0.16	0.58	0.65
Val Ile Ile	0.22	0.00	0.00	0.00
N-cyclopentyl-1-(3,4-dimethoxyphenyl)sulfonylpiperidine-3-carboxamide	0.21	0.01	0.14	0.12
Curcumin III	0.22	0.73	0.38	0.50
3a,7a,12a-Trihydroxy-5b-cholestan-26-al	0.39	0.38	0.28	0.21
(S)-(4-Hydroxy-phenyl)-(S)-piperidin-2-yl-acetic acid methyl ester hydrochloride	0.25	0.08	0.83	0.91
HT-2 Toxin	0.22	0.18	0.99	0.94
Megestrol	0.50	0.50	0.19	0.21
DG(20:2(11Z,14Z)/18:4(6Z,9Z,12Z,15Z)/0:0)	0.47	0.31	0.95	0.84
N-Methyl-L-Threonine	0.61	0.17	0.76	0.87
4,4'-DIMETHOXYDALBERGIONE	0.27	0.38	0.19	0.14
6-Fluoromevalonate	0.23	0.29	0.31	0.31
1,5-Isoquinolinediol	0.24	0.00	0.01	0.00

N-Methylaniline	0.52	0.05	0.75	0.56
26-Deoxyactein	0.77	0.32	0.19	0.21
Ecdysterone	0.42	0.04	0.92	1.00
2,3-Dinor-11b-PGF2a	0.23	0.09	0.07	0.04
6-Ketoprostaglandin E1	0.25	0.05	0.12	0.12
27-O-demethylrifabutin	0.31	0.08	0.01	0.01
DL-Difluoromethylornithine	0.23	0.02	0.31	0.38
Dehydrophytosphingosine	0.26	0.01	0.00	0.00
4-Hydroxy-3-polyprenylbenzoate	0.24	0.00	0.00	0.00
9,10-Epoxyoctadecanoic acid	0.35	0.02	0.00	0.00
Isoguvacine	0.22	0.34	0.07	0.04
Isonicotinic acid	0.44	0.00	0.05	0.04
L-2-Aminobutyric acid	0.21	0.21	0.80	0.65
Polysorbate 80	0.66	0.74	0.34	0.41
LysoPE(15:0/0:0)	0.36	0.73	0.29	0.35
Ile-Ile-Ile-Pro	0.40	0.00	0.09	0.06
Olamufloxacin	0.50	0.76	0.42	0.56
DG(PGJ2/2:0/0:0)	0.36	0.98	0.65	0.62
(10E,12Z)-(9S)-9-Hydroperoxyoctadeca-10,12-dienoic acid	0.35	0.89	0.02	0.01
Avocadyne 4-acetate	0.23	0.98	0.53	0.54
13,14-Dihydro PGF-1a	0.42	0.71	0.50	0.54
Caproic acid	0.47	0.21	0.00	0.00
2,3-dinor Prostaglandin E1	0.60	0.77	0.92	0.93
4,5-Dihydrovomifoliol	0.28	0.27	0.66	0.66
(R)-11,12,13-Trinor-1(5),6,9-guaiatrien-8-one	0.38	0.01	0.44	0.59
Heptyl 4-hydroxybenzoate	0.34	0.24	0.83	0.73
(R)-1-Octen-3-ol	0.34	0.88	0.97	0.98
Gingerol	0.34	0.02	0.14	0.13
Dihydrohydroxy-O-methylsterigmatocystin	0.21	0.91	0.31	0.34
(S,E)-Zearalenone	0.39	0.75	0.63	0.61
12-O-D-Glucuronoside-13-hydroxyoctadec-9Z-enoate	0.68	0.69	0.29	0.28
Desglucocheirotoxin	0.23	0.47	0.65	0.56
Nodakenetic	0.31	0.93	0.07	0.07
Demethylsuberosin	0.30	0.78	0.04	0.04
Mitoflaxone	0.25	0.45	0.57	0.56
Apigenin 7,4'-dimethyl ether	0.26	0.99	0.19	0.23
Tryptophyl-Serine	0.31	0.04	0.01	0.00
4-Hydroxydecanedioylcarnitine	0.21	0.40	0.01	0.01
P-Menthane-3,8-diol	0.38	0.03	0.01	0.00
1-Hydroxy-3-octanone	0.44	0.77	0.36	0.28
4-Megastigmene-6a,9R-diol 9-[apiosyl-(1->6)-glucoside]	0.45	0.97	0.14	0.11

2-(Acetylamino)-2-deoxy-alpha-D-mannopyranose	0.38	0.16	0.00	0.00
7-Nonene-3,5-diyn-1-ol	0.24	0.44	0.03	0.02
P-Menthan-trans-2,5-diol	0.41	0.54	0.60	0.55
3-ethylphenyl Sulfate	0.35	0.44	0.08	0.07
(1xi,3xi)-1,2,3,4-Tetrahydro-1-methyl-beta-carboline-3-carboxylic acid	0.35	0.05	0.01	0.01
Capsiate	0.53	0.28	0.48	0.43
2,3-dinor-6-oxoprostaglandin F1alpha	0.26	0.03	0.31	0.26
5Z-7-Oxozeaenol	0.25	0.14	0.10	0.14
Adouetine X	0.40	0.58	0.64	0.40
Pelubiprofen	0.24	0.37	0.00	0.00
Daumone	0.20	0.03	0.70	0.79
Hydroxymethyl cimetidine	0.25	0.29	0.87	0.91
3-Oxohexanoic acid	0.29	0.48	0.30	0.30
2-ISOPRENYL-3-HYDROXY-5-METHYL-a-PYRONE	0.23	0.95	0.67	0.78
1-Octen-3-yl glucoside	0.84	0.09	0.00	0.00
10-(2,3-Dihydroxypropoxy)-10-oxodecanoic acid	0.28	0.40	0.50	0.41
5-Hydroxyvalproic acid	0.65	0.04	0.09	0.05
Coniferaldehyde	0.27	0.33	0.64	0.66
3-Hydroxysebacic Acid	0.26	0.73	0.76	0.73
6-(2-Hydroxyethoxy)-6-oxohexanoic acid	0.51	0.26	0.99	0.98
Gamma-Glutamyl-beta-aminopropiononitrile	0.24	0.00	0.00	0.00
Oxyphencyclimine	0.32	0.27	0.69	0.68
Saccharin	0.22	0.91	0.71	0.93
Octa-3,5-dienedioylcarnitine	0.23	0.03	0.08	0.08
5-Heptyltetrahydro-2-oxo-3-furancarboxylic acid	0.36	0.93	0.42	0.35
Nateglinide	0.34	0.23	0.83	0.92
Phenazine	0.21	0.52	0.56	0.57
Pantothenic Acid	0.30	0.26	0.08	0.06
Histidylcysteine	0.22	0.01	0.01	0.00
DL-Allylglycine	0.21	0.00	0.00	0.00
Isopropyl beta-D-glucoside	0.63	0.40	0.06	0.04
5-(4-Carboxybutylperoxy)pentanoic acid	0.30	0.09	0.31	0.24
Atorvastatin	0.26	0.06	0.00	0.00
N-(2-Furoyl)glycine	0.34	0.98	0.24	0.28
Sarmentosin	0.25	0.07	0.01	0.01
20-Hydroxy-leukotriene E4	0.80	0.00	0.01	0.00
6"-p-Coumaroylprunin	0.56	0.71	0.71	0.76
(Z)-3-Oxo-2-(2-pentenyl)-1-cyclopenteneacetic acid	0.27	0.15	0.83	0.66

(4S,8R)-8,9-Dihydroxy-p-menth-1(6)-en-2-one	0.33	0.39	0.87	0.92
DTDP-4-acetamido-4,6-dideoxy-D-galactose	0.87	0.00	0.00	0.00
4-((6-Methoxyquinolin-8-yl)amino)pentanoic acid	0.38	0.93	0.31	0.30
Aspartame	0.21	0.18	0.36	0.49
Semilepidinoside B	0.24	0.00	0.00	0.00
Tenuazonic acid	0.23	0.01	0.00	0.00
LysoPE(18:2(9Z,12Z)/0:0)	0.30	0.14	0.45	0.44
Serotonin	0.49	0.16	0.38	0.38
Kaempferol 3-rhamnoside 4'-xyloside	0.23	0.36	0.78	0.89
6-Hydroxynicotinic Acid	0.23	0.57	0.40	0.45
Cochinchinenin	0.32	0.00	0.00	0.00
3-Carbamoyl-2-phenylpropionic acid	0.27	0.13	0.04	0.03
Gravacridonetriol glucoside	0.37	0.08	0.07	0.08
Pteroic acid	0.32	0.00	0.04	0.02
3-[[(2S)-2,4-Dihydroxy-3,3-dimethylbutanoyl]amino]propanoic acid	0.30	0.23	0.10	0.07
N-(3,5-Dichloro-4-hydroxyphenyl)-2-hydroxybenzamide	0.34	0.68	0.97	0.93

Table S9. Genetic associations with 121 rumen heritable metabolites as expourse factor

Metabolite feature	SNP	EA exposure	OA exposure	EA outcome	OA outcome	eaf exposure	beta exposure	beta outcome	pval outcome
Cochinchinenin	10:27553874	A	T	T	A	0.10	-0.34	-0.02	0.94
Cochinchinenin	10:27554081	C	T	T	C	0.10	-0.34	-0.14	0.63
Cochinchinenin	10:27554851	G	A	A	G	0.08	-0.41	-0.20	0.51
Cochinchinenin	10:27554870	A	G	G	A	0.08	-0.41	-0.15	0.61
Cochinchinenin	10:27823770	C	T	T	C	0.09	-0.38	-0.09	0.79
Cochinchinenin	10:31381073	A	G	G	A	0.08	-0.37	0.11	0.69
Cochinchinenin	10:31461378	G	A	A	G	0.10	-0.36	0.10	0.71
Cochinchinenin	10:31490581	T	C	C	T	0.09	-0.32	-0.30	0.29
Cochinchinenin	10:92903316	G	A	A	G	0.15	-0.27	0.17	0.48
Cochinchinenin	10:99409559	A	G	G	A	0.10	-0.38	0.50	0.12
Cochinchinenin	10:99409563	A	G	G	A	0.10	-0.38	0.50	0.12
Cochinchinenin	10:99409689	G	A	A	G	0.09	-0.40	1.13	0.00
Cochinchinenin	11:22692698	T	C	C	T	0.06	-0.47	-0.60	0.09
Cochinchinenin	11:31230929	T	C	C	T	0.10	-0.34	0.00	1.00
Cochinchinenin	11:37001017	A	T	T	A	0.11	-0.32	-0.61	0.05
Cochinchinenin	11:38749715	C	T	T	C	0.08	-0.39	-0.01	0.97
Cochinchinenin	12:61191655	A	G	G	A	0.11	-0.32	-0.52	0.21
Cochinchinenin	12:62281020	A	T	T	A	0.14	-0.33	0.48	0.08
Cochinchinenin	13:31958991	G	A	A	G	0.07	-0.47	0.09	0.78
Cochinchinenin	13:37780738	T	G	G	T	0.10	-0.45	0.19	0.49
Cochinchinenin	13:38088333	G	C	C	G	0.07	-0.41	0.05	0.87
Cochinchinenin	13:69557480	G	A	A	G	0.12	-0.34	0.12	0.67
Cochinchinenin	13:69863173	T	C	C	T	0.07	-0.48	0.20	0.61
Cochinchinenin	13:69870750	A	G	G	A	0.08	-0.40	0.38	0.21
Cochinchinenin	13:69870905	G	A	A	G	0.06	-0.41	0.53	0.16
Cochinchinenin	13:69871059	A	G	G	A	0.06	-0.46	0.33	0.31
Cochinchinenin	13:69873477	G	A	A	G	0.05	-0.48	0.17	0.66
Cochinchinenin	13:69886065	C	T	T	C	0.07	-0.40	0.38	0.21
Cochinchinenin	13:69937085	T	C	C	T	0.06	-0.49	0.21	0.52
Cochinchinenin	13:70139806	C	A	A	C	0.06	-0.44	-0.36	0.32
Cochinchinenin	13:70143963	C	T	T	C	0.09	-0.32	0.08	0.82
Cochinchinenin	13:70180271	G	T	T	G	0.07	-0.37	0.06	0.88
Cochinchinenin	13:70180274	A	G	G	A	0.07	-0.37	-0.14	0.67
Cochinchinenin	13:70303721	T	A	A	T	0.06	-0.48	0.38	0.22
Cochinchinenin	13:70550719	T	G	G	T	0.07	-0.46	0.79	0.08
Cochinchinenin	13:74892859	G	A	A	G	0.06	-0.48	-0.11	0.75
Cochinchinenin	13:77543231	T	G	G	T	0.08	-0.41	-0.30	0.44
Cochinchinenin	13:77807665	C	T	T	C	0.09	-0.36	-0.26	0.54
Cochinchinenin	14:71184353	G	T	T	G	0.09	-0.43	0.05	0.87
Cochinchinenin	14:71412620	G	A	A	G	0.07	-0.41	-0.37	0.31
Cochinchinenin	14:81476798	G	T	T	G	0.09	-0.36	-0.15	0.62

Cochinchinenin	14:81697048	C	T	T	C	0.06	-0.50	0.47	0.24
Cochinchinenin	14:81698652	G	A	A	G	0.06	-0.40	0.25	0.49
Cochinchinenin	15:46231060	A	T	T	A	0.07	-0.44	-0.30	0.34
Cochinchinenin	15:46231068	A	C	C	A	0.06	-0.47	-0.31	0.37
Cochinchinenin	16:80952768	G	C	C	G	0.09	-0.37	-0.53	0.14
Cochinchinenin	16:9248219	A	C	C	A	0.05	-0.46	-0.04	0.90
Cochinchinenin	17:51954972	C	T	T	C	0.13	-0.34	-0.48	0.08
Cochinchinenin	17:51954991	A	G	G	A	0.11	-0.34	-0.41	0.15
Cochinchinenin	17:6018764	T	G	G	T	0.07	-0.44	0.12	0.74
Cochinchinenin	17:6048558	T	C	C	T	0.08	-0.39	-0.61	0.09
Cochinchinenin	17:6061328	C	T	T	C	0.07	-0.46	-0.62	0.07
Cochinchinenin	17:61268088	G	A	A	G	0.08	-0.40	-0.42	0.11
Cochinchinenin	17:64717768	T	C	C	T	0.07	-0.49	-0.08	0.79
Cochinchinenin	17:65758081	A	G	G	A	0.07	-0.45	-0.05	0.87
Cochinchinenin	17:65788389	C	T	T	C	0.10	-0.39	-0.12	0.71
Cochinchinenin	17:65809688	G	A	A	G	0.08	-0.39	0.15	0.60
Cochinchinenin	17:65981650	T	G	G	T	0.06	-0.43	-0.01	0.99
Cochinchinenin	17:65982514	C	A	A	C	0.09	-0.38	-0.35	0.24
Cochinchinenin	17:65988472	G	A	A	G	0.08	-0.41	0.09	0.78
Cochinchinenin	17:65995728	A	C	C	A	0.09	-0.37	0.05	0.88
Cochinchinenin	17:66034560	C	T	T	C	0.08	-0.41	-0.31	0.31
Cochinchinenin	17:66214895	T	C	C	T	0.05	-0.45	-0.24	0.46
Cochinchinenin	17:66214909	G	A	A	G	0.06	-0.55	-0.28	0.40
Cochinchinenin	17:66239174	T	C	C	T	0.08	-0.47	-0.18	0.59
Cochinchinenin	17:66240588	G	C	C	G	0.11	-0.39	-0.31	0.30
Cochinchinenin	17:66240661	C	T	T	C	0.12	-0.33	-0.13	0.65
Cochinchinenin	17:66241603	A	G	G	A	0.07	-0.39	-0.17	0.63
Cochinchinenin	17:66241609	C	A	A	C	0.06	-0.41	-0.15	0.68
Cochinchinenin	17:67210700	C	A	A	C	0.07	-0.45	-0.25	0.45
Cochinchinenin	17:68544766	G	A	A	G	0.06	-0.40	-0.66	0.04
Cochinchinenin	17:68737910	A	C	C	A	0.05	-0.51	-0.34	0.39
Cochinchinenin	17:69595132	C	G	G	C	0.06	-0.47	-0.47	0.20
Cochinchinenin	17:69806427	A	G	G	A	0.06	-0.42	-0.26	0.42
Cochinchinenin	17:9809131	T	G	G	T	0.09	-0.39	-0.61	0.08
Cochinchinenin	18:30378296	C	T	T	C	0.06	-0.54	-0.20	0.60
Cochinchinenin	18:31062901	A	G	G	A	0.06	-0.41	-0.57	0.15
Cochinchinenin	18:31066593	T	C	C	T	0.07	-0.42	-0.32	0.36
Cochinchinenin	18:31067858	G	C	C	G	0.07	-0.37	-0.23	0.49
Cochinchinenin	18:31068090	T	C	C	T	0.08	-0.38	-0.11	0.70
Cochinchinenin	18:31257013	G	A	A	G	0.05	-0.53	-0.40	0.27
Cochinchinenin	18:31263997	T	C	C	T	0.05	-0.44	-0.15	0.68
Cochinchinenin	18:31264017	G	T	T	G	0.05	-0.50	-0.47	0.15
Cochinchinenin	18:31264860	T	G	G	T	0.06	-0.44	-0.40	0.28
Cochinchinenin	18:31266430	T	G	G	T	0.05	-0.48	-0.40	0.25

Cochinchinenin	18:31268881	G	A	A	G	0.05	-0.47	-0.61	0.08
Cochinchinenin	18:33154309	A	G	G	A	0.10	-0.37	-0.14	0.62
Cochinchinenin	18:33450057	G	C	C	G	0.05	-0.62	-0.32	0.36
Cochinchinenin	18:9718082	C	T	T	C	0.08	-0.40	-0.50	0.19
Cochinchinenin	18:9973049	G	A	A	G	0.06	-0.42	-0.52	0.09
Cochinchinenin	19:1451603	T	A	A	T	0.05	-0.69	-0.27	0.46
Cochinchinenin	19:1694500	C	T	T	C	0.08	-0.53	-0.83	0.01
Cochinchinenin	19:1788908	C	T	T	C	0.07	-0.47	-0.42	0.30
Cochinchinenin	19:1957200	T	A	A	T	0.11	-0.40	-0.67	0.03
Cochinchinenin	19:19625907	T	C	C	T	0.06	-0.48	-0.02	0.96
Cochinchinenin	19:19701583	A	T	T	A	0.07	-0.42	0.45	0.27
Cochinchinenin	19:19907684	C	T	T	C	0.07	-0.38	-0.33	0.28
Cochinchinenin	19:21297943	T	C	C	T	0.09	-0.36	0.22	0.52
Cochinchinenin	19:22372213	G	A	A	G	0.05	-0.66	0.08	0.82
Cochinchinenin	19:30883299	C	T	T	C	0.09	-0.31	0.41	0.14
Cochinchinenin	19:30897387	C	T	T	C	0.08	-0.36	0.01	0.97
Cochinchinenin	19:340447	T	C	C	T	0.08	-0.37	-0.17	0.53
Cochinchinenin	19:3585905	G	A	A	G	0.05	-0.56	-0.23	0.53
Cochinchinenin	19:3785539	G	A	A	G	0.05	-0.60	0.44	0.21
Cochinchinenin	19:417406	A	G	G	A	0.06	-0.48	-0.48	0.15
Cochinchinenin	19:6096274	T	C	C	T	0.09	-0.43	-0.47	0.14
Cochinchinenin	19:6097957	T	C	C	T	0.09	-0.35	-0.09	0.79
Cochinchinenin	19:8197927	T	G	G	T	0.05	-0.58	-0.61	0.13
Cochinchinenin	19:8232513	C	T	T	C	0.11	-0.34	-0.30	0.30
Cochinchinenin	19:8362907	G	A	A	G	0.12	-0.31	0.18	0.48
Cochinchinenin	2:11611087	G	C	C	G	0.07	-0.42	-0.73	0.05
Cochinchinenin	2:86230028	A	G	G	A	0.05	-0.52	-0.31	0.43
Cochinchinenin	20:33312140	T	C	C	T	0.06	-0.43	0.43	0.21
Cochinchinenin	20:46990829	C	T	T	C	0.08	-0.44	0.12	0.76
Cochinchinenin	21:17727875	A	G	G	A	0.07	-0.39	0.24	0.54
Cochinchinenin	21:55819096	A	G	G	A	0.07	-0.43	0.08	0.83
Cochinchinenin	21:6730029	C	T	T	C	0.10	-0.33	0.12	0.69
Cochinchinenin	21:6730034	T	C	C	T	0.10	-0.37	0.12	0.69
Cochinchinenin	21:6730079	G	A	A	G	0.11	-0.31	0.01	0.96
Cochinchinenin	21:6916012	T	A	A	T	0.08	-0.36	-0.32	0.29
Cochinchinenin	21:694922	G	A	A	G	0.07	-0.47	-0.44	0.28
Cochinchinenin	22:45903062	A	G	G	A	0.14	-0.31	-0.22	0.41
Cochinchinenin	22:4680835	T	G	G	T	0.05	-0.48	-0.10	0.79
Cochinchinenin	22:48829615	T	G	G	T	0.08	-0.39	0.01	0.97
Cochinchinenin	22:56250365	G	A	A	G	0.10	-0.36	-0.02	0.94
Cochinchinenin	22:56251819	G	A	A	G	0.13	-0.30	-0.42	0.13
Cochinchinenin	22:56562631	C	T	T	C	0.11	-0.33	-0.17	0.56
Cochinchinenin	22:58118435	G	C	C	G	0.09	-0.40	0.15	0.73
Cochinchinenin	24:10010120	C	A	A	C	0.14	-0.33	-0.25	0.28

Cochinchinenin	24:10766987	G	A	A	G	0.12	-0.35	-0.24	0.34
Cochinchinenin	24:10791282	A	T	T	A	0.15	-0.30	-0.17	0.45
Cochinchinenin	24:10791439	A	G	G	A	0.15	-0.31	-0.29	0.27
Cochinchinenin	24:10791448	G	A	A	G	0.15	-0.30	-0.29	0.26
Cochinchinenin	24:10795492	G	A	A	G	0.17	-0.27	-0.38	0.17
Cochinchinenin	24:11060721	C	A	A	C	0.06	-0.58	0.12	0.77
Cochinchinenin	24:11867949	T	C	C	T	0.09	-0.40	-0.70	0.06
Cochinchinenin	24:12261045	C	T	T	C	0.06	-0.44	0.21	0.49
Cochinchinenin	24:5907929	G	T	T	G	0.06	-0.47	-0.52	0.13
Cochinchinenin	24:5963214	G	A	A	G	0.07	-0.40	-0.33	0.32
Cochinchinenin	24:7312065	T	C	C	T	0.13	-0.30	0.18	0.54
Cochinchinenin	24:7334624	G	A	A	G	0.10	-0.35	0.08	0.78
Cochinchinenin	24:7334688	G	A	A	G	0.09	-0.36	-0.22	0.43
Cochinchinenin	24:7336532	T	C	C	T	0.08	-0.39	0.00	0.99
Cochinchinenin	24:7361836	G	A	A	G	0.09	-0.37	0.05	0.87
Cochinchinenin	24:8867426	G	T	T	G	0.06	-0.47	-0.19	0.62
Cochinchinenin	25:10921085	G	A	A	G	0.08	-0.44	-0.43	0.21
Cochinchinenin	25:4477754	C	T	T	C	0.17	-0.29	-0.11	0.60
Cochinchinenin	25:4508860	C	T	T	C	0.18	-0.26	-0.12	0.55
Cochinchinenin	25:4724226	C	T	T	C	0.16	-0.28	0.11	0.64
Cochinchinenin	25:7137658	A	G	G	A	0.08	-0.43	0.53	0.14
Cochinchinenin	25:972392	C	T	T	C	0.09	-0.40	0.02	0.94
Cochinchinenin	27:32809330	A	T	T	A	0.06	-0.52	-0.54	0.12
Cochinchinenin	27:33559630	A	G	G	A	0.05	-0.48	0.02	0.95
Cochinchinenin	27:35856579	T	C	C	T	0.05	-0.54	-0.84	0.03
Cochinchinenin	27:35858666	T	G	G	T	0.06	-0.47	-0.97	0.01
Cochinchinenin	27:44239543	T	C	C	T	0.05	-0.50	0.09	0.83
Cochinchinenin	3:4887740	G	A	A	G	0.13	-0.32	-0.04	0.89
Cochinchinenin	3:63247308	T	C	C	T	0.05	-0.60	-0.57	0.27
Cochinchinenin	3:73951900	C	A	A	C	0.06	-0.50	-0.80	0.06
Cochinchinenin	3:88494411	T	C	C	T	0.07	-0.43	-0.11	0.74
Cochinchinenin	30:117657119	C	G	G	C	0.08	-0.41	0.50	0.22
Cochinchinenin	30:127114432	G	A	A	G	0.19	-0.26	-0.36	0.14
Cochinchinenin	30:16346111	A	G	G	A	0.06	-0.45	-0.48	0.24
Cochinchinenin	30:18339972	A	C	C	A	0.12	-0.35	0.00	1.00
Cochinchinenin	4:111230521	A	C	C	A	0.06	-0.42	-0.16	0.66
Cochinchinenin	4:45207224	C	T	T	C	0.05	-0.50	0.13	0.75
Cochinchinenin	4:45281221	T	C	C	T	0.05	-0.45	0.15	0.67
Cochinchinenin	4:45281245	A	G	G	A	0.05	-0.44	0.23	0.55
Cochinchinenin	4:48153613	A	T	T	A	0.06	-0.47	-0.08	0.83
Cochinchinenin	4:52399772	C	A	A	C	0.10	-0.32	-0.18	0.60
Cochinchinenin	5:107736697	A	G	G	A	0.10	-0.34	-0.36	0.31
Cochinchinenin	5:17065542	A	C	C	A	0.05	-0.54	-0.61	0.06
Cochinchinenin	5:17136655	T	C	C	T	0.06	-0.50	-0.64	0.09

Cochinchinenin	5:22281654	A	T	T	A	0.06	-0.61	0.00	0.99
Cochinchinenin	5:22897331	T	C	C	T	0.08	-0.40	0.27	0.43
Cochinchinenin	5:32106090	C	T	T	C	0.08	-0.42	0.32	0.30
Cochinchinenin	5:36242094	A	T	T	A	0.06	-0.54	0.19	0.61
Cochinchinenin	5:88848537	C	T	T	C	0.10	-0.33	0.00	0.99
Cochinchinenin	5:91654726	A	G	G	A	0.09	-0.39	-0.08	0.83
Cochinchinenin	6:18761257	G	A	A	G	0.06	-0.42	-0.25	0.44
Cochinchinenin	6:47725788	T	C	C	T	0.09	-0.34	-0.33	0.45
Cochinchinenin	6:48732814	A	G	G	A	0.09	-0.37	-0.37	0.32
Cochinchinenin	6:58084482	A	G	G	A	0.06	-0.42	-0.44	0.25
Cochinchinenin	6:59518168	G	A	A	G	0.08	-0.43	-0.70	0.07
Cochinchinenin	6:96232301	G	C	C	G	0.07	-0.40	-0.22	0.47
Cochinchinenin	8:112694618	T	C	C	T	0.07	-0.42	-0.11	0.73
Cochinchinenin	8:36468515	T	G	G	T	0.08	-0.40	0.15	0.67
Cochinchinenin	8:36583994	C	T	T	C	0.08	-0.46	-0.55	0.10
Cochinchinenin	8:50046402	G	A	A	G	0.06	-0.49	-0.04	0.93
Cochinchinenin	8:50049707	G	A	A	G	0.06	-0.46	0.35	0.39
Cochinchinenin	8:50153636	T	C	C	T	0.06	-0.51	-0.16	0.68
Cochinchinenin	8:85634616	T	C	C	T	0.07	-0.38	0.07	0.87
Cochinchinenin	8:85634639	T	C	C	T	0.07	-0.37	0.07	0.86
Cochinchinenin	8:89985718	A	G	G	A	0.07	-0.43	-0.12	0.68
Cochinchinenin	8:95079340	A	G	G	A	0.07	-0.43	-0.26	0.44
Cochinchinenin	9:1996192	T	C	C	T	0.09	-0.39	-0.39	0.23
Cochinchinenin	9:246798	T	A	A	T	0.11	-0.35	-0.62	0.04
Cochinchinenin	9:36920654	C	T	T	C	0.10	-0.38	-0.38	0.23
Cochinchinenin	9:47042792	A	T	T	A	0.13	-0.32	-0.37	0.15
Cochinchinenin	9:50991087	G	T	T	G	0.06	-0.43	-0.32	0.46
Cochinchinenin	9:52393702	G	A	A	G	0.09	-0.33	0.11	0.72
Cochinchinenin	9:52662634	G	A	A	G	0.07	-0.42	0.21	0.61
Cochinchinenin	9:54867071	A	G	G	A	0.14	-0.30	0.23	0.37
Cochinchinenin	9:7230714	A	C	C	A	0.07	-0.41	-0.45	0.16
4-Hydroxy-3-polyprenylbenzoate	10:22748762	C	T	T	C	0.14	0.15	-0.10	0.73
4-Hydroxy-3-polyprenylbenzoate	12:72588164	G	A	A	G	0.12	0.16	-0.15	0.64
4-Hydroxy-3-polyprenylbenzoate	12:72588173	C	T	T	C	0.10	0.17	-0.52	0.14
4-Hydroxy-3-polyprenylbenzoate	12:72588178	C	T	T	C	0.10	0.18	-0.37	0.31
4-Hydroxy-3-polyprenylbenzoate	13:11068632	A	G	G	A	0.08	0.19	-0.10	0.78
4-Hydroxy-3-polyprenylbenzoate	13:11431274	G	A	A	G	0.06	0.23	0.82	0.06
4-Hydroxy-3-polyprenylbenzoate	13:20779369	T	A	A	T	0.07	0.20	-0.62	0.09
4-Hydroxy-3-polyprenylbenzoate	13:67734493	G	A	A	G	0.06	0.23	-0.34	0.38
4-Hydroxy-3-polyprenylbenzoate	13:71537085	T	C	C	T	0.11	0.15	0.22	0.50
4-Hydroxy-3-polyprenylbenzoate	13:9139573	C	T	T	C	0.06	0.19	-0.41	0.34
4-Hydroxy-3-polyprenylbenzoate	15:74070732	G	A	A	G	0.07	0.20	0.12	0.68
4-Hydroxy-3-polyprenylbenzoate	15:84258400	C	T	T	C	0.14	0.16	0.16	0.59
4-Hydroxy-3-polyprenylbenzoate	17:72311301	C	T	T	C	0.07	0.19	0.47	0.07

4-Hydroxy-3-polyprenylbenzoate	20:12828858	C	T	T	C	0.13	-0.15	-0.32	0.24
4-Hydroxy-3-polyprenylbenzoate	20:24853178	G	A	A	G	0.11	0.16	0.22	0.46
4-Hydroxy-3-polyprenylbenzoate	20:25047656	A	C	C	A	0.09	0.19	0.09	0.81
4-Hydroxy-3-polyprenylbenzoate	20:25207985	T	G	G	T	0.10	0.19	0.09	0.84
4-Hydroxy-3-polyprenylbenzoate	20:25256263	C	T	T	C	0.08	0.17	0.28	0.47
4-Hydroxy-3-polyprenylbenzoate	20:25748757	C	T	T	C	0.12	0.14	0.49	0.13
4-Hydroxy-3-polyprenylbenzoate	20:47322831	C	A	A	C	0.08	0.16	-0.61	0.11
4-Hydroxy-3-polyprenylbenzoate	23:16922	T	C	C	T	0.07	0.27	0.41	0.35
4-Hydroxy-3-polyprenylbenzoate	23:3631024	G	A	A	G	0.07	-0.18	0.12	0.74
4-Hydroxy-3-polyprenylbenzoate	26:43714613	C	T	T	C	0.09	-0.18	-0.53	0.14
4-Hydroxy-3-polyprenylbenzoate	27:115866	A	C	C	A	0.11	0.20	0.63	0.05
4-Hydroxy-3-polyprenylbenzoate	27:115881	C	T	T	C	0.13	0.17	0.08	0.79
4-Hydroxy-3-polyprenylbenzoate	27:7069850	T	A	A	T	0.10	0.20	-0.21	0.50
4-Hydroxy-3-polyprenylbenzoate	28:21646634	T	C	C	T	0.07	0.17	-0.06	0.84
4-Hydroxy-3-polyprenylbenzoate	28:21776905	A	G	G	A	0.08	0.19	-0.01	0.98
4-Hydroxy-3-polyprenylbenzoate	29:583806	T	C	C	T	0.08	0.20	-0.33	0.42
4-Hydroxy-3-polyprenylbenzoate	30:19012903	G	A	A	G	0.10	0.19	0.19	0.53
4-Hydroxy-3-polyprenylbenzoate	30:38390025	C	T	T	C	0.08	0.19	0.19	0.57
4-Hydroxy-3-polyprenylbenzoate	30:38390026	A	G	G	A	0.08	0.19	0.19	0.57
4-Hydroxy-3-polyprenylbenzoate	30:38390059	T	C	C	T	0.08	0.20	0.91	0.01
4-Hydroxy-3-polyprenylbenzoate	30:38390060	G	A	A	G	0.08	0.20	0.91	0.01
4-Hydroxy-3-polyprenylbenzoate	30:38390062	T	C	C	T	0.08	0.20	0.92	0.01
4-Hydroxy-3-polyprenylbenzoate	4:105607043	C	A	A	C	0.07	0.22	0.41	0.27
4-Hydroxy-3-polyprenylbenzoate	4:105607054	T	C	C	T	0.07	0.21	0.10	0.82
4-Hydroxy-3-polyprenylbenzoate	4:105607055	C	T	T	C	0.07	0.21	0.23	0.60
4-Hydroxy-3-polyprenylbenzoate	5:59745953	T	C	C	T	0.06	0.24	-0.11	0.81
4-Hydroxy-3-polyprenylbenzoate	9:100212477	C	T	T	C	0.12	0.15	0.03	0.88
4-Hydroxy-3-polyprenylbenzoate	9:99066480	T	G	G	T	0.17	-0.12	0.18	0.53
1,5-Isoquinolinediol	1:143088210	C	T	T	C	0.07	-0.19	-0.13	0.67
1,5-Isoquinolinediol	1:23704240	C	G	G	C	0.11	-0.16	-0.47	0.23
1,5-Isoquinolinediol	1:23736358	C	T	T	C	0.09	-0.19	-0.52	0.16
1,5-Isoquinolinediol	1:23769920	T	C	C	T	0.10	-0.19	-0.16	0.59
1,5-Isoquinolinediol	1:23792457	C	T	T	C	0.10	-0.18	-0.53	0.19
1,5-Isoquinolinediol	1:23826005	C	T	T	C	0.12	-0.16	-0.29	0.43
1,5-Isoquinolinediol	1:23826434	A	T	T	A	0.10	-0.17	-0.40	0.23
1,5-Isoquinolinediol	1:23863965	G	A	A	G	0.10	-0.18	-0.21	0.47
1,5-Isoquinolinediol	1:23905964	C	G	G	C	0.11	-0.17	-0.29	0.43
1,5-Isoquinolinediol	1:24165535	T	A	A	T	0.15	-0.14	-0.35	0.22
1,5-Isoquinolinediol	1:24213920	A	G	G	A	0.10	-0.17	-0.42	0.23
1,5-Isoquinolinediol	1:24311148	A	G	G	A	0.10	-0.18	-0.43	0.19
1,5-Isoquinolinediol	1:24455158	C	T	T	C	0.09	-0.19	0.09	0.84
1,5-Isoquinolinediol	13:3555901	T	C	C	T	0.15	-0.13	0.06	0.85
1,5-Isoquinolinediol	13:70139806	C	A	A	C	0.06	-0.21	-0.36	0.32
1,5-Isoquinolinediol	13:70143963	C	T	T	C	0.09	-0.17	0.08	0.82

1,5-Isoquinolinediol	13:77807665	C	T	T	C	0.09	-0.18	-0.26	0.54
1,5-Isoquinolinediol	16:76743378	C	T	T	C	0.15	-0.16	-0.53	0.07
1,5-Isoquinolinediol	20:65381928	T	C	C	T	0.16	-0.14	-0.39	0.13
1,5-Isoquinolinediol	22:56260867	A	G	G	A	0.16	-0.14	-0.52	0.08
1,5-Isoquinolinediol	29:39247093	A	G	G	A	0.09	-0.19	0.07	0.85
1,5-Isoquinolinediol	30:1018530	G	A	A	G	0.07	-0.19	0.25	0.49
1,5-Isoquinolinediol	30:110204969	G	T	T	G	0.05	-0.23	-0.25	0.44
1,5-Isoquinolinediol	8:60857328	A	G	G	A	0.07	-0.23	-0.19	0.64
1,5-Isoquinolinediol	9:3756603	G	A	A	G	0.12	-0.16	-0.03	0.92
1,5-Isoquinolinediol	9:66220675	A	G	G	A	0.06	-0.22	-0.39	0.23
1,5-Isoquinolinediol	9:66265068	G	A	A	G	0.07	-0.21	-0.19	0.60
6-Hydroxymelatonin	1:101300826	A	C	C	A	0.12	-0.36	0.18	0.56
6-Hydroxymelatonin	1:107561392	G	T	T	G	0.09	-0.38	0.27	0.36
6-Hydroxymelatonin	1:107573145	G	C	C	G	0.09	-0.39	0.36	0.18
6-Hydroxymelatonin	1:107639311	A	G	G	A	0.07	-0.48	0.67	0.10
6-Hydroxymelatonin	1:107909436	C	T	T	C	0.08	-0.60	0.72	0.04
6-Hydroxymelatonin	1:122630631	C	G	G	C	0.05	-0.54	0.52	0.17
6-Hydroxymelatonin	1:130412815	A	T	T	A	0.15	-0.37	0.12	0.67
6-Hydroxymelatonin	1:142424683	T	A	A	T	0.06	-0.48	0.97	0.00
6-Hydroxymelatonin	1:152551961	T	C	C	T	0.07	-0.44	0.86	0.04
6-Hydroxymelatonin	1:15692062	A	T	T	A	0.08	-0.41	-0.35	0.33
6-Hydroxymelatonin	1:44312680	G	A	A	G	0.06	-0.47	0.10	0.78
6-Hydroxymelatonin	1:59504033	T	C	C	T	0.14	-0.37	0.63	0.01
6-Hydroxymelatonin	1:62277247	C	T	T	C	0.12	-0.37	-0.12	0.68
6-Hydroxymelatonin	1:74955112	C	T	T	C	0.07	-0.67	0.71	0.05
6-Hydroxymelatonin	10:19161937	T	C	C	T	0.09	-0.42	0.19	0.58
6-Hydroxymelatonin	10:30905985	C	T	T	C	0.13	-0.35	0.55	0.06
6-Hydroxymelatonin	10:37427521	C	A	A	C	0.06	-0.53	0.10	0.74
6-Hydroxymelatonin	10:38327635	A	G	G	A	0.09	-0.38	0.04	0.91
6-Hydroxymelatonin	10:55896580	A	G	G	A	0.11	-0.38	0.25	0.42
6-Hydroxymelatonin	10:67561213	A	G	G	A	0.09	-0.50	-0.09	0.76
6-Hydroxymelatonin	10:68977487	A	G	G	A	0.09	-0.39	0.21	0.47
6-Hydroxymelatonin	10:69525000	G	A	A	G	0.13	-0.34	0.47	0.10
6-Hydroxymelatonin	10:73600059	G	A	A	G	0.05	-0.64	0.96	0.02
6-Hydroxymelatonin	10:77053816	A	T	T	A	0.08	-0.46	-0.31	0.39
6-Hydroxymelatonin	10:8612917	A	G	G	A	0.05	-0.47	-0.27	0.46
6-Hydroxymelatonin	10:8612981	C	T	T	C	0.06	-0.49	-0.45	0.18
6-Hydroxymelatonin	10:8612993	A	G	G	A	0.06	-0.49	-0.37	0.28
6-Hydroxymelatonin	11:103910659	C	G	G	C	0.07	-0.52	0.10	0.78
6-Hydroxymelatonin	11:12919955	T	C	C	T	0.06	-0.63	-0.19	0.59
6-Hydroxymelatonin	11:13114553	T	C	C	T	0.07	-0.53	0.47	0.28
6-Hydroxymelatonin	11:13166283	G	C	C	G	0.10	-0.42	-0.18	0.60
6-Hydroxymelatonin	11:13230013	T	G	G	T	0.10	-0.41	0.39	0.25
6-Hydroxymelatonin	11:13394615	G	A	A	G	0.08	-0.43	0.07	0.83

6-Hydroxymelatonin	11:13431111	C	G	G	C	0.07	-0.52	0.29	0.42
6-Hydroxymelatonin	11:16907940	C	T	T	C	0.13	-0.38	0.27	0.28
6-Hydroxymelatonin	11:21036317	T	G	G	T	0.11	-0.40	-0.14	0.63
6-Hydroxymelatonin	11:25902747	A	T	T	A	0.06	-0.48	0.05	0.89
6-Hydroxymelatonin	11:59567182	T	C	C	T	0.15	-0.35	-0.19	0.52
6-Hydroxymelatonin	11:63967640	G	A	A	G	0.12	-0.38	0.40	0.12
6-Hydroxymelatonin	11:66873402	G	A	A	G	0.07	-0.42	-0.10	0.76
6-Hydroxymelatonin	11:72363311	G	A	A	G	0.08	-0.46	0.47	0.23
6-Hydroxymelatonin	11:89933288	G	A	A	G	0.07	-0.48	0.31	0.30
6-Hydroxymelatonin	11:92350679	T	C	C	T	0.07	-0.55	0.49	0.15
6-Hydroxymelatonin	11:93162801	C	T	T	C	0.06	-0.55	0.19	0.60
6-Hydroxymelatonin	11:96048824	T	C	C	T	0.06	-0.51	0.15	0.68
6-Hydroxymelatonin	12:11944364	G	T	T	G	0.07	-0.49	0.57	0.11
6-Hydroxymelatonin	12:12926372	G	A	A	G	0.07	-0.43	0.57	0.13
6-Hydroxymelatonin	12:13503147	T	C	C	T	0.06	-0.50	0.05	0.88
6-Hydroxymelatonin	12:13503148	G	A	A	G	0.06	-0.50	0.05	0.88
6-Hydroxymelatonin	12:3174249	A	G	G	A	0.09	-0.44	-0.62	0.05
6-Hydroxymelatonin	12:3180625	T	C	C	T	0.12	-0.37	-0.59	0.02
6-Hydroxymelatonin	12:3222605	A	G	G	A	0.09	-0.41	-0.48	0.13
6-Hydroxymelatonin	12:37424384	A	T	T	A	0.06	-0.44	0.34	0.25
6-Hydroxymelatonin	12:50825346	A	G	G	A	0.06	-0.52	0.53	0.08
6-Hydroxymelatonin	12:54457358	C	T	T	C	0.06	-0.57	-0.24	0.47
6-Hydroxymelatonin	12:6840156	T	G	G	T	0.07	-0.48	0.28	0.38
6-Hydroxymelatonin	12:6957557	T	C	C	T	0.09	-0.40	-0.13	0.65
6-Hydroxymelatonin	12:72331253	G	T	T	G	0.05	-0.51	0.25	0.39
6-Hydroxymelatonin	12:72374102	A	T	T	A	0.09	-0.49	0.16	0.59
6-Hydroxymelatonin	12:72374110	C	A	A	C	0.08	-0.52	0.10	0.75
6-Hydroxymelatonin	12:76444005	C	T	T	C	0.07	-0.47	0.25	0.44
6-Hydroxymelatonin	12:85251957	C	T	T	C	0.15	-0.32	0.11	0.59
6-Hydroxymelatonin	13:37848874	G	C	C	G	0.06	-0.51	0.24	0.59
6-Hydroxymelatonin	14:126466	C	T	T	C	0.07	-0.56	0.13	0.74
6-Hydroxymelatonin	14:70702937	C	T	T	C	0.05	-0.59	0.20	0.66
6-Hydroxymelatonin	15:11224123	C	G	G	C	0.08	-0.46	-0.09	0.78
6-Hydroxymelatonin	15:17753427	A	G	G	A	0.12	-0.37	0.44	0.12
6-Hydroxymelatonin	15:17753431	G	A	A	G	0.12	-0.38	0.51	0.08
6-Hydroxymelatonin	15:3464433	T	C	C	T	0.10	-0.37	0.89	0.00
6-Hydroxymelatonin	15:3464476	A	G	G	A	0.10	-0.41	0.55	0.04
6-Hydroxymelatonin	15:3464510	T	C	C	T	0.12	-0.41	0.56	0.04
6-Hydroxymelatonin	15:3464602	T	C	C	T	0.11	-0.33	0.39	0.16
6-Hydroxymelatonin	15:3464672	G	A	A	G	0.11	-0.35	0.48	0.07
6-Hydroxymelatonin	15:3464723	A	G	G	A	0.11	-0.40	0.59	0.03
6-Hydroxymelatonin	15:3464858	T	A	A	T	0.12	-0.33	0.47	0.09
6-Hydroxymelatonin	15:3465274	A	G	G	A	0.08	-0.50	0.71	0.01
6-Hydroxymelatonin	15:3481096	C	T	T	C	0.12	-0.35	0.60	0.03

6-Hydroxymelatonin	15:38426601	C	T	T	C	0.05	-0.60	0.39	0.37
6-Hydroxymelatonin	15:40168946	C	T	T	C	0.05	-0.52	0.39	0.24
6-Hydroxymelatonin	15:47038920	A	C	C	A	0.09	-0.44	-0.08	0.78
6-Hydroxymelatonin	15:66604279	C	T	T	C	0.11	-0.34	-0.34	0.29
6-Hydroxymelatonin	15:77692866	C	T	T	C	0.08	-0.45	0.12	0.69
6-Hydroxymelatonin	16:23544281	C	T	T	C	0.18	-0.25	0.28	0.21
6-Hydroxymelatonin	16:24596351	C	G	G	C	0.11	-0.40	0.07	0.79
6-Hydroxymelatonin	16:71065597	C	G	G	C	0.06	-0.50	0.05	0.89
6-Hydroxymelatonin	16:75226297	C	G	G	C	0.08	-0.44	0.03	0.93
6-Hydroxymelatonin	16:75228940	T	C	C	T	0.14	-0.40	0.04	0.87
6-Hydroxymelatonin	16:75229446	A	C	C	A	0.17	-0.33	-0.11	0.68
6-Hydroxymelatonin	16:75229459	G	A	A	G	0.16	-0.37	-0.14	0.59
6-Hydroxymelatonin	16:75229472	A	G	G	A	0.17	-0.35	-0.09	0.72
6-Hydroxymelatonin	16:75229551	A	G	G	A	0.14	-0.34	0.32	0.24
6-Hydroxymelatonin	16:79880089	C	T	T	C	0.07	-0.39	0.46	0.14
6-Hydroxymelatonin	17:12033424	G	C	C	G	0.06	-0.54	-0.19	0.52
6-Hydroxymelatonin	17:12037094	T	C	C	T	0.07	-0.54	-0.30	0.29
6-Hydroxymelatonin	17:44134377	C	T	T	C	0.09	-0.39	0.58	0.09
6-Hydroxymelatonin	17:4966382	T	C	C	T	0.09	-0.44	-0.09	0.77
6-Hydroxymelatonin	17:4966383	G	T	T	G	0.09	-0.44	-0.06	0.84
6-Hydroxymelatonin	17:4966415	G	C	C	G	0.11	-0.43	0.14	0.65
6-Hydroxymelatonin	17:5548600	G	A	A	G	0.06	-0.48	-0.24	0.51
6-Hydroxymelatonin	17:5574496	A	G	G	A	0.10	-0.39	-0.09	0.73
6-Hydroxymelatonin	18:11505135	T	C	C	T	0.06	-0.52	0.91	0.04
6-Hydroxymelatonin	18:11507674	C	T	T	C	0.07	-0.52	1.00	0.02
6-Hydroxymelatonin	18:11608642	T	G	G	T	0.05	-0.60	0.52	0.17
6-Hydroxymelatonin	18:11608646	C	T	T	C	0.05	-0.60	0.53	0.16
6-Hydroxymelatonin	18:11610859	G	C	C	G	0.06	-0.48	0.48	0.10
6-Hydroxymelatonin	18:11613424	C	T	T	C	0.06	-0.48	0.91	0.01
6-Hydroxymelatonin	18:15944891	A	C	C	A	0.08	-0.40	0.39	0.25
6-Hydroxymelatonin	18:15950807	C	T	T	C	0.05	-0.59	0.13	0.77
6-Hydroxymelatonin	18:16351282	T	C	C	T	0.06	-0.51	0.47	0.26
6-Hydroxymelatonin	18:16446746	C	T	T	C	0.07	-0.58	0.04	0.90
6-Hydroxymelatonin	18:16469993	T	A	A	T	0.06	-0.49	-0.23	0.55
6-Hydroxymelatonin	18:16469994	T	C	C	T	0.06	-0.48	-0.24	0.54
6-Hydroxymelatonin	18:16488450	G	A	A	G	0.08	-0.43	0.13	0.69
6-Hydroxymelatonin	18:16490118	A	C	C	A	0.06	-0.49	-0.11	0.80
6-Hydroxymelatonin	18:31123298	A	T	T	A	0.06	-0.56	0.53	0.24
6-Hydroxymelatonin	18:31157871	G	T	T	G	0.08	-0.48	0.64	0.08
6-Hydroxymelatonin	18:6466934	G	A	A	G	0.07	-0.47	0.75	0.07
6-Hydroxymelatonin	18:9918435	C	T	T	C	0.06	-0.67	0.85	0.02
6-Hydroxymelatonin	19:32677005	A	T	T	A	0.05	-0.64	0.72	0.05
6-Hydroxymelatonin	19:32677183	C	A	A	C	0.07	-0.54	0.24	0.42
6-Hydroxymelatonin	19:32677634	C	T	T	C	0.06	-0.52	0.16	0.66

6-Hydroxymelatonin	19:32682840	T	C	C	T	0.07	-0.52	0.69	0.04
6-Hydroxymelatonin	19:32683040	T	C	C	T	0.06	-0.45	0.69	0.06
6-Hydroxymelatonin	19:32683041	G	A	A	G	0.06	-0.45	0.83	0.03
6-Hydroxymelatonin	19:32683058	G	A	A	G	0.06	-0.50	0.58	0.09
6-Hydroxymelatonin	19:32687831	G	A	A	G	0.06	-0.56	0.71	0.07
6-Hydroxymelatonin	19:32691250	G	A	A	G	0.07	-0.49	0.84	0.02
6-Hydroxymelatonin	19:32691315	G	A	A	G	0.07	-0.47	0.28	0.39
6-Hydroxymelatonin	19:32691378	G	T	T	G	0.08	-0.46	0.53	0.09
6-Hydroxymelatonin	19:32691386	A	G	G	A	0.07	-0.53	0.55	0.08
6-Hydroxymelatonin	19:32693053	C	T	T	C	0.08	-0.43	0.53	0.06
6-Hydroxymelatonin	19:32694044	A	G	G	A	0.07	-0.50	0.52	0.11
6-Hydroxymelatonin	19:32694104	C	T	T	C	0.07	-0.53	0.15	0.66
6-Hydroxymelatonin	19:32694190	G	A	A	G	0.07	-0.45	0.20	0.53
6-Hydroxymelatonin	19:32695171	G	A	A	G	0.10	-0.34	0.60	0.04
6-Hydroxymelatonin	19:32695246	G	C	C	G	0.09	-0.37	0.28	0.27
6-Hydroxymelatonin	19:32695451	G	A	A	G	0.08	-0.46	0.43	0.11
6-Hydroxymelatonin	19:32695614	G	T	T	G	0.08	-0.40	0.61	0.07
6-Hydroxymelatonin	19:32696211	T	G	G	T	0.09	-0.41	0.34	0.31
6-Hydroxymelatonin	19:32696221	A	G	G	A	0.09	-0.41	0.34	0.30
6-Hydroxymelatonin	19:32696232	C	T	T	C	0.09	-0.41	0.17	0.58
6-Hydroxymelatonin	19:32700836	T	G	G	T	0.08	-0.51	0.05	0.86
6-Hydroxymelatonin	19:32704177	A	G	G	A	0.08	-0.44	0.56	0.09
6-Hydroxymelatonin	19:32705059	T	C	C	T	0.07	-0.46	0.66	0.09
6-Hydroxymelatonin	19:7839241	G	C	C	G	0.05	-0.50	0.33	0.34
6-Hydroxymelatonin	19:7840073	C	A	A	C	0.06	-0.59	0.36	0.33
6-Hydroxymelatonin	19:7842483	C	T	T	C	0.05	-0.56	0.07	0.85
6-Hydroxymelatonin	2:101029629	A	C	C	A	0.06	-0.47	-0.25	0.37
6-Hydroxymelatonin	2:101121154	G	T	T	G	0.08	-0.42	-0.06	0.85
6-Hydroxymelatonin	2:101126052	G	C	C	G	0.07	-0.53	-0.27	0.36
6-Hydroxymelatonin	2:101159688	T	C	C	T	0.05	-0.51	-0.23	0.63
6-Hydroxymelatonin	2:101160361	T	C	C	T	0.08	-0.46	-0.27	0.34
6-Hydroxymelatonin	2:101894257	T	C	C	T	0.07	-0.64	-0.14	0.65
6-Hydroxymelatonin	2:101901236	T	A	A	T	0.08	-0.49	0.07	0.82
6-Hydroxymelatonin	2:102152103	A	G	G	A	0.06	-0.61	-0.23	0.50
6-Hydroxymelatonin	2:102331536	T	C	C	T	0.06	-0.55	-0.25	0.43
6-Hydroxymelatonin	2:102841192	A	G	G	A	0.08	-0.47	0.07	0.79
6-Hydroxymelatonin	2:103171717	G	A	A	G	0.06	-0.48	-0.17	0.59
6-Hydroxymelatonin	2:103172001	A	G	G	A	0.06	-0.59	0.08	0.79
6-Hydroxymelatonin	2:103185042	G	A	A	G	0.06	-0.61	0.27	0.42
6-Hydroxymelatonin	2:103223888	A	G	G	A	0.05	-0.53	-0.14	0.61
6-Hydroxymelatonin	2:103335280	G	C	C	G	0.06	-0.45	0.05	0.86
6-Hydroxymelatonin	2:103640530	T	C	C	T	0.11	-0.36	-0.39	0.13
6-Hydroxymelatonin	2:103641999	A	G	G	A	0.13	-0.35	-0.24	0.28
6-Hydroxymelatonin	2:104048792	C	T	T	C	0.06	-0.64	-0.21	0.50

6-Hydroxymelatonin	2:110130565	T	C	C	T	0.07	-0.54	0.00	1.00
6-Hydroxymelatonin	2:125936002	C	T	T	C	0.14	-0.31	-0.05	0.84
6-Hydroxymelatonin	2:16267646	G	A	A	G	0.10	-0.49	0.01	0.99
6-Hydroxymelatonin	2:17590711	A	G	G	A	0.10	-0.38	0.33	0.29
6-Hydroxymelatonin	2:26102068	G	A	A	G	0.07	-0.49	0.16	0.60
6-Hydroxymelatonin	2:26102101	C	G	G	C	0.07	-0.46	-0.07	0.84
6-Hydroxymelatonin	2:6070914	G	A	A	G	0.11	-0.38	0.81	0.02
6-Hydroxymelatonin	2:61441181	C	A	A	C	0.06	-0.49	0.13	0.74
6-Hydroxymelatonin	2:6284257	G	A	A	G	0.08	-0.52	0.08	0.80
6-Hydroxymelatonin	2:6286450	G	A	A	G	0.09	-0.37	0.03	0.91
6-Hydroxymelatonin	2:6295170	T	C	C	T	0.08	-0.51	-0.08	0.77
6-Hydroxymelatonin	20:23286217	C	A	A	C	0.07	-0.51	-0.17	0.66
6-Hydroxymelatonin	20:23850498	G	A	A	G	0.09	-0.44	0.15	0.67
6-Hydroxymelatonin	20:23943305	C	A	A	C	0.07	-0.54	-0.12	0.74
6-Hydroxymelatonin	20:33515895	G	A	A	G	0.05	-0.66	-0.08	0.84
6-Hydroxymelatonin	20:53949244	G	A	A	G	0.09	-0.42	0.00	0.99
6-Hydroxymelatonin	20:54781653	G	A	A	G	0.09	-0.41	0.07	0.80
6-Hydroxymelatonin	20:55627224	A	C	C	A	0.09	-0.41	0.39	0.17
6-Hydroxymelatonin	20:55630123	G	A	A	G	0.14	-0.33	0.70	0.01
6-Hydroxymelatonin	20:56368557	T	C	C	T	0.13	-0.37	0.31	0.24
6-Hydroxymelatonin	20:56371247	G	T	T	G	0.11	-0.44	0.63	0.05
6-Hydroxymelatonin	22:30588519	A	G	G	A	0.06	-0.50	-0.14	0.72
6-Hydroxymelatonin	22:30653705	G	A	A	G	0.07	-0.43	-0.30	0.40
6-Hydroxymelatonin	22:5127799	G	A	A	G	0.12	-0.39	0.54	0.08
6-Hydroxymelatonin	22:5435684	C	G	G	C	0.11	-0.38	0.02	0.94
6-Hydroxymelatonin	22:60629167	G	C	C	G	0.09	-0.43	0.60	0.02
6-Hydroxymelatonin	22:6791478	C	G	G	C	0.13	-0.38	0.48	0.11
6-Hydroxymelatonin	22:8327056	G	A	A	G	0.08	-0.40	0.55	0.09
6-Hydroxymelatonin	22:8328427	C	T	T	C	0.10	-0.44	0.43	0.14
6-Hydroxymelatonin	22:8328436	A	G	G	A	0.11	-0.39	0.67	0.02
6-Hydroxymelatonin	22:8612713	T	C	C	T	0.10	-0.43	0.63	0.03
6-Hydroxymelatonin	22:8749015	G	A	A	G	0.13	-0.36	0.51	0.11
6-Hydroxymelatonin	23:2268290	C	T	T	C	0.06	-0.55	-0.22	0.57
6-Hydroxymelatonin	23:23273491	G	A	A	G	0.06	-0.66	-0.10	0.78
6-Hydroxymelatonin	23:25973232	A	T	T	A	0.12	-0.45	0.89	0.00
6-Hydroxymelatonin	23:25973250	C	T	T	C	0.09	-0.44	0.78	0.03
6-Hydroxymelatonin	23:25985341	A	C	C	A	0.12	-0.44	0.32	0.29
6-Hydroxymelatonin	23:25985351	T	C	C	T	0.12	-0.45	0.24	0.44
6-Hydroxymelatonin	23:25985354	G	C	C	G	0.12	-0.42	0.26	0.40
6-Hydroxymelatonin	23:25985363	C	T	T	C	0.13	-0.42	0.18	0.54
6-Hydroxymelatonin	23:25985364	C	T	T	C	0.12	-0.45	-0.02	0.96
6-Hydroxymelatonin	23:25985367	A	C	C	A	0.13	-0.40	0.28	0.33
6-Hydroxymelatonin	23:25985384	A	T	T	A	0.13	-0.41	0.25	0.36
6-Hydroxymelatonin	23:25985394	A	G	G	A	0.14	-0.39	0.34	0.19

6-Hydroxymelatonin	23:26009631	A	G	G	A	0.08	-0.48	0.76	0.07
6-Hydroxymelatonin	23:28334729	G	C	C	G	0.06	-0.55	-0.08	0.83
6-Hydroxymelatonin	23:29120124	T	C	C	T	0.11	-0.41	0.07	0.81
6-Hydroxymelatonin	23:29146061	T	C	C	T	0.07	-0.45	-0.08	0.78
6-Hydroxymelatonin	23:29146124	C	T	T	C	0.08	-0.47	0.06	0.82
6-Hydroxymelatonin	23:29151480	T	C	C	T	0.07	-0.50	-0.28	0.28
6-Hydroxymelatonin	23:29468413	T	C	C	T	0.06	-0.58	-0.13	0.74
6-Hydroxymelatonin	23:3925120	C	A	A	C	0.10	-0.46	-0.28	0.29
6-Hydroxymelatonin	23:8811362	T	C	C	T	0.07	-0.48	-0.31	0.43
6-Hydroxymelatonin	24:28347884	C	T	T	C	0.07	-0.47	0.32	0.20
6-Hydroxymelatonin	24:31435305	T	C	C	T	0.18	-0.31	0.01	0.96
6-Hydroxymelatonin	24:32141363	G	A	A	G	0.05	-0.53	0.57	0.08
6-Hydroxymelatonin	24:33452288	T	A	A	T	0.07	-0.49	0.40	0.17
6-Hydroxymelatonin	24:6630108	C	T	T	C	0.07	-0.49	-0.15	0.67
6-Hydroxymelatonin	24:6698286	A	T	T	A	0.08	-0.37	-0.54	0.04
6-Hydroxymelatonin	24:6728623	T	C	C	T	0.06	-0.48	0.41	0.31
6-Hydroxymelatonin	25:30366529	A	C	C	A	0.05	-0.48	-0.32	0.38
6-Hydroxymelatonin	25:31495887	A	G	G	A	0.05	-0.47	-0.29	0.39
6-Hydroxymelatonin	25:31495895	A	G	G	A	0.05	-0.48	-0.30	0.37
6-Hydroxymelatonin	25:31506883	C	T	T	C	0.06	-0.63	-0.28	0.42
6-Hydroxymelatonin	25:3438815	T	C	C	T	0.06	-0.61	-0.29	0.53
6-Hydroxymelatonin	25:5806714	T	A	A	T	0.10	-0.37	0.49	0.07
6-Hydroxymelatonin	25:5854560	A	C	C	A	0.07	-0.51	0.80	0.04
6-Hydroxymelatonin	25:6253282	C	T	T	C	0.06	-0.54	0.69	0.05
6-Hydroxymelatonin	26:20939445	C	A	A	C	0.07	-0.48	0.17	0.66
6-Hydroxymelatonin	26:24871089	T	C	C	T	0.15	-0.36	-0.06	0.82
6-Hydroxymelatonin	26:46190352	G	A	A	G	0.07	-0.46	0.51	0.15
6-Hydroxymelatonin	26:7217692	G	A	A	G	0.13	-0.34	-0.01	0.96
6-Hydroxymelatonin	26:8416728	C	T	T	C	0.08	-0.52	0.48	0.15
6-Hydroxymelatonin	27:3022180	G	A	A	G	0.09	-0.39	0.46	0.13
6-Hydroxymelatonin	27:3023439	T	A	A	T	0.08	-0.53	0.81	0.01
6-Hydroxymelatonin	27:30509451	T	C	C	T	0.10	-0.41	-0.23	0.45
6-Hydroxymelatonin	27:31653066	C	T	T	C	0.09	-0.49	0.13	0.62
6-Hydroxymelatonin	27:31670861	G	A	A	G	0.10	-0.39	0.14	0.63
6-Hydroxymelatonin	27:3285455	T	C	C	T	0.10	-0.37	0.13	0.61
6-Hydroxymelatonin	27:42457420	C	T	T	C	0.09	-0.51	0.20	0.53
6-Hydroxymelatonin	27:42489865	C	T	T	C	0.08	-0.49	0.34	0.24
6-Hydroxymelatonin	27:42562181	C	T	T	C	0.06	-0.50	0.26	0.41
6-Hydroxymelatonin	28:13663320	G	A	A	G	0.07	-0.49	-0.12	0.77
6-Hydroxymelatonin	28:34502085	C	T	T	C	0.08	-0.44	0.75	0.01
6-Hydroxymelatonin	28:34505486	C	T	T	C	0.08	-0.51	0.28	0.45
6-Hydroxymelatonin	28:36384535	G	T	T	G	0.06	-0.54	0.13	0.77
6-Hydroxymelatonin	28:42654102	G	A	A	G	0.06	-0.45	0.12	0.74
6-Hydroxymelatonin	29:39266945	T	C	C	T	0.07	-0.47	-0.01	0.99

6-Hydroxymelatonin	29:39266947	C	G	G	C	0.07	-0.47	-0.03	0.94
6-Hydroxymelatonin	29:39266951	G	C	C	G	0.07	-0.47	0.01	0.97
6-Hydroxymelatonin	29:41820305	C	A	A	C	0.05	-0.63	-0.10	0.78
6-Hydroxymelatonin	29:43615938	G	A	A	G	0.06	-0.45	0.26	0.42
6-Hydroxymelatonin	3:112427851	C	T	T	C	0.06	-0.55	0.28	0.50
6-Hydroxymelatonin	3:114334742	G	A	A	G	0.08	-0.48	0.27	0.46
6-Hydroxymelatonin	3:63922876	C	T	T	C	0.07	-0.56	-0.07	0.87
6-Hydroxymelatonin	3:68740444	T	C	C	T	0.09	-0.34	0.41	0.22
6-Hydroxymelatonin	3:68759737	T	C	C	T	0.05	-0.47	0.49	0.14
6-Hydroxymelatonin	3:74467519	T	C	C	T	0.06	-0.57	0.14	0.71
6-Hydroxymelatonin	3:74571776	T	C	C	T	0.08	-0.43	0.26	0.44
6-Hydroxymelatonin	3:74571817	G	A	A	G	0.08	-0.57	0.55	0.12
6-Hydroxymelatonin	3:75073286	A	G	G	A	0.07	-0.56	0.06	0.86
6-Hydroxymelatonin	3:75151778	G	A	A	G	0.09	-0.48	-0.13	0.63
6-Hydroxymelatonin	3:75878570	T	C	C	T	0.10	-0.39	0.15	0.55
6-Hydroxymelatonin	3:78230168	C	T	T	C	0.06	-0.53	0.68	0.05
6-Hydroxymelatonin	3:90292949	G	T	T	G	0.09	-0.38	0.51	0.20
6-Hydroxymelatonin	30:107021257	T	C	C	T	0.06	-0.48	0.81	0.02
6-Hydroxymelatonin	30:11925223	T	C	C	T	0.12	-0.41	0.45	0.22
6-Hydroxymelatonin	30:427777	G	C	C	G	0.05	-0.51	0.39	0.26
6-Hydroxymelatonin	30:4434171	C	T	T	C	0.07	-0.51	0.00	1.00
6-Hydroxymelatonin	30:81244088	T	A	A	T	0.06	-0.52	0.08	0.80
6-Hydroxymelatonin	30:86935837	C	T	T	C	0.10	-0.45	-0.06	0.83
6-Hydroxymelatonin	4:104606782	C	T	T	C	0.07	-0.60	0.23	0.53
6-Hydroxymelatonin	4:104610563	A	G	G	A	0.12	-0.38	0.16	0.57
6-Hydroxymelatonin	4:104684871	C	T	T	C	0.07	-0.48	0.27	0.46
6-Hydroxymelatonin	4:104688660	C	T	T	C	0.06	-0.47	0.51	0.19
6-Hydroxymelatonin	4:105081924	C	T	T	C	0.14	-0.33	-0.13	0.68
6-Hydroxymelatonin	4:105126107	G	A	A	G	0.10	-0.37	0.21	0.50
6-Hydroxymelatonin	4:116966646	G	T	T	G	0.06	-0.50	0.67	0.03
6-Hydroxymelatonin	4:16514605	G	A	A	G	0.12	-0.37	-0.29	0.26
6-Hydroxymelatonin	4:17166286	A	G	G	A	0.06	-0.57	-0.04	0.92
6-Hydroxymelatonin	4:20459157	G	A	A	G	0.08	-0.45	0.40	0.36
6-Hydroxymelatonin	4:20466619	A	G	G	A	0.06	-0.67	0.18	0.68
6-Hydroxymelatonin	4:20478977	T	C	C	T	0.05	-0.56	0.78	0.02
6-Hydroxymelatonin	4:22749998	G	A	A	G	0.05	-0.50	0.32	0.37
6-Hydroxymelatonin	4:23548707	A	G	G	A	0.08	-0.73	-0.05	0.89
6-Hydroxymelatonin	4:23558874	C	A	A	C	0.07	-0.50	-0.03	0.93
6-Hydroxymelatonin	4:23558875	C	G	G	C	0.07	-0.50	-0.02	0.96
6-Hydroxymelatonin	4:24030096	C	G	G	C	0.05	-0.54	0.67	0.05
6-Hydroxymelatonin	4:31873176	G	A	A	G	0.06	-0.49	-0.18	0.49
6-Hydroxymelatonin	4:43974557	T	C	C	T	0.09	-0.43	-0.01	0.98
6-Hydroxymelatonin	4:43975031	A	G	G	A	0.10	-0.45	0.20	0.49
6-Hydroxymelatonin	4:75139094	C	A	A	C	0.08	-0.51	0.48	0.24

6-Hydroxymelatonin	4:75262763	C	T	T	C	0.09	-0.41	0.62	0.04
6-Hydroxymelatonin	4:75405126	C	T	T	C	0.09	-0.46	0.35	0.26
6-Hydroxymelatonin	4:75427701	T	G	G	T	0.09	-0.47	0.11	0.70
6-Hydroxymelatonin	4:75474044	G	A	A	G	0.07	-0.47	0.43	0.26
6-Hydroxymelatonin	4:75502609	C	A	A	C	0.08	-0.48	0.23	0.52
6-Hydroxymelatonin	4:75510696	T	C	C	T	0.11	-0.38	0.53	0.11
6-Hydroxymelatonin	4:75629050	C	T	T	C	0.11	-0.38	0.19	0.56
6-Hydroxymelatonin	4:75666657	C	T	T	C	0.07	-0.46	0.44	0.21
6-Hydroxymelatonin	4:75668000	C	T	T	C	0.08	-0.49	0.74	0.04
6-Hydroxymelatonin	4:75805688	C	T	T	C	0.13	-0.34	0.18	0.54
6-Hydroxymelatonin	4:75805709	G	T	T	G	0.13	-0.29	0.24	0.40
6-Hydroxymelatonin	4:75890683	T	C	C	T	0.11	-0.39	0.39	0.25
6-Hydroxymelatonin	4:75899205	A	T	T	A	0.10	-0.38	0.40	0.22
6-Hydroxymelatonin	4:75970439	G	A	A	G	0.11	-0.42	0.33	0.29
6-Hydroxymelatonin	4:75974931	T	C	C	T	0.06	-0.60	0.02	0.96
6-Hydroxymelatonin	4:75983636	G	A	A	G	0.12	-0.39	0.26	0.46
6-Hydroxymelatonin	4:76022495	C	T	T	C	0.10	-0.45	0.36	0.24
6-Hydroxymelatonin	4:76023590	A	T	T	A	0.08	-0.46	0.49	0.17
6-Hydroxymelatonin	4:76036675	C	T	T	C	0.08	-0.47	0.28	0.44
6-Hydroxymelatonin	4:76047603	C	T	T	C	0.08	-0.46	0.44	0.21
6-Hydroxymelatonin	4:76049484	C	A	A	C	0.11	-0.43	0.35	0.24
6-Hydroxymelatonin	4:76078470	G	A	A	G	0.11	-0.35	0.60	0.05
6-Hydroxymelatonin	4:76090039	G	C	C	G	0.11	-0.38	0.36	0.29
6-Hydroxymelatonin	4:76114212	T	A	A	T	0.12	-0.44	0.48	0.12
6-Hydroxymelatonin	4:76123261	C	T	T	C	0.12	-0.39	0.77	0.01
6-Hydroxymelatonin	4:76125557	A	G	G	A	0.13	-0.34	0.62	0.04
6-Hydroxymelatonin	4:76159057	C	T	T	C	0.11	-0.43	0.85	0.01
6-Hydroxymelatonin	4:76159085	G	C	C	G	0.11	-0.42	0.83	0.00
6-Hydroxymelatonin	4:76422540	G	A	A	G	0.12	-0.34	0.55	0.08
6-Hydroxymelatonin	4:8508987	G	A	A	G	0.06	-0.51	0.35	0.38
6-Hydroxymelatonin	4:85998463	C	T	T	C	0.15	-0.32	-0.06	0.79
6-Hydroxymelatonin	4:86079622	T	C	C	T	0.11	-0.45	0.21	0.49
6-Hydroxymelatonin	4:86105928	G	A	A	G	0.13	-0.35	0.02	0.93
6-Hydroxymelatonin	4:89229856	T	G	G	T	0.11	-0.34	0.19	0.56
6-Hydroxymelatonin	4:89230014	C	A	A	C	0.09	-0.47	0.23	0.48
6-Hydroxymelatonin	4:89230896	A	C	C	A	0.07	-0.53	0.23	0.52
6-Hydroxymelatonin	4:89366667	G	A	A	G	0.09	-0.40	-0.04	0.89
6-Hydroxymelatonin	4:90000082	C	A	A	C	0.10	-0.38	0.22	0.50
6-Hydroxymelatonin	4:91158741	C	G	G	C	0.07	-0.44	0.08	0.83
6-Hydroxymelatonin	4:91158748	A	G	G	A	0.08	-0.44	0.06	0.86
6-Hydroxymelatonin	4:91537108	A	G	G	A	0.10	-0.41	0.29	0.41
6-Hydroxymelatonin	4:91762436	T	A	A	T	0.06	-0.46	0.07	0.85
6-Hydroxymelatonin	4:91844944	G	A	A	G	0.08	-0.44	-0.02	0.94
6-Hydroxymelatonin	4:94647450	A	G	G	A	0.05	-0.51	0.59	0.12

6-Hydroxymelatonin	4:94683267	C	T	T	C	0.05	-0.54	0.90	0.02
6-Hydroxymelatonin	4:95510381	G	C	C	G	0.05	-0.49	0.74	0.08
6-Hydroxymelatonin	4:97434203	G	A	A	G	0.06	-0.51	0.22	0.57
6-Hydroxymelatonin	4:97579487	G	A	A	G	0.14	-0.32	-0.09	0.74
6-Hydroxymelatonin	4:97954195	T	G	G	T	0.10	-0.40	0.22	0.44
6-Hydroxymelatonin	4:97963412	G	A	A	G	0.11	-0.38	0.13	0.66
6-Hydroxymelatonin	5:100319328	A	G	G	A	0.06	-0.53	-0.18	0.52
6-Hydroxymelatonin	5:101648966	C	G	G	C	0.06	-0.57	0.49	0.16
6-Hydroxymelatonin	5:106315745	G	A	A	G	0.16	-0.32	-0.17	0.45
6-Hydroxymelatonin	5:106891734	G	A	A	G	0.14	-0.33	0.14	0.62
6-Hydroxymelatonin	5:106904058	C	T	T	C	0.07	-0.45	0.27	0.47
6-Hydroxymelatonin	5:106921998	A	G	G	A	0.07	-0.48	-0.09	0.79
6-Hydroxymelatonin	5:106922001	G	T	T	G	0.07	-0.48	-0.08	0.80
6-Hydroxymelatonin	5:106922087	T	C	C	T	0.07	-0.58	-0.22	0.50
6-Hydroxymelatonin	5:106922131	T	C	C	T	0.08	-0.48	-0.34	0.30
6-Hydroxymelatonin	5:106922164	G	A	A	G	0.08	-0.48	-0.34	0.30
6-Hydroxymelatonin	5:106924610	A	T	T	A	0.07	-0.52	-0.26	0.38
6-Hydroxymelatonin	5:106924948	A	G	G	A	0.08	-0.51	-0.20	0.56
6-Hydroxymelatonin	5:108272227	A	T	T	A	0.07	-0.50	0.38	0.23
6-Hydroxymelatonin	5:108323693	C	T	T	C	0.07	-0.41	1.08	0.00
6-Hydroxymelatonin	5:108405896	A	G	G	A	0.08	-0.50	0.30	0.32
6-Hydroxymelatonin	5:108421047	G	T	T	G	0.08	-0.46	0.33	0.31
6-Hydroxymelatonin	5:108422242	C	T	T	C	0.10	-0.38	0.33	0.30
6-Hydroxymelatonin	5:108443724	C	T	T	C	0.06	-0.49	0.25	0.39
6-Hydroxymelatonin	5:108458947	G	A	A	G	0.08	-0.45	0.09	0.74
6-Hydroxymelatonin	5:108480875	C	T	T	C	0.07	-0.57	0.54	0.12
6-Hydroxymelatonin	5:108500821	G	A	A	G	0.09	-0.40	0.06	0.83
6-Hydroxymelatonin	5:108502157	C	A	A	C	0.10	-0.41	0.16	0.59
6-Hydroxymelatonin	5:108513547	G	A	A	G	0.09	-0.45	0.24	0.43
6-Hydroxymelatonin	5:108601687	C	T	T	C	0.11	-0.34	0.33	0.19
6-Hydroxymelatonin	5:108662953	G	A	A	G	0.11	-0.37	0.29	0.34
6-Hydroxymelatonin	5:112684664	A	G	G	A	0.05	-0.56	0.04	0.92
6-Hydroxymelatonin	5:116752702	T	C	C	T	0.05	-0.52	-0.22	0.45
6-Hydroxymelatonin	5:47852096	T	C	C	T	0.09	-0.48	0.11	0.68
6-Hydroxymelatonin	5:50211578	C	T	T	C	0.07	-0.51	0.05	0.84
6-Hydroxymelatonin	5:59745953	T	C	C	T	0.06	-0.62	-0.11	0.81
6-Hydroxymelatonin	5:86793512	C	T	T	C	0.05	-0.53	0.81	0.04
6-Hydroxymelatonin	5:91416057	T	A	A	T	0.07	-0.42	-0.05	0.88
6-Hydroxymelatonin	6:111138050	G	A	A	G	0.12	-0.33	0.09	0.67
6-Hydroxymelatonin	6:49339291	G	A	A	G	0.10	-0.39	0.46	0.11
6-Hydroxymelatonin	6:49339898	G	A	A	G	0.06	-0.47	0.39	0.17
6-Hydroxymelatonin	6:49438487	A	T	T	A	0.07	-0.46	0.10	0.81
6-Hydroxymelatonin	6:63942559	C	T	T	C	0.07	-0.53	0.19	0.57
6-Hydroxymelatonin	6:70827404	T	C	C	T	0.06	-0.62	0.12	0.75

6-Hydroxymelatonin	7:106648878	A	T	T	A	0.09	-0.39	-0.34	0.33
6-Hydroxymelatonin	7:106843025	G	A	A	G	0.08	-0.48	0.19	0.47
6-Hydroxymelatonin	7:15259278	C	T	T	C	0.07	-0.45	0.96	0.01
6-Hydroxymelatonin	7:17766890	A	G	G	A	0.05	-0.66	0.33	0.42
6-Hydroxymelatonin	7:18215677	G	C	C	G	0.07	-0.44	0.31	0.36
6-Hydroxymelatonin	7:20093648	C	T	T	C	0.07	-0.48	0.89	0.01
6-Hydroxymelatonin	7:22095454	T	G	G	T	0.07	-0.47	0.40	0.16
6-Hydroxymelatonin	7:23004543	T	C	C	T	0.13	-0.34	-0.10	0.69
6-Hydroxymelatonin	7:3459856	G	T	T	G	0.08	-0.43	-0.09	0.73
6-Hydroxymelatonin	7:3708766	G	A	A	G	0.05	-0.58	0.22	0.49
6-Hydroxymelatonin	7:3860876	A	T	T	A	0.06	-0.51	0.42	0.15
6-Hydroxymelatonin	7:89592751	G	C	C	G	0.06	-0.54	-0.26	0.51
6-Hydroxymelatonin	7:89592757	T	G	G	T	0.06	-0.54	-0.25	0.52
6-Hydroxymelatonin	7:89592760	T	A	A	T	0.06	-0.54	-0.26	0.51
6-Hydroxymelatonin	7:89592766	A	G	G	A	0.06	-0.49	-0.28	0.47
6-Hydroxymelatonin	8:11385984	G	C	C	G	0.08	-0.43	-0.34	0.24
6-Hydroxymelatonin	8:13888172	A	G	G	A	0.09	-0.42	-0.06	0.85
6-Hydroxymelatonin	8:14382503	T	C	C	T	0.07	-0.51	0.38	0.33
6-Hydroxymelatonin	8:14839069	C	A	A	C	0.06	-0.53	0.32	0.39
6-Hydroxymelatonin	8:14920267	T	C	C	T	0.06	-0.47	-0.14	0.70
6-Hydroxymelatonin	8:14944516	T	G	G	T	0.06	-0.54	-0.01	0.97
6-Hydroxymelatonin	8:14963198	C	T	T	C	0.06	-0.52	0.13	0.71
6-Hydroxymelatonin	8:15105867	C	T	T	C	0.05	-0.57	-0.09	0.78
6-Hydroxymelatonin	8:15116177	C	T	T	C	0.06	-0.58	0.03	0.93
6-Hydroxymelatonin	8:15125936	C	T	T	C	0.06	-0.59	0.03	0.92
6-Hydroxymelatonin	8:15128273	C	T	T	C	0.06	-0.59	0.22	0.50
6-Hydroxymelatonin	8:15792238	G	A	A	G	0.06	-0.58	-0.01	0.98
6-Hydroxymelatonin	8:16004084	A	G	G	A	0.07	-0.43	0.27	0.46
6-Hydroxymelatonin	8:16176985	A	C	C	A	0.06	-0.57	-0.02	0.95
6-Hydroxymelatonin	8:16513165	C	T	T	C	0.08	-0.45	0.45	0.16
6-Hydroxymelatonin	8:16513194	C	T	T	C	0.08	-0.42	0.33	0.24
6-Hydroxymelatonin	8:16517626	T	G	G	T	0.07	-0.42	0.41	0.19
6-Hydroxymelatonin	8:16532804	C	T	T	C	0.07	-0.53	0.12	0.73
6-Hydroxymelatonin	8:16826264	C	T	T	C	0.06	-0.61	-0.12	0.73
6-Hydroxymelatonin	8:17152701	C	G	G	C	0.07	-0.46	0.31	0.34
6-Hydroxymelatonin	8:31964663	T	C	C	T	0.12	-0.37	0.00	0.99
6-Hydroxymelatonin	8:45212497	A	G	G	A	0.05	-0.53	-0.22	0.54
6-Hydroxymelatonin	8:45253477	C	T	T	C	0.06	-0.60	-0.37	0.35
6-Hydroxymelatonin	8:48959098	G	A	A	G	0.06	-0.61	0.87	0.04
6-Hydroxymelatonin	8:82716852	G	T	T	G	0.12	-0.39	0.58	0.07
6-Hydroxymelatonin	8:82872477	C	T	T	C	0.13	-0.32	0.40	0.12
6-Hydroxymelatonin	8:82872483	A	G	G	A	0.14	-0.33	0.40	0.11
6-Hydroxymelatonin	8:83230986	T	C	C	T	0.12	-0.36	0.27	0.32
6-Hydroxymelatonin	8:8362820	C	T	T	C	0.05	-0.62	0.21	0.58

6-Hydroxymelatonin	9:102155882	C	T	T	C	0.07	-0.45	0.21	0.50
6-Hydroxymelatonin	9:19348927	C	T	T	C	0.07	-0.44	-0.29	0.38
6-Hydroxymelatonin	9:19405211	T	C	C	T	0.07	-0.48	-0.26	0.41
6-Hydroxymelatonin	9:19405233	A	G	G	A	0.08	-0.39	-0.18	0.61
6-Hydroxymelatonin	9:2764123	A	C	C	A	0.06	-0.48	-0.28	0.42
6-Hydroxymelatonin	9:2793172	T	G	G	T	0.05	-0.51	-0.20	0.59
6-Hydroxymelatonin	9:304008	G	A	A	G	0.06	-0.53	0.18	0.65
6-Hydroxymelatonin	9:304010	G	A	A	G	0.06	-0.54	0.18	0.65
6-Hydroxymelatonin	9:304016	C	T	T	C	0.06	-0.54	0.13	0.75
6-Hydroxymelatonin	9:52204807	G	A	A	G	0.07	-0.53	0.58	0.14
6-Hydroxymelatonin	9:52204810	G	A	A	G	0.08	-0.50	0.58	0.14
6-Hydroxymelatonin	9:65976301	T	C	C	T	0.10	-0.45	0.06	0.83
1-(5Z,8Z,11Z,14Z-eicosatetraenoyl)-sn-glycero-3-phosphate	16:643042	T	C	C	T	0.15	0.28	-0.13	0.59
1-(5Z,8Z,11Z,14Z-eicosatetraenoyl)-sn-glycero-3-phosphate	16:75234253	T	C	C	T	0.13	0.26	0.02	0.95
1-(5Z,8Z,11Z,14Z-eicosatetraenoyl)-sn-glycero-3-phosphate	16:75979869	G	A	A	G	0.13	0.31	0.04	0.90
1-(5Z,8Z,11Z,14Z-eicosatetraenoyl)-sn-glycero-3-phosphate	16:75983945	C	T	T	C	0.10	0.33	0.08	0.79
1-(5Z,8Z,11Z,14Z-eicosatetraenoyl)-sn-glycero-3-phosphate	16:75985593	C	A	A	C	0.10	0.32	0.14	0.62
1-(5Z,8Z,11Z,14Z-eicosatetraenoyl)-sn-glycero-3-phosphate	16:76976577	C	T	T	C	0.09	0.42	-0.09	0.80
1-(5Z,8Z,11Z,14Z-eicosatetraenoyl)-sn-glycero-3-phosphate	16:77403358	T	C	C	T	0.06	0.47	0.47	0.20
1-(5Z,8Z,11Z,14Z-eicosatetraenoyl)-sn-glycero-3-phosphate	16:79089703	A	G	G	A	0.12	0.35	0.34	0.28
1-(5Z,8Z,11Z,14Z-eicosatetraenoyl)-sn-glycero-3-phosphate	16:79722419	T	C	C	T	0.07	0.39	0.43	0.28
1-(5Z,8Z,11Z,14Z-eicosatetraenoyl)-sn-glycero-3-phosphate	16:79793458	G	A	A	G	0.06	0.39	0.40	0.32
1-(5Z,8Z,11Z,14Z-eicosatetraenoyl)-sn-glycero-3-phosphate	16:79883544	C	T	T	C	0.08	0.39	0.36	0.33
1-(5Z,8Z,11Z,14Z-eicosatetraenoyl)-sn-glycero-3-phosphate	16:79924845	C	T	T	C	0.08	0.32	0.26	0.45
1-(5Z,8Z,11Z,14Z-eicosatetraenoyl)-sn-glycero-3-phosphate	16:80017009	G	T	T	G	0.08	0.37	0.17	0.60
1-(5Z,8Z,11Z,14Z-eicosatetraenoyl)-sn-glycero-3-phosphate	16:80078680	G	A	A	G	0.07	0.34	0.73	0.05
1-(5Z,8Z,11Z,14Z-eicosatetraenoyl)-sn-glycero-3-phosphate	16:80130299	A	G	G	A	0.07	0.35	0.32	0.37
1-(5Z,8Z,11Z,14Z-eicosatetraenoyl)-sn-glycero-3-phosphate	16:80130300	G	A	A	G	0.07	0.35	0.31	0.38

1-(5Z,8Z,11Z,14Z-eicosatetraenoyl)-sn-glycero-3-phosphate	16:80130650	A	G	G	A	0.05	0.42	0.86	0.04
1-(5Z,8Z,11Z,14Z-eicosatetraenoyl)-sn-glycero-3-phosphate	16:80132277	A	C	C	A	0.07	0.33	0.55	0.12
1-(5Z,8Z,11Z,14Z-eicosatetraenoyl)-sn-glycero-3-phosphate	16:80140344	A	G	G	A	0.08	0.35	0.64	0.10
1-(5Z,8Z,11Z,14Z-eicosatetraenoyl)-sn-glycero-3-phosphate	16:80153656	T	A	A	T	0.06	0.43	0.51	0.20
1-(5Z,8Z,11Z,14Z-eicosatetraenoyl)-sn-glycero-3-phosphate	16:80157272	C	T	T	C	0.06	0.42	0.41	0.23
1-(5Z,8Z,11Z,14Z-eicosatetraenoyl)-sn-glycero-3-phosphate	16:80213347	G	A	A	G	0.06	0.42	0.67	0.04
1-(5Z,8Z,11Z,14Z-eicosatetraenoyl)-sn-glycero-3-phosphate	19:34194998	A	G	G	A	0.06	-0.43	0.01	0.98
1-(5Z,8Z,11Z,14Z-eicosatetraenoyl)-sn-glycero-3-phosphate	19:34973424	T	C	C	T	0.09	-0.34	0.41	0.26
1-(5Z,8Z,11Z,14Z-eicosatetraenoyl)-sn-glycero-3-phosphate	19:40093355	G	A	A	G	0.05	-0.44	-0.04	0.93
1-(5Z,8Z,11Z,14Z-eicosatetraenoyl)-sn-glycero-3-phosphate	29:1273559	C	T	T	C	0.13	0.28	0.07	0.82
1-(5Z,8Z,11Z,14Z-eicosatetraenoyl)-sn-glycero-3-phosphate	29:3414800	G	A	A	G	0.08	0.37	0.78	0.05
1-(5Z,8Z,11Z,14Z-eicosatetraenoyl)-sn-glycero-3-phosphate	5:24241106	C	T	T	C	0.13	-0.30	0.03	0.90
1-(5Z,8Z,11Z,14Z-eicosatetraenoyl)-sn-glycero-3-phosphate	5:24247775	C	T	T	C	0.11	-0.30	0.68	0.01
D-Citrulline	1:10058037	G	C	C	G	0.11	-0.97	0.23	0.47
D-Citrulline	1:10058164	C	T	T	C	0.12	-0.89	0.30	0.28
D-Citrulline	1:10072211	T	C	C	T	0.12	-0.90	-0.01	0.96
D-Citrulline	1:10072261	C	T	T	C	0.14	-0.66	-0.03	0.89
D-Citrulline	1:10072692	C	T	T	C	0.13	-0.76	0.06	0.84
D-Citrulline	1:10075541	A	C	C	A	0.14	-0.81	0.17	0.59
D-Citrulline	1:10076255	T	C	C	T	0.11	-0.88	0.01	0.98
D-Citrulline	1:10076329	T	C	C	T	0.09	-0.85	0.26	0.41
D-Citrulline	1:10077152	G	C	C	G	0.14	-0.69	0.01	0.98
D-Citrulline	1:10077185	G	A	A	G	0.13	-0.78	0.19	0.52
D-Citrulline	1:10077197	C	T	T	C	0.10	-1.00	0.11	0.72
D-Citrulline	1:10077200	A	T	T	A	0.10	-1.00	0.03	0.94
D-Citrulline	1:10077247	T	C	C	T	0.09	-0.79	0.17	0.55
D-Citrulline	1:10077249	G	T	T	G	0.09	-0.79	0.17	0.57
D-Citrulline	1:10077281	T	C	C	T	0.12	-0.83	0.18	0.57
D-Citrulline	1:10077340	C	T	T	C	0.14	-0.82	0.19	0.55
D-Citrulline	1:10077367	G	C	C	G	0.14	-0.79	0.22	0.47
D-Citrulline	1:10089103	C	T	T	C	0.12	-0.89	0.08	0.77

D-Citrulline	1:73339496	T	C	C	T	0.08	-0.99	-0.43	0.20
D-Citrulline	1:87537786	T	C	C	T	0.06	-1.13	-0.22	0.60
D-Citrulline	1:9712407	G	A	A	G	0.10	-0.90	0.48	0.12
D-Citrulline	10:20379196	A	G	G	A	0.08	-1.02	-0.17	0.52
D-Citrulline	10:20626787	A	C	C	A	0.09	-0.98	0.17	0.60
D-Citrulline	10:20626789	A	G	G	A	0.09	-0.98	0.19	0.57
D-Citrulline	10:20627701	G	A	A	G	0.11	-0.83	0.22	0.49
D-Citrulline	10:23023483	G	C	C	G	0.08	-0.97	0.44	0.18
D-Citrulline	10:32257425	T	C	C	T	0.06	-0.98	-0.56	0.13
D-Citrulline	10:83248491	C	A	A	C	0.07	-1.08	-0.32	0.35
D-Citrulline	10:91807289	C	T	T	C	0.07	-1.08	-0.16	0.67
D-Citrulline	11:81868368	C	A	A	C	0.10	-0.93	-0.52	0.13
D-Citrulline	11:82118372	G	A	A	G	0.08	-1.00	-0.45	0.18
D-Citrulline	11:84490458	C	T	T	C	0.11	-0.90	-0.46	0.08
D-Citrulline	12:10917704	C	T	T	C	0.10	-0.89	-0.18	0.58
D-Citrulline	12:11232104	C	T	T	C	0.16	-0.71	0.03	0.92
D-Citrulline	12:48532193	T	C	C	T	0.07	-1.12	-0.13	0.69
D-Citrulline	12:56213747	G	A	A	G	0.13	-0.88	-0.17	0.53
D-Citrulline	12:56354658	T	G	G	T	0.17	-0.71	-0.40	0.09
D-Citrulline	12:61831069	C	T	T	C	0.05	-1.08	-0.47	0.15
D-Citrulline	12:61831080	T	C	C	T	0.06	-1.09	-0.50	0.13
D-Citrulline	12:61831081	C	A	A	C	0.06	-1.09	-0.53	0.11
D-Citrulline	12:84752486	G	A	A	G	0.06	-1.04	-0.04	0.91
D-Citrulline	12:86740972	C	T	T	C	0.09	-0.91	-0.30	0.31
D-Citrulline	12:86779788	C	T	T	C	0.10	-1.02	-0.47	0.16
D-Citrulline	13:28564124	C	T	T	C	0.15	-0.72	-0.01	0.96
D-Citrulline	13:32256300	T	A	A	T	0.08	-1.02	-0.06	0.86
D-Citrulline	13:32283991	T	A	A	T	0.09	-0.93	0.06	0.86
D-Citrulline	13:82066140	C	T	T	C	0.09	-0.88	-0.19	0.58
D-Citrulline	14:14034325	C	A	A	C	0.05	-1.01	-0.37	0.40
D-Citrulline	14:29721675	T	C	C	T	0.10	-0.86	-0.25	0.44
D-Citrulline	15:25724500	C	T	T	C	0.11	-0.80	0.07	0.81
D-Citrulline	15:27346763	G	A	A	G	0.13	-0.80	-0.17	0.62
D-Citrulline	15:27504450	A	G	G	A	0.15	-0.70	0.07	0.81
D-Citrulline	15:27968754	T	A	A	T	0.14	-0.69	0.18	0.57
D-Citrulline	15:28186688	T	A	A	T	0.14	-0.81	-0.18	0.56
D-Citrulline	15:28490496	C	T	T	C	0.13	-0.72	0.03	0.94
D-Citrulline	15:28546739	T	C	C	T	0.11	-0.84	0.20	0.58
D-Citrulline	15:59347786	C	T	T	C	0.18	-0.65	-0.25	0.37
D-Citrulline	15:81779901	G	C	C	G	0.08	-0.99	-0.27	0.43
D-Citrulline	16:17332599	G	A	A	G	0.08	-0.95	-0.24	0.49
D-Citrulline	16:17332605	A	C	C	A	0.09	-0.83	-0.04	0.90
D-Citrulline	16:17436782	A	G	G	A	0.06	-0.91	-0.88	0.03
D-Citrulline	16:4611153	G	A	A	G	0.06	-0.91	1.03	0.01

D-Citrulline	16:4633336	T	C	C	T	0.07	-1.01	0.72	0.02
D-Citrulline	16:59732744	T	G	G	T	0.08	-0.82	0.06	0.85
D-Citrulline	16:6141368	C	A	A	C	0.13	-0.71	0.14	0.63
D-Citrulline	16:75682187	G	A	A	G	0.07	-1.07	0.09	0.77
D-Citrulline	16:79933378	T	C	C	T	0.07	-1.14	0.20	0.52
D-Citrulline	16:80140467	G	A	A	G	0.09	-0.86	0.14	0.66
D-Citrulline	16:80158951	A	G	G	A	0.06	-0.98	0.36	0.42
D-Citrulline	17:51627966	A	G	G	A	0.11	-0.84	0.09	0.75
D-Citrulline	18:28258947	G	A	A	G	0.11	-0.90	-0.14	0.62
D-Citrulline	18:28451606	A	G	G	A	0.14	-0.73	-0.02	0.96
D-Citrulline	18:29068739	C	T	T	C	0.13	-0.83	0.21	0.50
D-Citrulline	18:30192830	A	G	G	A	0.07	-1.08	-0.37	0.31
D-Citrulline	18:30204169	A	T	T	A	0.07	-1.17	-0.28	0.37
D-Citrulline	18:30209724	T	A	A	T	0.06	-1.10	-0.69	0.02
D-Citrulline	18:30647207	C	A	A	C	0.13	-0.73	0.09	0.76
D-Citrulline	18:31391997	A	G	G	A	0.09	-0.93	0.08	0.79
D-Citrulline	18:31406293	T	A	A	T	0.05	-1.27	-0.30	0.35
D-Citrulline	18:31683336	T	A	A	T	0.06	-1.33	-0.51	0.18
D-Citrulline	18:31686676	G	A	A	G	0.05	-1.14	-0.23	0.50
D-Citrulline	18:31704833	A	G	G	A	0.06	-1.41	-0.35	0.36
D-Citrulline	18:31707721	T	C	C	T	0.06	-1.45	0.15	0.66
D-Citrulline	18:31725978	C	T	T	C	0.06	-1.20	-0.58	0.10
D-Citrulline	18:31792545	A	G	G	A	0.15	-0.81	-0.21	0.37
D-Citrulline	18:31824297	A	G	G	A	0.05	-1.24	-0.33	0.37
D-Citrulline	18:31885848	A	G	G	A	0.05	-1.55	-0.03	0.93
D-Citrulline	18:31988859	C	A	A	C	0.05	-1.16	0.13	0.67
D-Citrulline	18:32082567	C	T	T	C	0.07	-1.11	-0.62	0.06
D-Citrulline	18:32101639	G	T	T	G	0.07	-0.98	-0.50	0.09
D-Citrulline	18:32103837	G	A	A	G	0.09	-0.86	-0.75	0.01
D-Citrulline	18:32122462	A	G	G	A	0.07	-1.11	-0.68	0.04
D-Citrulline	18:32129909	G	T	T	G	0.07	-1.08	-0.74	0.01
D-Citrulline	18:32383958	C	A	A	C	0.05	-1.61	-0.40	0.28
D-Citrulline	18:32396449	C	G	G	C	0.07	-1.03	-0.30	0.36
D-Citrulline	18:32414491	C	A	A	C	0.06	-1.03	0.04	0.91
D-Citrulline	18:32456710	A	G	G	A	0.07	-1.08	-0.50	0.05
D-Citrulline	18:32464668	C	T	T	C	0.10	-1.03	-0.31	0.23
D-Citrulline	18:32464901	C	A	A	C	0.09	-0.96	-0.44	0.16
D-Citrulline	18:32922896	T	C	C	T	0.05	-1.49	-0.71	0.08
D-Citrulline	18:32924467	C	T	T	C	0.05	-1.30	-0.42	0.26
D-Citrulline	18:32931904	T	C	C	T	0.06	-1.32	-0.22	0.55
D-Citrulline	18:32944471	C	T	T	C	0.05	-1.43	-0.24	0.45
D-Citrulline	18:32985625	C	T	T	C	0.06	-1.38	-0.46	0.15
D-Citrulline	18:33703345	G	A	A	G	0.05	-1.32	-0.74	0.03
D-Citrulline	18:33928495	C	T	T	C	0.08	-0.88	-0.32	0.23

D-Citrulline	18:34828479	C	G	G	C	0.12	-0.82	0.13	0.61
D-Citrulline	18:34916572	C	T	T	C	0.13	-0.82	-0.20	0.45
D-Citrulline	18:35056328	A	G	G	A	0.14	-0.74	-0.06	0.80
D-Citrulline	18:35158036	A	G	G	A	0.05	-1.34	0.06	0.86
D-Citrulline	18:35216967	C	T	T	C	0.05	-1.56	-0.18	0.59
D-Citrulline	18:35344336	C	G	G	C	0.05	-1.20	-0.28	0.44
D-Citrulline	18:35379148	C	G	G	C	0.05	-1.36	-0.39	0.19
D-Citrulline	18:35476385	G	A	A	G	0.07	-1.05	-0.11	0.69
D-Citrulline	18:35824553	C	T	T	C	0.12	-0.77	-0.21	0.37
D-Citrulline	18:36767189	A	C	C	A	0.05	-1.18	-0.57	0.12
D-Citrulline	19:2824699	C	T	T	C	0.06	-1.14	-0.01	0.97
D-Citrulline	19:3422551	T	C	C	T	0.05	-1.17	-0.34	0.37
D-Citrulline	19:3427952	T	A	A	T	0.06	-1.36	-0.53	0.15
D-Citrulline	19:3428137	G	A	A	G	0.06	-1.41	-0.39	0.31
D-Citrulline	19:3428147	G	A	A	G	0.06	-1.19	-0.41	0.30
D-Citrulline	19:3428222	C	A	A	C	0.06	-1.14	-0.54	0.14
D-Citrulline	19:38393946	T	C	C	T	0.10	-0.93	-0.37	0.33
D-Citrulline	19:41842640	C	T	T	C	0.14	-0.69	0.34	0.14
D-Citrulline	19:47496378	T	C	C	T	0.16	-0.74	-0.36	0.16
D-Citrulline	19:5576393	C	A	A	C	0.06	-1.12	-0.30	0.41
D-Citrulline	19:5576456	C	T	T	C	0.07	-0.98	-0.08	0.83
D-Citrulline	19:62436580	C	T	T	C	0.07	-0.95	-0.23	0.58
D-Citrulline	19:762284	C	G	G	C	0.05	-1.26	-0.53	0.18
D-Citrulline	2:111464823	T	C	C	T	0.13	-0.73	0.11	0.71
D-Citrulline	2:111471276	G	A	A	G	0.07	-1.13	0.15	0.66
D-Citrulline	2:114065304	T	C	C	T	0.07	-1.09	0.06	0.88
D-Citrulline	2:123310696	T	C	C	T	0.06	-1.29	0.00	0.99
D-Citrulline	2:123310707	T	C	C	T	0.06	-1.19	0.23	0.55
D-Citrulline	2:123310711	T	A	A	T	0.06	-1.24	0.20	0.60
D-Citrulline	2:123310712	G	A	A	G	0.06	-1.24	0.20	0.60
D-Citrulline	2:123310721	T	A	A	T	0.06	-1.18	0.23	0.56
D-Citrulline	2:123310722	C	G	G	C	0.06	-1.18	0.23	0.56
D-Citrulline	2:123310741	G	C	C	G	0.05	-1.14	0.26	0.52
D-Citrulline	2:128941892	T	G	G	T	0.09	-0.98	0.63	0.12
D-Citrulline	2:12909998	C	T	T	C	0.08	-0.91	0.28	0.37
D-Citrulline	2:134776021	G	A	A	G	0.06	-1.16	0.04	0.91
D-Citrulline	2:135172061	A	G	G	A	0.07	-0.96	-0.17	0.57
D-Citrulline	2:135201206	C	A	A	C	0.06	-1.07	-0.14	0.70
D-Citrulline	2:17003365	G	A	A	G	0.12	-0.74	-0.16	0.58
D-Citrulline	2:21319626	T	C	C	T	0.16	-0.68	0.08	0.73
D-Citrulline	2:21340553	G	A	A	G	0.09	-1.08	0.13	0.68
D-Citrulline	2:23195915	T	C	C	T	0.08	-1.06	0.41	0.19
D-Citrulline	2:23289736	T	C	C	T	0.07	-0.97	0.37	0.44
D-Citrulline	2:53230628	G	A	A	G	0.10	-0.90	-0.17	0.53

D-Citrulline	2:53230635	G	A	A	G	0.10	-0.96	-0.21	0.45
D-Citrulline	2:60856616	C	G	G	C	0.09	-0.87	-0.35	0.30
D-Citrulline	2:60966140	T	G	G	T	0.06	-1.20	0.17	0.63
D-Citrulline	2:62089916	G	A	A	G	0.09	-0.90	-0.01	0.98
D-Citrulline	2:62402676	C	T	T	C	0.09	-0.90	0.00	0.99
D-Citrulline	2:62517068	C	T	T	C	0.10	-0.85	-0.33	0.23
D-Citrulline	2:62601401	G	T	T	G	0.10	-0.86	-0.21	0.44
D-Citrulline	2:62660907	A	G	G	A	0.11	-1.05	0.03	0.92
D-Citrulline	2:62686982	T	C	C	T	0.07	-0.95	-0.18	0.59
D-Citrulline	2:62710239	G	A	A	G	0.10	-1.00	-0.24	0.45
D-Citrulline	2:62721475	A	C	C	A	0.10	-0.95	0.02	0.94
D-Citrulline	2:62728133	T	C	C	T	0.11	-0.90	-0.22	0.47
D-Citrulline	2:63026857	A	G	G	A	0.11	-0.86	0.05	0.86
D-Citrulline	2:63094927	C	T	T	C	0.07	-0.94	-0.21	0.52
D-Citrulline	2:63734550	G	A	A	G	0.08	-1.00	-0.27	0.40
D-Citrulline	2:63911496	G	A	A	G	0.15	-0.78	0.23	0.36
D-Citrulline	2:63937874	T	C	C	T	0.10	-0.94	0.15	0.60
D-Citrulline	2:63950550	C	T	T	C	0.11	-0.85	0.55	0.06
D-Citrulline	2:64087750	C	A	A	C	0.07	-1.17	-0.27	0.46
D-Citrulline	2:64094348	C	T	T	C	0.08	-1.15	-0.36	0.26
D-Citrulline	2:64114997	T	G	G	T	0.10	-0.90	0.02	0.96
D-Citrulline	2:64173024	G	A	A	G	0.09	-0.88	-0.23	0.45
D-Citrulline	2:64431755	A	G	G	A	0.13	-0.79	0.13	0.65
D-Citrulline	2:64437638	G	C	C	G	0.13	-0.83	0.33	0.19
D-Citrulline	2:64455437	G	A	A	G	0.10	-0.78	-0.04	0.91
D-Citrulline	2:64484988	C	T	T	C	0.12	-0.81	0.08	0.78
D-Citrulline	2:64504449	C	T	T	C	0.11	-0.78	0.20	0.49
D-Citrulline	2:64540028	C	A	A	C	0.12	-0.79	0.16	0.56
D-Citrulline	2:64543869	C	G	G	C	0.12	-0.77	-0.03	0.91
D-Citrulline	2:64574070	C	T	T	C	0.07	-1.21	0.07	0.83
D-Citrulline	2:66068955	C	A	A	C	0.08	-1.14	0.09	0.77
D-Citrulline	2:66073134	C	G	G	C	0.07	-1.05	0.15	0.68
D-Citrulline	2:66074276	G	A	A	G	0.08	-0.92	-0.12	0.69
D-Citrulline	2:66109316	C	A	A	C	0.07	-1.13	0.19	0.59
D-Citrulline	2:74407895	G	A	A	G	0.15	-0.75	0.13	0.61
D-Citrulline	20:2407617	C	G	G	C	0.08	-0.94	-0.30	0.42
D-Citrulline	20:6403980	G	A	A	G	0.08	-1.03	-0.20	0.63
D-Citrulline	20:6403996	T	A	A	T	0.07	-1.15	-0.10	0.82
D-Citrulline	20:65616367	A	G	G	A	0.07	-0.94	-0.59	0.18
D-Citrulline	20:6617335	T	C	C	T	0.08	-0.91	-0.22	0.62
D-Citrulline	20:6714833	A	G	G	A	0.07	-0.91	-0.20	0.61
D-Citrulline	20:68398299	T	C	C	T	0.08	-0.89	-0.47	0.27
D-Citrulline	20:6850906	C	G	G	C	0.08	-1.00	0.19	0.56
D-Citrulline	20:68989598	T	G	G	T	0.06	-0.94	-0.48	0.21

D-Citrulline	20:6972230	T	G	G	T	0.09	-0.82	-0.31	0.44
D-Citrulline	20:70353398	C	T	T	C	0.07	-0.95	-0.24	0.61
D-Citrulline	20:70971752	C	T	T	C	0.06	-1.10	-0.21	0.56
D-Citrulline	20:7572256	G	A	A	G	0.09	-0.89	0.19	0.65
D-Citrulline	20:7761671	C	T	T	C	0.09	-0.79	-0.10	0.72
D-Citrulline	20:8970272	G	A	A	G	0.08	-0.95	0.53	0.18
D-Citrulline	21:62033297	C	T	T	C	0.11	-0.88	-0.41	0.18
D-Citrulline	21:62044925	T	C	C	T	0.10	-0.96	-0.36	0.20
D-Citrulline	22:55733221	G	C	C	G	0.09	-0.93	-0.15	0.63
D-Citrulline	22:58047826	C	T	T	C	0.07	-1.13	0.64	0.11
D-Citrulline	23:23529159	T	A	A	T	0.05	-1.05	-0.11	0.76
D-Citrulline	23:23991651	G	A	A	G	0.06	-1.21	-0.08	0.86
D-Citrulline	23:24045230	C	T	T	C	0.06	-1.25	0.04	0.91
D-Citrulline	23:24191669	T	C	C	T	0.10	-0.91	-0.26	0.45
D-Citrulline	23:25562502	T	C	C	T	0.07	-0.97	0.56	0.15
D-Citrulline	23:25562519	G	A	A	G	0.08	-0.87	0.79	0.03
D-Citrulline	23:25885881	T	C	C	T	0.08	-0.93	0.49	0.22
D-Citrulline	23:26621277	C	T	T	C	0.11	-0.93	0.42	0.31
D-Citrulline	23:27093101	G	A	A	G	0.13	-0.71	0.07	0.76
D-Citrulline	23:27110089	C	T	T	C	0.05	-1.15	0.26	0.49
D-Citrulline	23:27323973	A	G	G	A	0.15	-0.66	0.42	0.09
D-Citrulline	23:27359098	G	A	A	G	0.13	-0.66	0.40	0.08
D-Citrulline	23:27360333	C	A	A	C	0.11	-0.75	0.46	0.05
D-Citrulline	23:27384970	G	C	C	G	0.11	-0.76	0.47	0.17
D-Citrulline	23:28040314	C	T	T	C	0.06	-0.97	0.66	0.08
D-Citrulline	23:28048169	C	T	T	C	0.13	-0.79	0.29	0.31
D-Citrulline	23:28048170	A	G	G	A	0.13	-0.79	0.29	0.31
D-Citrulline	23:28051017	T	C	C	T	0.12	-0.82	0.74	0.01
D-Citrulline	23:28186386	T	C	C	T	0.06	-1.23	0.52	0.23
D-Citrulline	23:28196763	A	G	G	A	0.09	-0.85	0.33	0.36
D-Citrulline	23:28416393	C	T	T	C	0.11	-0.86	0.16	0.61
D-Citrulline	23:28560150	G	T	T	G	0.07	-1.02	0.42	0.34
D-Citrulline	23:28604794	C	T	T	C	0.13	-0.72	0.36	0.20
D-Citrulline	23:28816977	C	G	G	C	0.11	-0.85	0.65	0.03
D-Citrulline	23:28840519	G	A	A	G	0.07	-0.95	0.41	0.32
D-Citrulline	23:29286918	G	C	C	G	0.06	-1.02	0.14	0.71
D-Citrulline	23:5966279	T	G	G	T	0.10	-0.87	0.47	0.08
D-Citrulline	24:16517850	C	T	T	C	0.13	-0.79	-0.04	0.90
D-Citrulline	24:52665797	T	C	C	T	0.09	-0.97	0.44	0.13
D-Citrulline	25:19670035	A	T	T	A	0.15	-0.66	-0.13	0.56
D-Citrulline	25:19895228	C	T	T	C	0.11	-0.83	-0.14	0.55
D-Citrulline	25:19940625	C	T	T	C	0.17	-0.63	-0.03	0.90
D-Citrulline	25:20007824	T	A	A	T	0.12	-0.74	-0.02	0.91
D-Citrulline	25:20141857	G	A	A	G	0.13	-0.77	-0.19	0.39

D-Citrulline	25:31154126	G	T	T	G	0.08	-0.88	-0.11	0.67
D-Citrulline	25:31158531	T	C	C	T	0.10	-0.94	0.11	0.70
D-Citrulline	25:31162226	T	C	C	T	0.07	-1.00	0.24	0.46
D-Citrulline	25:32354073	G	A	A	G	0.07	-1.09	0.25	0.49
D-Citrulline	25:33181102	G	A	A	G	0.12	-0.81	-0.07	0.80
D-Citrulline	25:34213144	G	A	A	G	0.11	-0.86	-0.11	0.67
D-Citrulline	25:995272	C	A	A	C	0.10	-0.87	0.03	0.91
D-Citrulline	26:4377947	T	G	G	T	0.09	-0.97	-0.34	0.33
D-Citrulline	26:51738127	C	T	T	C	0.06	-1.00	-0.19	0.61
D-Citrulline	27:20767669	A	G	G	A	0.13	-0.73	-0.01	0.97
D-Citrulline	27:20861343	G	C	C	G	0.09	-0.94	-0.22	0.52
D-Citrulline	27:22168787	G	A	A	G	0.13	-0.85	-0.12	0.68
D-Citrulline	27:22743247	A	G	G	A	0.14	-0.77	-0.25	0.41
D-Citrulline	27:22846388	G	A	A	G	0.12	-0.82	0.07	0.82
D-Citrulline	27:22846396	C	T	T	C	0.11	-0.86	0.08	0.80
D-Citrulline	27:26683361	A	G	G	A	0.07	-0.93	-0.43	0.25
D-Citrulline	28:37685572	G	T	T	G	0.08	-0.95	0.75	0.01
D-Citrulline	28:40317580	G	A	A	G	0.06	-1.04	-0.05	0.87
D-Citrulline	28:41485565	A	G	G	A	0.06	-1.08	0.14	0.64
D-Citrulline	28:43265187	A	G	G	A	0.07	-1.05	0.07	0.80
D-Citrulline	28:43273065	T	G	G	T	0.05	-1.36	0.29	0.44
D-Citrulline	28:7405816	C	T	T	C	0.06	-0.97	0.30	0.44
D-Citrulline	29:24827280	A	G	G	A	0.14	-0.76	0.15	0.56
D-Citrulline	29:40070841	C	T	T	C	0.05	-1.24	-0.30	0.47
D-Citrulline	3:10281224	C	T	T	C	0.07	-1.12	-0.31	0.39
D-Citrulline	3:10282331	T	G	G	T	0.05	-1.50	-0.34	0.35
D-Citrulline	3:10284597	C	T	T	C	0.08	-0.91	-0.28	0.37
D-Citrulline	3:20041079	A	T	T	A	0.10	-1.12	-0.24	0.41
D-Citrulline	3:22754566	C	T	T	C	0.06	-1.02	-0.15	0.67
D-Citrulline	3:22756540	A	G	G	A	0.08	-0.86	-0.24	0.59
D-Citrulline	3:3242368	G	A	A	G	0.05	-1.32	-0.42	0.22
D-Citrulline	3:33447195	T	C	C	T	0.05	-1.21	0.22	0.52
D-Citrulline	3:33521044	G	A	A	G	0.06	-1.03	0.09	0.80
D-Citrulline	3:34068134	A	C	C	A	0.08	-0.95	0.19	0.58
D-Citrulline	3:34175291	C	T	T	C	0.08	-0.92	0.23	0.57
D-Citrulline	3:34201660	G	A	A	G	0.08	-0.88	0.25	0.45
D-Citrulline	3:34466332	C	T	T	C	0.08	-0.94	0.23	0.56
D-Citrulline	3:36921851	A	G	G	A	0.12	-0.86	-0.19	0.52
D-Citrulline	3:37477601	C	T	T	C	0.08	-1.03	-0.18	0.54
D-Citrulline	30:111605602	G	A	A	G	0.06	-1.09	-0.33	0.38
D-Citrulline	30:111894562	A	G	G	A	0.09	-0.92	-0.65	0.07
D-Citrulline	30:111895478	T	C	C	T	0.10	-0.84	-0.69	0.05
D-Citrulline	30:121230456	A	G	G	A	0.16	-0.78	-0.34	0.22
D-Citrulline	30:121368202	C	T	T	C	0.19	-0.67	0.28	0.35

D-Citrulline	30:12668756	C	A	A	C	0.06	-1.02	0.01	0.98
D-Citrulline	30:126951792	T	C	C	T	0.11	-0.78	0.28	0.44
D-Citrulline	30:12793316	G	C	C	G	0.07	-1.16	0.33	0.28
D-Citrulline	30:137596753	C	T	T	C	0.09	-0.90	0.25	0.42
D-Citrulline	30:137812781	C	T	T	C	0.10	-0.87	0.07	0.81
D-Citrulline	30:137812782	A	C	C	A	0.10	-0.87	0.08	0.79
D-Citrulline	30:137817876	G	A	A	G	0.09	-0.93	-0.09	0.79
D-Citrulline	30:137817993	C	T	T	C	0.15	-0.67	-0.15	0.56
D-Citrulline	30:137891724	T	G	G	T	0.08	-0.89	0.10	0.82
D-Citrulline	30:137944772	A	G	G	A	0.11	-0.86	0.28	0.44
D-Citrulline	30:16856957	G	A	A	G	0.10	-0.91	-0.04	0.90
D-Citrulline	30:16906774	G	A	A	G	0.10	-0.93	0.17	0.58
D-Citrulline	30:17728325	A	C	C	A	0.15	-0.80	-0.08	0.75
D-Citrulline	30:24602667	T	A	A	T	0.06	-1.13	0.33	0.40
D-Citrulline	30:27221094	T	C	C	T	0.11	-0.81	0.14	0.67
D-Citrulline	30:27221212	G	A	A	G	0.11	-0.77	0.40	0.13
D-Citrulline	30:31215126	G	A	A	G	0.09	-0.90	0.27	0.50
D-Citrulline	30:37174447	G	A	A	G	0.06	-1.31	0.73	0.15
D-Citrulline	30:37181288	C	T	T	C	0.07	-0.91	0.61	0.16
D-Citrulline	30:40434364	A	T	T	A	0.06	-1.07	0.38	0.40
D-Citrulline	30:49199408	C	T	T	C	0.05	-1.23	0.24	0.60
D-Citrulline	30:89955741	C	T	T	C	0.10	-0.93	-0.19	0.54
D-Citrulline	30:99927576	T	A	A	T	0.07	-1.01	-0.35	0.29
D-Citrulline	4:15395213	T	C	C	T	0.19	-0.61	-0.04	0.86
D-Citrulline	4:15572079	A	G	G	A	0.06	-1.09	-0.10	0.75
D-Citrulline	4:22655426	T	C	C	T	0.06	-1.08	0.15	0.73
D-Citrulline	4:22909590	C	T	T	C	0.07	-1.06	0.45	0.25
D-Citrulline	4:22917377	G	A	A	G	0.06	-1.06	0.21	0.53
D-Citrulline	4:22926728	C	T	T	C	0.07	-0.91	-0.26	0.44
D-Citrulline	4:22928192	T	G	G	T	0.06	-1.01	0.23	0.53
D-Citrulline	4:22939929	T	G	G	T	0.07	-1.10	-0.09	0.80
D-Citrulline	4:22995429	G	A	A	G	0.08	-0.95	0.01	0.98
D-Citrulline	4:29292110	G	A	A	G	0.06	-1.15	-0.14	0.70
D-Citrulline	4:31884349	C	G	G	C	0.10	-0.91	-0.20	0.56
D-Citrulline	4:6723660	A	C	C	A	0.08	-0.93	-0.02	0.95
D-Citrulline	4:6728099	T	C	C	T	0.09	-0.89	0.03	0.93
D-Citrulline	4:7082236	A	C	C	A	0.05	-1.43	0.28	0.41
D-Citrulline	4:7095537	T	C	C	T	0.08	-0.86	0.05	0.89
D-Citrulline	4:7095857	T	G	G	T	0.06	-1.02	-0.01	0.97
D-Citrulline	4:7098291	G	A	A	G	0.05	-1.19	0.02	0.96
D-Citrulline	4:7098311	G	A	A	G	0.06	-1.01	0.20	0.50
D-Citrulline	4:7208339	G	C	C	G	0.11	-0.82	0.10	0.75
D-Citrulline	4:7211178	T	C	C	T	0.10	-0.98	-0.35	0.24
D-Citrulline	4:7222476	G	A	A	G	0.09	-1.09	-0.11	0.69

D-Citrulline	4:7222517	G	C	C	G	0.11	-1.03	-0.05	0.86
D-Citrulline	4:7222760	C	T	T	C	0.12	-0.77	-0.21	0.47
D-Citrulline	4:7222865	C	G	G	C	0.14	-0.73	0.02	0.94
D-Citrulline	4:7222869	A	T	T	A	0.14	-0.71	-0.09	0.69
D-Citrulline	4:7223011	A	G	G	A	0.12	-0.82	-0.47	0.06
D-Citrulline	4:7435606	C	G	G	C	0.05	-1.25	-0.28	0.49
D-Citrulline	4:7435613	T	C	C	T	0.05	-1.25	-0.28	0.49
D-Citrulline	4:7435624	T	A	A	T	0.05	-1.26	-0.32	0.42
D-Citrulline	4:7435643	C	A	A	C	0.05	-1.25	-0.33	0.41
D-Citrulline	4:7612350	G	A	A	G	0.17	-0.66	-0.27	0.21
D-Citrulline	4:7612439	A	C	C	A	0.17	-0.75	-0.38	0.13
D-Citrulline	5:118850597	C	G	G	C	0.06	-0.99	0.81	0.05
D-Citrulline	5:118851611	G	A	A	G	0.05	-1.21	0.46	0.30
D-Citrulline	5:118990534	G	C	C	G	0.10	-0.83	-0.21	0.40
D-Citrulline	5:35692520	G	A	A	G	0.05	-0.99	-0.37	0.37
D-Citrulline	5:36874841	T	C	C	T	0.16	-0.66	0.16	0.58
D-Citrulline	5:96649450	C	T	T	C	0.07	-1.04	-0.24	0.47
D-Citrulline	6:106520205	T	C	C	T	0.06	-1.12	-0.29	0.46
D-Citrulline	6:55444370	C	T	T	C	0.06	-1.16	0.27	0.44
D-Citrulline	7:90160238	T	C	C	T	0.16	-0.65	-0.24	0.33
D-Citrulline	7:92084264	C	T	T	C	0.06	-1.11	-0.43	0.25
D-Citrulline	7:92422947	C	T	T	C	0.10	-0.96	0.58	0.05
D-Citrulline	9:100476700	T	C	C	T	0.15	-0.70	0.18	0.54
D-Citrulline	9:100532424	G	A	A	G	0.16	-0.73	-0.06	0.81
D-Citrulline	9:100649205	A	G	G	A	0.17	-0.68	0.07	0.81
D-Citrulline	9:3777892	A	C	C	A	0.17	-0.67	-0.24	0.41
D-Citrulline	9:88265282	T	C	C	T	0.08	-0.83	0.48	0.15
D-Citrulline	9:90470916	T	C	C	T	0.17	-0.67	0.27	0.25
D-Citrulline	9:99998768	G	A	A	G	0.10	-0.85	-0.01	0.98
Val Leu Gly	11:30752266	C	T	T	C	0.10	-0.27	-0.39	0.29
Val Leu Gly	12:13199017	G	A	A	G	0.10	-0.28	-0.02	0.95
Val Leu Gly	12:13339808	T	G	G	T	0.11	-0.28	-0.05	0.89
Val Leu Gly	12:35206011	C	T	T	C	0.14	-0.27	-0.06	0.83
Val Leu Gly	14:30998512	T	C	C	T	0.05	-0.43	-0.66	0.13
Val Leu Gly	14:33927189	C	T	T	C	0.06	-0.41	-0.66	0.07
Val Leu Gly	16:75234253	T	C	C	T	0.13	-0.23	0.02	0.95
Val Leu Gly	16:75985593	C	A	A	C	0.10	-0.30	0.14	0.62
Val Leu Gly	16:76606682	G	A	A	G	0.08	-0.41	0.32	0.30
Val Leu Gly	16:76617942	A	G	G	A	0.08	-0.31	0.27	0.42
Val Leu Gly	16:76617950	C	T	T	C	0.07	-0.36	0.18	0.61
Val Leu Gly	16:76628823	C	A	A	C	0.09	-0.31	0.36	0.29
Val Leu Gly	16:76636361	A	T	T	A	0.07	-0.34	0.40	0.22
Val Leu Gly	16:76796531	A	T	T	A	0.09	-0.30	0.04	0.91
Val Leu Gly	16:76921192	G	A	A	G	0.07	-0.34	0.11	0.72

Val Leu Gly	16:76976577	C	T	T	C	0.09	-0.41	-0.09	0.80
Val Leu Gly	16:76993870	G	A	A	G	0.08	-0.33	-0.08	0.80
Val Leu Gly	16:77011129	T	A	A	T	0.10	-0.29	0.25	0.46
Val Leu Gly	16:77012711	G	A	A	G	0.08	-0.37	0.08	0.84
Val Leu Gly	16:77021822	A	T	T	A	0.09	-0.38	0.16	0.64
Val Leu Gly	16:77021823	T	C	C	T	0.09	-0.38	0.11	0.75
Val Leu Gly	16:77033279	C	T	T	C	0.09	-0.28	-0.28	0.44
Val Leu Gly	16:77042188	C	T	T	C	0.09	-0.28	-0.08	0.83
Val Leu Gly	16:77202268	G	A	A	G	0.10	-0.29	0.11	0.72
Val Leu Gly	16:77403358	T	C	C	T	0.06	-0.43	0.47	0.20
Val Leu Gly	16:78119235	G	A	A	G	0.08	-0.37	0.28	0.40
Val Leu Gly	16:78275057	C	T	T	C	0.10	-0.32	0.05	0.89
Val Leu Gly	16:78293956	G	A	A	G	0.12	-0.24	0.46	0.16
Val Leu Gly	16:79094857	A	C	C	A	0.19	-0.22	-0.01	0.98
Val Leu Gly	16:79100573	C	G	G	C	0.07	-0.38	0.58	0.10
Val Leu Gly	16:79100906	C	T	T	C	0.08	-0.29	0.38	0.32
Val Leu Gly	16:79104587	C	T	T	C	0.10	-0.29	0.10	0.74
Val Leu Gly	16:79291232	A	C	C	A	0.08	-0.35	0.43	0.22
Val Leu Gly	16:79722419	T	C	C	T	0.07	-0.40	0.43	0.28
Val Leu Gly	16:79726216	C	T	T	C	0.07	-0.34	0.37	0.24
Val Leu Gly	16:79750910	T	G	G	T	0.07	-0.33	0.69	0.02
Val Leu Gly	16:79793384	G	A	A	G	0.07	-0.35	0.58	0.14
Val Leu Gly	16:79793458	G	A	A	G	0.06	-0.35	0.40	0.32
Val Leu Gly	16:79862333	A	G	G	A	0.07	-0.32	0.10	0.77
Val Leu Gly	16:79876597	G	A	A	G	0.08	-0.36	0.83	0.03
Val Leu Gly	16:79883544	C	T	T	C	0.08	-0.35	0.36	0.33
Val Leu Gly	16:79889426	T	A	A	T	0.10	-0.29	0.53	0.09
Val Leu Gly	16:79912088	T	A	A	T	0.06	-0.40	0.45	0.23
Val Leu Gly	16:79924845	C	T	T	C	0.08	-0.36	0.26	0.45
Val Leu Gly	16:79952687	A	G	G	A	0.09	-0.37	0.17	0.62
Val Leu Gly	16:79952923	A	G	G	A	0.09	-0.31	0.01	0.97
Val Leu Gly	16:79953140	C	T	T	C	0.08	-0.32	-0.18	0.57
Val Leu Gly	16:79977320	T	C	C	T	0.08	-0.29	0.14	0.64
Val Leu Gly	16:79977920	C	G	G	C	0.10	-0.29	0.09	0.77
Val Leu Gly	16:79978546	C	T	T	C	0.08	-0.35	0.31	0.37
Val Leu Gly	16:79984567	T	C	C	T	0.08	-0.36	0.01	0.99
Val Leu Gly	16:79996725	T	C	C	T	0.09	-0.33	-0.21	0.47
Val Leu Gly	16:79999440	C	T	T	C	0.09	-0.30	-0.14	0.65
Val Leu Gly	16:80002732	A	G	G	A	0.09	-0.37	-0.04	0.90
Val Leu Gly	16:80006574	C	T	T	C	0.10	-0.31	0.39	0.23
Val Leu Gly	16:80010858	C	A	A	C	0.09	-0.37	0.16	0.66
Val Leu Gly	16:80017009	G	T	T	G	0.08	-0.31	0.17	0.60
Val Leu Gly	16:80017103	C	T	T	C	0.08	-0.30	-0.01	0.98
Val Leu Gly	16:80022824	A	G	G	A	0.08	-0.32	0.33	0.29

Val Leu Gly	16:80023667	C	G	G	C	0.07	-0.37	0.01	0.97
Val Leu Gly	16:80027242	T	C	C	T	0.06	-0.36	0.17	0.62
Val Leu Gly	16:80027576	G	A	A	G	0.08	-0.35	-0.11	0.74
Val Leu Gly	16:80027639	G	A	A	G	0.08	-0.34	0.12	0.70
Val Leu Gly	16:80057301	G	T	T	G	0.09	-0.34	0.07	0.82
Val Leu Gly	16:80073892	G	A	A	G	0.07	-0.34	0.44	0.25
Val Leu Gly	16:80078655	C	T	T	C	0.09	-0.29	0.42	0.19
Val Leu Gly	16:80078680	G	A	A	G	0.07	-0.30	0.73	0.05
Val Leu Gly	16:80128597	T	A	A	T	0.07	-0.40	0.07	0.85
Val Leu Gly	16:80129109	T	C	C	T	0.09	-0.35	0.41	0.21
Val Leu Gly	16:80130299	A	G	G	A	0.07	-0.29	0.32	0.37
Val Leu Gly	16:80130300	G	A	A	G	0.07	-0.29	0.31	0.38
Val Leu Gly	16:80130461	G	A	A	G	0.07	-0.34	0.28	0.37
Val Leu Gly	16:80130650	A	G	G	A	0.05	-0.43	0.86	0.04
Val Leu Gly	16:80135290	A	C	C	A	0.07	-0.37	0.46	0.26
Val Leu Gly	16:80140344	A	G	G	A	0.08	-0.39	0.64	0.10
Val Leu Gly	16:80149837	T	C	C	T	0.06	-0.37	0.53	0.14
Val Leu Gly	16:80151069	C	T	T	C	0.06	-0.41	0.67	0.10
Val Leu Gly	16:80153656	T	A	A	T	0.06	-0.42	0.51	0.20
Val Leu Gly	16:80156302	T	C	C	T	0.07	-0.39	0.71	0.05
Val Leu Gly	16:80156631	G	A	A	G	0.08	-0.37	0.19	0.61
Val Leu Gly	16:80157016	T	C	C	T	0.06	-0.38	0.59	0.13
Val Leu Gly	16:80157272	C	T	T	C	0.06	-0.39	0.41	0.23
Val Leu Gly	16:80157476	C	G	G	C	0.06	-0.36	0.44	0.23
Val Leu Gly	16:80157485	T	C	C	T	0.07	-0.34	0.44	0.23
Val Leu Gly	16:80158218	C	A	A	C	0.06	-0.37	0.51	0.15
Val Leu Gly	16:80158225	C	G	G	C	0.06	-0.38	0.46	0.18
Val Leu Gly	16:80158273	T	C	C	T	0.06	-0.35	0.39	0.27
Val Leu Gly	16:80161455	C	T	T	C	0.07	-0.35	0.52	0.19
Val Leu Gly	16:80161768	A	G	G	A	0.09	-0.27	0.49	0.15
Val Leu Gly	16:80162560	G	C	C	G	0.07	-0.35	0.48	0.23
Val Leu Gly	16:80162592	A	G	G	A	0.06	-0.38	0.58	0.15
Val Leu Gly	16:80164989	A	G	G	A	0.08	-0.30	0.77	0.05
Val Leu Gly	16:80165944	G	A	A	G	0.07	-0.34	0.76	0.06
Val Leu Gly	16:80165984	C	T	T	C	0.06	-0.32	0.29	0.43
Val Leu Gly	16:80166059	G	A	A	G	0.06	-0.35	0.13	0.74
Val Leu Gly	16:80166633	C	G	G	C	0.07	-0.32	-0.05	0.88
Val Leu Gly	16:80166920	C	G	G	C	0.06	-0.31	0.86	0.03
Val Leu Gly	16:80213347	G	A	A	G	0.06	-0.47	0.67	0.04
Val Leu Gly	16:80224236	G	A	A	G	0.07	-0.32	0.12	0.70
Val Leu Gly	16:80236594	G	A	A	G	0.06	-0.38	0.22	0.50
Val Leu Gly	16:80239086	C	G	G	C	0.08	-0.28	0.37	0.32
Val Leu Gly	16:80361906	A	G	G	A	0.06	-0.41	0.24	0.45
Val Leu Gly	16:80389923	A	G	G	A	0.09	-0.33	-0.11	0.74

Val Leu Gly	16:80389924	A	C	C	A	0.09	-0.33	-0.14	0.68
Val Leu Gly	16:80390813	C	T	T	C	0.06	-0.38	0.37	0.31
Val Leu Gly	16:80393485	T	C	C	T	0.13	-0.26	0.51	0.03
Val Leu Gly	16:80404582	C	T	T	C	0.05	-0.44	0.18	0.64
Val Leu Gly	16:80408713	C	T	T	C	0.06	-0.35	0.39	0.32
Val Leu Gly	16:80432856	G	A	A	G	0.08	-0.36	0.35	0.33
Val Leu Gly	16:80433479	G	A	A	G	0.10	-0.30	0.45	0.17
Val Leu Gly	16:80513954	C	T	T	C	0.08	-0.31	0.52	0.08
Val Leu Gly	16:80529684	C	T	T	C	0.05	-0.42	0.60	0.11
Val Leu Gly	16:80533891	G	A	A	G	0.09	-0.31	0.29	0.33
Val Leu Gly	16:80611536	C	T	T	C	0.06	-0.45	0.37	0.30
Val Leu Gly	16:80785293	G	T	T	G	0.06	-0.42	0.49	0.22
Val Leu Gly	17:54716766	G	A	A	G	0.06	-0.35	-0.10	0.76
Val Leu Gly	19:36644437	G	C	C	G	0.14	-0.25	-0.29	0.25
Val Leu Gly	19:36644456	G	A	A	G	0.13	-0.26	-0.25	0.32
Val Leu Gly	19:40437809	T	C	C	T	0.13	-0.25	0.16	0.60
Val Leu Gly	19:40969550	A	G	G	A	0.13	-0.22	0.03	0.92
Val Leu Gly	19:42519358	G	A	A	G	0.13	-0.24	0.00	0.99
Val Leu Gly	19:44684933	T	C	C	T	0.12	-0.29	0.05	0.89
Val Leu Gly	21:19093575	C	T	T	C	0.16	0.23	-0.21	0.39
Val Leu Gly	21:20231520	C	G	G	C	0.06	-0.37	-0.14	0.74
Val Leu Gly	29:15711528	G	A	A	G	0.06	-0.38	0.00	1.00
Val Leu Gly	29:6700464	C	A	A	C	0.06	-0.38	-0.18	0.68
Val Leu Gly	3:10584441	A	C	C	A	0.15	-0.24	0.28	0.41
Val Leu Gly	3:10614407	T	C	C	T	0.13	-0.27	0.17	0.65
Val Leu Gly	3:13585637	G	A	A	G	0.11	-0.25	0.24	0.51
Val Leu Gly	3:2962343	A	G	G	A	0.17	-0.23	0.42	0.15
Val Leu Gly	3:9338072	C	T	T	C	0.10	-0.29	0.76	0.04
Val Leu Gly	3:9534597	G	A	A	G	0.13	-0.26	0.41	0.30
Val Leu Gly	3:9908927	T	C	C	T	0.12	-0.28	0.55	0.12
Val Leu Gly	4:95879687	C	T	T	C	0.08	-0.31	-0.10	0.78
Val Leu Gly	8:72067618	C	T	T	C	0.06	0.42	-0.40	0.29
Val Leu Gly	8:76703706	T	C	C	T	0.07	-0.35	-0.04	0.92
Val Leu Gly	8:76703893	G	A	A	G	0.06	-0.40	0.11	0.65
Val Leu Gly	8:76704017	C	T	T	C	0.07	-0.41	0.22	0.47
25-Acetylvulgaroside	10:94778237	G	A	A	G	0.06	-0.08	-0.31	0.34
25-Acetylvulgaroside	16:14453086	G	T	T	G	0.13	-0.05	-0.20	0.47
25-Acetylvulgaroside	16:14463672	T	C	C	T	0.12	-0.06	-0.36	0.22
25-Acetylvulgaroside	16:14463676	G	T	T	G	0.11	-0.06	-0.36	0.22
25-Acetylvulgaroside	20:1018341	C	T	T	C	0.11	-0.06	-0.10	0.77
25-Acetylvulgaroside	20:1020765	T	C	C	T	0.14	-0.05	0.00	0.99
25-Acetylvulgaroside	20:1020884	A	G	G	A	0.08	-0.07	-0.08	0.75
25-Acetylvulgaroside	20:1067752	C	T	T	C	0.10	-0.06	-0.22	0.43
25-Acetylvulgaroside	20:1098025	T	C	C	T	0.11	-0.06	-0.25	0.42

25-Acetylvulgaroside	20:1138430	G	T	T	G	0.12	-0.06	-0.16	0.62
25-Acetylvulgaroside	20:1159977	C	T	T	C	0.12	-0.06	-0.37	0.22
25-Acetylvulgaroside	20:1233770	A	G	G	A	0.13	-0.06	-0.09	0.72
25-Acetylvulgaroside	20:1295208	G	A	A	G	0.15	-0.05	-0.08	0.75
25-Acetylvulgaroside	20:1453098	A	G	G	A	0.14	-0.05	-0.12	0.64
25-Acetylvulgaroside	20:1513086	A	G	G	A	0.10	-0.07	0.19	0.49
25-Acetylvulgaroside	20:1558916	C	T	T	C	0.13	-0.06	-0.18	0.46
25-Acetylvulgaroside	21:8026721	G	A	A	G	0.08	0.07	0.35	0.41
25-Acetylvulgaroside	25:2364407	G	A	A	G	0.10	-0.07	0.51	0.14
25-Acetylvulgaroside	29:37994190	A	C	C	A	0.07	-0.08	-0.73	0.10
25-Acetylvulgaroside	30:117889892	T	C	C	T	0.11	-0.06	0.27	0.43
25-Acetylvulgaroside	8:44004159	C	T	T	C	0.08	-0.07	-0.35	0.19
25-Acetylvulgaroside	8:8298809	G	A	A	G	0.11	-0.06	-0.37	0.20
25-Acetylvulgaroside	8:83113410	C	G	G	C	0.09	-0.07	-0.24	0.52
25-Acetylvulgaroside	8:85630542	G	A	A	G	0.14	-0.05	-0.31	0.20
4-Hydroxyproline galactoside	11:102298750	C	T	T	C	0.05	-0.30	-0.53	0.15
4-Hydroxyproline galactoside	11:78736207	G	A	A	G	0.06	-0.29	-0.03	0.94
4-Hydroxyproline galactoside	16:35648018	G	C	C	G	0.11	-0.22	0.24	0.43
4-Hydroxyproline galactoside	16:35648068	T	G	G	T	0.12	-0.22	0.26	0.36
4-Hydroxyproline galactoside	16:39612030	C	T	T	C	0.10	-0.23	0.10	0.74
4-Hydroxyproline galactoside	17:9800018	G	A	A	G	0.11	-0.23	-0.19	0.55
4-Hydroxyproline galactoside	17:9809131	T	G	G	T	0.09	-0.23	-0.61	0.08
4-Hydroxyproline galactoside	2:68672456	C	G	G	C	0.06	-0.34	-0.31	0.38
4-Hydroxyproline galactoside	23:16922	T	C	C	T	0.07	-0.30	0.41	0.35
4-Hydroxyproline galactoside	29:37994201	C	T	T	C	0.06	-0.32	-0.79	0.06
4-Hydroxyproline galactoside	4:99618493	G	A	A	G	0.07	-0.26	0.07	0.84
4-Hydroxyproline galactoside	5:102791743	A	T	T	A	0.05	-0.33	-0.89	0.03
4-Hydroxyproline galactoside	5:93601529	A	T	T	A	0.07	-0.27	-0.04	0.90
4-Hydroxyproline galactoside	7:72584320	C	T	T	C	0.10	-0.22	0.01	0.97
4-Hydroxyproline galactoside	7:72826344	T	C	C	T	0.17	-0.17	-0.02	0.95
4-Hydroxyproline galactoside	7:74276274	A	G	G	A	0.10	-0.24	0.32	0.42
4-Hydroxyproline galactoside	8:36333177	C	T	T	C	0.08	-0.28	-0.20	0.54
4-Hydroxyproline galactoside	8:36354692	C	A	A	C	0.07	-0.30	-0.36	0.24
4-Hydroxyproline galactoside	8:36373022	G	C	C	G	0.08	-0.31	-0.16	0.64
4-Hydroxyproline galactoside	8:36398546	G	C	C	G	0.09	-0.25	-0.36	0.28
4-Hydroxyproline galactoside	8:36408820	T	C	C	T	0.08	-0.29	-0.57	0.11
4-Hydroxyproline galactoside	8:36441814	G	T	T	G	0.08	-0.32	-0.41	0.19
4-Hydroxyproline galactoside	8:36461053	T	G	G	T	0.09	-0.26	-0.31	0.37
4-Hydroxyproline galactoside	8:36558263	C	T	T	C	0.09	-0.30	-0.26	0.44
4-Hydroxyproline galactoside	8:36578418	A	C	C	A	0.10	-0.28	-0.43	0.15
4-Hydroxyproline galactoside	8:36584219	C	T	T	C	0.08	-0.28	-0.30	0.32
4-Hydroxyproline galactoside	8:36584529	C	T	T	C	0.09	-0.31	-0.61	0.06
4-Hydroxyproline galactoside	8:36587603	G	A	A	G	0.09	-0.25	-0.27	0.32
4-Hydroxyproline galactoside	8:36588714	C	T	T	C	0.09	-0.26	-0.43	0.21

4-Hydroxyproline galactoside	8:36594279	A	G	G	A	0.08	-0.28	-0.65	0.03
4-Hydroxyproline galactoside	8:36598207	A	G	G	A	0.08	-0.28	-0.21	0.49
4-Hydroxyproline galactoside	8:85629643	C	T	T	C	0.08	-0.26	-0.55	0.11
4-Hydroxyproline galactoside	9:92013861	C	T	T	C	0.10	-0.24	0.22	0.43
4-Hydroxyproline galactoside	9:92013870	C	A	A	C	0.10	-0.23	0.22	0.44
4-Hydroxyproline galactoside	9:92013878	G	A	A	G	0.10	-0.23	0.22	0.44
Niflumic Acid	1:139843617	A	G	G	A	0.13	-0.22	-0.05	0.88
Niflumic Acid	1:139843618	C	T	T	C	0.13	-0.22	-0.05	0.88
Niflumic Acid	10:23726234	C	A	A	C	0.10	-0.27	-0.13	0.69
Niflumic Acid	10:27863087	A	G	G	A	0.09	-0.28	-0.66	0.02
Niflumic Acid	11:102451092	A	G	G	A	0.05	-0.32	-0.35	0.22
Niflumic Acid	12:49555887	A	T	T	A	0.09	-0.27	-0.24	0.42
Niflumic Acid	15:54961497	C	T	T	C	0.13	-0.25	-0.45	0.11
Niflumic Acid	15:54961500	G	A	A	G	0.13	-0.25	-0.36	0.22
Niflumic Acid	15:54961503	C	T	T	C	0.12	-0.26	-0.29	0.35
Niflumic Acid	15:54961511	T	C	C	T	0.11	-0.29	-0.25	0.47
Niflumic Acid	15:54961513	G	A	A	G	0.11	-0.28	-0.13	0.73
Niflumic Acid	15:54961515	T	C	C	T	0.11	-0.27	-0.13	0.73
Niflumic Acid	15:77722146	A	G	G	A	0.07	-0.35	0.09	0.76
Niflumic Acid	15:77722158	T	C	C	T	0.07	-0.37	0.11	0.71
Niflumic Acid	19:15805634	A	G	G	A	0.05	-0.33	0.03	0.93
Niflumic Acid	19:15812999	T	C	C	T	0.06	-0.29	-0.32	0.42
Niflumic Acid	19:15813009	G	A	A	G	0.06	-0.29	-0.29	0.46
Niflumic Acid	19:15813025	C	T	T	C	0.07	-0.32	-0.10	0.79
Niflumic Acid	19:15819566	A	G	G	A	0.07	-0.30	-0.24	0.51
Niflumic Acid	19:15819709	T	G	G	T	0.07	-0.31	-0.25	0.49
Niflumic Acid	19:15819726	C	T	T	C	0.06	-0.34	-0.12	0.75
Niflumic Acid	2:37279642	T	C	C	T	0.05	-0.38	-0.28	0.47
Niflumic Acid	23:25678525	G	A	A	G	0.09	-0.30	-0.22	0.52
Niflumic Acid	23:26005939	T	A	A	T	0.10	-0.33	-0.29	0.48
Niflumic Acid	23:26005946	T	A	A	T	0.10	-0.30	-0.29	0.47
Niflumic Acid	23:3918252	G	A	A	G	0.10	-0.25	0.01	0.98
Niflumic Acid	23:5964357	G	A	A	G	0.07	-0.32	-0.02	0.96
Niflumic Acid	3:62414486	C	T	T	C	0.15	-0.21	-0.13	0.59
Niflumic Acid	6:115346914	G	A	A	G	0.10	-0.28	-0.39	0.17
Niflumic Acid	7:31189140	C	T	T	C	0.10	-0.28	-0.20	0.53
2',3'-Didehydro-2',3'-dideoxycytidine	1:31755732	A	G	G	A	0.06	-0.63	-0.52	0.16
2',3'-Didehydro-2',3'-dideoxycytidine	1:31798727	C	A	A	C	0.08	-0.56	-0.17	0.61
2',3'-Didehydro-2',3'-dideoxycytidine	1:39673923	C	T	T	C	0.11	-0.48	-0.07	0.82
2',3'-Didehydro-2',3'-dideoxycytidine	1:571864	C	G	G	C	0.09	-0.58	-0.20	0.61
2',3'-Didehydro-2',3'-dideoxycytidine	10:1208645	G	A	A	G	0.14	-0.43	-0.57	0.03
2',3'-Didehydro-2',3'-dideoxycytidine	10:22662067	C	T	T	C	0.10	-0.45	-0.51	0.11
2',3'-Didehydro-2',3'-dideoxycytidine	10:22787430	T	C	C	T	0.06	-0.80	-0.51	0.23
2',3'-Didehydro-2',3'-dideoxycytidine	10:22798942	A	G	G	A	0.06	-0.68	0.36	0.28

2',3'-Didehydro-2',3'-dideoxycytidine	10:27554851	G	A	A	G	0.08	-0.55	-0.20	0.51
2',3'-Didehydro-2',3'-dideoxycytidine	10:27554870	A	G	G	A	0.08	-0.56	-0.15	0.61
2',3'-Didehydro-2',3'-dideoxycytidine	10:27823770	C	T	T	C	0.09	-0.52	-0.09	0.79
2',3'-Didehydro-2',3'-dideoxycytidine	10:53560918	T	C	C	T	0.05	-0.66	-0.50	0.22
2',3'-Didehydro-2',3'-dideoxycytidine	10:53560919	C	T	T	C	0.05	-0.66	-0.50	0.22
2',3'-Didehydro-2',3'-dideoxycytidine	10:54110863	T	G	G	T	0.07	-0.65	-0.49	0.14
2',3'-Didehydro-2',3'-dideoxycytidine	10:57781228	C	T	T	C	0.06	-0.76	-0.59	0.10
2',3'-Didehydro-2',3'-dideoxycytidine	10:63676540	A	G	G	A	0.10	-0.52	-0.14	0.58
2',3'-Didehydro-2',3'-dideoxycytidine	10:68197393	G	A	A	G	0.08	-0.57	0.20	0.53
2',3'-Didehydro-2',3'-dideoxycytidine	11:1868597	G	T	T	G	0.08	-0.52	-0.20	0.43
2',3'-Didehydro-2',3'-dideoxycytidine	11:19188387	C	T	T	C	0.07	-0.63	-0.15	0.61
2',3'-Didehydro-2',3'-dideoxycytidine	11:47176729	C	A	A	C	0.08	-0.67	-0.28	0.50
2',3'-Didehydro-2',3'-dideoxycytidine	11:47176749	C	G	G	C	0.09	-0.59	-0.12	0.76
2',3'-Didehydro-2',3'-dideoxycytidine	11:70892722	T	C	C	T	0.07	-0.53	-0.27	0.31
2',3'-Didehydro-2',3'-dideoxycytidine	12:11027645	T	C	C	T	0.11	-0.49	0.11	0.66
2',3'-Didehydro-2',3'-dideoxycytidine	12:50147120	A	G	G	A	0.07	-0.54	-0.76	0.08
2',3'-Didehydro-2',3'-dideoxycytidine	12:50154873	C	T	T	C	0.08	-0.53	-0.20	0.52
2',3'-Didehydro-2',3'-dideoxycytidine	12:74815513	C	T	T	C	0.05	-0.65	-0.27	0.47
2',3'-Didehydro-2',3'-dideoxycytidine	13:11068632	A	G	G	A	0.08	-0.62	-0.10	0.78
2',3'-Didehydro-2',3'-dideoxycytidine	13:20779369	T	A	A	T	0.07	-0.84	-0.62	0.09
2',3'-Didehydro-2',3'-dideoxycytidine	13:27906668	C	T	T	C	0.14	-0.51	-0.23	0.35
2',3'-Didehydro-2',3'-dideoxycytidine	13:30214270	T	C	C	T	0.10	-0.48	-0.01	0.98
2',3'-Didehydro-2',3'-dideoxycytidine	13:31918921	G	A	A	G	0.08	-0.63	-0.20	0.55
2',3'-Didehydro-2',3'-dideoxycytidine	13:31958991	G	A	A	G	0.07	-0.83	0.09	0.78
2',3'-Didehydro-2',3'-dideoxycytidine	13:32116900	T	A	A	T	0.08	-0.54	-0.16	0.60
2',3'-Didehydro-2',3'-dideoxycytidine	13:77864663	G	A	A	G	0.08	-0.52	-0.23	0.46
2',3'-Didehydro-2',3'-dideoxycytidine	15:29991182	A	T	T	A	0.11	-0.54	-0.23	0.49
2',3'-Didehydro-2',3'-dideoxycytidine	15:77723212	C	T	T	C	0.05	-0.76	0.11	0.74
2',3'-Didehydro-2',3'-dideoxycytidine	16:20580150	C	T	T	C	0.09	-0.50	0.23	0.43
2',3'-Didehydro-2',3'-dideoxycytidine	16:23299546	A	G	G	A	0.05	-0.67	-0.53	0.16
2',3'-Didehydro-2',3'-dideoxycytidine	16:24013766	T	C	C	T	0.07	-0.61	-0.20	0.63
2',3'-Didehydro-2',3'-dideoxycytidine	16:24018592	G	A	A	G	0.05	-0.69	0.02	0.95
2',3'-Didehydro-2',3'-dideoxycytidine	16:24116738	T	C	C	T	0.05	-0.63	-0.63	0.12
2',3'-Didehydro-2',3'-dideoxycytidine	16:5874791	G	A	A	G	0.07	-0.63	0.36	0.34
2',3'-Didehydro-2',3'-dideoxycytidine	16:70415122	C	A	A	C	0.07	-0.58	-0.32	0.32
2',3'-Didehydro-2',3'-dideoxycytidine	17:49465102	A	G	G	A	0.10	-0.62	0.28	0.42
2',3'-Didehydro-2',3'-dideoxycytidine	17:6048558	T	C	C	T	0.08	-0.55	-0.61	0.09
2',3'-Didehydro-2',3'-dideoxycytidine	17:65758081	A	G	G	A	0.07	-0.61	-0.05	0.87
2',3'-Didehydro-2',3'-dideoxycytidine	17:65759627	C	T	T	C	0.11	-0.48	-0.13	0.62
2',3'-Didehydro-2',3'-dideoxycytidine	17:65982514	C	A	A	C	0.09	-0.52	-0.35	0.24
2',3'-Didehydro-2',3'-dideoxycytidine	17:65988472	G	A	A	G	0.08	-0.54	0.09	0.78
2',3'-Didehydro-2',3'-dideoxycytidine	17:66034560	C	T	T	C	0.08	-0.54	-0.31	0.31
2',3'-Didehydro-2',3'-dideoxycytidine	17:66215786	A	G	G	A	0.06	-0.56	-0.18	0.60
2',3'-Didehydro-2',3'-dideoxycytidine	17:66238632	A	G	G	A	0.07	-0.55	-0.15	0.64

2',3'-Didehydro-2',3'-dideoxycytidine	17:66239174	T	C	C	T	0.08	-0.67	-0.18	0.59
2',3'-Didehydro-2',3'-dideoxycytidine	17:68737910	A	C	C	A	0.05	-0.68	-0.34	0.39
2',3'-Didehydro-2',3'-dideoxycytidine	18:17943043	C	T	T	C	0.08	-0.53	-0.14	0.75
2',3'-Didehydro-2',3'-dideoxycytidine	18:29691725	G	A	A	G	0.05	-0.61	-0.29	0.49
2',3'-Didehydro-2',3'-dideoxycytidine	18:31190107	T	C	C	T	0.06	-0.61	-0.24	0.41
2',3'-Didehydro-2',3'-dideoxycytidine	18:37596243	A	G	G	A	0.08	-0.64	-0.04	0.91
2',3'-Didehydro-2',3'-dideoxycytidine	19:1451603	T	A	A	T	0.05	-1.09	-0.27	0.46
2',3'-Didehydro-2',3'-dideoxycytidine	19:15670223	G	A	A	G	0.05	-0.70	-0.15	0.63
2',3'-Didehydro-2',3'-dideoxycytidine	19:1694500	C	T	T	C	0.08	-0.66	-0.83	0.01
2',3'-Didehydro-2',3'-dideoxycytidine	19:1957200	T	A	A	T	0.11	-0.53	-0.67	0.03
2',3'-Didehydro-2',3'-dideoxycytidine	19:6096274	T	C	C	T	0.09	-0.65	-0.47	0.14
2',3'-Didehydro-2',3'-dideoxycytidine	19:6097957	T	C	C	T	0.09	-0.51	-0.09	0.79
2',3'-Didehydro-2',3'-dideoxycytidine	2:1044798	C	G	G	C	0.17	-0.36	-0.13	0.56
2',3'-Didehydro-2',3'-dideoxycytidine	2:1086196	C	A	A	C	0.07	-0.50	-0.05	0.87
2',3'-Didehydro-2',3'-dideoxycytidine	2:115511060	G	A	A	G	0.05	-0.72	-0.49	0.18
2',3'-Didehydro-2',3'-dideoxycytidine	2:19187277	T	C	C	T	0.05	-0.85	-0.31	0.34
2',3'-Didehydro-2',3'-dideoxycytidine	2:22601448	T	C	C	T	0.08	-0.62	-0.87	0.01
2',3'-Didehydro-2',3'-dideoxycytidine	2:39960633	T	C	C	T	0.12	-0.51	-0.75	0.01
2',3'-Didehydro-2',3'-dideoxycytidine	20:23352370	G	A	A	G	0.19	-0.35	0.00	0.99
2',3'-Didehydro-2',3'-dideoxycytidine	20:23394824	C	A	A	C	0.09	-0.61	-0.42	0.29
2',3'-Didehydro-2',3'-dideoxycytidine	20:23394825	T	A	A	T	0.09	-0.61	-0.41	0.30
2',3'-Didehydro-2',3'-dideoxycytidine	20:25029301	G	T	T	G	0.09	-0.58	-0.06	0.85
2',3'-Didehydro-2',3'-dideoxycytidine	20:25047656	A	C	C	A	0.09	-0.65	0.09	0.81
2',3'-Didehydro-2',3'-dideoxycytidine	20:25713918	C	T	T	C	0.10	-0.51	0.46	0.24
2',3'-Didehydro-2',3'-dideoxycytidine	20:41534233	T	C	C	T	0.10	-0.52	0.11	0.74
2',3'-Didehydro-2',3'-dideoxycytidine	20:47322831	C	A	A	C	0.08	-0.52	-0.61	0.11
2',3'-Didehydro-2',3'-dideoxycytidine	20:47528609	C	T	T	C	0.09	-0.58	0.19	0.60
2',3'-Didehydro-2',3'-dideoxycytidine	20:47604296	G	A	A	G	0.09	-0.54	-0.01	0.97
2',3'-Didehydro-2',3'-dideoxycytidine	21:27860121	C	A	A	C	0.08	-0.54	-0.15	0.69
2',3'-Didehydro-2',3'-dideoxycytidine	21:32997580	G	A	A	G	0.14	-0.45	-0.08	0.78
2',3'-Didehydro-2',3'-dideoxycytidine	21:52346839	G	A	A	G	0.06	-0.67	-0.42	0.23
2',3'-Didehydro-2',3'-dideoxycytidine	21:59127223	C	T	T	C	0.06	-0.66	0.16	0.67
2',3'-Didehydro-2',3'-dideoxycytidine	21:60157551	G	C	C	G	0.13	-0.43	-0.29	0.34
2',3'-Didehydro-2',3'-dideoxycytidine	21:60157554	G	C	C	G	0.13	-0.43	-0.31	0.31
2',3'-Didehydro-2',3'-dideoxycytidine	23:15086177	T	G	G	T	0.09	-0.49	-0.41	0.07
2',3'-Didehydro-2',3'-dideoxycytidine	23:16922	T	C	C	T	0.07	-0.81	0.41	0.35
2',3'-Didehydro-2',3'-dideoxycytidine	23:27308455	G	A	A	G	0.05	-0.63	-0.73	0.03
2',3'-Didehydro-2',3'-dideoxycytidine	23:32693522	G	T	T	G	0.06	-0.63	0.05	0.88
2',3'-Didehydro-2',3'-dideoxycytidine	23:42597	G	A	A	G	0.14	-0.51	0.37	0.27
2',3'-Didehydro-2',3'-dideoxycytidine	23:47866599	T	G	G	T	0.06	-0.71	0.24	0.55
2',3'-Didehydro-2',3'-dideoxycytidine	23:49015627	C	A	A	C	0.06	-0.73	0.11	0.74
2',3'-Didehydro-2',3'-dideoxycytidine	23:49193455	C	T	T	C	0.08	-0.59	-0.11	0.74
2',3'-Didehydro-2',3'-dideoxycytidine	23:49215414	A	G	G	A	0.06	-0.67	0.03	0.92
2',3'-Didehydro-2',3'-dideoxycytidine	23:49219165	T	C	C	T	0.10	-0.49	-0.12	0.69

2',3'-Didehydro-2',3'-dideoxycytidine	23:49219178	G	A	A	G	0.10	-0.48	-0.14	0.64
2',3'-Didehydro-2',3'-dideoxycytidine	23:49219183	A	C	C	A	0.10	-0.45	-0.15	0.62
2',3'-Didehydro-2',3'-dideoxycytidine	23:49271989	C	G	G	C	0.06	-0.65	0.24	0.50
2',3'-Didehydro-2',3'-dideoxycytidine	23:49456698	C	T	T	C	0.06	-0.58	0.19	0.55
2',3'-Didehydro-2',3'-dideoxycytidine	23:49526562	A	G	G	A	0.06	-0.59	-0.05	0.90
2',3'-Didehydro-2',3'-dideoxycytidine	23:50351809	A	G	G	A	0.06	-0.65	-0.39	0.19
2',3'-Didehydro-2',3'-dideoxycytidine	23:51566026	G	A	A	G	0.06	-0.69	0.43	0.25
2',3'-Didehydro-2',3'-dideoxycytidine	23:51927363	T	C	C	T	0.07	-0.59	-0.35	0.36
2',3'-Didehydro-2',3'-dideoxycytidine	27:6433745	T	C	C	T	0.08	-0.72	-0.48	0.18
2',3'-Didehydro-2',3'-dideoxycytidine	29:5759592	G	A	A	G	0.13	-0.54	-0.05	0.87
2',3'-Didehydro-2',3'-dideoxycytidine	30:20754292	G	A	A	G	0.08	-0.57	0.53	0.16
2',3'-Didehydro-2',3'-dideoxycytidine	30:4650149	A	G	G	A	0.07	-0.66	-0.38	0.26
2',3'-Didehydro-2',3'-dideoxycytidine	4:103673218	G	A	A	G	0.09	-0.54	0.03	0.92
2',3'-Didehydro-2',3'-dideoxycytidine	4:107453664	C	A	A	C	0.06	-0.57	0.08	0.85
2',3'-Didehydro-2',3'-dideoxycytidine	4:107453666	A	G	G	A	0.06	-0.56	0.08	0.84
2',3'-Didehydro-2',3'-dideoxycytidine	4:107888868	G	A	A	G	0.09	-0.48	0.25	0.45
2',3'-Didehydro-2',3'-dideoxycytidine	4:108011942	A	G	G	A	0.11	-0.40	0.20	0.46
2',3'-Didehydro-2',3'-dideoxycytidine	4:110518989	C	T	T	C	0.07	-0.56	0.42	0.26
2',3'-Didehydro-2',3'-dideoxycytidine	4:110609536	G	A	A	G	0.05	-0.65	0.39	0.34
2',3'-Didehydro-2',3'-dideoxycytidine	4:111196346	G	A	A	G	0.09	-0.53	0.05	0.86
2',3'-Didehydro-2',3'-dideoxycytidine	4:111197047	A	C	C	A	0.08	-0.56	-0.14	0.66
2',3'-Didehydro-2',3'-dideoxycytidine	4:111230521	A	C	C	A	0.06	-0.72	-0.16	0.66
2',3'-Didehydro-2',3'-dideoxycytidine	4:111231805	G	T	T	G	0.07	-0.59	-0.01	0.97
2',3'-Didehydro-2',3'-dideoxycytidine	4:111231934	C	A	A	C	0.09	-0.52	0.09	0.76
2',3'-Didehydro-2',3'-dideoxycytidine	4:111245883	C	T	T	C	0.08	-0.62	-0.24	0.47
2',3'-Didehydro-2',3'-dideoxycytidine	4:111245884	A	G	G	A	0.11	-0.56	-0.14	0.63
2',3'-Didehydro-2',3'-dideoxycytidine	4:111262131	G	A	A	G	0.09	-0.50	0.09	0.78
2',3'-Didehydro-2',3'-dideoxycytidine	4:111262146	G	C	C	G	0.09	-0.50	0.10	0.74
2',3'-Didehydro-2',3'-dideoxycytidine	4:111300251	A	C	C	A	0.09	-0.50	-0.03	0.92
2',3'-Didehydro-2',3'-dideoxycytidine	4:111486721	T	G	G	T	0.06	-0.59	0.32	0.32
2',3'-Didehydro-2',3'-dideoxycytidine	4:112123767	G	A	A	G	0.06	-0.60	-0.33	0.31
2',3'-Didehydro-2',3'-dideoxycytidine	4:112135363	C	T	T	C	0.08	-0.54	0.06	0.82
2',3'-Didehydro-2',3'-dideoxycytidine	4:118940988	C	G	G	C	0.06	-0.59	-0.33	0.30
2',3'-Didehydro-2',3'-dideoxycytidine	4:25364792	C	T	T	C	0.05	-0.65	0.02	0.96
2',3'-Didehydro-2',3'-dideoxycytidine	4:82544449	G	A	A	G	0.10	-0.51	-0.18	0.47
2',3'-Didehydro-2',3'-dideoxycytidine	4:82670843	T	G	G	T	0.14	-0.42	-0.47	0.09
2',3'-Didehydro-2',3'-dideoxycytidine	4:82675966	C	T	T	C	0.06	-0.66	-0.22	0.56
2',3'-Didehydro-2',3'-dideoxycytidine	4:82824368	A	G	G	A	0.06	-0.69	-0.35	0.38
2',3'-Didehydro-2',3'-dideoxycytidine	4:98883991	C	A	A	C	0.08	-0.47	0.07	0.84
2',3'-Didehydro-2',3'-dideoxycytidine	5:36242094	A	T	T	A	0.06	-0.71	0.19	0.61
2',3'-Didehydro-2',3'-dideoxycytidine	5:59745953	T	C	C	T	0.06	-0.78	-0.11	0.81
2',3'-Didehydro-2',3'-dideoxycytidine	5:99146504	T	C	C	T	0.12	-0.49	0.46	0.14
2',3'-Didehydro-2',3'-dideoxycytidine	7:65108143	A	G	G	A	0.06	-0.55	-0.10	0.79
2',3'-Didehydro-2',3'-dideoxycytidine	7:69580511	A	T	T	A	0.08	-0.51	0.24	0.53

2',3'-Didehydro-2',3'-dideoxycytidine	7:72650846	A	G	G	A	0.15	-0.48	0.08	0.75
2',3'-Didehydro-2',3'-dideoxycytidine	7:74309898	C	T	T	C	0.05	-0.57	0.24	0.51
2',3'-Didehydro-2',3'-dideoxycytidine	8:60111282	C	T	T	C	0.09	-0.59	-0.04	0.90
2',3'-Didehydro-2',3'-dideoxycytidine	9:104036241	C	T	T	C	0.06	-0.73	-0.21	0.49
2',3'-Didehydro-2',3'-dideoxycytidine	9:47042792	A	T	T	A	0.13	-0.42	-0.37	0.15
2',3'-Didehydro-2',3'-dideoxycytidine	9:99365062	C	T	T	C	0	-0.4776655	0	0.070

Table S10. Two-sample MR identifying the causal associations between rumen metabolites and prevotella

id.exposure (IVW)	Prevotella_bryantii			Prevotella_mizrahii			Prevotella_sp._AGR2160		
	b	se	FDR	b	se	FDR	b	se	FDR
2',3'-Didehydro-2',3'-dideoxycytidine	0.31	0.06	0.00	0.14	0.03	0.00	0.30	0.06	0.00
Linalool (8-hydroxydihydro-)	0.58	0.42	0.17	0.33	0.18	0.07	0.50	0.39	0.43
Myo-Inositol	0.13	0.51	0.80	-0.08	0.24	0.75	-0.05	0.49	0.97
Niflumic Acid	0.91	0.31	0.00	0.40	0.14	0.00	0.81	0.29	0.04
3,4-Dihydroxy-2-hydroxymethyl-1-pyrrolidinepropanamide	0.11	0.33	0.75	-0.01	0.19	0.96	0.17	0.31	0.78
N-formimidoyl-glutamic acid	0.23	0.24	0.33	0.03	0.14	0.83	0.15	0.23	0.72
4-Oxododecanedioic acid	2.10	0.81	0.01	0.44	0.38	0.26	1.84	0.77	0.09
(-)-11-Hydroxy-9,15,16-trioxooctadecanoic acid	0.94	1.18	0.42	0.25	0.57	0.66	0.71	1.17	0.75
MG(0:0/14:1(9Z)/0:0)	1.49	1.66	0.37	1.12	0.79	0.16	1.32	1.58	0.61
2,2'-(3-methylcyclohexane-1,1-diyl)diacetic acid	2.10	2.53	0.41	1.38	1.20	0.25	2.50	2.42	0.54
(9S,10S)-9,10-dihydroxyoctadecanoate	1.01	0.43	0.02	0.48	0.24	0.04	0.92	0.42	0.10
SM(d16:2(4E,8Z)/20:5(6E,8Z,11Z,14Z,17Z)-OH(5))	0.06	0.07	0.37	0.02	0.03	0.62	0.06	0.07	0.59
4-Hydroxyproline galactoside	1.30	0.38	0.00	0.44	0.18	0.01	1.26	0.37	0.01
Guadecitabine	-0.01	0.05	0.81	-0.02	0.03	0.52	-0.03	0.05	0.82
N(G)-Nitroarginine-4-nitroanilide	-0.18	0.12	0.14	-0.01	0.06	0.90	-0.13	0.12	0.49
1,2,3,4,5,6-Hexahydro-5-methyl-7H-cyclopenta[b]pyridin-7-one	-0.52	0.19	0.01	-0.24	0.09	0.00	-0.46	0.18	0.07
Gladiatoside C2	-0.10	0.09	0.28	-0.04	0.04	0.31	-0.07	0.08	0.60
Betanin	-0.22	0.13	0.10	-0.09	0.07	0.24	-0.23	0.13	0.25
Junosine	-0.22	0.09	0.01	-0.14	0.04	0.00	-0.19	0.09	0.10
5-Ethyl-1,2,3,4,5,6-hexahydro-7H-cyclopenta[b]pyridin-7-one	-0.20	0.06	0.00	-0.07	0.03	0.01	-0.17	0.06	0.03
6-Hydroxysandoricin	0.74	0.57	0.19	0.33	0.27	0.22	0.69	0.54	0.43
D-Gluconic acid	0.26	0.28	0.36	0.08	0.13	0.57	0.10	0.27	0.84
Undecanedioic acid	-0.14	0.69	0.84	0.20	0.33	0.54	-0.21	0.66	0.86
4-Heptyloxyphenol	0.09	0.27	0.74	0.03	0.13	0.79	0.10	0.26	0.84
Asparaginylaspartic acid	0.45	0.37	0.22	0.25	0.17	0.13	0.29	0.36	0.62
Oxypurinol	0.73	0.29	0.01	0.18	0.14	0.21	0.61	0.27	0.10
Histidinyl-Asparagine	0.64	0.43	0.14	0.07	0.18	0.70	0.45	0.39	0.48
Zilascorb	0.66	0.31	0.03	0.26	0.15	0.07	0.68	0.30	0.10
Ethoxyquin	0.16	0.21	0.45	0.10	0.10	0.32	0.20	0.20	0.54
Taurochenodesoxycholic acid	0.28	0.16	0.07	0.05	0.07	0.47	0.31	0.15	0.13
25-Acetylvulgaroside	0.57	0.19	0.00	0.33	0.09	0.00	0.53	0.17	0.02
16-Hydroxyhexadecanoylcarnitine	0.43	0.34	0.21	0.07	0.16	0.65	0.42	0.32	0.42
DTMP	0.02	0.07	0.78	0.02	0.04	0.61	0.00	0.07	0.99
Val Leu Gly	0.04	0.02	0.03	0.03	0.01	0.00	0.07	0.02	0.01
LysoPS(16:0/0:0)	0.05	0.11	0.62	0.07	0.05	0.20	0.10	0.10	0.54
3'-demethyletoposide	0.06	0.07	0.37	0.09	0.04	0.02	-0.03	0.07	0.84

[3-[2-Aminoethoxy(hydroxy)phosphoryl]oxy-2-hydroxypropyl] hexadecanoate	0.44	0.70	0.52	-0.08	0.33	0.82	0.46	0.66	0.69
1,2,3-Benzotriazine	-0.16	0.11	0.14	-0.01	0.05	0.79	-0.18	0.10	0.22
Cynaropicrin	0.02	0.03	0.51	0.02	0.02	0.25	0.04	0.03	0.40
D-Galactose	0.86	0.45	0.06	0.07	0.21	0.75	0.56	0.43	0.42
D-Glucamine	0.42	0.23	0.07	0.31	0.10	0.00	0.52	0.24	0.10
Vidarabine	0.46	0.20	0.02	0.08	0.11	0.47	0.42	0.19	0.10
Sudan I	0.11	0.05	0.02	0.05	0.02	0.01	0.11	0.04	0.05
Gly-Ile	-1.20	0.50	0.02	-0.36	0.28	0.19	-1.19	0.49	0.08
Lotaustralin	-0.67	0.17	0.00	-0.17	0.08	0.03	-0.56	0.16	0.01
Glycyl-leucine	-0.07	1.56	0.96	0.15	0.80	0.85	0.03	1.53	0.99
Acrylamide-sodium acrylate resin	-0.15	0.69	0.83	0.14	0.33	0.67	-0.20	0.66	0.86
Valeraldehyde propyleneglycol acetal	0.44	0.51	0.39	0.44	0.24	0.07	0.70	0.49	0.36
Adenosine	0.38	0.29	0.19	-0.02	0.15	0.90	0.32	0.27	0.47
Asparaginyl-Proline	-0.28	0.33	0.39	-0.24	0.15	0.12	-0.41	0.31	0.42
Glutamylarginine	-0.05	0.06	0.36	-0.05	0.03	0.08	-0.02	0.05	0.86
N-[4-[Acetyl(3-aminopropyl)amino]butyl]-N-(3-aminopropyl)acetamide	-0.18	0.23	0.43	0.07	0.09	0.44	-0.19	0.22	0.59
Lys Val	0.19	0.08	0.01	0.06	0.04	0.08	0.23	0.07	0.02
D-fructose 6-phosphate	0.28	0.36	0.44	0.09	0.17	0.59	0.34	0.34	0.54
Miserotoxin	0.21	0.06	0.00	0.08	0.04	0.03	0.19	0.06	0.02
N1-Acetylspermine	-0.16	0.09	0.08	-0.02	0.04	0.63	-0.14	0.09	0.29
Levulinic acid	1.96	1.56	0.21	1.11	0.68	0.10	1.95	1.42	0.40
2',3' cyclic CMP	0.01	0.17	0.93	0.15	0.08	0.06	0.06	0.16	0.84
2-amino-3,7-dideoxy-D-threo-hept-6-ulosonate	-0.08	0.26	0.74	-0.02	0.12	0.90	-0.15	0.25	0.75
Alanylisoleucine	-0.74	0.91	0.42	-0.01	0.43	0.98	-0.89	0.86	0.54
Guanosine	0.33	0.16	0.04	0.06	0.08	0.44	0.29	0.15	0.18
DG(2:0/0:0/18:3(10,12,15)-OH(9))	1.48	2.15	0.49	0.72	0.94	0.44	1.60	2.05	0.63
Cytarabine	1.00	0.41	0.02	0.24	0.20	0.22	0.89	0.40	0.10
LysoPE(18:3(6Z,9Z,12Z)/0:0)	0.16	0.07	0.01	0.00	0.03	0.87	0.16	0.06	0.06
Prostaglandin PGE2 1-glyceryl ester	0.77	2.01	0.70	1.34	0.95	0.16	0.85	1.91	0.84
1-(4Z,7Z,10Z,13Z,16Z,19Z-docosahexaenoyl)-glycero-3-phosphate	-0.01	0.05	0.82	0.03	0.02	0.17	0.00	0.05	0.99
Ganoderic acid F	0.25	0.35	0.48	0.04	0.23	0.87	0.16	0.33	0.84
Eriodictyol 7-(6-trans-p-coumaroylglucoside)	-0.05	0.05	0.33	-0.02	0.02	0.49	-0.03	0.05	0.71
1-Naphthylamine	-0.22	0.67	0.74	-0.12	0.47	0.79	-0.19	0.64	0.86
Monoacetyldiglyceride	0.25	0.24	0.31	0.12	0.11	0.29	0.25	0.22	0.49
5-Methyl-3'-Deoxyuridine	-0.20	0.30	0.51	0.04	0.14	0.76	0.02	0.29	0.98
6-Acetyl-1,2,3,4-tetrahydropyridine	0.37	0.91	0.68	0.39	0.42	0.35	0.24	0.87	0.86
4-(3-Hydroxypropyl)morpholine	0.13	0.57	0.82	0.16	0.23	0.50	0.25	0.53	0.84
2-(4-Amino-1-isopropyl-1H-pyrazolo[3,4-d]pyrimidin-3-yl)-1H-indol-5-ol	-0.08	0.23	0.74	0.05	0.11	0.64	0.03	0.22	0.97
L-phenylalanyl-L-proline	1.10	0.67	0.10	0.12	0.32	0.69	1.07	0.64	0.26
Serratol	-0.41	0.14	0.00	-0.13	0.07	0.05	-0.42	0.13	0.02

D-Citrulline	-0.68	0.09	0.00	-0.50	0.04	0.00	-0.68	0.09	0.00
Vadocaine	-0.14	0.14	0.30	-0.07	0.06	0.27	-0.12	0.13	0.59
Dhv-PGE2	-1.54	0.75	0.04	-0.28	0.32	0.38	-1.29	0.76	0.25
LysoPC(14:0/0:0)	0.46	0.21	0.03	-0.28	0.10	0.00	0.46	0.20	0.10
Pheophorbide a	0.10	0.09	0.24	0.05	0.04	0.21	0.08	0.08	0.55
Glycyl-D-Alanine	0.06	0.52	0.90	-0.07	0.24	0.79	0.14	0.49	0.86
6-Hydroxymelatonin	2.79	0.99	0.00	1.23	0.47	0.01	3.20	0.95	0.01
PE(16:0/0:0)	0.63	0.32	0.04	0.28	0.14	0.04	0.50	0.30	0.27
Vaccinoside	0.54	0.75	0.47	-0.04	0.35	0.91	0.61	0.71	0.60
4-hydroxy Nonenal	-1.33	2.75	0.63	-0.43	1.11	0.70	-1.17	2.72	0.84
9-Hydroxylinoleic acid	1.25	0.88	0.15	0.91	0.42	0.03	1.32	0.84	0.30
5(S),14(R)-Lipoxin B4	0.17	1.00	0.87	-0.27	0.47	0.56	0.08	0.95	0.98
2,6-diamino-4-hydroxy-5-formamidopyrimidine	0.42	0.20	0.04	0.25	0.09	0.01	0.38	0.20	0.17
Patulin	0.57	0.17	0.00	0.16	0.08	0.04	0.53	0.16	0.01
Trigonelline	0.30	0.99	0.76	0.43	0.47	0.36	0.31	0.94	0.86
FAPy-adenine	-0.11	0.61	0.86	-0.37	0.34	0.28	-0.24	0.60	0.84
2,3-dinor-8-iso-PGF2a	2.12	1.43	0.14	0.37	0.65	0.57	1.61	1.40	0.48
1-(5Z,8Z,11Z,14Z-eicosatetraenoyl)-sn-glycero-3-phosphate	-0.30	0.04	0.00	-0.23	0.02	0.00	-0.29	0.04	0.00
Diosbulbinoside F	0.18	0.13	0.15	0.09	0.06	0.12	0.23	0.12	0.18
Tuliposide A	0.06	0.19	0.75	-0.08	0.09	0.41	0.04	0.18	0.88
8-Aminoguanosine	1.59	0.63	0.01	0.43	0.30	0.15	1.26	0.60	0.13
Beta-Thujaplicin	0.09	0.23	0.71	-0.15	0.11	0.18	0.08	0.22	0.84
Arginyllysine	-0.14	0.35	0.68	-0.09	0.16	0.60	-0.07	0.33	0.89
Nandrolone	0.31	0.27	0.24	0.09	0.13	0.46	0.45	0.25	0.22
3-Indolepropionic acid	-0.57	0.28	0.04	0.08	0.13	0.54	-0.34	0.27	0.43
Val Ile Ile	0.39	0.46	0.39	-0.07	0.22	0.76	0.35	0.44	0.63
1,5-Isoquinolinediol	0.80	0.23	0.00	0.35	0.11	0.00	0.71	0.22	0.01
Dehydrophytosphingosine	0.41	0.38	0.29	0.31	0.16	0.05	0.42	0.35	0.46
4-Hydroxy-3-polyprenylbenzoate	0.67	0.22	0.00	0.31	0.10	0.00	0.72	0.21	0.01
9,10-Epoxyoctadecanoic acid	-0.26	0.76	0.74	0.53	0.36	0.14	-0.02	0.73	0.99
Tryptophyl-Serine	-0.28	0.26	0.29	-0.09	0.12	0.46	-0.24	0.25	0.55
P-Menthane-3,8-diol	1.56	1.28	0.22	0.86	0.42	0.04	1.15	1.23	0.56
Gamma-Glutamyl-beta-aminopropiononitrile	0.13	0.59	0.83	-0.15	0.26	0.56	0.00	0.52	1.00
Histidylcysteine	0.29	0.44	0.50	0.11	0.21	0.59	0.16	0.42	0.84
DL-Allylglycine	0.19	0.15	0.19	0.08	0.08	0.30	0.21	0.14	0.35
20-Hydroxy-leukotriene E4	-0.13	0.10	0.22	-0.07	0.05	0.13	-0.12	0.10	0.47
DTDP-4-acetamido-4,6-dideoxy-D-galactose	-0.04	0.03	0.13	-0.01	0.01	0.27	-0.04	0.03	0.32
Semilepidinoside B	0.13	0.08	0.08	-0.01	0.04	0.82	0.10	0.07	0.42
Tenuazonic acid	-0.54	0.21	0.01	-0.15	0.09	0.10	-0.42	0.20	0.14
Cochinchinenin	0.23	0.05	0.00	0.09	0.02	0.00	0.25	0.05	0.00

Table S11. The sources of rumen metabolites

Name	Origin
Deoxyuridine	Host; Microbiota; Food related; Drug related
2-Deoxyuridine	Host; Microbiota; Food related; Drug related
4-PyridoxicAcid	Host; Microbiota; Food related
Beta-Alanyl-L-histidine	Host; Microbiota; Food related; Drug related; Environment
Adenine	Host; Microbiota; Food related; Drug related
TaurocholicAcid	Host; Microbiota; Food related; Drug related
Betaine	Host; Microbiota; Food related; Drug related
Ascorbicacid	Host; Microbiota; Food related; Drug related
Adenosinemonophosphate	Microbiota; Food related; Drug related
Deoxyadenosine	Host; Microbiota; Food related; Drug related; Environment
Adenosine	Host; Microbiota; Food related; Drug related; Environment
L-Carnitine	Microbiota; Food related; Drug related; Environment
Creatine	Host; Microbiota; Food related; Drug related; Environment
(-)-Epinephrine	Host; Microbiota; Food related; Drug related
Epinephrine	Host; Microbiota; Food related; Drug related
Deoxyinosine	Host; Microbiota; Food related; Drug related
Dopamine	Host; Microbiota; Food related; Drug related
Glycerylphosphorylcholine	Host; Microbiota; Food related; Drug related
Cytidine	Host; Microbiota; Food related; Drug related
CitricAcid	Host; Microbiota; Food related; Drug related; Environment
Guanidoaceticacid	Host; Microbiota; Food related; Drug related
Glycerol	Host; Microbiota; Food related; Drug related; Environment
Guanine	Host; Microbiota; Food related; Drug related
Guanosine	Host; Microbiota; Food related; Drug related
GlycocholicAcid	Host; Microbiota; Food related; Drug related
GlycericAcid	Host; Microbiota; Food related
N-Choloylglycine	Host; Microbiota; Food related; Drug related
L-GlutamicAcid	Host; Microbiota; Food related; Drug related
Gluconolactone	Host; Microbiota; Food related; Drug related
MalicAcid	Host; Microbiota; Food related
Hypoxanthine	Host; Microbiota; Food related; Drug related
L-Tyrosine	Host; Microbiota; Food related; Drug related; Environment
L-Phenylalanine	Host; Microbiota; Food related; Drug related; Environment
Phenyl-Alanine	Host; Microbiota; Food related; Drug related; Environment
L-Proline	Host; Microbiota; Food related; Drug related; Environment
L-Threonine	Host; Microbiota; Food related; Drug related; Environment
L-Isoleucine	Host; Microbiota; Food related; Drug related; Environment
Inosine5-Phosphate	Host; Microbiota; Food related; Drug related
L-Lysine	Host; Microbiota; Food related; Drug related; Environment
L-Serine	Host; Microbiota; Food related; Drug related; Environment
2-Amino-3-MethylsuccinicAcid	Host; Microbiota; Food related; Drug related
L-AsparticAcid	Host; Microbiota; Food related; Drug related

Indole-3-Carboxaldehyde	Microbiota; Food related; Drug related
O-Acetylcarnitine	Microbiota; Food related; Drug related
Nepsilon-Acetyl-L-lysine	Microbiota; Food related
N-Epsilon-Acetyl-L-Lysine	Microbiota; Food related
N6-Acetyl-L-lysine	Microbiota; Food related
Oxoglutaricacid	Host; Microbiota; Food related; Drug related
Phenylaceticacid	Host; Microbiota; Food related; Drug related; Environment
PantothenicAcid	Host; Microbiota; Food related; Drug related
Myo-Inositol	Host; Microbiota; Food related; Drug related
N-Acetylgalactosamine	Food related; Drug related
L-Ornithine	Host; Microbiota; Food related; Drug related
Orotidylicacid	Host; Microbiota; Food related; Drug related
L-Palmitoylcarnitine	Host; Microbiota; Food related
OroticAcid	Host; Microbiota; Food related; Drug related
Phenol	Microbiota; Food related; Drug related; Environment
N-Acetylneuraminicacid	Host; Microbiota; Food related; Drug related
Propionicacid	Host; Microbiota; Food related; Drug related
Porphobilinogen	Host; Microbiota; Food related; Drug related
D-Sorbitol	Host; Microbiota; Food related; Drug related
SuccinicAcid	Host; Microbiota; Food related; Drug related
Sucrose	Host; Microbiota; Food related; Drug related
Serotonin	Host; Microbiota; Food related; Drug related
Thymine	Host; Microbiota; Food related; Drug related
PyroglutamicAcid	Host; Microbiota; Food related; Drug related
Sphinganine	Host; Microbiota; Food related
3-phospho-L-serine	Host; Microbiota; Food related; Drug related
Sphingosine1-phosphate	Host; Microbiota; Food related
N6-(L-1,3-Dicarboxypropyl)-L-lysine	Host; Microbiota; Food related; Drug related
Uridine5-monophosphate	Host; Microbiota; Food related; Drug related
Uridine-5-Monophosphate	Host; Microbiota; Food related; Drug related
UricAcid	Host; Microbiota; Food related; Drug related
Xanthine	Host; Microbiota; Food related; Drug related
Uridine	Host; Microbiota; Food related; Drug related
Xanthosine	Host; Microbiota; Food related
Uracil	Host; Microbiota; Food related; Drug related
Tyramine	Host; Microbiota; Food related; Drug related
2-Hydroxyestrone	Host; Food related
Estriol	Host; Food related
3-HydroxysebacicAcid	Food related
3-Hydroxy-3-methylglutarate	Food related; Drug related
17-Epiestriol	Food related; Drug related
Dihydro-3-coumaricacid	Microbiota; Food related
2-Methylbutyroylcarnitine	Food related
3-Hydroxydodecanoicacid	Microbiota; Food related

7-Ketodeoxycholicacid	Food related
3-Hydroxytetradecanedioicacid	Food related
2,3-Dihydroxybenzoicacid	Microbiota; Food related; Drug related
2-PyrocatechuicAcid	Microbiota; Food related; Drug related
3a,6b,7a,12a-Tetrahydroxy-5b-cholanoicacid	Host; Microbiota; Food related
2-IsopropylmalicAcid	Microbiota; Food related
2-Hydroxyadenine	Food related
2-Methoxyestradiol	Host; Food related; Drug related
2-Hydroxy-3-MethylbutyricAcid	Food related
3-Hydroxydodecanedioicacid	Food related
Alpha-MuricholicAcid	Food related
3,4-Dihydroxyhydrocinnamicacid	Microbiota; Food related
2-Hydroxydecanedioicacid	Food related
3a,7b,12a-Trihydroxy-5a-Cholanoicacid	Host; Food related
N-(2-Furoyl)glycine	Microbiota; Food related
2-Furoylglycine	Microbiota; Food related
Nalpha-Acetyl-L-Lysine	Food related
7a,12a-Dihydroxy-3-oxo-4-cholenoicacid	Food related
AdipicAcid	Microbiota; Food related; Drug related; Environment
Cis-4-Hydroxycyclohexylaceticacid	Microbiota; Food related
3-Methylcrotonylglycine	Food related
Allantoin	Microbiota; Food related; Drug related; Environment
3-Methylindole	Microbiota; Food related
5-Hydroxy-L-tryptophan	Host; Microbiota; Food related; Drug related
Allopurinolriboside	Food related
N-Acetyl-L-phenylalanine	Microbiota; Food related
6-Ketoestriol	Food related
Acetylglycine	Food related; Drug related
Caproicacid	Microbiota; Food related
8-Hydroxyadenine	Host; Food related
3-MethyladipicAcid	Food related
Goshuyicacid	Food related
Cinnamicacid	Microbiota; Food related
Trans-CinnamicAcid	Microbiota; Food related
D-Arabitol	Host; Microbiota; Food related
Gamma-Glutamylphenylalanine	Food related
Cis-4-Decenedioicacid	Food related
2-FuroicAcid	Microbiota; Food related
D-Ribulose5-Phosphate	Host; Microbiota; Food related; Drug related
Cholicacid	Host; Microbiota; Food related; Drug related
D-Ribulose	Host; Microbiota; Food related
DodecanedioicAcid	Food related
D-Gluconicacid	Host; Microbiota; Food related; Drug related
Cytosine	Microbiota; Food related

MesaconicAcid	Microbiota; Food related; Drug related
Chenodeoxyglycocholicacid	Host; Microbiota; Food related; Drug related
Chenodeoxycholicacidglycineconjugate	Host; Microbiota; Food related; Drug related
L-Glutamine	Host; Microbiota; Food related; Drug related; Environment
L-Arabinopyranose	Host; Microbiota; Food related; Drug related
D-Fructose	Microbiota; Food related; Drug related
Heptanoicacid	Microbiota; Food related; Drug related
IndolelacticAcid	Microbiota; Food related; Drug related
L-Homocitrulline	Food related
Indoxylsulfuricacid	Microbiota; Food related; Drug related; Environment
Kynurenine	Host; Microbiota; Food related; Drug related
L-Kynurenine	Host; Microbiota; Food related; Drug related
L-Leucine	Host; Microbiota; Food related; Drug related; Environment
MalonicAcid	Host; Microbiota; Food related; Drug related
4-HydroxyphenylpyruvicAcid	Host; Microbiota; Food related; Drug related
Isoursodeoxycholicacid	Microbiota; Food related
HippuricAcid	Microbiota; Food related; Drug related; Environment
KynurenicAcid	Microbiota; Food related; Drug related
L-Pipecolicacid	Host; Microbiota; Food related
PipecolicAcid	Host; Microbiota; Food related
L-Homoserine	Microbiota; Food related; Drug related
Levulinicacid	Microbiota; Food related; Drug related
Glycylproline	Food related
4-Hydroxy-L-Proline	Host; Microbiota; Food related; Drug related; Environment
Isovalerylglutamicacid	Food related
Indoleacrylicacid	Microbiota; Food related
Hydroxyphenylacetylglycine	Food related
Indole	Microbiota; Food related; Drug related
Lactulose	Food related; Drug related
3-(4-Hydroxyphenyl)lactate	Microbiota; Food related
Glycyl-leucine	Food related
Glycylleucine	Food related
5-Hydroxyindoleaceticacid	Host; Microbiota; Food related
5-Hydroxyindoleacetate	Host; Microbiota; Food related
Hydrocinnamicacid	Microbiota; Food related; Drug related
Pseudouridine	Microbiota; Food related
Phenyllacticacid	Microbiota; Food related; Drug related
AzelaicAcid	Microbiota; Food related; Drug related; Environment
Oxypurinol	Food related; Drug related
Decanedioicacid	Microbiota; Food related; Drug related; Environment
SebacicAcid	Microbiota; Food related; Drug related; Environment
Beta-N-Acetylglucosamine	Food related
Myristicacid	Host; Microbiota; Food related; Drug related
Normetanephrine	Host; Microbiota; Food related

Phenylacetylglycine	Host; Microbiota; Food related
4-HydroxymandelicAcid	Food related
Propionylcarnitine	Food related; Drug related
Neuraminicacid	Food related
Capryloylglycine	Food related
Salicyluricacid	Microbiota; Food related
Pelargonicacid	Microbiota; Food related
Rhamnose	Microbiota; Food related; Drug related
N-Acetyl-b-D-galactosamine	Food related
N-Acetyl-D-galactosamine	Host; Food related
N-formimidoyl-glutamicacid	Host; Microbiota; Food related
Formiminoglutamicacid	Host; Microbiota; Food related
PimelicAcid	Microbiota; Food related; Drug related
N-Acetyltyrosine	Food related; Drug related
Acetyl-L-tyrosine	Food related; Drug related
N-Acetyl-L-Tyrosine	Food related; Drug related
Tetradecanedioicacid	Food related
Trigonelline	Food related
L-Valine	Host; Microbiota; Food related; Drug related; Environment
Ribothymidine	Microbiota; Food related
Undecanedioicacid	Food related
SubericAcid	Food related
Valproicacidglucuronide	Food related
Citrulline	Host; Microbiota; Food related; Drug related
Tridecanoicacid	Microbiota; Food related; Drug related
Succinyladenosine	Food related
L-Tryptophan	Host; Microbiota; Food related; Drug related; Environment
TraumaticAcid	Food related
S-Adenosylhomocysteine_1	Host; Microbiota; Food related; Drug related
S-Adenosylhomocysteine_2	Host; Microbiota; Food related; Drug related
Threonolactone	Food related
ThreonicAcid	Microbiota; Food related; Drug related
Taurochenodesoxycholicacid	Host; Microbiota; Food related; Drug related
Suberylglycine	Food related
FerulicAcid	Microbiota; Food related; Drug related
Isoferulicacid	Food related; Drug related
Pyrocatechol	Microbiota; Food related; Drug related; Environment
Trans-AconiticAcid	Microbiota; Food related
Trehalose	Host; Microbiota; Food related; Drug related
4-(2-Aminophenyl)-2,4-dioxobutanoicacid	Host; Microbiota; Food related
3-Pyridinebutanoicacid	Food related
Cotinineglucuronide	Host; Microbiota; Food related
N,N-Dimethylaniline	Food related
3-Ketolactose	Food related

Cotinine	Food related; Environment
4-Aminobutyraldehyde	Host; Microbiota; Food related
P-AnisicAcid	Microbiota; Food related; Drug related
N-Acetyl-b-glucosaminylamine	Food related
4-Hydroxy-4-(3-pyridyl)-butanoicacid	Food related
2-AminobenzoicAcid	Host; Microbiota; Food related; Drug related
6-Phosphonoglucono-D-lactone	Host; Microbiota; Food related
N-Acetyl-D-mannosamine	Host; Microbiota; Food related
Nicotinicacidmononucleotide	Host; Microbiota; Food related; Drug related
N-Acetyl-L-GlutamicAcid	Host; Microbiota; Food related; Drug related
4-TrimethylammoniobutanoicAcid	Host; Microbiota; Food related
Trans-1,2-Dihydrobenzene-1,2-diol	Food related
Cytidine5-monophosphate-N-acetylneuraminicacid	Host; Microbiota; Food related; Drug related
N1-Acetylspermine	Microbiota; Food related
Indole-3-acetaldehyde	Host; Microbiota; Food related
Indoleacetaldehyde	Host; Microbiota; Food related
N2-Succinyl-L-ornithine	Microbiota; Food related; Drug related
N-Formylkynurenine	Host; Microbiota; Food related
Deoxycytidylicacid	Host; Microbiota; Food related; Drug related
ProstaglandinE2	Host; Microbiota; Food related; Drug related
DTMP	Host; Microbiota; Food related; Drug related
4-Nitrophenol	Microbiota; Food related; Drug related; Environment
Spermidine	Host; Microbiota; Food related; Drug related
Allysine	Host; Microbiota; Food related; Drug related
L-Sorbinose	Microbiota; Food related
N1-Acetylspermidine	Microbiota; Food related
2-Aminomuconicacidsemialdehyde	Host; Microbiota; Food related
Maltotetraose	Microbiota; Food related; Drug related
CGMP	Host; Microbiota; Food related; Drug related
3,5-CyclicGMP	Host; Microbiota; Food related; Drug related
N6,N6,N6-Trimethyl-L-lysine	Host; Microbiota; Food related; Drug related
Beta-Hydroxypyruvicacid	Host; Microbiota; Food related; Drug related
LL-2,6-DiaminopimelicAcid	Microbiota; Food related; Drug related
5-GuanylicAcid	Host; Microbiota; Food related; Drug related
ProstaglandinD2	Host; Microbiota; Food related; Drug related
Niacinamide	Host; Microbiota; Food related; Drug related; Environment
7,8-Dihydropteroicacid	Host; Microbiota; Food related
Putrescine	Host; Microbiota; Food related; Drug related
3-Hydroxy-N6,N6,N6-trimethyl-L-lysine	Host; Microbiota; Food related
Agmatine	Host; Microbiota; Food related; Drug related
ProstaglandinE1	Food related; Drug related
ThromboxaneA2	Host; Food related
Tiglicacid	Food related
3-HydroxyanthranilicAcid	Host; Microbiota; Food related; Drug related

Ribose1-phosphate	Host; Microbiota; Food related; Drug related
Vanylglycol	Host; Microbiota; Food related
Benzene	Microbiota; Food related; Environment
20-Hydroxy-leukotrieneB4	Food related
5-Formiminotetrahydrofolicacid	Host; Microbiota; Food related
Phenylglyoxylicacid	Microbiota; Food related; Drug related
M-Coumaricacid	Microbiota; Food related
5-Hydroxytryptophol	Food related
ProtocatechuicAcid	Microbiota; Food related; Drug related
M-Cresol	Microbiota; Food related; Drug related; Environment
Acetaminophen	Food related; Drug related; Environment
2-HydroxypentanoicAcid	Food related
3,4-DihydroxymandelicAcid	Host; Microbiota; Food related
BenzoicAcid	Microbiota; Food related; Drug related; Environment
Valproicacid	Food related; Drug related; Environment
Thymol	Food related; Drug related
M-Aminobenzoicacid	Food related; Drug related
Aspartame	Food related; Drug related
5-Methoxytryptophol	Food related
Aminocaproicacid	Microbiota; Food related; Drug related
Atenolol	Food related; Drug related; Environment
Salbutamol	Food related; Drug related; Environment
3-Hydroxyoctanoicacid	Food related
CaffeicAcid	Microbiota; Food related; Drug related; Environment
5-Deoxyadenosine	Microbiota; Food related
4-Hydroxycyclohexylcarboxylicacid	Microbiota; Food related
9-Tetradecenoicacid	Food related
Myristoleicacid	Food related
MethionineSulfoxide	Microbiota; Food related
Butyryl-L-carnitine	Food related
4-Carboxyphenylglycine	Unknown
Ureidoisobutyricacid	Microbiota; Food related
N(6)-Methyllysine	Food related; Drug related
1-Methyl-2-Pyrrolidinone	Food related
5-Phenylvalericacid	Microbiota; Food related; Drug related
O-Cresol	Microbiota; Food related; Environment
N-Acetylputrescine	Host; Microbiota; Food related
6-Lactoyltetrahydropterin	Host; Microbiota; Food related
N-Oleoylethanolamine	Food related; Drug related
PalmitoylEthanolamide	Food related; Drug related
PhthalicAcid	Microbiota; Food related; Drug related
CurcuminIII	Food related
ProstaglandinF3a	Food related
1,3,7-TrimethyluricAcid	Host; Microbiota; Food related

3Hydroxycoumarin	Food related
(S)-beta-Aminoisobutyricacid	Host; Microbiota; Food related
S-(2-carboxypropyl)-Cysteamine	Food related
N1,N12-Diacetylspermine	Food related
L-Threo-3-Phenylserine	Food related
N8-Acetylspermidine	Microbiota; Food related
N-Carboxyethyl-gamma-aminobutyricacid	Food related
3-Hydroxycapricacid	Food related
Cuminaldehyde	Microbiota; Food related
N-Acetyl-6-O-L-fucosyl-D-glucosamine	Food related
Adrenicacid	Food related
11Z-Eicosenoicacid	Microbiota; Food related; Drug related
PicolinicAcid	Food related; Drug related
2-HydroxymyristicAcid	Food related
14,15-DiHETrE	Host; Microbiota; Food related
Imidazolepropionicacid	Microbiota; Food related
2,3-Dinor-11b-PGF2a	Food related
2,3-Dinor-6-keto-prostaglandinF1a	Food related
N-Acetylcadaverine	Microbiota; Food related
(R)-beta-Aminoisobutyricacid	Host; Microbiota; Food related
3-Indolepropionicacid	Microbiota; Food related; Drug related
LeukotrieneB4ethanolamide	Food related
Cadaverine	Host; Microbiota; Food related; Drug related
1,11-Undecanedicarboxylicacid	Food related
Imidazoleaceticacidriboside	Food related
BiochaninA	Food related; Drug related; Environment
5-Methoxytryptophan	Food related
2-Methylbenzoicacid	Microbiota; Food related
Capsidiol	Food related
Hawkinsin	Food related
Terephthalicacid	Microbiota; Food related
Isorhamnetin	Food related; Drug related
6-HydroxynicotinicAcid	Microbiota; Food related
PGF1alpha	Food related
ProstaglandinF1a	Food related
Nandrolone	Food related; Drug related
13,14-Dihydro-15-keto-PGE2	Host; Food related
Levonordefrin	Food related
Deacetyldiltiazem	Food related
6-Keto-prostaglandinF1a	Food related
5-Methylcytosine	Microbiota; Food related
N-Acetylserine	Food related; Drug related
PGD3	Food related
ProstaglandinD3	Food related

Arabinosylhypoxanthine	Food related
Lactaldehyde	Microbiota; Food related; Drug related
Shikimicacid	Microbiota; Food related
Raffinose	Host; Microbiota; Food related; Drug related
Genistein	Microbiota; Food related; Drug related; Environment
Sedoheptulose	Host; Food related
Palmitelaidicacid	Microbiota; Food related; Drug related
Vaccenicacid	Food related; Drug related
ProstaglandinG2	Host; Food related; Drug related
ThromboxaneB2	Food related
NicotinicAcid	Host; Food related
Daidzein	Host; Microbiota; Food related; Drug related
Oxidizedglutathione	Host; Microbiota; Food related; Drug related
Glutamate	Host; Microbiota; Food related; Drug related
5-AminovalericAcid	Microbiota; Food related
N2-Acetylornithine	Host; Microbiota; Food related
N2-Acetyl-L-ornithine	Host; Microbiota; Food related
Tryptophol	Microbiota; Food related
4-Pyridoxolactone	Microbiota; Food related
4-GuanidinobutanoicAcid	Host; Microbiota; Food related
L-Gulonolactone	Host; Microbiota; Food related
3a,7a,12a-Trihydroxy-5b-cholestan-26-al	Host; Food related
Levan	Microbiota; Food related
Levann	Microbiota; Food related
Adenosine3-monophosphate	Microbiota; Food related
3-AMP	Microbiota; Food related
Butanal	Microbiota; Food related
Stachyose	Host; Microbiota; Food related
GibberellinA3	Food related; Drug related
N-Methyltyramine	Microbiota; Food related
(S)-(-)-Perillylalcohol	Food related
Beta-Leucine	Food related
4-Coumarylalcohol	Host; Microbiota; Food related
N-Methylputrescine	Host; Microbiota; Food related
4-Acetamidobutanoate	Host; Microbiota; Food related
Glutamylalanine	Food related
4-Hydroxyphenylacetaldehyde	Host; Microbiota; Food related
3,4-Dihydroxyphenylacetaldehyde	Host; Microbiota; Food related
3-Amino-3-(4-hydroxyphenyl)propanoicAcid	Food related
Beta-Tyrosine	Food related
3-Aminoisobutanoicacid	Food related
7-Methylinosine	Host; Microbiota; Food related
L-Allothreonine	Host; Microbiota; Food related
Gentisatealdehyde	Host; Microbiota; Food related

Leucodopachrome	Food related
4-Vinylphenol	Microbiota; Food related
5-Hydroxykynurenamine	Host; Microbiota; Food related
6-Hydroxymelatonin	Host; Microbiota; Food related
4-(2-Amino-3-hydroxyphenyl)-2,4-dioxobutanoicacid	Host; Microbiota; Food related
Indoxyl	Food related
5-Methoxyindoleacetate	Host; Food related
3-Methyldioxyindole	Food related
N1-Methyl-2-pyridone-5-carboxamide	Host; Food related
Glutamyllysine	Microbiota; Food related
Pantothenol	Food related; Drug related
6-KetoprostaglandinE1	Food related
11-Dehydro-thromboxaneB2	Food related
Tyrosol	Microbiota; Food related; Drug related
Isopyridoxal	Food related
1-Butanol	Microbiota; Food related; Drug related
Estroneglucuronide	Host; Food related
(S)-Carvone	Microbiota; Food related
Demethylphylloquinone	Microbiota; Food related
MG(0:0/20:4(5Z,8Z,11Z,14Z)/0:0)	Food related
9-Hydroxylinoleicacid	Food related; Drug related
9(S)-HODE	Food related; Drug related
9,10-DiHOME	Food related
9,10-DHOME	Food related
12,13-DHOME	Food related
9,10,13-TriHOME	Food related
3-Methyluridine	Food related
FAPy-adenine	Food related
Biotinsulfone	Food related
P-Octopamine	Food related; Drug related
Prolinebetaine	Microbiota; Food related; Drug related
Atorvastatin	Food related; Drug related; Environment
Olanzapine	Food related; Drug related; Environment
Venlafaxine	Food related; Drug related; Environment
Nitrolinoleicacid	Food related
Enterodiol	Food related
13,14-DihydroPGF-1a	Food related
5(S),14(R)-LipoxinB4	Food related
ProstaglandinD1	Food related
Glycitein	Microbiota; Food related
Gingerol	Food related
Indole-3-Carbinol	Food related; Drug related
Quercetin	Host; Microbiota; Food related; Drug related; Environment
P-Cymene	Microbiota; Food related

Eugenol	Food related; Drug related; Environment
7-Methylguanosine	Food related
3-O-Methylguanosine	Food related
20-carboxy-LTB4	Food related
20-Carboxy-leukotrieneB4	Food related
4-Hydroxyphenylacetylglutamicacid	Food related
(+/-)-Enterolactone	Food related
Propyleneglycol	Microbiota; Food related; Drug related
1-a,24R,25-TrihydroxyvitaminD2	Food related
S-aminomethyldihydrolipoamide	Host; Microbiota; Food related; Drug related
4-Hydroxyretinoicacid	Host; Microbiota; Food related
16-Hydroxyhexadecanoicacid	Food related
Phenylacetylglutamine	Microbiota; Food related
Pseudoecgonine	Food related
Stearidonylcarnitine	Food related
Calcitroicacid	Food related
Sphingosylphosphorylcholine	Food related
AsparticAcid	Host; Microbiota; Food related; Drug related
N-Acetyl-L-glutamate5-semialdehyde	Microbiota; Food related
StearidonicAcid	Host; Food related
L-4-Hydroxyglutamatesemialdehyde	Host; Microbiota; Food related
Lactosamine	Host; Microbiota; Food related
3a,21-Dihydroxy-5b-pregnane-11,20-dione	Host; Food related
5-(2-Carboxyethyl)-4,6-Dihydroxypicolinate	Food related
Beta-D-Fructose2-phosphate	Microbiota; Food related
D-Pantothenoyl-L-cysteine	Host; Microbiota; Food related
(S)-2-Acetolactate	Microbiota; Food related
S-(2-Methylpropionyl)-dihydrolipoamide-E	Microbiota; Food related
D-Tagatose6-phosphate	Host; Microbiota; Food related
1-Pyrroline-2-carboxylicacid	Host; Microbiota; Food related; Drug related
(S)-2-Aceto-2-hydroxybutanoicacid	Microbiota; Food related
DG(14:0/18:3(9Z,12Z,15Z)/0:0)	Food related
DG(14:0/20:0/0:0)	Food related
DG(14:1(9Z)/18:4(6Z,9Z,12Z,15Z)/0:0)	Food related
DG(15:0/18:2(9Z,12Z)/0:0)	Food related
DG(15:0/18:3(9Z,12Z,15Z)/0:0)	Food related
DG(15:0/18:4(6Z,9Z,12Z,15Z)/0:0)	Food related
DG(20:1(11Z)/16:1(9Z)/0:0)	Food related
DG(20:2(11Z,14Z)/18:4(6Z,9Z,12Z,15Z)/0:0)	Food related
LysoPA(0:0/18:1(9Z))	Host; Microbiota; Food related
LysoPA(0:0/18:2(9Z,12Z))	Host; Microbiota; Food related
PE(14:0/14:0)	Host; Microbiota; Food related; Drug related
PE(16:1(9Z)/P-18:1(9Z))	Host; Microbiota; Food related; Drug related
PE(18:4(6Z,9Z,12Z,15Z)/20:5(5Z,8Z,11Z,14Z,17Z))	Host; Microbiota; Food related; Drug related

PE(22:6(4Z,7Z,10Z,13Z,16Z,19Z)/16:1(9Z))	Host; Microbiota; Food related; Drug related
11-Epi-PGF2a	Host; Food related
L-Rhamnulose	Microbiota; Food related
Beta-D-Glucopyranuronicacid	Food related
Acetaminophenglucuronide	Food related
Phenethylamineglucuronide	Host; Microbiota; Food related
Ethylglucuronide	Food related
Tyramineglucuronide	Host; Microbiota; Food related
Retinylbeta-glucuronide	Food related
6-Hydroxy-5-methoxyindoleglucuronide	Host; Microbiota; Food related
5-Hydroxy-6-methoxyindoleglucuronide	Host; Microbiota; Food related
LysoPC(14:0/0:0)	Host; Microbiota; Food related
PC(15:0/0:0)	Host; Microbiota; Food related
PC(16:0/0:0)	Host; Microbiota; Food related
LysoPC(16:1(9Z)/0:0)	Host; Microbiota; Food related
LysoPC(18:0/0:0)	Microbiota; Food related
PC(18:0/0:0)	Host; Microbiota; Food related
LysoPC(18:1(11Z)/0:0)	Host; Microbiota; Food related
1-Linoleoyl-sn-Glycero-3-Phosphocholine	Food related
LysoPC(18:3(6Z,9Z,12Z)/0:0)	Host; Microbiota; Food related
LysoPC(18:3(9Z,12Z,15Z)/0:0)	Host; Microbiota; Food related
LysoPC(20:0/0:0)	Host; Microbiota; Food related
LysoPC(20:3(5Z,8Z,11Z)/0:0)	Host; Microbiota; Food related
LysoPC(20:3(8Z,11Z,14Z)/0:0)	Host; Microbiota; Food related
LysoPC(20:4(8Z,11Z,14Z,17Z)/0:0)	Host; Microbiota; Food related
LysoPC(20:5(5Z,8Z,11Z,14Z,17Z)/0:0)	Host; Microbiota; Food related
LysoPC(P-18:1(9Z)/0:0)	Host; Microbiota; Food related
CerP(d18:1/12:0)	Food related
3-Oxohexanoicacid	Food related
(R)-3-Hydroxyoctanoicacid	Food related
3-Oxododecanoicacid	Food related
Hypusine	Food related
Glutamylhydroxyproline	Food related
Gamma-Glutamylisoleucine	Food related
Gamma-Glu-leu	Food related
Gamma-Glutamylvaline	Food related
Isoleucylproline	Food related
Leucylproline	Food related
Phenylalanylhydroxyproline	Food related
L-phenylalanyl-L-proline	Food related
Phenylalanylproline	Food related
Prolylphenylalanine	Food related
Prolylproline	Microbiota; Food related
LysoPE(0:0/16:0)	Microbiota; Food related

LysoPE(0:0/18:3(6Z,9Z,12Z))	Food related
LysoPE(0:0/18:4(6Z,9Z,12Z,15Z))	Food related
LysoPE(14:1(9Z)/0:0)	Food related
LysoPE(15:0/0:0)	Food related
LysoPE(16:0/0:0)	Microbiota; Food related
LysoPE(16:1(9Z)/0:0)	Food related
LysoPE(18:2(9Z,12Z)/0:0)	Food related
LysoPE(18:3(6Z,9Z,12Z)/0:0)	Food related
LysoPE(18:4(6Z,9Z,12Z,15Z)/0:0)	Food related
LysoPE(20:0/0:0)	Food related
MG(0:0/14:1(9Z)/0:0)	Food related
7-Methyladenine	Food related
Adenosine2-phosphate	Microbiota; Food related
Dulcitol	Host; Food related
TaurohyocholicAcid	Food related
3beta-hydroxy-4beta-methyl-5alpha-cholest-7-ene-4alpha-carboxylate	Food related
6-(alpha-D-Glucosaminyl)-1D-myo-inositol	Microbiota; Food related; Drug related
D-Xylono-1,5-lactone	Host; Microbiota; Food related
P-Cresolglucuronide	Food related
2,3cyclicCMP	Host; Microbiota; Food related; Drug related
Vanilpyruvicacid	Food related
Nonate	Food related
4-Hydroxybenzaldehyde	Microbiota; Food related; Drug related
1-Kestose	Food related
Melezitose	Food related
Gamma-Glutamylglutamicacid	Food related
N2-gamma-Glutamylglutamine	Food related
Gamma-Glutamyltyrosine	Food related
2-Piperidinone	Food related
N-Acetylleucine	Food related; Drug related
Acetyl-DL-Leucine	Food related; Drug related
N-Acetyl-DL-Valine	Food related
GangliosideGM2(d18:1/23:0)	Food related
5,6-DihydroxyprostaglandinF1a	Food related
(3S)-3,6-Diaminohexanoate	Microbiota; Food related
2,3,4,5-Tetrahydro-2-pyridinecarboxylicacid	Host; Microbiota; Food related
1-Amino-propan-2-ol	Microbiota; Food related; Drug related
2-Isopropyl-3-oxosuccinate	Microbiota; Food related
3-Isopropylmalicacid	Microbiota; Food related
4-(Glutamylamino)butanoate	Microbiota; Food related
5-Aminopentanamide	Microbiota; Food related
Trans-Zeatin	Food related; Drug related
Dopaminequinone	Food related

DTDP-4-acetamido-4,6-dideoxy-D-galactose	Microbiota; Food related
Glutaratesemialdehyde	Microbiota; Food related
Isopentenyladenine-9-N-glucoside	Food related
Isopropylmaleicacid	Microbiota; Food related
L-2,3-Dihydrodipicolinate	Microbiota; Food related
N-Succinyl-2-amino-6-ketopimelate	Microbiota; Food related
N-Succinyl-L,L-2,6-diaminopimelate	Microbiota; Food related
Tetrahydrodipicolinate	Microbiota
(S)-2,3,4,5-tetrahydrodipicolinate	Microbiota
Vanillylamine	Food related
3-O-Sulfogalactosylceramide(d18:1/14:0)	Host; Microbiota; Food related
Palmitoleicacid	Food related; Drug related
3beta,7alpha-Dihydroxy-5-cholestenoate	Host; Food related
7alpha-Hydroxy-3-oxo-4-cholestenoate	Host; Food related
(-)-Salsoline	Food related
Norlaudanosoline	Food related
1-(1,2,3,4,5-Pentahydroxypent-1-yl)-1,2,3,4-tetrahydro-beta-carboline-3-carboxylate	Food related
12S-HHT	Food related
12-oxo-20-dihydroxy-leukotrieneB4	Food related
20-Hydroxy-leukotrieneE4	Food related
3-Dehydroquinicacid	Microbiota; Food related; Drug related
5-Aminopentanal	Host; Microbiota; Food related
Acetamidopropanal	Food related
Adipatesemialdehyde	Microbiota; Food related
Coniferylalcohol	Host; Microbiota; Food related
Gamma-Tocotrienol	Microbiota; Food related
1-Hexanol	Microbiota; Food related
Kinetensin1-3	Food related
Leukoaminochrome	Food related
NeuromedinB(1-3)	Food related
ProstaglandinPGE21-glycerylester	Food related
Sinapylalcohol	Host; Microbiota; Food related
Valproylglycine	Food related
Hydroxypropionylcarnitine	Food related
5-Hydroxytryptopholglucuronide	Food related
3-Guanidinopropanoate	Food related
3-GuanidinopropionicAcid	Food related
Nomega-Acetylhistamine	Food related; Drug related
N-Acetylhistamine	Food related; Drug related
N-Undecanoylglycine	Food related
PGP(18:1(9Z)/18:3(9Z,12Z,15Z))	Food related
1,4-Dithiothreitol	Microbiota; Food related; Drug related
12(13)Ep-9-KODE	Food related

LinoleoylEthanolamide	Food related
Cervonoylethanolamide	Food related
8-[(Aminomethyl)sulfanyl-6-sulfanyloctanoicacid	Food related
6-Methylnicotinamide	Food related
N-Acetyltryptophan	Food related
Norvaline	Unknown
2-Furanmethanol	Food related
2-Methoxy-4-vinylphenol	Food related; Drug related
4-Vinylguaiacol	Food related; Drug related
2-Hydroxypyridine	Microbiota
2,3-dihydrobenzofuran	Food related
SulfamethoxazoleN1-glucuronide	Microbiota
S-4-Hydroxymephenytoin	Unknown
3,4-Dihydrodiol	Unknown
4-ene-Valproicacid	Food related
5-Hydroxyvalproicacid	Food related
N-Deschlorobenzoylindomethacin	Unknown
N-Deisopropyl-fluvastatin	Unknown
Carbidopa	Drug related
Vidarabine	Drug related
Midodrine	Drug related
Lymecycline	Drug related; Environment
Carmustine	Food related; Drug related; Environment
Sulfisoxazole	Drug related
TranexamicAcid	Drug related
Megestrol	Food related; Drug related
Clozapine	Drug related; Environment
Mexiletine	Drug related
Oxyphencyclimine	Drug related
Streptozocin	Drug related; Environment
STREPTOZOTOCIN	Drug related; Environment
Enoxacin	Drug related
Zanamivir	Drug related
Furazolidone	Food related; Drug related
Stavudine	Drug related; Environment
Metaxalone	Drug related
Mycophenolicacid	Drug related
Procaine	Food related; Drug related; Environment
Methoxamine	Drug related
Nateglinide	Drug related
Irinotecan	Host; Drug related
Mometasone	Food related; Drug related
Racemetirosine	Drug related
Clavulanate	Microbiota; Food related; Drug related

Perindopril	Drug related
Primidone	Drug related
Phenmetrazine	Drug related; Environment
Phensuximide	Drug related
Methylphenobarbital	Drug related
Didanosine	Drug related; Environment
Buprenorphine	Drug related; Environment
Felbamate	Host; Drug related; Environment
Lomefloxacin	Drug related
Cytarabine	Food related; Drug related; Environment
Cyclacillin	Drug related
Ketoprofen	Drug related
Cerulenin	Drug related; Environment
Thalidomide	Drug related; Environment
Lubiprostone	Food related; Drug related
Acetophenazine	Drug related
Vigabatrin	Drug related; Environment
Pilocarpine	Drug related; Environment
Phenacemide	Food related; Drug related; Environment
Cloxacillin	Drug related
Clindamycin	Drug related; Environment
Metipranolol	Drug related
Gemfibrozil	Drug related; Environment
Pirbuterol	Drug related
Aprobarbital	Drug related
Colchicine	Food related; Drug related
NiflumicAcid	Drug related
METHACHOLINE	Drug related
UreidoglutaricAcid	Drug related
Alanyl-Asparticacid	Food related
Alanylasparticacid	Food related
Alanylisoleucine	Food related
ALA-ILE	Food related
Alanylproline	Food related
L-Alanyl-l-Valine	Food related
Arginylasparagine	Food related
Arginylglutamine	Food related
Arginyllysine	Food related
Arginylproline	Food related
Asparaginylasparticacid	Food related
Asparaginylhydroxyproline	Food related
Asparaginyl-Lysine	Food related
Asparaginyl-Proline	Food related
Asparaginyl-Valine	Food related

Cysteinylhydroxyproline	Food related
Glutaminylhistidine	Food related
Glutaminylthreonine	Food related
Glutamylarginine	Food related
Glutamylglutamicacid	Microbiota; Food related
Glutamylisoleucine	Food related
Glutamylleucine	Food related
Glutamylproline	Food related
Glutamyltryptophan	Food related; Drug related
Glutamylvaline	Food related
Gly-Ile	Food related
Glycylvaline	Food related
Glycyl-Gamma-glutamate	Food related
Hydroxyprolyl-Methionine	Food related
Hydroxyprolyl-Proline	Food related
Histidylarginine	Food related
Histidylcysteine	Food related
Histidylhistidine	Microbiota; Food related
Histidyltryptophan	Food related
Histidylvaline	Food related
Isoleucyl-Arginine	Food related
Isoleucyl-Asparagine	Food related
Isoleucyl-Glutamate	Food related
Isoleucyl-Valine	Food related
Leucyl-Glutamate	Food related
Leucylhydroxyproline	Food related
Lysylhydroxyproline	Food related
Lysyllysine	Microbiota; Food related
Lysyl-Lysine	Microbiota; Food related
Lysyltryptophan	Food related
Methionylhydroxyproline	Food related
Methionyl-Isoleucine	Food related
Methionyl-Proline	Food related
Phenylalanylthreonine	Food related
Prolyl-Arginine	Food related
Prolyl-Lysine	Food related
Serylvaline	Food related
Threonylglutamicacid	Food related
Threonylhydroxyproline	Food related
Threonylphenylalanine	Food related
Threonylproline	Food related
Tryptophyl-Lysine	Food related
Tryptophyl-Proline	Food related
Tryptophyl-Serine	Food related

Tryptophyl-Gamma-glutamate	Food related
Tyrosylhydroxyproline	Food related
Tyrosyl-Proline	Food related
Tyrosyl-Serine	Food related
Tyrosyl-Threonine	Food related
Tyrosyl-Tryptophan	Food related
Valylglutamine	Food related
Valylglutamicacid	Food related
Valylhydroxyproline	Food related
Valylisoleucine	Food related
Valylleucine	Food related
N-Valylphenylalanine	Food related
Valylproline	Food related
N2-gamma-L-Glutamyl-L-arginine	Food related
Gamma-Glutamylglutamine	Food related
Glutamylphenylalanine	Unknown
Gamma-Glutamylproline	Food related
3-Hydroxyadipicacid3,6-lactone	Food related
UrolithinA-3-O-glucuronide	Food related
4-Hydroxystachydrine	Food related
3-Feruloyl-1,5-quinolactone	Food related
OphiopogoninC	Food related
Gravacridonetriolglucoside	Food related
4-Hydroxyprolinegalactoside	Food related
Solacauline	Food related
(E,E)-Trichostachine	Food related
Sinapine	Microbiota; Food related
L-3-Aminodihydro-2(3H)-furanone	Food related; Drug related
Cucurbicacid	Food related
N-Jasmonoylisoleucine	Food related
OchratoxinA	Microbiota; Food related
(S)-a-Amino-2,5-dihydro-5-oxo-4-isoxazolepropanoicacidN2-glucoside	Food related
Betanin	Food related
Betonicine	Food related
(S)-alpha-Amino-4-carboxy-3-furanpropanoicacid	Food related
L-trans-5-Hydroxy-2-piperidinecarboxylicacid	Food related
Nopalinicacid	Microbiota; Food related
L-Pyridosine	Food related
(2R,3R,4R)-2-Amino-4-hydroxy-3-methylpentanoicacid	Food related
Isoeugenitol	Food related
Licoricone	Microbiota; Food related
Liquiritigenin	Microbiota; Food related; Drug related
4-Hydroxy-3,5,6,7,8-pentamethoxyflavone	Food related

Cyclohexane	Microbiota; Food related
Lubiminol	Food related
4-MethylbenzoicAcid	Microbiota; Food related
P-Tolualdehyde	Microbiota; Food related
Eremopetasinorol	Food related
6-pentadecylSalicylicAcid	Food related
Cinnamylalcohol	Food related; Drug related
Trans-Cinnamylalcohol	Food related; Drug related
Saccharin	Food related; Drug related
Herniarin	Food related
7,8-Dihydrovomifoliol9-[rhamnosyl-(1-6)-glucoside	Food related
KanzonolV	Food related
(Z)-15-Oxo-11-eicosenoicacid	Food related
Momordol	Food related
Harmalol	Food related
VulgaxanthinII	Food related
Umbelliferone	Microbiota; Food related
2-C-Methyl-1,4-erythrono-D-lactone	Food related
Xylobiose	Microbiota; Food related
Serotinose	Food related
Inulobiose	Microbiota; Food related; Drug related
ArtoninT	Food related
TetraphyllinB	Food related
Arabinofuranobiose	Food related
Methylcellulose	Food related
D-manno-2-Heptulose	Food related
Panose	Food related
6-Amino-9H-purine-9-propanoicacid	Food related
Ascorbalamicacid	Food related
D-glycero-L-galacto-Octulose	Food related
Ethylbeta-D-glucopyranoside	Food related
CoriandroneD	Food related
Elenolide	Food related
Jasmolone	Food related
Carissanol	Food related
Glucosamine	Host; Food related
Homoferreirin	Food related
D-ononitol	Food related
4-Hydroxy-5,7-dimethoxyflavan	Food related
Venoterpine	Food related
(Z)-3-Oxo-2-(2-pentenyl)-1-cyclopenteneaceticacid	Food related
HovenineA	Food related
3-(3,4,5-Trimethoxyphenyl)propanoicacid	Food related
Nigellicine	Food related

Dihydromaleimidebeta-D-glucoside	Food related
Harmine	Microbiota; Food related; Drug related
(R)-Pelletierine	Food related
(+/-)-Pelletierine	Food related
Methylisopelletierine	Food related
6-Acetyl-1,2,3,4-tetrahydropyridine	Food related
Isoguvacine	Food related
(3R,5Z)-5-Octene-1,3-diol	Food related
3-Hydroxy-5Z-octenylacetate	Food related
(+/-)-Tryptophan	Microbiota; Food related
L-2-Amino-3-(4-aminophenyl)propanoicacid	Food related
L-4-Chlorotryptophan	Food related
2-Amino-3,4-dihydroxypentanedioicacid	Food related
Lycoperdicacid	Food related
28-Homobrassinolide	Food related
1,4-Ipomeadiol	Food related
4-Ipomeanol	Food related
Sativan	Microbiota; Food related
Piceid	Food related; Drug related
(Z)-Resveratrol4-glucoside	Food related
Silymonin	Food related
Dihydrosterigmatocystin	Microbiota; Food related
(-)-Wikstromol	Food related
MammeaB/BCcycloE	Food related
Mukurozidiol	Food related
Chrysoeriol	Food related; Drug related
Chrysophanol	Microbiota; Food related
P-CoumaricAcid	Food related
4-Hydroxycinnamicacid	Food related
5-Deoxydiplosporin	Food related
Sarmentosin	Food related
Dhurrin	Host; Food related
Taxiphyllin	Food related
3,4-Di-O-caffeoylquinicacid	Food related
Porson	Food related
AmericaninA	Food related
Anethole	Food related
4,5,7-Trimethoxyflavone	Food related
Dihydrokaempferol	Microbiota; Food related
Austrobailignan7	Food related
Rutarin	Food related
2,3-Secoporrigenin	Food related
1-Hydroxyepiacorone	Food related
9,10,13-Trihydroxystearicacid	Food related

2-Hexen-1-ol	Food related
CorchoionosideB	Food related
5-Hexyltetrahydro-2-oxo-3-furancarboxylicacid	Food related
Alpha-Carboxy-delta-decalactone	Food related
2-Carboxy-5,7-dimethyl-4-octanolide	Food related
2-Carboxy-4-dodecanolide	Food related
5-Pentyltetrahydro-2-oxo-3-furancarboxylicacid	Food related
Alpha-Carboxy-delta-nonalactone	Food related
5-Heptyltetrahydro-2-oxo-3-furancarboxylicacid	Food related
(2E,4Z,7Z,8E)-Colnelenicacid	Food related
Avocadene1-acetate	Food related
9E-Heptadecenoicacid	Food related
Avocadyne4-acetate	Food related
(Z)-10-Hydroxy-8-decene-4,6-diynoicacid	Food related
(S)-14-Methylhexadecanoicacid	Microbiota; Food related
(Z)-7-Hexadecen-1,16-olide	Food related
(9Z,11E,13E,15Z)-4-Oxo-9,11,13,15-octadecatetraenoicacid	Food related
Phaeophorbideb	Food related
DiallylTrisulfide	Food related
2-Methyl-4-pentenoicacid	Food related
6-Hydroxy-1H-indole-3-acetamide	Food related
TricarballylicAcid	Microbiota; Food related; Drug related
Isophorone	Food related
Zymonicacid	Food related
2-EthylhexanoicAcid	Food related
1-Hydroxy-3-octanone	Food related
(R)-1-Octen-3-ol	Food related
Osmundalin	Food related
5-Acetyl-3,4-dihydro-2H-pyrrole	Food related
Benzylacetate	Food related
2-Pentyl-3-phenyl-2-propenal	Food related; Drug related
1-Butylamine	Food related; Drug related
Cyclohexanecarboxylicacid	Microbiota; Food related
L-cis-Cyclo(aspartylphenylalanyl)	Food related
(+/-)-DulciolE	Food related
Mimosine	Food related; Drug related
2,3-Dimethyl-2-cyclohexen-1-one	Food related
Cis-Piceid	Food related
[8-Shogaol	Food related
Trans-2-Hexenal	Food related; Environment
DiacetoneAlcohol	Food related
(+/-)-3-Hydroxynonanoicacid	Food related
(E)-8-Hydroxy-2-octene-4,6-diynoicacid	Food related
D-Malicacid	Microbiota; Food related; Drug related

1-Methyl-1,3-cyclohexadiene	Food related
Morpholine	Food related; Drug related
1-Phenyl-2-pentanol	Food related
Ferreirin	Microbiota; Food related
3-Methyl-1-butylamine	Food related
Cis-3-Hexenylb-primeveroside	Food related
Xi-3-Hydroxy-5-phenylpentanoicacidO-beta-D-Glucopyranoside	Food related
(S,E)-Zearalenone	Food related
Methyl7-epi-12-hydroxyjasmonateglucoside	Food related
Heptenophos	Food related
Neotussilagine	Food related
Gamma-Glutamyl-S-methylcysteinesulfoxide	Food related
Prenylglucoside	Food related
6-Hydroxypentadecanedioicacid	Food related
Phaseolicacid	Food related
PorricacidA	Food related
Panaxytriol	Food related
Dimethyl(1R*,2S*,3S*)-2-carboxy-3-(3,4-dihydroxyphenyl)-2,3-dihydro-5,6-dihydroxy-1H-indene-1-acetate	Food related
(3b,6b,8a,12a)-8,12-Epoxy-7(11)-eremophilene-6,8,12-trimethoxy-3-ol	Food related
(3b,6b,8b,12a)-8,12-Epoxy-7(11)-eremophilene-6-angeloyloxy-8,12-dimethoxy-3-ol	Food related
2,5-Dimethylbenzaldehyde	Food related
(E)-3-(2,3-Dihydroxyphenyl)-2-propenoicacid	Microbiota; Food related
1,2,3,4-Tetrahydro-b-carboline-1,3-dicarboxylicacid	Food related
2,3-Dimethylphenol	Food related
Bakersyeastextract	Food related
Glyceryl5-hydroxydecanoate	Food related
3-Hydroxy-4-phenylbutan-2-one	Food related
Menthylpyrrolidonecarboxylate	Food related
2-Methylbutylamine	Food related
Polyethylene,oxidized	Food related
Polyoxyethylene40monostearate	Food related
1,1-(Tetrahydro-6a-hydroxy-2,3a,5-trimethylfuro[2,3-d-1,3-dioxole-2,5-diyl)bis-ethanone	Food related
Cis-3-Hexenylacetate	Food related
Triethanolamine	Food related; Drug related; Environment
Valeraldehydepropyleneglycolacetal	Food related
Diphenylamine	Food related; Environment
(4-Ethoxyphenyl)urea	Food related
2-Hydroxyacetophenone	Food related
4-Methoxychalcone	Food related
Zingerone	Food related; Drug related

4-Propylphenol	Food related
BatatasinIV	Food related
(S)-9-Hydroxy-10-undecenoicacid	Microbiota; Food related
Zeranol	Food related; Drug related
Isopropylbeta-D-glucoside	Food related
N-Methyl-1H-indole-3-propanamide	Food related
Nb-trans-Feruloylserotoninglucoside	Food related
Jasmonicacid	Microbiota; Food related
(R)-1-O-[b-D-Apiofuranosyl-(1-2)-b-D-glucopyranoside-1,3-octanediol	Food related
Niazirin	Food related
1-Methylnaphthalene	Microbiota; Food related; Environment
Sedanonicacid	Food related
1-(2-Furanyl)-2-propanone	Food related
3-Furanmethanolglucoside	Food related
1-Octen-3-ylglucoside	Food related
1-Octen-3-ylprimeveroside	Food related
7-Hydroxy-6-methyl-2H-1-benzopyran-2-one	Food related
(2R,3R)-(-)-2,3-Butanediol	Microbiota; Food related; Drug related
2,3-Butanediolapiosylglucoside	Food related
(E)-2-Methyl-2-buten-1-olO-beta-D-Glucopyranoside	Food related
(-)-erythro-Anetholeglycol2-glucoside	Food related
3-(1-Hydroxymethyl-1-propenyl)pentanedioicacid	Food related
KanzonolZ	Food related
SemilepidinosideB	Food related
NeoacrimarineK	Food related
Curcumenone	Food related
P-CHLOROPHENYLALANINE	Food related
Theaflagallin	Food related
7-(4-Hydroxyphenyl)-1-phenyl-4-hepten-3-one	Food related
(4S,6S)-3,4,5,6-Tetrahydro-4-hydroxy-6-methyl-2H-pyran-2-one	Food related
MollicellinH	Food related
Fagomine	Food related
(9Z,11R,12S,13S,15Z)-12,13-Epoxy-11-hydroxy-9,15-octadecadienoicacid	Food related
GonyautoxinVI	Food related
4,6-Dihydroxy-2-quinolinecarboxylicacid	Food related
Schaftoside	Food related
Melilotoside	Food related
Safrole	Food related; Environment
N5-(4-Methoxybenzyl)glutamine	Food related
(S)-[8-Gingerol	Food related
(1S,2S,4S,5S)-2,4-Thujanediol4-O-beta-D-Glucopyranoside	Food related
Dihydrohydroxy-O-methylsterigmatocystin	Food related

Undecylenicacid	Food related; Drug related
Cyperine	Food related
4-Heptenoicacid	Food related
Isoprenylalcohol	Microbiota; Food related
Lotaustralin	Microbiota; Food related
Glycyrol	Microbiota; Food related
Ixocarpanolide	Food related
Tomenin	Food related
Acetophenone	Microbiota; Food related; Drug related
(+/-)-erythro-Isoleucine	Food related
Cajanol	Food related
3-Hydroxymugineicacid	Food related
2-Deoxy-L-ribono-1,4-lactone	Food related
Curcumenol	Food related
Ethylbenzoate	Food related
7-Methoxy-2-methylisoflavone	Food related
2,7-Dihydroxy-4,6-dimethoxyisoflavan	Food related
Cristacarpin	Food related
6-O-Acetylarbutin	Food related
WithaperuvinH	Food related
SinapicAcid	Food related
WithaperuvinC	Food related
24-Epibrassinolide	Food related
(3R)-3,4-Dihydroxy-3-(hydroxymethyl)butanenitrile4-glucoside	Food related
Chavicol	Food related
FrenolicinB	Food related
1-Methoxy-4-(2-propenyl)benzene	Food related
Toluene	Microbiota; Food related; Drug related; Environment
2-Hydroxybenzaldehyde	Microbiota; Food related
Salicylaldehyde	Microbiota; Food related
LicorisoflavanA	Microbiota; Food related
5-Deoxykievitone	Food related
AdouetineX	Food related
Chrysoobtusin	Food related
HygromycinB	Food related
Styrene	Food related; Environment
Isoquinoline	Food related; Drug related
2-Aminoheptanedioicacid	Food related
L-Coprine	Food related
5,6-Dihydro-5-hydroxy-6-methyl-2H-pyran-2-one	Food related
Floionolicacid	Microbiota; Food related
(9Z,12R)-12-Hydroxyoctadec-9-enoicacid	Food related
Patulin	Microbiota; Food related; Drug related
Piperidine	Microbiota; Food related

Isopimpinellin	Food related
S-Allylcysteine	Food related
Desglucocheirotoxin	Food related
L-Theanine	Food related; Drug related
Imperatorin	Microbiota; Food related
Phaseollinisoflavan	Food related
1-(2,6-Dihydroxy-4-methoxyphenyl)-3-phenyl-1-propanone	Food related
Neocnidilide	Food related
Exaltolide	Food related
Heptyl4-hydroxybenzoate	Microbiota; Food related; Environment
NiveusinC	Food related
Valtrate	Food related
(3E,7E)-4,8,12-Trimethyl-1,3,7,11-tridecatetraene	Food related
BarringtogenolC	Food related
7-Nonene-3,5-diyn-1-ol	Food related
3-Hydroxy-T2-triol	Food related
ShoyuflavoneB	Food related
(5alpha,8beta,9beta)-5,9-Epoxy-3,6-megastigmadien-8-ol	Food related
CinncassiolD2	Food related
3-Hydroxy-4-butanolide	Food related
Thalictroidine	Food related
Artemin	Food related
Fumonisinb1	Food related
Curdione	Food related
Vaccinoside	Food related
Epidermin	Food related
Capsiate	Food related
Dihydrocapsiate	Food related
LansiumarinA	Food related
Methyl(Z)-5-(1-propynyl)-2-thienylacrylate	Food related
3-Carboxy-2,3,4,9-tetrahydro-1H-pyrido[3,4-bindole-1-propanoicacid	Food related
3alpha,4,5,7alpha-Tetrahydro-5-hydroxy-1H-isoindole-1,3(2H)-dione	Food related
3alpha,4,7,7alpha-Tetrahydro-4-hydroxy-1H-isoindole-1,3(2H)-dione	Food related
(S)-3-[(Cyanophenylmethyl)amino-3-oxopropanoicacid	Food related
Geniposidicacid	Food related
Capillene	Food related
Sambunigrin	Food related
Prulaurasin	Host; Food related
Acacetin7-[apiosyl(1-6)-glucoside	Food related
Alpha-Cyperol	Food related
(x)-2-Heptanolglucoside	Food related

Dehydrocyanaropicrin	Food related
Phaseolusepsilon	Food related
Epinepetalactone	Food related
(R)-Carvone	Microbiota; Food related
(S)-Abscisicacid	Food related
Nerol	Microbiota; Food related; Drug related; Environment
Paullinicacid	Microbiota; Food related
CinncassiolA	Food related
Beta-Thujaplicin	Microbiota; Food related; Drug related
Dihydroactinidiolide	Food related
1-Phenyl-1,2-propanedione	Food related
Taraxinicacidglucosylester	Food related
Solasodine	Food related
GanolucidicacidD	Food related
SterebinA	Food related
N-Methyl-1-deoxynojirimycin	Food related
CitreoviridinolA1	Food related
T2Triol	Food related
Dehydrocurdione	Food related
Kaempferol3-(6-acetylgalactoside)	Food related
2,3,4,5-Tetrahydroxy-4-prenylstilbene	Food related
Xi-2,3-Dihydro-2-oxo-1H-indole-3-aceticacid	Microbiota; Food related
(Z)-3-Methyl-2-(2-pentenyl)-2-cyclopenten-1-one	Food related
BetulalbusideA	Food related
3,14-Dihydroxy-11,13-dihydrocostunolide	Food related
Melleolide	Food related
Matairesinol	Food related; Drug related
LucidenicacidJ	Food related
Aloesol	Food related
(1R,2S,3S,4R)-p-Menthane-2,3-diol	Food related
DukunolideD	Food related
Arlatin	Food related
Phytuberin	Microbiota; Food related
8-Hydroxy-4(6)-lactarene-5,14-diol	Food related
Piperdial	Food related
S-Japonin	Food related
Genipin	Food related
13-Hydroxy-5-O-methylmelledonal	Food related
11-Hydroxy-9-tridecenoicacid	Food related
Germacrone4,5-epoxide	Food related
PhyllanthusolB	Food related
7(14)-Bisabolene-2,3,10,11-tetrol	Food related
CorchorifattyacidF	Microbiota; Food related
Isoalantolactone	Microbiota; Food related

(10Z,14E,16E)-10,14,16-Octadecatrien-12-ynoicacid	Food related
MomordicinII	Food related
LucidenicacidM	Microbiota; Food related
GanodericacidF	Food related
Cinnzeylanol	Food related
Dehydrooreadone	Food related
Tenuazonicacid	Microbiota; Food related
Vulgarin	Food related
P-Menthane-3,8-diol	Food related
4,11,13,15-TetrahydroridentinB	Food related
Arabsin	Food related
3-(Hydroxymethyl)-2-heptanone	Food related
2-Benzylidene-1-heptanol	Food related
GladiatosideC2	Food related
GladiatosideC1	Food related
(E)-2-Glucosyl-3,4,5-trihydroxystilbene	Food related
9,13-Dihydroxy-4-megastigmen-3-one9-glucoside	Food related
Xi-2,3-Dihydro-3,5-dihydroxy-6-methyl-4H-pyran-4-one	Food related
Cynaropicrin	Food related
Chamazulene	Food related; Drug related
HydroxyvalerenicAcid	Food related
Beta-Ionone	Food related
ValidamycinA	Microbiota; Food related
3,4-Dihydro-2H-1-benzopyran-2-one	Food related
Phlorizin	Food related; Drug related
Furanofukinin	Food related
Matricin	Food related
Guaiazulene	Food related; Drug related
Jacarandicacid	Microbiota; Food related
2beta,9xi-Dihydroxy-8-oxo-1(10),4,11(13)-germacratrien-12,6alpha-olide	Food related
Hydroxypelenolide	Food related
Cyperolone	Food related
3-Epinobilin	Food related
Heliangin	Food related
YucalexinP8	Food related
Zederone	Food related
Tavulin	Food related
DiosbulbinosideF	Food related
Dehydrovomifoliol	Food related
3-Hydroxy-beta-ionone	Food related
Sclareol	Food related
IcarisideB8	Food related
CinncassiolD3	Food related

CinncassiolC3	Food related
GinkgolideA	Food related; Drug related
Marasmal	Food related
GibberellinA53	Food related
GarcimangosoneC	Food related
(1alpha,4alpha,5beta,6alpha,11betaH)-1,4-Epoxy-5-hydroxy-10(14)-germacren-12,6-olide	Food related
5-Hydroxy-p-mentha-6,8-dien-2-one	Food related
Sporol	Food related
5,8-Dimethoxychalepensin	Food related
(R)-8-Acetoxycarvotanacetone	Food related
Polysorbate80	Food related; Drug related
5,8-Dihydroxy-3-(4-hydroxybenzyl)-7-methoxy-4-chromanone8-acetate	Food related
(R)-2-Hydroxy-7,8-dimethoxy-2H-1,4-benzoxazin-3(4H)-one2-glucoside	Food related
2-Ethoxy-1-methyl-4-(1-methylethyl)benzene	Food related
2-Methyl-3-(2-pentenyl)-2-cyclopenten-1-one	Food related
Cerasinone	Food related
Isoliquiritin	Microbiota; Food related
Traumatin	Food related
(1S,3R,4R)-p-Menthane-1,3-diol	Food related
Kaempferide	Food related
Trilobatin	Food related
Sandoricin	Food related
6-Hydroxysandoricin	Microbiota; Food related
3,7,8,15-Scirpenetetrol	Food related
6-beta-D-Glucopyranosyl-4,5-dihydroxy-3,7-dimethoxyflavone	Microbiota; Food related
6-p-Coumaroylprunin	Food related
4-Hydroxy-2,6,6-trimethyl-3-oxo-1,4-cyclohexadiene-1-carboxaldehyde	Food related
10beta-12,13-Dinor-8-oxo-6-eremophilen-11-al	Food related
(10betaH,11xi)-11-Hydroxy-13-nor-6-eremophilen-8-one	Food related
13-Nor-6-eremophilene-8,11-dione	Food related
CoriandroneB	Food related
CrassostreaSecocarotenoid	Food related
(3S,3S,5R,5R,6R)-3,6-Epoxy-5,6-dihydro-3,5,8-trihydroxy-beta,kappa-caroten-6-one	Food related
2-Propenylcyclohexanepentanoate	Food related
Glucosyl(2E,6E,10x)-10,11-dihydroxy-2,6-farnesadienoate	Food related
(1xi,3xi)-1,2,3,4-Tetrahydro-1-methyl-beta-carboline-3-carboxylicacid	Food related
Catechin7-glucoside	Food related
(+/-)-3,4-Methylenedioxy-5,7-dimethylepicatechin	Food related

Cryptomeridiol11-rhamnoside	Food related
2-Propenyl3-methylbutanoate	Food related
(R)-2,7-Dihydroxy-2H-1,4-benzoxazin-3(4H)-one	Food related
Dehydrophytosphingosine	Food related
(3E,6Z)-Nonadien-1-ylacetate	Food related
Quinceoxepine	Food related
Kievitonehydrate	Food related
Kievitol	Food related
Junosine	Food related
3,11,12-Trihydroxy-1(10)-spirovetiven-2-one	Food related
2-(2-Hydroxy-4-methylphenyl)-3-pentanone	Food related
Peperinicacid	Food related
Alpha-BisabololoxideA	Food related
Alpha-BisabololoxideB	Food related
Zedoarol	Food related
5-Isopropyl-2-(2-methylpropyl)-2-cyclohexen-1-one	Food related
PterosinN	Food related
Cis-3-Hexenylpyruvate	Food related
Xi-Tetrahydro-6-propyl-2H-pyran-2-one	Food related
(R)-2-Hydroxy-2H-1,4-benzoxazin-3(4H)-one	Food related
VerimolA	Food related
3-HydroxybenzylAlcohol	Food related
Alpha-Hydroxy-1-methyl-1H-indole-3-propanoicacid	Food related
LucidenicacidN	Microbiota; Food related
GambiriinA1	Food related
Glucosinalbin	Food related
Auxina	Food related
PolyporusteroneB	Food related
Cibaricacid	Food related
Dihydrophaseicacid	Food related
6-Methyl-2-methylene-6-octene-1,3,8-triol	Food related
PerillosideA	Food related
4,7-Megastigmadien-9-ol	Food related
(3S,5R,6R,7E)-3,5,6-Trihydroxy-7-megastigmen-9-one	Food related
N-Hydroxy-L-tyrosine	Food related
10-Hydroxymelleolide	Food related
(1(10)E,4a,5E)-1(10),5-Germacradiene-12-acetoxy-4,11-diol	Food related
2,4-Dihydroxy-4,6-dimethoxy-3-prenylchalcone	Microbiota; Food related
Maltotriose	Food related
4-O-beta-D-Galactopyranosyl-D-xylose	Food related
Sesamose	Food related
5-O-a-L-Arabinofuranosyl-L-arabinose	Food related
Syringaresinol	Microbiota; Food related
(-)-11-Hydroxy-9,15,16-trioxooctadecanoicacid	Food related

(1beta,2beta,5beta)-p-Menth-3-ene-1,2,5-triol	Food related
(3b,4b,5b)-4,5-Epoxy-p-menth-1-en-3-ol3-glucoside	Food related
(S)-p-Menth-1-ene-4,7-diol4-glucoside	Food related
(S)-17-Hydroxy-9,11,13,15-octadecatetraynoicacid	Food related
Nepetariaside	Food related
Trans-Grandmarin	Food related
(4S,8R)-8,9-Dihydroxy-p-menth-1(6)-en-2-one	Food related
Chrysophanein	Food related
N-Carboxyacetyl-D-phenylalanine	Food related
(2S,3S)-alpha-Amino-2-carboxy-5-oxo-1-pyrrolidinebutanoicacid	Food related
Pisatoside	Food related
Citrubuntin	Food related
(R)-11,12,13-Trinor-1(5),6,9-guaiatrien-8-one	Food related
Kaempferol3-rhamnoside4-xyloside	Food related
SoyasapogenolB3-O-b-D-glucuronide	Food related
3,4,5-Trihydroxy-3,7-dimethoxyflavone5-glucoside	Food related
Dihydroprudomenin	Food related
GinsenosideRh6	Food related
Macaridine	Food related
2-(Malonylamino)benzoicacid	Food related
4-Dihydroabscisicacid	Food related
Ethoxyquin	Food related; Drug related; Environment
(E)-10-Hydroxy-8-decenoicacid	Food related
BLII	Food related
Pergillin	Food related
Sorgolactone	Food related
4-Formyl-3-(formylmethyl)-4-hexenoicacid	Food related
5-(2-Furanyl)-1,2,3,4,5,6-hexahydro-7H-cyclopenta[bpyridin-7-one	Food related
1,2,3,4,5,6-Hexahydro-5-methyl-7H-cyclopenta[bpyridin-7-one	Food related
2,3,6,7-Tetrahydro-7-methylcyclopent[bazepin-8(1H)-one	Food related
5-Ethyl-1,2,3,4,5,6-hexahydro-7H-cyclopenta[bpyridin-7-one	Food related
3-O-alpha-D-Glucopyranuronosyl-D-xylose	Food related
4,4alpha,5,6-Tetrahydro-7-methyl-2(3H)-naphthalenone	Food related
A-L-Arabinofuranosyl-(1-2)-[a-D-mannopyranosyl-(1-6)-D-mannose	Food related
Serratol	Food related
Eriodictyol7-(6-trans-p-coumaroylglucoside)	Food related
3,4-Dihydroxy-2-hydroxymethyl-1-pyrrolidinepropanamide	Food related
Menthone1,3-glycerylketal	Food related
2,3-Dihydro-6-methyl-1H-pyrrolizine-5-carboxaldehyde	Food related
Hexyl2-methyl-3-pentenoate	Food related
Glycerol1-(5-hydroxydodecanoate)	Food related
Menthonelactone	Food related
1-Propene-1-thiol	Food related

5-Hydroxy-3,4,7-trimethoxyflavan	Food related
4,5-Dihydrovomifoliol	Food related
Dulcin	Food related
4-Hydroxy-6-methyl-3-(1-oxobutyl)-2H-pyran-2-one	Food related
16,17-Dihydro-16alpha,17-dihydroxygibberellinA717-glucoside	Food related
9-Hydroxy-7-megastigmen-3-oneglucoside	Food related
Allixin	Food related
D-Linalool3-(6-malonylglucoside)	Food related
(3beta,8beta)-3-Hydroxy-7(11)-eremophilen-12,8-olide	Food related
Eremopetasidione	Food related
2,3-Butanediolglucoside	Food related
(S)-11,12,13-Trinor-7-calamenone	Food related
3-[4-Hydroxy-3-(3-methyl-2-butenyl)phenyl-2-propenal	Food related
Methyl(3x,10R)-dihydroxy-11-dodecene-6,8-diynoate10-glucoside	Food related
Ipomeatetrahydrofuran	Food related
CoixindenB	Food related
1,17-Heptadecanediol	Food related
P-Coumaraldehyde	Microbiota; Food related
1-Acetoxychavicolacetate	Food related
Sugeonol	Food related
5,7-Megastigmadien-9-olglucoside	Food related
KanzonolM	Food related
Prenylcis-caffeate	Food related
AntibioticSB202742	Food related
Methyl(3b,11x)-3-Hydroxy-8-oxo-6-eremophilen-12-oate	Food related
Pantoyllactoneglucoside	Food related
Uralenneoside	Food related
16-Hydroxy-10-oxohexadecanoicacid	Food related
25-Acetylvulgaroside	Food related
Exo,exo-1,8-Epoxy-p-menthane-2,6-diol	Food related
ErinapyroneA	Food related
ErinapyroneB	Food related
2,3-Dihydro-2,3-dihydroxy-4-(4-methoxyphenyl)-1H-phenalen-1-one	Food related
Benzylformate	Food related; Drug related
Beta-D-Glucopyranosylanthranilate	Food related
(1R,2R,4R)-1,8-Epoxy-p-menthane-2,4-diol	Food related
3,8-Dihydroxy-6-methoxy-7(11)-eremophilen-12,8-olide	Food related
4-Megastigmene-6a,9R-diol9-[apiosyl-(1-6)-glucoside	Food related
SapidolideA	Food related
Coumarinicacid	Microbiota; Food related
2-Hydroxycinnamicacid	Microbiota; Food related
Lactose	Host; Microbiota; Food related
4-Hydroxy-(3,4-dihydroxyphenyl)-valericacid	Food related

Cis-Resveratrol4-O-glucuronide	Food related
Dihydrocaffeicacid3-sulfate	Food related
Dihydroferulicacid4-O-sulfate	Food related
Dihydrosinapicacid	Food related
Equol4-O-glucuronide	Food related
Equol7-O-glucuronide	Food related
Trans-Resveratrol3-O-glucuronide	Food related
Trans-Resveratrol4-sulfate	Food related
2-Naphthylamine	Environment
Beta-Zearalenol	Food related
Alpha-Zearalenol	Food related
Isonicotinicacid	Host; Drug related
Ketobemidone	Drug related
3,4-Methylenedioxyamphetamine	Drug related
N-Acetyldopamine	Food related
Toluidine	Food related; Environment
Ritalinicacid	Unknown
DG(15:0/0:0/16:1n7)	Food related
CL(18:0/22:5(7Z,10Z,13Z,16Z,19Z)/20:4(5Z,8Z,11Z,14Z)/18:0)	Host; Food related
CL(18:1(11Z)/18:1(11Z)/20:4(5Z,8Z,11Z,14Z)/18:0)	Host; Food related
5-phosphonooxy-L-lysine	Host; Microbiota
1-Indanol	Unknown
Thiomorpholine3-carboxylate	Unknown
4-Methylumbelliferone	Drug related
(9S,10S)-9,10-dihydroxyoctadecanoate	Food related
2-HydroxynicotinicAcid	Unknown
3,4-Methylenesebacicacid	Food related
4-Hydroxy-3-methoxy-cinnamoylglycine	Unknown
2,6-Dimethylnaphthalene	Food related
3-Methylsubericacid	Food related
M-Xylene	Microbiota; Food related; Environment
Ethyl3-hydroxydodecanoate	Food related
3-Ethylphenol	Food related
Alpha-Ionone	Food related
Ethylbenzene	Microbiota; Food related; Environment
2-(1-Ethoxyethoxy)propanoicacid	Unknown
5-(3,4,5-Trihydroxyphenyl)-gamma-valerolactone-3-O-sulphate	Food related
Neomenthol-glucuronide	Food related
Pyrogallol-2-O-glucuronide	Food related
Hyaluronanbiosynthesis,precursor1	Unknown
Cis-Melilotoside	Microbiota; Food related
12-O-D-Glucuronoside-13-hydroxyoctadec-9Z-enoate	Food related
L-3-Cyanoalanine	Microbiota; Food related
3-Hydroxykynurenamine	Host; Microbiota

Uridine3-monophosphate	Microbiota; Drug related
(7R)-7-(5-Carboxy-5-oxopentanoyl)aminocephalosporinate	Microbiota
2-Oxosuccinamate	Host; Microbiota
3-Carbamoyl-2-phenylpropionicacid	Host
3-Methoxyanthranilate	Unknown
3-Polyprenyl-4,5-dihydroxybenzoate	Host; Microbiota
4-Amino-1-piperidinecarboxylicacid	Unknown
4-Hydroxy-3-polyprenylbenzoate	Food related
4-Hydroxy-5-phenyltetrahydro-1,3-oxazin-2-one	Host
4-HydroxyphenylaceticAcid	Host; Microbiota
5-Phenyl-1,3-oxazinane-2,4-dione	Host
5-Deoxy-5-fluorocytidine	Host; Microbiota
5-Deoxy-5-fluorouridine	Host; Microbiota; Drug related
Didemethylcitalopram	Host; Microbiota
Gamma-Glutamyl-beta-aminopropiononitrile	Microbiota
L-Formylkynurenine	Host; Microbiota
N-Acetylmuramoyl-Ala	Unknown
Sterol	Food related
KukoamineC	Food related
KukoamineD	Food related
Nirvanol	Unknown
Perindoprilat	Drug related
P-Hydroxyfelbamate	Host
2-Hydroxyfelbamate	Host; Food related
2-Propyl-2,4-pentadienoicacid	Food related
2-n-Propyl-4-oxopentanoicacid	Unknown
2-PropylglutaricAcid	Food related
3-Oxovalproicacid	Unknown
Alcoifosfamide	Unknown
3-O-Methyl-a-methyldopa	Unknown
4-Carboxynevirapine	Unknown
4-Hydroxyphenytoinglucuronide	Unknown
5-Carboxy-2-deoxyuridine	Microbiota
6-allyl-8b-Carboxy-ergoline	Unknown
Melatoninglucuronide	Unknown
Meta-O-Dealkylatedflecainidelactam	Unknown
N,N,O-Tridesmethyltramadol	Unknown
P-O-glucuroniderosiglitazone	Unknown
7-Hydroxy-R-phenprocoumon	Unknown
Fluvoxaminoacid	Unknown
10-alpha-methoxy-9,10-dihydrolysergol	Unknown
Noralfentanil	Unknown
3-demethyletoposide	Unknown
27-O-demethylrifabutin	Unknown

5-ethyl-5-methyl-2,4-oxazolidinedione	Unknown
Dihydroartemisinin(DHA)	Food related
3-Carboxy-4-methyl-5-propyl-2-furanpropionicacid	Food related; Drug related
4-Hydroxy-alprenolol	Unknown
Monicacid	Food related
N,N,O-Tridesmethylvenlafaxine	Unknown
N-(3-acetamidopropyl)pyrrolidin-2-one	Unknown
3-carboxy-4-methyl-5-pentyl-2-furanpropanoicacid	Food related
3,4-Dimethyl-5-pentyl-2-furantridecanoicacid	Food related
9,10-Epoxyoctadecanoicacid	Microbiota; Food related
3-hydroxypristanicacid	Food related
3-hydroxytridecanoicacid	Food related
3,4,5,6-Tetrahydrohippuricacid	Unknown
6-(2-Hydroxyethoxy)-6-oxohexanoicacid	Food related
N-(5-Amino-2-hydroxybenzoyl)glycine	Unknown
5-Hydroxypentanoicacid	Microbiota; Food related; Drug related
N-lactoyl-phenylalanine	Unknown
N-Lactoylphenylalanine	Unknown
N-Lactoylisoleucine	Unknown
N-lactoyl-Methionine	Unknown
3alpha,7alpha,12alpha-trihydroxy-5beta-cholestanate	Host; Food related
1-(3-Pyridinyl)-1,4-butanediol	Unknown
1,2-Dihydroxy-3,4-epoxy-1,2,3,4-tetrahydronaphthalene	Unknown
1-(11Z-eicosenoyl)-glycero-3-phosphate	Food related
1-(4Z,7Z,10Z,13Z,16Z,19Z-docosahexaenoyl)-glycero-3-phosphate	Food related
1-(5Z,8Z,11Z,14Z-eicosatetraenoyl)-sn-glycero-3-phosphate	Food related
(6R,8Z)-6-Hydroxy-3-oxotetradecenoicacid	Unknown
4-[(Hydroxymethyl)nitrosoamino-1-(3-pyridinyl)-1-butanone	Host
4alpha-carboxy-5alpha-cholesta-8,24-dien-3beta-ol	Host; Food related
5-(3-Pyridyl)-2-hydroxytetrahydrofuran	Unknown
6-[2,3-Dihydroxy-1-(hydroxymethyl)propyl-1,2-dihydro-7-hydroxy-9-methoxy-cyclopenta[c[1benzopyran-3,4-dione	Unknown
Alpha-(1,2-Dihydroxyethyl)-1,2,3,4-tetrahydro-7-hydroxy-9-methoxy-3,4-dioxocyclopenta[c[1benzopyran-6-acetaldehyde	Unknown
Alpha-[3-[(Hydroxymethyl)nitrosoaminopropyl-3-pyridinemethanol	Unknown
Benzo[apyrene-7,8-dihydrodiol-9,10-oxide	Host
NeuNGc	Host; Microbiota
TetraHCA	Food related
16alpha-HydroxyDHEA3-sulfate	Food related
UrocanicAcid	Host; Microbiota
(3R,5S)-1-pyrroline-3-hydroxy-5-carboxylicAcid	Host; Microbiota
ProstaglandinG22-glycerylEster	Food related
2,3-dinor-6-oxoprostaglandinF1alpha	Food related
N(2)-phenylacetyl-L-glutaminate	Unknown

N-Methyl-L-glutamate	Microbiota
Chitobiose	Host; Microbiota
3-ethylphenylSulfate	Food related
(R)-3-hydroxybutyrylcarnitine	Food related
12(S)-HETrE	Food related
EcgonineMethylEster	Food related; Drug related
Epsilon-caprolactam	Food related
TG(8:0/8:0/i-17:0)	Host; Food related
MG(a-15:0/0:0/0:0)[rac	Food related
N-Acetylproline	Drug related
9-Oxo-nonanoicacid	Food related
Hydroxybutyrylglycine	Unknown
3,4-Dimethyl-5-pentyl-2-furanpropanoicacid	Food related
PS(18:4(6Z,9Z,12Z,15Z)/16:1(9Z))	Host; Food related
6-(3-carboxyphenoxy)-3,4,5-trihydroxyoxane-2-carboxylicacid	Unknown
6-[4-(carboxymethyl)-2-hydroxyphenoxy-3,4,5-trihydroxyoxane-2-carboxylicacid	Unknown
3-[4-(sulfooxy)phenylpropanoicacid	Unknown
3,5-dihydroxy-4-(sulfooxy)benzoicacid	Unknown
Apigenin7,4-dimethylether	Food related
Coniferaldehyde	Microbiota; Food related
2,8-Quinolinediol	Unknown
Pelargonidin3-glucoside	Food related
Urolithin-3-sulfate	Food related
LysoPS(16:0/0:0)	Unknown
GlycohyocholicAcid	Unknown
Ectoine	Microbiota
Succinimide	Drug related
8-Amino-7-oxononanoicacid	Microbiota; Drug related
(9Z,12Z)-3-Hydroxyhexadecadienoylcarnitine	Unknown
3-Hydroxy-cis-5-octenoylcarnitine	Unknown
Palmitoylcarnitine	Host; Microbiota; Food related
(4Z)-dec-4-enedioylcarnitine	Unknown
4-Hydroxydecanedioylcarnitine	Unknown
Undeca-5,7,9-trienedioylcarnitine	Unknown
Dodeca-3,6,9-trienedioylcarnitine	Unknown
3,11-Dihydroxydodecanoylcarnitine	Unknown
16-Hydroxyhexadecanoylcarnitine	Unknown
(7Z)-Octadec-7-enoylcarnitine	Unknown
2-Tiglylcarnitine	Unknown
(3E)-Glutaconylcarnitin	Unknown
Hept-5-enoylcarnitine	Unknown
3-Hydroxyhept-4-enoylcarnitine	Unknown
2-Hydroxyhept-5-enoylcarnitine	Unknown

(2E)-Hept-2-enedioylcarnitine	Unknown
3-Hydroxy-6-octenoylcarnitine	Unknown
2-Hydroxy-4-octenoylcarnitine	Unknown
Octa-3,5-dienedioylcarnitine	Unknown
4-Amino-3-hydroxybutanoylcarnitine	Unknown
(5Z,8Z,11Z,13E,15S)-15-Hydroperoxyicosa-5,8,11,13-tetraenoylcarnitine	Unknown
N-PalmitoylCysteine	Unknown
N-PalmitoylHistidine	Unknown
N-OleoylHistidine	Unknown
N-LinoleoylHistidine	Unknown
N-LinoleoylLeucine	Unknown
N-DocosahexaenoylAsparagine	Unknown
N-LauroylIsoleucine	Unknown
N-LauroylLysine	Unknown
Delta-CEHC	Unknown
(R)-1-Methylpiperidine-2-carboxylicacid	Unknown
N(5)-Acetylornithine	Unknown
Docosanedioicacid	Food related
(2s)-2-[6-(Sulfooxy)naphthalen-2-ylpropanoicacid	Microbiota
Octadeca-2,4-dienedioicacid	Unknown
(-)-2-Difluoromethylornithine	Drug related
(+-)-Carbovir	Unknown
Artemisinin	Drug related
Isofebrifugine	Unknown
(+)-Lysergicacid	Unknown
(1r,3r)-1-Aminocyclopentane-1,3-dicarboxylicacid	Unknown
Cholylphenylalanine	Unknown
Cholylhistidine	Unknown
TaurochenodeoxycholicAcid	Host; Microbiota; Food related; Drug related
Deoxycholylmethionine	Unknown
(2-Ethyl-2,4,4-trimethyl-1,3-oxazolidin-3-yl)oxidanyl	Unknown
(2-Phenylallyl)hydrazine	Unknown
Diaminopimelicacid	Microbiota; Food related
D-Glucose	Host; Food related
(2s,3r,4s,5r)-3,4,5-Trihydroxy-6-oxopiperidine-2-carboxylicacid	Unknown
Ecdysterone	Food related
Glucosylsphingosine	Food related
(2S,3R,4S,5R,6R)-6-Ethyloxane-2,3,4,5-tetrol	Unknown
Integerrimine	Unknown
Monensin	Drug related
Ptaquiloside	Unknown
(-)-Abscisicacid	Unknown
AbscisicAcid	Food related; Drug related

Anisomycin	Drug related
(+)-cis-Khellactone	Unknown
Dihydrotestosterone	Host; Food related; Drug related
(3R)-3-Hydroxy-N-[(3S)-2-oxooxolan-3-ylbutanamide	Unknown
Equol	Microbiota; Food related; Drug related
[(1R,5R)-5-(6-Aminopurin-9-yl)cyclohex-3-en-1-ylmethanol	Unknown
2-Methylthiazolidine-4-carboxylicacid	Unknown
1-(2-Aminoacetyl)-N-(4-nitrophenyl)pyrrolidine-2-carboxamide	Unknown
1-[3,4-Dihydroxy-5-(hydroxymethyl)oxolan-2-yl-4-hydroxyhydropyridin-2-one	Unknown
1-Aminocyclohexanecarboxylicacid	Unknown
1-Cyclohexene-1-carboxylicacid	Unknown
1-Hydroxy-2,2,5,5-tetramethylpyrrolidine-3-carboxamide	Unknown
1-Methyl-4-piperidylacetate	Unknown
1-Methyladenosine	Food related
1-Methyladenosin	Unknown
1-Naphthylamine	Unknown
1-Nitrohydroxyphenyl-N-benzoylalanine	Unknown
N-Nitrosopiperidine	Unknown
1,10-Phenanthroline	Drug related
1,2-Cyclohexanediol	Unknown
1,2-Dihydroisoquinoline	Unknown
1,2-Naphthoquinone	Unknown
1,2,3,4-Tetrahydronaphthalene	Unknown
1,4-Diamino-2-chloro-2-butene	Unknown
1,5-Isoquinolinediol	Unknown
1,6-Hexanediamine	Drug related
N6-Etheno2-deoxyadenosine	Unknown
10-Hydroxy-2-decenoicacid	Unknown
10-Hydroxydecanoicacid	Unknown
2-Methyl-5-nitroimidazol-1-ylaceticacid	Unknown
11,17-Dihydroxy-3,20-dioxopregn-4-en-21-ylacetate	Unknown
Guanidine,(4-chloro-3-methoxy-1-oxo-1H-2-benzopyran-7-yl)-	Unknown
Articainicacid	Unknown
S-(6-Purinyl)cysteine	Unknown
N-(1-Adamantyl)urea	Unknown
2-Methyl-3-(2-methylphenyl)pyrido[2,3-dpyrimidin-4-one	Unknown
1-Tert-butyl4-ethyl5-oxoazepane-1,4-dicarboxylate	Unknown
4-Chloro-L-phenylalanine	Unknown
S-(2-(N,N-Diisopropylamino)ethyl)isothiourea	Unknown
Desmethylrizatriptan	Unknown
N-(3-Aminopropyl)-N-methylcarbamicacidtert-butylester	Unknown
N-alpha-Benzoyl-L-arginine	Unknown
2-Aminobicyclo[3.1.0hexane-2,6-dicarboxylicacid	Unknown

Adb-chminaca,(+/-)-	Unknown
Hexahydro-4-methylphthalicanhydride	Unknown
Oxindole	Unknown
1H-Pyrazolo[3,4-dpyrimidin-4-amine	Unknown
2-(2-Amino-1-hydroxyethyl)phenol	Unknown
2-[[(4-Hydroxyphenyl)azophenylbenzoicacid	Unknown
2-Amino-2-methyl-1-propanol	Unknown
2-Amino-3-cyclohexylpropanoicacid	Unknown
6-Methyl-5,6-dihydro-4H-1,3-thiazin-2-amine	Unknown
2-Aminobiphenyl	Unknown
2-Aminobutan-1-ol	Unknown
L-2-Aminobutyricacid	Host; Food related; Drug related
2-Aminopurine	Unknown
Beta-D-Galactosamine	Unknown
Deoxyribose	Host; Microbiota; Food related
2-Fluoroadenosine	Drug related
2-Hydroxyestriol	Unknown
2-Hydroxypropyloctanoate	Unknown
2-(4-Morpholinyl)ethyl	Unknown
2-Nitrophenol	Unknown
4-Hydroxynonenal	Food related
2-Nonene-1,4-diol	Unknown
2-Octynoicacid	Unknown
2-Phenylimidazo[1,2-apyridine	Unknown
2-Quinolinylmethanol	Unknown
2,2,6,6-Tetramethylpiperidine	Unknown
2,3-Dinor-TXB1	Unknown
2,4-Dichloro-6-nitrophenol	Unknown
2,4-Dinitrophenol	Microbiota; Food related; Drug related
2,5-Dihydro-1H-pyrrole-2-carboxylicacid	Unknown
2,6-Dichloro-4-nitrophenol	Unknown
2-Deoxy-2-methylenecytidine	Unknown
2-Fluoro-2,3-dideoxyadenosine	Unknown
2,3-Dideoxy-3-fluorouridine	Unknown
2,3-Didehydro-2,3-dideoxycytidine	Unknown
2,3-Dideoxyguanosine	Unknown
2,3-Dideoxyuridine	Unknown
2,5-Dideoxyadenosine	Unknown
20-hydroxyleukotrieneB4	Food related
9-(4-Fluoro-3-(hydroxymethyl)butyl)guanine	Unknown
21-Deoxycortisone	Unknown
Isopentenyladenine	Microbiota; Food related; Drug related
24-Fluorocalcitriol	Unknown
2-Deoxy-2,3-dehydro-n-acetyl-neuraminicacid	Unknown

26-Deoxyactein	Unknown
6-Hydroxy-5-methoxy-1h-indole-2-carboxylicacid	Unknown
3-(1H-Pyrrol-2-yl)propanoicAcid	Unknown
3-Aminopicolinaldehyde	Unknown
3-Butene-1,2-diol	Unknown
3-Gonal	Unknown
3-Hydroxycotinineglucuronide	Unknown
3-Iodo-alpha-methyl-l-tyrosine	Unknown
3-Methoxypropylamine	Unknown
3-O-DemethylfortimicinA	Unknown
3-Pyridinemethanol	Drug related
D-Gluconicacid,delta-lactone	Unknown
3,5,6,7-Tetramethoxyflavone	Unknown
Zidovudine	Drug related; Environment
3-Deoxyinosine	Unknown
3-Deoxythymidine	Unknown
3-Hydroxyamobarbital	Unknown
2,4,6(1H,3H,5H)-Pyrimidinetrione,5-ethyl-5-(3-hydroxy-1-methylbutyl)-	Unknown
3-thiacytidine	Unknown
Octaethyleneglycolmonododecylether	Unknown
1-[(2R,3S,5R)-3,4-Dihydroxy-5-(hydroxymethyl)oxolan-2-ylpyrimidine-2,4-dione	Food related
4-Aminophenylmannoside	Unknown
L-NIO	Drug related
Ethanone,1-(4-butylphenyl)-	Unknown
3h-Adrenaline	Unknown
4-(4-Fluorophenyl)piperidin-4-ol	Unknown
4-(Phenylamino)benzoicacid	Unknown
Fenamicacid	Unknown
4-(Phosphonomethyl)piperidine-2-carboxylicacid	Unknown
4-[(2,4-Dihydroxy-3,3-dimethylbutanoyl)aminobutanoicacid	Unknown
4-Amino-2,2,6,6-tetramethylpiperidin-1-ol	Unknown
Trans-4-Aminocyclohexanecarboxylicacid	Unknown
4-Chlorophenylaceticacid	Unknown
4-Deoxypyridoxine	Unknown
4-Heptyloxyphenol	Unknown
4-Heptylphenol	Drug related
4-Hydroxyquinoline	Unknown
4,4-Dihydroxystilbene	Unknown
4,5-Dimethoxybenzene-1,2-diamine	Unknown
4,6-O-Ethylidene-alpha-D-glucose	Unknown
4-Azidocytidine	Unknown
1,2,3,4-Tetrahydroisoquinoline-1-carboxylicacid	Unknown

4-Hydroxy-3-methyl-2-(2-propynyl)-2-cyclopentene-1-one	Unknown
4-(3-Hydroxypropyl)morpholine	Unknown
Navarixin	Drug related
D-Glucamine	Unknown
Phorbol-12,13-dibutyrate	Unknown
5-(2-Aminopropyl)benzofuran	Unknown
5-Benzylacyclouridine	Drug related
5-Hydroxy-6-methoxy-1h-indole-2-carboxylicacid	Unknown
5-Phenylhydantoin	Unknown
5,6-Dihydro-5-azacytidine	Drug related
5-Amino-5-deoxyadenosine	Unknown
2-Hydroxy-L-methionine	Unknown
6-Hydroxy-3,4-dihydro-2(1H)-quinolinone	Unknown
Alpha-Methylene-Gamma-Butyrolactone	Unknown
5Z-7-Oxozeaenol	Unknown
ACEXAMICACID	Unknown
D-Quinovose	Unknown
6-Fluoromevalonate	Unknown
6-Methyluracil	Unknown
6-Thioguanosine	Unknown
6,7-Dimethoxy-1,2,3,4-tetrahydroisoquinoline-3-carboxylicacid	Unknown
ProstaglandinJ2	Food related
4-Dimethylaminocinnamaldehyde	Unknown
Alpha-(Chloromethyl)-2-hydroxymethyl-5-nitroimidazole-1-ethanol	Unknown
N-[(3s)-2-Oxotetrahydrofuran-3-Ylbutanamide	Unknown
7-Amino-4-methylcoumarin	Drug related
7-Aminoheptanoicacid	Unknown
7-Deoxydoxorubicinone	Unknown
7,4-Dihydroxy-8-methylflavan	Unknown
7,4-Dihydroxyflavone	Food related
Inosine	Host; Microbiota; Food related; Drug related
Ara-HX	Unknown
1-Tert-Butyl4-ethyl3-oxopiperidine-1,4-dicarboxylate	Unknown
4-Bis(2-hydroxyethyl)amino-L-phenylalanine	Unknown
4-Amino-5-hydroxymethyl-2-methylpyrimidine	Microbiota; Food related; Drug related
3,4-Dehydrocilostazol	Unknown
(R)-2-(4-(Tert-butoxycarbonyl)morpholin-3-yl)aceticacid	Unknown
2-Amino-5-phosphonopentanoicacid	Unknown
4-((6-Methoxyquinolin-8-yl)amino)pentanoicacid	Unknown
8-Aminoguanosine	Unknown
8-Chloroinosine	Unknown
8,8-Diferulicacid	Microbiota; Food related; Drug related
ProstaglandinE3	Food related
N(G)-Nitroarginine-4-nitroanilide	Unknown

BafilomycinA1(Baf-A1)	Unknown
2-((4-Chlorophenyl)thio)-N-(4-(pyridin-2-yl)thiazol-2-yl)acetamide	Unknown
9-Octadecen-1-ol,(9Z)-	Unknown
Oleamide	Microbiota; Food related; Drug related
Dextran	Microbiota; Food related; Drug related
7-Methyl-2-(2-furyl)-1,8-naphthyridine-4(1H)-one	Unknown
Gemfibrozil1-O-beta-Glucuronide	Unknown
9h-Purin-9-amine	Unknown
9H-Purine-9-ol	Unknown
9(S)-HOTrE	Microbiota
LinoleicAcid	Host; Microbiota; Food related; Drug related; Environment
Pentanoicacid,5-(dipentylamino)-5-oxo-4-((3-quinolinylcarbonyl)amino)-,(R)-	Unknown
Abeado	Unknown
Abemaciclib	Drug related
2-(5-Methoxy-1H-indol-3-yl)ethylacetate	Unknown
Acarviosin	Unknown
2-(p-Acetamidophenyl)-2-ethylglutarimide	Unknown
Acetophenone,4-hydroxy-3-methoxy-2-(2-piperidyl)-	Unknown
Acetylshikonin	Unknown
Ala-Hyp-Gly	Unknown
Alaptide	Unknown
Alongside	Unknown
2-(Acetylamino)-2-deoxy-alpha-D-mannopyranose	Unknown
Alpha-Methyl-m-tyrosine	Unknown
Muramicacid	Microbiota; Food related
Therafectin	Drug related
Anisperimus	Unknown
5-Sulfamoyl-2-chloroadenosine	Unknown
Arasc	Unknown
Arginyl-glycyl-asparticacid	Unknown
Arginyl-glycyl-glutamyl-serine	Unknown
Aristospan	Drug related
ArphamenineB	Unknown
Artemisin	Unknown
Aurin	Unknown
Azimexon	Unknown
Aziridine	Food related
Aziridylbenzoquinone	Unknown
BaohuosideI	Unknown
7-Hydroxy-2-(4-hydroxyphenyl)-6-(3-methylbut-2-enyl)-2,3-dihydrochromen-4-one	Unknown
Bendiocarb	Food related
Benfuracarb	Unknown

Benzarone	Unknown
1,2,3-Benzotriazine	Unknown
Benzyledta	Unknown
Besifloxacin	Unknown
Bialaphos	Microbiota
Bilin	Unknown
Bms-707035	Unknown
Brexpiprazole	Drug related
Brivanibalaninate	Unknown
Butanedioicacid,octenyl-	Unknown
Butyl4-aminobenzoate	Drug related
CalceinBlue	Unknown
Camonagrel	Unknown
CANTHARIDIN	Drug related
Carbaryl	Drug related
Carboxyethyl-hydroxychroman	Unknown
Carboxyethyllysine	Unknown
Cbhcy	Unknown
Ceftazidimeanhydrous	Drug related
Cenisertib	Drug related
CephamycinC	Microbiota
Cepharanthine	Drug related
Cfp-aaf-pab	Unknown
AntcinK	Unknown
N-Dealkylatedderivative	Unknown
3beta-Hydroxy-17-(1H-imidazol-1-yl)androsta-5,16-diene	Unknown
(S,E)-2-(5-Hydroxy-8,9-dihydro-5H-benzo[7annulen-6(7H)-ylidene)aceticacid	Unknown
3-(2-Azetidinylmethoxy)-2-fluoropyridine	Unknown
Cholinesulfate	Microbiota
Chromane	Unknown
Chroman-2-ol	Unknown
Cimicifugoside	Unknown
Cimifugin	Unknown
Cis-Linoleicacid	Unknown
Cladosporin	Unknown
Cochinchinenin	Unknown
CombretastatinA4	Unknown
Coniferylferulate	Unknown
Coumafuryl	Unknown
Coumatetralyl	Unknown
3-Piperidinamine,N-((2-methoxyphenyl)methyl)-2-phenyl-,(2R,3R)-	Unknown
Creatininecitrate	Unknown
CucurbitacinI	Unknown

CucurbitacinS	Unknown
Cyclo(L-Phe-L-Pro)	Unknown
Cyclocreatine	Unknown
Cyclohexa-2,4-dienylmethanol	Unknown
1,2-Cyclohexanedicarboxylicacid	Unknown
Cyclohexene	Unknown
Cycloheximide	Drug related
Cyclohexyladenosine	Unknown
Cyclopentenylcytosine	Unknown
Cyclopropanecarboxamide,1,2,2,3,3-pentamethyl-	Unknown
D-Citrulline	Food related
D-Erythrose	Microbiota; Food related
GlucosaminicAcid	Unknown
D-Galactose	Host; Food related
D-Kynurenine	Food related
3-[[(2S)-2,4-Dihydroxy-3,3-dimethylbutanoylaminopropanoicacid	Food related; Drug related
D-Phenylalanine	Microbiota; Drug related
D,L-Buthionine	Unknown
Daumone	Unknown
Decursin	Unknown
Deferiprone	Drug related
Dehydromonocrotaline	Unknown
Deltaline	Unknown
Deoxyloganin	Unknown
Dexelvucitabine	Drug related
Dhv-PGE2	Unknown
Dioctylsebacate	Unknown
Diacetyl-L-tartaricAnhydride	Unknown
Dibenzo-18-crown-6	Unknown
Diflunisalglucuronideester	Unknown
Diflunisalglucuronideether	Unknown
Digitoxigeninbisdigitoxide	Unknown
D-Digitoxose	Unknown
Diglycolicacid	Unknown
Dihydrocorticosterone	Food related
Dimethylphthalate	Food related; Drug related
Dimethyl-3-hydroxypyrid-4-one	Unknown
Diterpenealkaloid	Unknown
DL-2,5-Dihydrophenylalanine	Unknown
Ala-Leu	Microbiota
DL-Allylglycine	Unknown
DL-Difluoromethylornithine	Unknown
3-Amino-2,3-dihydrobenzoicacid	Food related; Drug related
Methyldopa	Food related; Drug related; Environment

Methyldopate	Food related; Drug related; Environment
Norleucine	Food related; Drug related
Dodecylbenzenesulfonate	Unknown
Dropropizine	Drug related
Emtricitabine	Food related; Drug related
Ent-Emtricitabine	Unknown
Epanolol	Drug related
Eptifibatide	Drug related
Erythromycylamine	Unknown
Ethylmethylhydroxypyridinesuccinate	Unknown
3-C-Ethynylcytidine	Unknown
Levetiracetam	Drug related
Etiracetam	Drug related
4-Ethoxymethylene-2-phenyl-2-oxazolin-5-one	Unknown
Etoxazole	Unknown
Fantofarone	Unknown
Fasoracetam	Drug related
Favipiravir	Drug related
Cytidine,5-chloro-2,3-dideoxy-3-fluoro-	Unknown
Fenobucarb	Unknown
Flavonol3-O-D-glucoside	Unknown
Floctafenine	Drug related
Flumethasone	Drug related
Formetanate	Unknown
Formycinb	Unknown
Formyllysine	Unknown
Forodesine	Drug related
Fructoselactate	Unknown
Fructosylvaline	Unknown
Isoniazid	Host; Microbiota; Food related; Drug related; Environment
Fumagillol	Microbiota
FusaricAcid	Food related
Gabapentinenacarbil	Drug related
Gabazine	Unknown
Galactoselactate	Unknown
Gaxilose	Drug related
GentamicinB	Unknown
Germacrone	Food related
Gibberellins	Food related
Glucitol-lysine	Unknown
Glucoheptonicacid	Unknown
Glucomannan	Food related
Glucoselactatepyruvateglutamate	Unknown
Glucosepropionate	Unknown

Glufosinate	Drug related
Glutarimide	Unknown
Glutathioneperoxide	Unknown
Glycylprolylarginine	Unknown
Glycerolglutamate	Unknown
Glycerophosphoserine	Microbiota
Glycerylphosphorylinositol	Unknown
Glycyl-D-Alanine	Unknown
Glycyl-l-histidyl-l-lysine	Drug related
Glycyllysylarginine	Unknown
Glycylsarcosine	Unknown
2-[4-[4-(4-Carbamimidoylphenyl)piperazin-1-ylpiperidin-1-ylaceticacid	Unknown
Guadecitabine	Drug related
Phenylalanineamide	Unknown
Phenylalanyl-prolyl-arginine	Unknown
Val-pro-pro	Food related
Halofenozide	Unknown
Haloxyfop-P	Drug related
HaptenA	Unknown
Tetranor-PGEM	Unknown
(4E,7E,10E,13E)-Hexadeca-4,7,10,13-tetraenoicacid	Unknown
Hexamethylphosphoramide	Unknown
Hexaminolevulinate	Drug related
Hexenal	Food related
Homoeriodictyol	Food related
HT-2Toxin	Unknown
HydroxyTyrosol-Acetate	Unknown
Hydroxyethylmethacrylicacid	Unknown
Hydroxymethylcimetidine	Unknown
Parthenin	Unknown
Hymenoxon	Unknown
Iduronate2-sulfate	Unknown
Iganidipine	Unknown
Imetit	Unknown
Imexon	Drug related
Indene	Unknown
Indolepropionylglycine	Unknown
Indolophenanthridine	Unknown
Ipazine	Unknown
Iprofenin	Drug related
Islatravir	Drug related
Isobutyl4-hydroxybenzoate	Unknown
Isobutyramide	Unknown

Isoimperatorin	Microbiota; Food related
Isomaltoside	Unknown
Kainicacid	Unknown
Benzamide,3-(3-((2-(3,4-dihydroxyphenyl)ethyl)amino)butyl)-	Unknown
2,2-Difluoro-n-(2-hydroxyethyl)-3-(2-nitro-1h-imidazol-1-yl)propanamide	Unknown
3-Pyridinemethanol,6-amino-alpha-(((1-methyl-4-phenylbutyl)amino)methyl)-	Unknown
L-DOPAn-ButylEster	Unknown
L-Proline,1-acetyl-4-hydroxy-,cis-	Unknown
L-Threoninol	Drug related
L-Valine,N-(2-hydroxy-3-butenyl)-	Unknown
Lenperone	Drug related
LEUCOGENENOL	Unknown
Lignans	Microbiota; Food related; Drug related
Inflatine	Unknown
Lobucavir	Drug related
Lovastatinacid	Unknown
Lavoltidine	Drug related
Lumichrome	Drug related
Lupex	Unknown
Lycorine	Microbiota; Food related
MANNOHEPTULOSE	Food related
MEDICA16	Unknown
Mentholglucuronide	Unknown
Meranzinhydrate	Unknown
Metoprololacid	Unknown
MilbemycinA3	Unknown
Miproxifenephosphate	Unknown
Miserotoxin	Unknown
Mitoflaxone	Unknown
Monoacetyldiglyceride	Unknown
Monoketocholicacid	Unknown
Monopropionylcadaverine	Unknown
Motolimod	Drug related
Mycarose	Unknown
Myxin	Unknown
N-Acetyl-1,6-diaminohexane	Unknown
N-Acetyl-D-tryptophan	Unknown
N-Acetyl-dl-penicillamine	Unknown
N-Allylglycine	Unknown
N-Chlorophenylalanine	Unknown
N-hydroxy-L-tryptophan	Food related
N-Methacryloyl-L-glutamicacid	Unknown

N-Methylaniline	Food related
N-methylsphingosine	Unknown
N-Methylsuccinimide	Unknown
N-OCTANOYL-L-HOMOSERINELACTONE	Unknown
N-octyl-beta-D-thioglucopyranoside	Unknown
N-Phenethylacetamide	Microbiota; Food related
N-propyl-l-arginine	Unknown
N,N-METHYLENEBISACRYLAMIDE	Unknown
N6-Methyl-2-deoxyadenosine	Unknown
N6-SuccinylAdenosine	Unknown
10-Hydroxy-2-oxabicyclo[6.2.2dodeca-1(10),8,11-trien-3-one	Unknown
2-Amino-4-[carbamimidoyl(methyl)aminobutanoicacid	Unknown
3-(4-Hydroxyphenyl)-3,5,6,8-tetrahydro-2H-chromene-4,7-dione	Unknown
Narciclasine	Unknown
L-3-(3-Hydroxy-4-pivaloyloxyphenyl)alanine	Unknown
Neamine	Microbiota; Drug related
Netivudine	Drug related
Nikkomycin	Drug related
Nitenpyram	Drug related
Norcholicacid	Unknown
Noroxyhydrastinine	Unknown
1-Pyrrolidinyloxy,3-(aminocarbonyl)-2,2,5,5-tetramethyl-	Unknown
9-O-Acetylneuraminicacid	Unknown
O-Demethylencainide	Unknown
O-Demethylmetoprolol	Unknown
O-Succinyl-L-homoserine	Microbiota
Olamufloxacin	Unknown
Oxindanac	Unknown
Oxiracetam	Drug related
[3-[2-Aminoethoxy(hydroxy)phosphoryloxy-2-hydroxypropylhexadecanoate	Unknown
1,2-Cyclohexanediol,1-methyl-4-(1-methylethenyl)-	Food related
P-Tolyl-ss-D-glucuronide	Unknown
PaeonimetabolinI	Unknown
Palatinose	Microbiota
Pazufloxacin	Drug related
PC(16:1(9E)/0:0)	Unknown
PC(17:2(9Z,12Z)/0:0)	Unknown
Pelubiprofen	Drug related
Pentaethyleneglycol	Unknown
Pentrinitrol	Drug related
Perylene-1,2-dione	Unknown
ProstaglandinF-mainurinarymetabolite	Unknown
Phellopterin	Food related

Phenacetin	Drug related
Phenazine	Unknown
Phenylacetylglutaminate	Unknown
1-Phenylbiguanide	Unknown
Pheophorbidea	Microbiota; Food related
Pinacidil	Drug related
Piperoxan	Unknown
Plicaticacid	Unknown
Plumieride	Unknown
Policapram	Drug related
6-Amino-N-[6-keto-6-(6-ketohexylamino)hexylhexanamide	Unknown
Polygonal	Unknown
Polyribophosphate	Unknown
2-(4-Amino-1-isopropyl-1H-pyrazolo[3,4-dpyrimidin-3-yl)-1H-indol-5-ol	Unknown
3,17,20-Trihydroxypregnane	Unknown
Pristimerin	Drug related
2-Propyl-7H-purin-6-amine	Unknown
Proscillaridin	Drug related
ProstaglandinM	Unknown
Proxyphylline	Drug related
Pteroicacid	Drug related
Pyridine-3,4-diol	Unknown
PyripyropeneA	Unknown
Pyropheophorbidea	Microbiota; Food related
4-QuinolinecarboxylicAcid	Unknown
Quipazine	Unknown
ResolvinE2	Unknown
Ritipenem	Unknown
Rolafagrel	Unknown
Rosmarinate	Food related; Drug related
Rosmarinicacid	Food related; Drug related
Ruscogenin	Drug related
Ryanodine	Food related
S-(2-Chloroethyl)cysteine	Unknown
S-Nitrosohomocysteine	Unknown
S-Petasin	Unknown
S-Pyruvylglutathione	Unknown
Sabine	Unknown
Salidroside	Food related
Sanfetrinem	Unknown
Sapacitabine	Drug related
Sapienicacid	Unknown
Sarmazenil	Drug related

[3-(2-Aminopropyl)-6-methylidenecyclohexa-1,3-dien-1-ylmethanediol	Unknown
(2S)-4-(2-Aminophenyl)-2-formamido-4-oxobutanoicacid	Unknown
10-(2,3-Dihydroxypropoxy)-10-oxodecanoicacid	Unknown
1-[6-(2-Carboxypyrrolidin-1-yl)-6-oxohexanoylpyrrolidine-2-carboxylicacid	Unknown
2-[(2S)-2,6-Diaminohexanoyloxyaceticacid	Unknown
Ribosyl-5-amino-4-imidazolcarboxamid	Unknown
18-Nitrooctadec-9-enoicacid	Unknown
3-Propyl-3a,4,5,9b-tetrahydro-2H-benzo[g[1,3benzoxazole	Unknown
(2S)-3-Hydroxy-1-[(2S)-pyrrolidine-2-carbonylpyrrolidine-2-carboxylicacid	Unknown
5-[(2R)-2-Aminopropyl-2-hydroxybenzoicacid	Unknown
2-[(2R)-2-Aminopropyl-5-hydroxybenzoicacid	Unknown
[(2S,3R)-2-Amino-3-hydroxybutanoyl(2S)-pyrrolidine-2-carboxylate	Unknown
3-[(2R)-2-Aminopropylindole-1-carboxylicacid	Unknown
(6S)-Tetrahydro-L-biopterin	Unknown
2-(Hydroxymethoxy)aceticacid	Unknown
Carbamoyl(2R)-2,5-diaminopentanoate	Unknown
1-[(2S,5S)-3,4-Dihydroxy-5-(hydroxymethyl)oxolan-2-yl-1,3-diazinane-2,4-dione	Unknown
2-Amino-9-[(2R,4S,5R)-5-(hydroperoxymethyl)-4-hydroxyoxolan-2-yl-1H-purin-6-one	Unknown
Fluorodeoxyuridinemonophosphate	Unknown
[(2S)-6-Amino-1-methoxy-1-oxohexan-2-ylcarbamicacid	Unknown
3-(3-Oxobutanoyloxy)butanoicacid	Unknown
(2S,3S,4S,5R)-3,4,5-Trihydroxy-6-sulfooxyoxane-2-carboxylicacid	Unknown
[2-Oxo-3-(3-oxo-1-phenylbutyl)chromen-4-yl2-acetyloxybenzoate	Unknown
2-Benzyl-2-hydroxybutanamide	Unknown
3-AdenylicAcid	Microbiota; Food related
SCHRADAN	Unknown
N-(3,5-Dichloro-4-hydroxyphenyl)-2-hydroxybenzamide	Unknown
Sec-o-Glucosylhamaudol	Unknown
SenecionineN-oxide	Unknown
SENECIPHYLLINE	Unknown
Sibrafiban	Drug related
Silodosin	Drug related
Simazine	Unknown
SolutolHS15	Unknown
2-Hydroxy-4-[(1R)-1-hydroxy-8-methyl-6-oxononyl-3-methyl-2H-furan-5-one	Unknown
Streptidine	Unknown
SudanI	Unknown

Sulfametrole	Microbiota; Drug related
Cinnamaldehyde	Food related; Drug related; Environment
Sweroside	Unknown
Takinib	Unknown
Tautomycin	Unknown
Tazolol	Unknown
Terbuthylazine	Unknown
TETRAHYDROURIDINE	Drug related
Thienamycin	Microbiota
Thymolblue	Unknown
Tri-glyceride	Unknown
Tributyrylglycerol	Food related; Drug related
TriethylPhosphate	Drug related
Triflumizole	Unknown
TRIMETHYLCOLCHICINICACID	Unknown
Trinexapac-ethyl	Unknown
Tripdiolide	Unknown
Triterpenoids	Unknown
TritonX-100	Food related; Drug related
Tropolone	Unknown
Tyrosinelactate	Unknown
Ultram	Drug related
2-Acetamido-2,6-dideoxygalactose	Unknown
Ureaglutamate	Unknown
Vadocaine	Unknown
Valinelactate	Unknown
VASICINONE	Unknown
Verubecestat	Drug related
Y1Receptorantagonist1	Unknown
Zilascorb	Unknown
1-[(5-Methoxy-2,3-dihydro-1H-indol-3-yl)methylideneamino-2-pentylguanidine	Unknown
4-(1-Hydroxy-2-methoxyethyl)-5-(hydroxymethyl)-2-methylpyridin-3-ol	Unknown
5-(4-Carboxybutylperoxy)pentanoicacid	Unknown
5-Formyl-2H-pyran-6-carboxamide	Unknown
N-[4-[Acetyl(3-aminopropyl)aminobutyl-N-(3-aminopropyl)acetamide	Unknown
MG(20:4(5Z,8Z,11Z,13E)-OH(15S)/0:0/0:0)	Unknown
MG(5-isoPGF2VI/0:0/0:0)	Unknown
MG(18:1(12Z)-2OH(9,10)/0:0/0:0)	Unknown
MG(18:1(9Z)-O(12,13)/0:0/0:0)	Unknown
MG(PGD1/0:0/0:0)	Unknown
PE(20:3(5Z,8Z,11Z)/20:4(5Z,8Z,11Z,14Z)-OH(19S))	Unknown

PE(20:3(5Z,8Z,11Z)/22:6(4Z,7Z,11E,13Z,15E,19Z)-2OH(10S,17))	Unknown
PE(22:6(4Z,7Z,11E,13Z,15E,19Z)-2OH(10S,17)/20:3(5Z,8Z,11Z))	Unknown
PE(20:4(8Z,11Z,14Z,17Z)/20:3(6,8,11)-OH(5))	Unknown
PA(20:3(8Z,11Z,14Z)-2OH(5,6)/12:0)	Unknown
PG(20:1(11Z)/18:1(12Z)-2OH(9,10))	Unknown
PG(i-24:0/20:4(6E,8Z,11Z,13E)-2OH(5S,15S))	Unknown
PE(18:2(9Z,11E)+=O(13)/22:6(4Z,7Z,10Z,13Z,16Z,19Z))	Unknown
PE(20:4(8Z,11Z,14Z,17Z)-2OH(5S,6R)/DiMe(13,5))	Unknown
PC(16:0/18:1(12Z)-O(9S,10R))	Unknown
PC(16:0/18:1(9Z)-O(12,13))	Unknown
PC(18:4(6Z,9Z,12Z,15Z)/18:3(10,12,15)-OH(9))	Unknown
SM(d16:2(4E,8Z)/20:5(6E,8Z,11Z,14Z,17Z)-OH(5))	Unknown
SM(d18:1/PGF2alpha)	Unknown
SM(d18:2(4E,14Z)/PGJ2)	Unknown
SM(d20:1/TXB2)	Unknown
DG(5-isoPGF2VI/0:0/13:0)	Unknown
DG(17:0/PGJ2/0:0)	Unknown
DG(PGJ2/2:0/0:0)	Unknown
DG(2:0/0:0/18:3(10,12,15)-OH(9))	Unknown
DG(20:3(8Z,11Z,14Z)-2OH(5,6)/0:0/a-13:0)	Unknown
DG(TXB2/a-15:0/0:0)	Unknown
DG(a-15:0/0:0/PGD1)	Unknown
DG(i-12:0/PGE2/0:0)	Unknown
Isohydroxymatairesinol	Food related
Ligstroside-aglycone	Food related
Zierin	Food related
GlabrinA	Food related
Parasorbicacid	Food related; Drug related
13(S)-HpOTrE	Food related
13(S)-Hydroperoxylinolenicacid	Food related
12-OPDA	Microbiota; Food related
3-Oxo-2-(2-entenyl)cyclopentaneoctanoicacid	Microbiota; Food related
(-)-Arctiin	Food related
(-)-Olivil	Food related
2-Hydroxycampholonicacid	Food related
Alpha-Campholonicacid	Food related
Isoketocamphoricacid	Food related
12-Hydroxyjasmonicacidglucoside	Food related
(+)-gamma-Hydroxy-L-homoarginine	Food related
Glucolepidiin	Food related
Phaseolotoxin	Food related
Lapachenole	Food related
8-Deoxylactucin	Food related
7-Methylxanthosine	Food related

(1R,2R,4R,5S)-(+)-p-Menthane-2,5-diol	Food related
Mycorradicin	Food related
7-Acetylintermedine	Food related
CarthamosideA1	Food related
9,10-Epoxy-18-hydroxy-octadecanoicacid	Microbiota; Food related
1-Dehydro-[6-gingerdione	Food related
PanaxginsengTetrapeptide	Food related
7-Hydroxy-3,3,4,5,6-pentamethoxyflavone	Food related
Indole-3-aceticAcid	Host; Microbiota; Food related
(1R,4R)-Dihydrocarvone	Food related
Cnidilide	Food related
Isobutylidene	Food related
N-6-Isopent-2-enyl-adenosine	Food related
Nodakenetic	Microbiota; Food related
SenkyunolideN	Food related
9,10-Dihydroxystearicacid	Food related
Alpha-Cephalin	Food related
3-Hexen-1-ol	Food related
Psilostachyin	Food related
Isopropyltartaricacid	Food related
7-Acetyllycopsamine	Food related
Acetyllycopsamine	Food related
Supinine	Food related
Loliolide	Food related
P-Aminobenzaldehyde	Food related
VanillicAcidGlucoside	Food related
Vanilloylglucose	Food related
19-MonoacetylcincassiolA	Food related
Lupinicacid	Food related
Byakangelicol	Food related
Neobyakangelicol	Food related
Arabinogalactose	Food related
5-Hydroxy-4-methoxybisabola-2,10-dien-9-one	Food related
Dodecadienoicacid	Food related
Anisatin	Food related
Heptadecadienoicacid	Food related
Canin	Food related
Artecanin	Food related
Artemorin	Food related
RothinB	Food related
12-oxo-PDA	Food related
(+)-8-Acetoxycarvone	Food related
P-Menthan-trans-2,5-diol	Food related
2-Aminoadipicacid	Host; Microbiota; Food related

L-2-Aminoadipicacid	Host; Microbiota; Food related
Aminoadipicacid	Host; Microbiota; Food related
CalendulosideE	Food related
Halleridone	Food related
(+)-Dehydrovomifoliol	Microbiota; Food related
(11R,12S,13S)-Epoxy-hydroxyoctadeca-cis-9-cis-15-dien-1-oicacid	Food related
D-Alanylglycine	Food related
D-Arabinono-1,4-lactone	Microbiota; Food related
Sativicacid	Food related
Phaseicacid	Food related
Kaempferol3-O-arabinosyl7-O-rhamnoside	Food related
N-Isobutyloctadeca-trans-2-trans-4-dienamide	Food related
1-3-(4,5-dihydrofuranone)-5-(hydroxymethyl)-pyrrole-2-carboxyaldehyde	Food related
Hygrine	Microbiota; Food related
Rishitin	Food related
Risbitin	Food related
Tetracosanedioicacid	Food related
Taraxacine	Food related
4-Hydroxybenzoylglucose	Food related
Gentianine	Food related
Verbenalol	Food related
TuliposideA	Food related
Astringin	Food related
Acetolein	Food related
Acrylamide-sodiumacrylateresin	Food related
Palmiticacid	Host; Food related
(+/-)-1-Hepten-3-ol	Food related
2-(L-Menthoxy)ethanol	Food related; Drug related
L-Monomenthylglutarate	Food related
Lunatone	Food related
Vellein	Food related
3-Phenyl-2-propenenitrile	Food related
3,5-Dihydroxy-6,7-megastigmadien-9-one	Food related
GibberellinA4	Food related
GibberellinA34	Food related
GibberellinA14	Food related
GibberellinA20	Food related
GibberellinA29	Food related
GibberellinA24	Food related
GibberellinA7	Microbiota; Food related
Thymylacetate	Food related
2-Isopropyl-5-methylphenolacetate	Food related
4,4-Dihydroxy-3,3-dimethoxy-9,9-epoxylignan	Food related

EdeticAcid	Food related; Drug related
Vomifoliol	Food related
Malvidin3-rhamnoside	Food related
Epiheterodendrin	Food related
Methyloxindole-3-acetate	Food related
Plastoquinone	Food related
PhaseolosideA	Food related
7-Epi-12-hydroxyjasmonicacid	Microbiota; Food related
1,10-Epoxy-3,8-dihydroxy-4,11(13)-germacradien-12,6-olide	Food related
7-Epijasmonicacid	Food related
3-Oxo-alpha-ionol	Food related
5-Megastigmen-7-yne-3,9-diol	Food related
11b,13-Dihydrolactucin	Food related
Petunidin3-rhamnoside	Food related
Dalbergin	Food related
(+)-marmesin	Food related
(1R,6R)-6-hydroxy-2-succinylcyclohexa-2,4-diene-1-carboxylate	Microbiota; Food related; Drug related
(2E)-2-butylidene-4-hydroxy-5-methyl-3(2H)-furanone	Food related
(3S,5R,6R)-3,5-dihydroxy-6,7-didehydro-5,6-dihydro-12-apo-beta-caroten-12-al	Food related
(3Z)-phytochromobilin	Food related
15-Keto-prostaglandinE2	Host; Food related
(10E,12Z)-(9S)-9-Hydroperoxyoctadeca-10,12-dienoicacid	Food related
(Z)-[(4-hydroxyphenyl)acetaldehydeoxime	Food related
1,2-dibutyrin	Microbiota; Food related
1,3-dihydroxy-N-methylacridone	Food related
2,6-diamino-4-hydroxy-5-formamidopyrimidine	Food related
2-(5-methylthio)pentylmalate	Food related
2-(6-methylthio)hexylmalate	Food related
2-(7-methylthio)heptylmalate	Food related
2-amino-3,7-dideoxy-D-threo-hept-6-ulosonate	Food related
2-oxo-3-phenylpropanoate	Host; Microbiota; Food related
3-(5-methylthio)pentylmalate	Food related
3-(7-methylthio)heptylmalate	Food related
3-dehydro-6-deoxoteasterone	Food related
3-ureido-isobutyrate	Microbiota; Food related
3-beta-D-galactosyl-sn-glycerol	Host; Microbiota; Food related
P-Aminobenzoicacid	Microbiota; Food related; Drug related
4-aminobenzoate	Microbiota; Food related; Drug related
7-methylsulfinylheptylglucosinolate	Food related
CMP-3-deoxy-D-manno-octulosonate	Food related
Coumarylacetate	Food related
D-fructose6-phosphate	Host; Food related
Demethylsuberosin	Microbiota; Food related

Dihydroxyphaseicacid	Food related
GibberellinA34-catabolite	Food related
GibberellinA8-catabolite	Food related
GeranicAcid	Food related
Indole-3-glycolaldehyde	Food related
Isochorismate	Microbiota; Food related; Drug related
Riboprine	Microbiota; Food related; Drug related
Isopentenyladenosine	Microbiota; Food related; Drug related
L-arginino-succinate	Food related
L-histidinol-phosphate	Microbiota; Food related; Drug related
L-quinate	Microbiota; Food related
N5-formyl-N5-hydroxy-L-ornithine	Food related
Caprylicacid	Host; Microbiota; Food related
Octanoate	Host; Microbiota; Food related
Vanillylmandelicacid	Food related
HMBOAtetrahexose	Food related
16-B1-phytoprostane	Food related
9-F1-phytoprostane	Food related
Glutamicacid-betaxanthin	Food related
Linalool(8-hydroxydihydro-)	Unknown
ProcyanidindimerB7	Unknown
Gamma-Aminobutyricacid-betaxanthin	Food related
Histidinyl-Asparagine	Food related
L-Leucyl-L-Alanine	Food related
Ile-Ile-Ile-Pro	Food related
Leu-Leu-Tyr	Food related
Lys-Asp-Tyr	Food related
Pretyrosine	Microbiota; Food related
Pro-Ile	Food related
11b-Hydroxyprogesterone	Host; Food related
Lysozyme	Food related
Selumetinib	Drug related
4-Hydroxyphenylaceticacidsulfate	Unknown
Indolecarboxylicacidsulfate	Unknown
3-Acetylphenolsulfate	Unknown
AminocatecholN-acetatesulfate	Unknown
3-Methyloxindole	Unknown
N-Acetyl-DL-Leucine	Drug related
6-Hydroxy-4-Methylcoumarin	Unknown
N-Acetyl-DL-Methionine	Unknown
PE(13:0/0:0)	Unknown
PE(12:0/0:0)	Unknown
PE(16:0/0:0)	Microbiota; Food related
PC(18:2/0:0)	Unknown

6,10-Dimethyl-5(E),9-undecadien-2-one	Unknown
13Z-Docosenamide	Unknown
8(S)-HETrE	Unknown
20-trifluoro-LTB4	Unknown
9S,10S,11R-trihydroxy-12Z-octadecenoicacid	Unknown
10-hydroxy-2E-decenoicacid	Unknown
3-oxo-2-propyl-1,4-dihydroisoquinoline-1-carboxylicacid	Unknown
6-Methylthioguanine	Unknown
N-Methyl-L-Threonine	Unknown
2,3-dinorProstaglandinE1	Unknown
Dioctylsuccinate	Unknown
PG(16:0/0:0)[U	Unknown
Tributylcitrate	Unknown
4-Oxododecanedioicacid	Unknown
ValLeuGly	Unknown
GPEtn(16:0/12:0)	Unknown
N-(3-acetylphenyl)-4,5,6,7-tetrahydro-1-benzothiophene-3-carboxamide	Unknown
3-Phenyl-1-(2,4,6-trihydroxyphenyl)propan-1-one	Unknown
5beta-CHOLANICACID-3alpha,12alpha-DIOLN-(2-SULPHOETHYL)-AMIDE	Unknown
ValVal	Unknown
4,4-DIMETHOXYDALBERGIONE	Unknown
2,3-dinor-8-iso-PGF2a	Unknown
3-Deoxyuridine	Unknown
2,2-(3-methylcyclohexane-1,1-diyl)diaceticacid	Unknown
IlePhe	Unknown
(S)-(4-Hydroxy-phenyl)-(S)-piperidin-2-yl-aceticacidmethylesterhydrochloride	Unknown
P-TolylSulfate	Unknown
LysVal	Unknown
LeuProAsp	Unknown
N-(5-acetamidopentyl)acetamide	Unknown
2-ISOPRENYL-3-HYDROXY-5-METHYL-a-PYRONE	Unknown
ValIleIle	Unknown
Metazin	Unknown
3-Methyl-N-phenylaniline	Unknown
5-Methyl-3-Deoxyuridine	Unknown
4-hydroxyNonenal	Food related
N-cyclopentyl-1-(3,4-dimethoxyphenyl)sulfonylpiperidine-3-carboxamide	Unknown
P-Acetaminobenzaldehyde	Unknown
CyclicCMP	Unknown
1-Ethyl-9H-pyrido[3,4-bindole	Unknown

LeuProThr	Unknown
Dimethyl(R)-pyrrolidine-1,2-dicarboxylate	Unknown
ValIleGlu	Unknown
Nalpha-Acetyl-L-arginine	Unknown
GluLeu	Unknown
DodecylHydrogenSulfate	Unknown
GlnIleGlu	Unknown
LeuLys	Unknown
PheAla	Unknown
3-Phenyl-3-(pyridin-2-yl)propanoicAcid	Unknown
N-Carbobenzyloxyleucine	Unknown
PE(15:1/0:0)	Unknown
PE(14:1/0:0)	Unknown
LeuValAla	Unknown
IleValIle	Unknown
(2-oxo-2,3-Dihydro-1H-Indol-3-yl)aceticAcid	Unknown
LeuTrp	Unknown
ValGlu	Unknown
4,5-Dihydroxy-3-Propylcyclopent-2-EN-1-ONE	Unknown
3-(6-methyl-2-oxo-1H-quinolin-3-yl)propanoicacid	Unknown
(E)-2-UndecenedioicAcid	Unknown
ValValIle	Unknown
IleGly	Unknown
(1S)-(-)-CamphanicAcid	Unknown
LysLeu	Unknown
IleLys	Unknown
GlnIle	Unknown
Lauryldiethanolamine	Unknown
IleValGlu	Unknown
GlyLeuLeu	Unknown
3-Methyl-1-phenyl-1-butanone	Unknown
ValLeuAla	Unknown
IleValGly	Unknown
1-Methylpseudouridine	Unknown
Ent-16beta,17-Dihydroxy-19-KauranoicAcid	Unknown
DiethylDiallylmalonate	Unknown
GlyPhe	Unknown
4-fluoro-N,3-dimethyl-N-(oxan-4-ylmethyl)benzenesulfonamide	Unknown
GlyLysLeu	Unknown
4-Decan-4-YlbenzenesulfonicAcid	Unknown
CR2249	Unknown
1-Octylpyrrolidin-2-one	Unknown
1,2,3-trimethylindole-5-carboxamide	Unknown
4-(2-chloroanilino)-4-oxobutanoicacid	Unknown

ORTHOTHYMOTINICACID	Unknown
PE(16:1/0:0)	Unknown
1-(4-ethoxyphenyl)sulfonyl-N-(oxolan-2-ylmethyl)piperidine-4-carboxamide	Unknown
GlnVal	Unknown
(11E,15Z)-9,10,13-Trihydroxy-11,15-octadecadienoicacid	Unknown
2-Methylindoline	Unknown
Piperidine-4-Carboxamide	Unknown
Butylisopropylamine	Unknown
HisThrLys	Unknown
4-Hydroxyisoleucine	Food related
13,14-dihydroProstaglandinF1alpha	Unknown
3-ethyl-N-propan-2-yl-[1,2,4triazolo[4,3-bpyridazin-6-amine	Unknown
GliocladicAcid	Unknown
9-Methoxycarbonyldec-9-EnoicAcid	Unknown
1a,1b-dihomoPGF2alpha	Unknown
CoreyPG-LactoneDiol	Unknown
3-(4-Hydroxy-3-methoxyphenyl)propionicacid	Unknown
(R)-2-HydroxystearicAcid	Unknown
Bufexamac	Drug related
Walleminone	Unknown
Lys-Lys-OH	Unknown
CHEBI:69439	Unknown
4-Dodecylmorpholine	Unknown
1,4-CyclohexanedicarboxylicAcid	Unknown
Isobutyrylcarnitine	Food related
R-(-)-Mandelicacid	Unknown
LactarorufinA	Unknown
5-Megastigmene-3,9-Diol	Unknown
Laminine	Unknown
3-Pyridinol	Unknown
3-Methylpyridazine	Unknown
6,18,19-Trihydroxytrachyloban-2-one	Unknown
3,4-(Methylenedioxy)acetophenone	Unknown
Propenyl-L-NIO	Unknown
N-Acetyl-DL-Tryptophan	Unknown
(11xi)-4,7-Dihydroxy-12,13-Epoxytrichothec-9-en-8-one	Unknown
3-IsobutylglutaricAcid	Unknown
N-(2,4-Dimethylphenyl)formamide	Unknown
Triethylcitrate	Unknown
4-[3-[1-(4-methylphenyl)-5-oxopyrrolidin-3-yl-1,2,4-oxadiazol-5-ylbenzonitrile	Unknown
Furan-2-yl-(7-propan-2-yl-9-oxa-3,7-diazabicyclo[3.3.1nonan-3-yl)methanone	Unknown

4-Methyl-5-oxo-2-pentyl-2,5-dihydro-3-furancarboxylicacid	Unknown
LEVULINICACID,3-BENZYLIDENYL-	Unknown
PheGly	Unknown
5-fluoro-2-methoxy-N-[(4-methyloxan-4-yl)methylbenzenesulfonamide	Unknown
N-(3,5-dimethylphenyl)-2-(5-pyrazin-2-yl-1,3,4-oxadiazol-2-yl)acetamide	Unknown
2,6-Dimethylmorpholine	Unknown
4-Hydroxy-3,6,9-Trimethyl-3,3a,4,5,9a,9b-hexahydroazuleno[4,5-bfuran-2,7-Dione	Unknown
Delta-Tridecanolactone	Unknown
4-Methylpyrimidine	Unknown
12a-Hydroxy-5-Deoxydehydromunduserone	Unknown
Cis-Chrysanthemol	Unknown
4,6-Dihydroxy-3-(1-Hydroxyethyl)-5-Methoxy-3H-2-Benzofuran-1-One	Unknown
Euparin	Unknown
Azacyclotridecan-2-one	Unknown
N-Dodecanoyl-DL-HomoserineLactone	Unknown
N-(2-Hydroxy-3-methylbutanoyl)leucine	Unknown
SerVal	Unknown
LeuLysAlaValVal	Unknown
IleTrp	Unknown
ArgLeu	Unknown
2-Aminopropanol	Unknown
3-[(1-acetyl-3,4-dihydro-2H-quinolin-6-yl)sulfonyl-N-(4-ethylphenyl)propanamide	Unknown
MonensinA	Drug related
SALVINORINA	Unknown
8-Hydroxyquinoline	Drug related
Cycluron	Unknown
Dinoterb	Unknown
(+)-Aschantin	Unknown
9(S)-HpOTrE	Unknown
13(S)-HODE	Microbiota; Food related; Drug related
Beta-Zearalanol	Drug related
3-tert-Butylphenol	Unknown
Flindersine	Unknown
Encecalin	Unknown
2-Hydroxyquinoline	Drug related
N-Acetyl-D-phenylalanine	Microbiota
3-Hydroxybenzaldehyde	Microbiota
(S,S)-Butane-2,3-diol	Microbiota
SinapoylMalate	Unknown

2-Aminophenol	Microbiota; Drug related
Scytalone	Unknown
Gentiobiose	Microbiota
Spongothymidine	Unknown
3,5-CyclicAMP	Host; Microbiota; Food related; Drug related
[3-Oxo-2-(2-penten-1-yl)cyclopentylaceticAcid	Unknown
AleuriticAcid	Unknown
PE(14:0/0:0)	Unknown
PC(18:1/0:0)	Unknown

Table S12. Gene annotation results for host SNPs associated with 6-hydroxymelatonin.

Location	Allele	Consequence	SYMBOL	Gene	Existing_variation	REF_ALLELE
1:10440052-10440052	A	intron_variant	APP	ENSBTAG00000017753	rs110760396	G
1:10440052-10440052	A	intron_variant	APP	ENSBTAG00000017753	rs110760396	G
1:10440052-10440052	A	intron_variant	APP	ENSBTAG00000017753	rs110760396	G
1:10440052-10440052	A	intron_variant	APP	ENSBTAG00000017753	rs110760396	G
1:10440052-10440052	A	intron_variant	APP	ENSBTAG00000017753	rs110760396	G
1:10440052-10440052	A	intron_variant	APP	ENSBTAG00000017753	rs110760396	G
1:15691917-15691917	A	intron_variant	NCAM2	ENSBTAG00000003301	rs42381629	G
1:15692062-15692062	A	intron_variant	NCAM2	ENSBTAG00000003301	rs42381627	T
1:107909436-107909436	C	intron_variant	IQCJ	ENSBTAG00000003135	-	T
1:107909436-107909436	C	intron_variant	IQCJ	ENSBTAG00000003135	-	T
11:25313911-25313911	G	intergenic_variant	-	-	-	A
11:97063413-97063413	C	intergenic_variant	-	-	-	T
11:101731034-101731034	T	intron_variant	RAPGEF1	ENSBTAG00000020791	-	G
11:101731034-101731034	T	intron_variant	RAPGEF1	ENSBTAG00000020791	-	G
11:101731034-101731034	T	intron_variant	RAPGEF1	ENSBTAG00000020791	-	G
11:101731034-101731034	T	intron_variant	RAPGEF1	ENSBTAG00000020791	-	G
11:101731034-101731034	T	intron_variant	RAPGEF1	ENSBTAG00000020791	-	G
11:103619903-103619903	C	upstream_gene_variant	TMEM250	ENSBTAG00000046939	-	T
11:103619903-103619903	C	downstream_gene_variant	-	ENSBTAG00000066229	-	T
11:103619903-103619903	C	downstream_gene_variant	-	ENSBTAG00000066229	-	T
11:103619903-103619903	C	downstream_gene_variant	-	ENSBTAG00000066229	-	T
11:103619903-103619903	C	downstream_gene_variant	-	ENSBTAG00000066229	-	T
11:103619903-103619903	C	downstream_gene_variant	-	ENSBTAG00000066229	-	T
12:4526567-4526567	A	intergenic_variant	-	-	-	G
12:28170430-28170430	A	intergenic_variant	-	-	-	G
12:37424384-37424384	A	intergenic_variant	-	-	rs132639337	T
13:6343060-6343060	T	intergenic_variant	-	-	-	C
14:67023575-67023575	A	intron_variant	CPQ	ENSBTAG00000011908	-	G
14:67023575-67023575	A	downstream_gene_variant	bta-mir-584-1	ENSBTAG00000030067	-	G
14:67023575-67023575	A	intron_variant	CPQ	ENSBTAG00000011908	-	G
15:31237276-31237276	C	intron_variant	GRIK4	ENSBTAG00000017146	-	G
16:75228940-75228940	T	intron_variant	PLXNA2	ENSBTAG00000001173	-	C
16:75228940-75228940	T	intron_variant	PLXNA2	ENSBTAG00000001173	-	C
16:75228940-75228940	T	intron_variant	PLXNA2	ENSBTAG00000001173	-	C
16:75229446-75229446	A	intron_variant	PLXNA2	ENSBTAG00000001173	-	C
16:75229446-75229446	A	intron_variant	PLXNA2	ENSBTAG00000001173	-	C
16:75229446-75229446	A	intron_variant	PLXNA2	ENSBTAG00000001173	-	C
16:75229459-75229459	G	intron_variant	PLXNA2	ENSBTAG00000001173	-	A
16:75229459-75229459	G	intron_variant	PLXNA2	ENSBTAG00000001173	-	A
16:75229459-75229459	G	intron_variant	PLXNA2	ENSBTAG00000001173	-	A
16:75229472-75229472	A	intron_variant	PLXNA2	ENSBTAG00000001173	-	G

16:75229472-75229472	A	intron_variant	PLXNA2	ENSBTAG00000001173	-	G
16:75229472-75229472	A	intron_variant	PLXNA2	ENSBTAG00000001173	-	G
16:75229551-75229551	A	intron_variant	PLXNA2	ENSBTAG00000001173	-	G
16:75229551-75229551	A	intron_variant	PLXNA2	ENSBTAG00000001173	-	G
16:75229551-75229551	A	intron_variant	PLXNA2	ENSBTAG00000001173	-	G
17:5574496-5574496	A	intergenic_variant	-	-	rs133762551	G
18:51437487-51437487	T	downstream_gene_variant	-	ENSBTAG00000068662	-	C
19:51650901-51650901	C	intron_variant	CHMP6	ENSBTAG00000038745	-	T
20:802045-802045	C	intron_variant	SLIT3	ENSBTAG00000017746	-	T
20:802045-802045	C	intron_variant	SLIT3	ENSBTAG00000017746	-	T
20:802045-802045	C	intron_variant	SLIT3	ENSBTAG00000017746	-	T
22:5127799-5127799	G	intron_variant	TGFBR2	ENSBTAG00000019832	-	A
22:5127799-5127799	G	intron_variant	TGFBR2	ENSBTAG00000019832	-	A
22:5127799-5127799	G	intron_variant	TGFBR2	ENSBTAG00000019832	-	A
23:20022-20022	A	intergenic_variant	-	-	-	T
23:20031-20031	C	intergenic_variant	-	-	-	G
23:7854795-7854795	T	intron_variant	IP6K3	ENSBTAG00000033603	rs134032824	C
23:8811362-8811362	T	intron_variant	BLTP3A	ENSBTAG00000009267	-	C
23:8811362-8811362	T	regulatory_region_variant	-	-	-	C
24:20722-20722	A	intergenic_variant	-	-	-	G
25:11197-11197	A	intron_variant,non_coding_transcript_variant	-	ENSBTAG00000078224	-	T
25:11197-11197	A	intron_variant,non_coding_transcript_variant	-	ENSBTAG00000078224	-	T
25:11197-11197	A	non_coding_transcript_exon_variant	-	ENSBTAG00000078224	-	T
25:11197-11197	A	intron_variant,non_coding_transcript_variant	-	ENSBTAG00000078224	-	T
25:11197-11197	A	upstream_gene_variant	-	ENSBTAG00000078224	-	T
28:883065-883065	T	intergenic_variant	-	-	rs378266957	C
28:931577-931577	G	intergenic_variant	-	-	rs3423571019	A
3:41695201-41695201	C	intron_variant,non_coding_transcript_variant	-	ENSBTAG00000058967	-	T
4:23548707-23548707	A	intron_variant	AGMO	ENSBTAG00000034154	-	G
5:106922087-106922087	T	intron_variant	NRIP2	ENSBTAG00000007611	-	C
5:106922131-106922131	T	intron_variant	NRIP2	ENSBTAG00000007611	-	C
5:106922164-106922164	G	intron_variant	NRIP2	ENSBTAG00000007611	-	A
5:106924610-106924610	A	downstream_gene_variant	ITFG2	ENSBTAG00000007609	-	T
5:106924610-106924610	A	intron_variant	NRIP2	ENSBTAG00000007611	-	T
5:106924610-106924610	A	downstream_gene_variant	ITFG2	ENSBTAG00000007609	-	T
5:106926534-106926534	T	downstream_gene_variant	ITFG2	ENSBTAG00000007609	-	G
5:106926534-106926534	T	synonymous_variant	NRIP2	ENSBTAG00000007611	-	G
5:106926534-106926534	T	downstream_gene_variant	ITFG2	ENSBTAG00000007609	-	G
5:116745636-116745636	G	synonymous_variant	CELSR1	ENSBTAG00000008036	-	T
6:95774148-95774148	G	intergenic_variant	-	-	-	A
7:104299058-104299058	T	intergenic_variant	-	-	rs3423250727	G
7:106716301-106716301	G	intron_variant	EFNA5	ENSBTAG00000016515	rs43534465	A
7:106716301-106716301	G	intron_variant	EFNA5	ENSBTAG00000016515	rs43534465	A
8:15128273-15128273	C	intron_variant,non_coding_transcript_variant	-	ENSBTAG00000077780	-	T

8:15128273-15128273	C	intron_variant,non_coding_transcript_variant	-	ENSBTAG00000077780	-	T
8:15128273-15128273	C	intron_variant,non_coding_transcript_variant	-	ENSBTAG00000077780	-	T
8:15128273-15128273	C	intron_variant,non_coding_transcript_variant	-	ENSBTAG00000077780	-	T
9:44111308-44111308	A	intergenic_variant	-	-	rs209336970	G
9:44427165-44427165	G	intergenic_variant	-	-	-	A
X:28969015-28969015	T	regulatory_region_variant	-	-	-	C
X:28969015-28969015	T	intergenic_variant	-	-	-	C
X:28969042-28969042	G	regulatory_region_variant	-	-	-	A
X:28969042-28969042	G	intergenic_variant	-	-	-	A
X:28969055-28969055	T	regulatory_region_variant	-	-	-	C
X:28969055-28969055	T	intergenic_variant	-	-	-	C
X:28969077-28969077	A	regulatory_region_variant	-	-	-	G
X:28969077-28969077	A	intergenic_variant	-	-	-	G
X:28969086-28969086	C	regulatory_region_variant	-	-	-	T
X:28969086-28969086	C	intergenic_variant	-	-	-	T
X:28969126-28969126	C	regulatory_region_variant	-	-	-	G
X:28969126-28969126	C	intergenic_variant	-	-	-	G
X:51881972-51881972	A	intron_variant,non_coding_transcript_variant	-	ENSBTAG00000072626	-	T
X:64608433-64608433	G	intergenic_variant	-	-	-	A
